# Genomic Architecture of Rapid Parallel Adaptation to Fresh Water in a Wild Fish

**DOI:** 10.1093/molbev/msaa290

**Published:** 2020-11-04

**Authors:** Shao-Bing Zong, Yu-Long Li, Jin-Xian Liu

**Affiliations:** 1 CAS Key Laboratory of Marine Ecology and Environmental Sciences, Institute of Oceanology, Chinese Academy of Sciences, Qingdao, China; 2 Laboratory for Marine Ecology and Environmental Science, Qingdao National Laboratory for Marine Science and Technology, Qingdao, China; 3 Center for Ocean Mega-Science, Chinese Academy of Sciences, Qingdao, China; 4 University of Chinese Academy of Sciences, Beijing, China

**Keywords:** parallel evolution, local adaptation, chromosome inversion, complex traits, standing genetic variation

## Abstract

Rapid adaptation to novel environments may drive changes in genomic regions through natural selection. However, the genetic architecture underlying these adaptive changes is still poorly understood. Using population genomic approaches, we investigated the genomic architecture that underlies rapid parallel adaptation of *Coilia nasus* to fresh water by comparing four freshwater-resident populations with their ancestral anadromous population. Linkage disequilibrium network analysis and population genetic analyses revealed two putative large chromosome inversions on LG6 and LG22, which were enriched for outlier loci and exhibited parallel association with freshwater adaptation. Drastic frequency shifts and elevated genetic differentiation were observed for the two chromosome inversions among populations, suggesting that both inversions would undergo divergent selection between anadromous and resident ecotypes. Enrichment analysis of genes within chromosome inversions showed significant enrichment of genes involved in metabolic process, immunoregulation, growth, maturation, osmoregulation, and so forth, which probably underlay differences in morphology, physiology and behavior between the anadromous and freshwater-resident forms. The availability of beneficial standing genetic variation, large optimum shift between marine and freshwater habitats, and high efficiency of selection with large population size could lead to the observed rapid parallel adaptive genomic change. We propose that chromosomal inversions might have played an important role during the evolution of rapid parallel ecological divergence in the face of environmental heterogeneity in *C. nasus*. Our study provides insights into the genomic basis of rapid adaptation of complex traits in novel habitats and highlights the importance of structural genomic variants in analyses of ecological adaptation.

## Introduction

Understanding the mechanisms that facilitate adaptation of populations to changing environments is a long-standing central goal of evolutionary biology (Savolainen et al. 2013). Although populations can evolve rapidly in response to sudden environmental changes (Lescak et al. 2015; Reid et al. 2016), genetic architecture of rapid adaptation is still not well understood for complex traits controlled by a large number of genetic and environmental influences (Jain and Stephan 2017). Although empirical population genetics supports rapid adaptation as a result of selective sweep with large frequency changes of novel mutations at single or few loci, quantitative genetics presumes that phenotypic adaptation results from subtle allele frequency shifts at many loci (Pritchard and Di Rienzo 2010). Owing to recent advances in genomic technologies, emerging evidence suggests that structural genomic variants (SVs) of diverse forms are taxonomically ubiquitous and play a major role in a multitude of ecological and evolutionary processes (Mérot et al. 2020). SVs are predicted to be favored under adaptation and are expected to change the evolutionary trajectory of polygenic traits under selection because they involve many genes acting together like supergenes of large effect, rather than many loci of small effect (Oomen et al. 2020). Mounting evidence shows that chromosomal inversions are the most frequent SVs associated with adaptive phenotypes and have a pervasive role in eco-evolutionary processes, from mating systems, environmental adaptation, and reproductive isolation to speciation (Wellenreuther and Bernatchez 2018).

Evolutionary transitions from marine to freshwater environments are important in generating phyletic diversity within fishes (Vega and Wiens 2012). Transitions from marine to freshwater habitats constitute dramatic shifts between “adaptive zones,” which represent considerable adaptive challenges because many environmental conditions vary (e.g., salinity, oxygen, pH, food, predators, pathogens, symbionts, etc.) (Lee and Bell 1999). Adaption to fresh water likely involves multiple highly polygenic traits that contribute to the complex adaptive phenotype (Brennan et al. 2018). The Japanese grenadier anchovy, *Coilia nasus*, exists in two distinct life history forms (Yuan et al. 1976)*.* One is an ancestral anadromous form, widely distributed in coastal and estuarial regions of the Northwest Pacific, which migrates upstream into fresh water in the spring for breeding. The other is a landlocked freshwater-resident form spending its whole life in the affiliated lakes in lower reaches of the Yangtze River in China, such as Taihu Lake, Chaohu Lake, and Hongze Lake, and serving as the most dominant species in the lake ecosystem (Yuan et al. 1976; Cheng et al. 2019). A recent study demonstrates that the freshwater-resident form is not genetically distinguishable from the anadromous form, and had invaded freshwater environment after the formation of the lakes in late Holocene (Cheng et al. 2019). As a result, the freshwater-resident and anadromous *C. nasus* in the Yangtze River watershed are generally each other’s closest relatives. Because of the varied selection regimes in freshwater habitats, the derived freshwater-resident populations have consistently acquired a specific set of morphological, physiological, and behavioral traits allowing them to reside in fresh water, indicating rapid phenotypic changes in adaptation to fresh water (Yuan et al. 1976). Besides the complex combination of behavioral traits associated with migration, the anadromous and resident forms differ in an array of morphological traits, including number of vertebrae, number of soft rays of anal fin, eye size, shape and size of liver, ovary shape, and body color (Yuan et al. 1976). Furthermore, helminth community, muscle lipid content, and feeding behavior at spawning season are also different between the two forms (Yuan et al. 1976; Li and Wang 2014). These parallel phenotypic changes may have the same underlying genetic basis or may involve different genetic changes. Therefore, this natural system provides a compelling opportunity to infer genetic architecture of rapid phenotypic adaptation by comparing sets of derived freshwater populations with their anadromous ancestors.

Here, we seek to investigate genomic architecture that underlies rapid parallel adaptation to fresh water in *C. nasus* by comparing four freshwater-resident populations with their anadromous ancestors in the Yangtze River system. We performed joint analysis of single nucleotide polymorphisms (SNPs) and SVs in a population genomic framework to characterize how differentiation is structured across the genome, which would allow us to assess whether the same chromosomal features are implicated in divergence between the anadromous population and the four freshwater-resident populations from different lakes. The source of adaptive genetic variants for freshwater adaptation was also illustrated. The results will provide a general understanding of the genetics of rapid adaptation of complex phenotypes to changing environment in wild populations.

## Results

### Genetic Variation, *N*_e_ Estimation, Population Structure, and Demographic History

The published chromosome-level genome assembly for *C. nasus* (Xu et al. 2020) contains a total of 81,894 *Eco*RI restriction sites (one per 10 kb) across 24 linkage groups (chromosomes). The RAD sequencing covered about 110 Mb (14%) of the total genome with a size of 812 Mb (supplementary fig. S1, Supplementary Material online). After comparison with the reference genome, 6,542,393 SNPs were called. A total of 123,792 SNPs were retained after the filtering procedure, which were distributed evenly across the genome with a mean depth of 20× (supplementary fig. S2 and table S1, Supplementary Material online) and covered ∼65% of the whole genome when considering a moderate linkage disequilibrium (LD) extent of 10 kb. There was an average of 5,158 SNPs per chromosome with a minimum of 3,204 SNPs on LG20 and a maximum of 8,071 SNPs on LG14. Most (85%) of the SNPs were within 10 kb from their nearest neighbors (supplementary fig. S3, Supplementary Material online). The observed and expected heterozygosity values were similar among populations, with *H*_O_ ranged from 0.21 to 0.23 and *H*_E_ ranged from 0.20 to 0.24 (supplementary table S2, Supplementary Material online). The *N*_e_ estimate for the Yangtze River Estuary anadromous population was 12,753 (95% CI: 8,735–23,614), which was much larger than those in the four freshwater-resident populations. In addition, the *N*_e_ estimates of Taihu Lake population (3,494; 95% CI: 3,163–3,904) and Chaohu Lake population (3,492; 95% CI: 3,195–3,850) were a magnitude higher than those of Hongze Lake population (171; 95% CI: 170–172) and Luoma Lake population (160; 95% CI: 160-161) (supplementary table S2, Supplementary Material online).

**Fig. 1.**
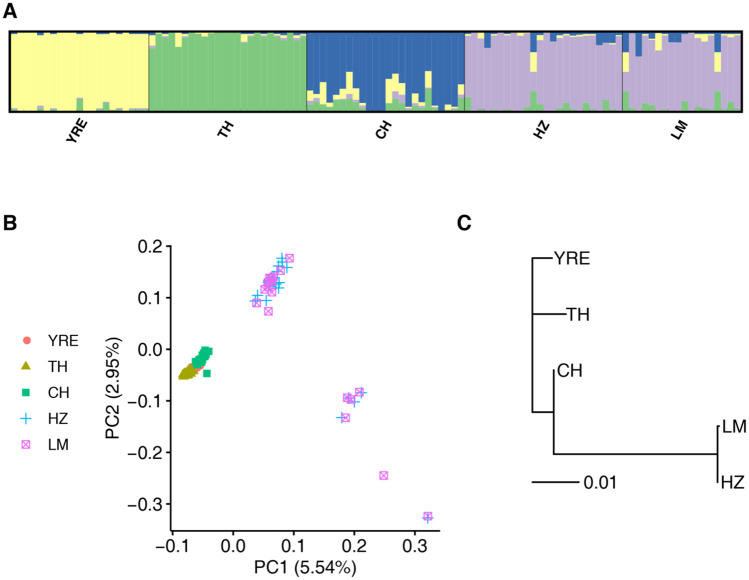
Population genetic structure based on neutral loci for one anadromous and four freshwater-resident populations. (*A*) Admixture plot for *K* = 4. (*B*) PCA showing the clustering of individuals. First two PCs are shown, each individual is represented by one symbol and color label corresponds to its population of origin. (*C*) NJ tree based on Weir and Cockerham’s *F*_ST_ values between population pairs. YRE, Yangtze River Estuary; TH, Taihu Lake; CH, Chaohu Lake; HZ, Hongze Lake; LM, Luoma Lake.

The genome-wide fixation indexes (*F*_ST_) between the anadromous population and the four freshwater-resident populations were generally low but significant, with an average value of 0.07 for all SNPs and 0.03 for neutral SNPs, indicating that the freshwater populations were recently derived from the anadromous one (supplementary table S3, Supplementary Material online). Admixture results indicated that genetic variation was strongly partitioned by geography with highest support for *K* = 4 (Hongze Lake and Luoma Lake formed one cluster and other three populations formed distinct clusters) (fig. 1*A* and supplementary fig. S4, Supplementary Material online), which was consistent with neighbor-joining (NJ) tree based on *F*_ST_ (fig. 1*C*). Principal component analysis (PCA) based on the first two PCs indicated that individuals from Hongze Lake and Luoma Lake were distinct from the other populations, and individuals from Chaohu Lake and Taihu Lake also formed two closely related clusters, whereas individuals from Yangtze River Estuary population were overlapped with those from Chaohu Lake and Taihu Lake (fig. 1*B*).

The demographic analysis indicated that scenario 4 was the best model, in which the freshwater-resident populations were derived independently from their common ancestral anadromous population (supplementary fig. S5, Supplementary Material online). The posterior probability of scenario 4 was significantly higher than those of the other scenarios (posterior probability = 1.00, 95% CI: 0.99–1.00; supplementary table S4, Supplementary Material online). The Hongze Lake population was derived from the Yangtze River Estuary population first, which happened about 365 (95% CI: 105–1,100) generations ago. Then the Chaohu Lake population and Taihu Lake population formed in sequence, which happened about 83 (95% CI: 48–120) and 61 (95% CI: 32–96) generations ago, respectively (supplementary table S5, Supplementary Material online).

### Candidate Outlier SNPs and Parallel Allele Frequency Shifts

**Fig. 2.**
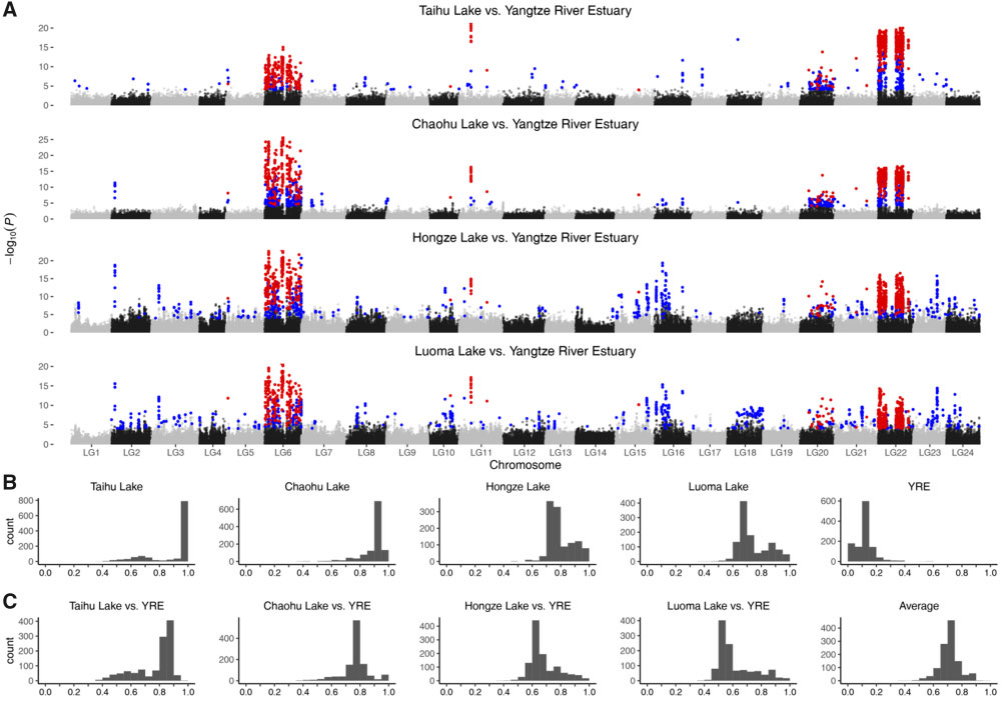
(*A*) Manhattan plot showing the distribution of candidate outlier SNPs for the four freshwater–anadromous population pairs. *Y*-axes represent *P* values calculated by FET. Dots in red represent the outliers detected by both FET and pcadapt methods and are shared among the four population pairs. Dots in blue represent outliers detected by both FET and pcadapt methods, but are only in one population pair. (*B*) Distribution of the frequency of the FWA for the 1,147 candidate outlier SNPs in five populations. (*C*) Allele frequency increment of the FWA for the 1,147 candidate outlier SNPs in the four freshwater–anadromous population pairs and their means.

The two methods of outlier detection identified a total of 16,960 outlier loci across four population pairs, of which 2,814 were detected by both methods (fig. 2*A*, supplementary fig. S6 and table S6, Supplementary Material online). Fisher’s exact test (FET) identified a total of 5,835 outlier SNPs, of which 1,269 were shared among all four population pairs. Pcadapt detected a total of 13,939 outlier SNPs, of which 1,188 were shared among all population pairs. The number of outlier SNPs detected by both methods in each population pair ranged from 1,568 in Taihu Lake versus Yangtze River Estuary to 1,849 in Luoma Lake versus Yangtze River Estuary. At last, a total of 1,147 outlier SNPs detected by both methods were shared among the four population pairs. Most (96%) of the 1,147 candidate outlier SNPs were preferentially distributed on chromosomes LG6 (263) and LG22 (838), clustering into similar genomic islands among population pairs (fig. 2*A*). The rest 46 outlier SNPs were located on six chromosomes, with an average of eight SNPs on each chromosome. These results suggested that genomic regions exhibiting signatures of selection were remarkably consistent across multiple, independently derived freshwater populations, indicating that parallel phenotypic evolution in *C. nasus* may be occurring through extensive parallel genetic evolution.

Large allele frequency shifts were observed for the freshwater favored allele (FWA) of the 1,147 candidate SNPs in all freshwater–anadromous population pairs (fig. 2*B* and *C*). The FWA of most candidate SNPs was nearly fixed in the freshwater populations. The average frequency of the FWA was 0.89 in Taihu Lake and Chaohu Lake, which was higher than in Hongze Lake (0.80) and Luoma Lake (0.74) (fig. 2*B*). In contrast, the frequency of FWA in the anadromous population was generally low with an average of 0.12 (SD = 0.07). Frequency of the FWA of most candidate outlier SNPs had increased by at least 0.5 in different freshwater populations (94–99%), with an average frequency increment across all candidate outlier SNPs ranging from 0.62 in Luoma Lake to 0.77 in Chaohu Lake and Taihu Lake (fig. 2*C*). Both the frequency and the increment of frequency for the FWA were higher in Taihu Lake and Chaohu Lake than those in Hongze Lake and Luoma Lake, which was consistent with their estimates of *N*_e_.

The FWA for 1,024 of the 1,147 candidate outlier SNPs (89%) occurred as standing genetic variation in the Yangtze River Estuary anadromous population, suggesting that standing variation was the predominant source for adaptation to fresh water. Considering the relatively low frequency of the FWA as standing variations in the anadromous population and the relatively small sample size, it was not unlikely that FWA of the other 11% candidate SNPs could also present as standing variation in the anadromous population. In the anadromous population, frequency of the FWA for the candidate SNPs (average = 0.12, SD = 0.07) was significantly higher than the minor allele frequency (MAF) of the neutral SNPs (average MAF = 0.11, SD = 0.10; Welch two-sample *t*-test *P *<* *2.2e-16; supplementary fig. S7, Supplementary Material online).

### Putative Chromosome Inversion Regions Related to Parallel Freshwater Adaptation

**Fig. 3.**
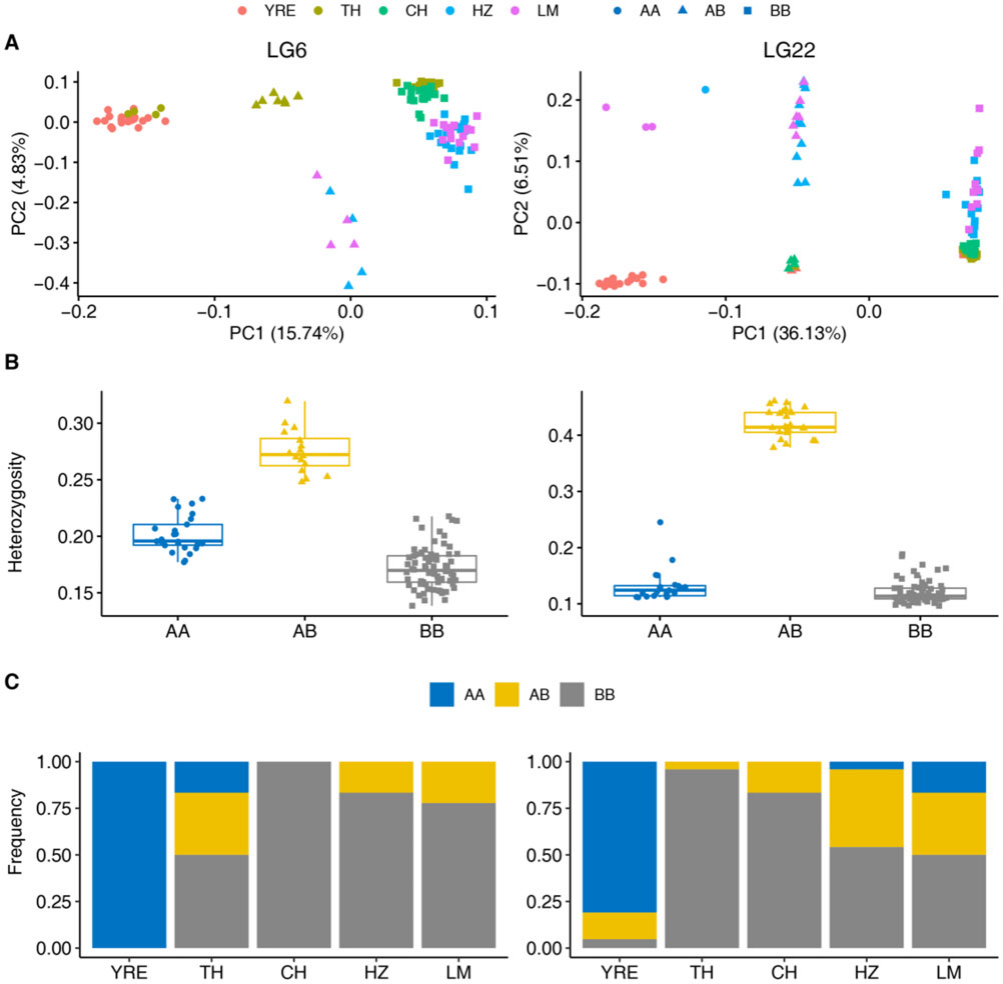
Characterizations of the two chromosome inversions on LG6 (left) and LG22 (right). (*A*) Scatter plots for PCA showing there are three distinct groups of individuals partitioned by PC1. Population origin of individuals is labeled by different colors. The three groups represented by different shapes correspond to three karyotypes: homokaryotypes for the reference arrangement (filled circle, AA), heterokaryotypes with both reference and alternative arrangements (filled triangle, AB), and homokaryotypes for the alternative inverted arrangement (filled square, BB). The group for homokaryotypes of the reference arrangement (AA) consists the majority of individuals from the anadromous population (YRE). The rearrangement karyotype for each individual is determined using PC1 of PCA based on the “k-means” algorithm. (*B*) Box plot for average heterozygosity of SNPs within the two chromosome inversions for individuals of the three groups identified by PCA. The heterokaryotypes (yellow filled triangle, AB) display the highest heterozygosity, whereas homokaryotypes (blue filled circle AA and gray filled square BB) show reduced heterozygosity. (*C*) Bar plot for karyotypic frequency of the two chromosome inversions in each population. The frequency of three karyotypes is represented by different colors: yellow for heterokaryotype AB, blue for homokaryotype AA, and gray for homokaryotype BB. YRE, Yangtze River Estuary; TH, Taihu Lake; CH, Chaohu Lake; HZ, Hongze Lake; LM, Luoma Lake.

Linkage disequilibrium network analysis (LDna) identified a total of five single-outlier clusters (SOCs) on different chromosomes, with median LD *r*^2^ values ranging from 0.33 to 1.00, number of loci ranging from 53 to 1,070, and sizes ranging from 1.61 to 31.54 Mb (table 1). Population genetic analysis of PCA and heterozygosity for the five SOCs suggested that these SOCs corresponded to five putative chromosome inversions which separated the individuals into three groups along PC1 (supplementary fig. S8, Supplementary Material online). The PCAs of all SNPs in the five chromosome inversions displayed three distinct clusters: the homokaryotypes of reference arrangement, the heterokaryotypes and the homokaryotypes of the alternative arrangement. All chromosome inversions were characterized with high LD, reduced heterozygosity in the homokaryotype, and strong divergence between the inverted and the uninverted rearrangement. Three of the five putative chromosome inversions on LG3, LG18, and LG19 broadly separated individuals of Hongze Lake and Luoma Lake from the other populations, suggesting their association with geographical population structure. The other two chromosome inversions on LG6 and LG22 broadly separated the freshwater-resident ecotype and the anadromous ecotype, suggesting their association with anadromous–freshwater adaptive divergence (fig. 3*A* and supplementary fig. S8*B* and *E*, Supplementary Material online). Most of the outlier SNPs located on LG6 and LG22 (96%) fell within the identified region of chromosome inversions.

**Table 1.** List of SOCs Identified by LDna.

Chromosome	Type	nLoci	nE	Lambda	Median.LD	Size (Mb)	Inferred cause
LG3	SOC	53	1,109	1.43	1.00	1.61	Inversion/geographic structure
LG6	SOC	94	1,615	4.23	0.78	31.54	Inversion/parallel adaptation
LG18	SOC	172	10,607	5.33	0.58	22.87	Inversion/geographic structure
LG19	SOC	65	141	0.65	0.33	31.39	Inversion/geographic structure
LG22	SOC	1,070	524,218	203.30	0.81	21.97	Inversion/parallel adaptation

Note.—nLoci, number of highly linked loci in this cluster; nE, number of edges; Lambda, λ value; Median.LD, median *r*^2^ between pairs of loci.

The identified putative inversion on LG6 covered about 31.54 Mb containing 1,077 genes, and the inversion on LG22 covered about 27.40 Mb with 759 genes (supplementary table S7, Supplementary Material online). Combined, the two inversion regions in LGs 6 and 22 covered more than 50 Mb (∼6% of the genome) and contained more than 1,800 genes (supplementary table S7, Supplementary Material online). However, the sizes of the inversions should be treated with caution, as their estimations were highly dependent on the accuracy of the reference genome assembly. The proportions of properly paired reads (defined as the forward read and the reverse read mapped on the same chromosome and with right orientation as well as proper insert size) aligned to the reference genome assembly were generally low for all individuals (∼80%, supplementary fig. S9, Supplementary Material online), which further indicated that some putative wrongly ordered contigs existed on linkage groups. Thus, the quality of the chromosome-level reference genome for *C. nasus* is not very high. Moreover, we found a large “gap” in the distribution of outliers on LG22 (fig. 2*A*), which was consistent with the identified positions of SNPs in the detected SOC. LD was also high between SNPs from either ends of the gap (median *r*^2^ = 0.80), implying that there might be assembly errors here. We thus excluded this gap from the inversion region on LG22, which resulted in a size of 21.97 Mb. All the following related results were based on the “gap” removed inversion on LG22.

The frequency of the putative chromosome rearrangements on LG6 and LG22 displayed large shifts between the anadromous and freshwater-resident populations. For inversion on LG6, all the individuals of the anadromous population (Yangtze River Estuary) were reference homokaryotype (AA), whereas most of the freshwater individuals were alternative homokaryotype (BB, mean karyotype frequency = 0.78, SD = 0.21) (fig. 3*C* and supplementary table S8, Supplementary Material online). In particular, all the individuals of Chaohu Lake were alternative homokaryotype BB. The heterokaryotypic individuals (AB) with both inverted and uninverted arrangements appeared in three freshwater populations, including Taihu Lake, Hongze Lake, and Luoma Lake. For inversion on LG22, 17 of the 21 individuals (81%) of the Yangtze River Estuary population were reference homokaryotype (AA), whereas most of the freshwater individuals were alternative homokaryotype (BB, mean karyotype frequency = 0.71, SD = 0.22). Interestingly, three individuals with heterokaryotype AB and one individual with alternative homokaryotype BB were found in the anadromous population (fig. 3*C*), which indicated that the inverted arrangement on LG22 presented as standing genetic variation in the anadromous population. The large frequency shifts of chromosome rearrangements of both chromosome inversions on LG6 and LG22 between the anadromous and freshwater-resident populations further explained the drastic allele frequency shifts for the detected outlier SNPs. The *F*_ST_ between the anadromous population and the four freshwater-resident populations calculated with all SNPs located in the chromosome inversion regions on LG6 and LG22 showed elevated differentiation with an average value of 0.37, which was an order of magnitude larger than the *F*_ST_ values based on neutral SNPs (supplementary table S9, Supplementary Material online).

### Gene Annotations of Outlier SNPs and GO Enrichment Analyses of Inversion Regions

Annotations for 859 of the 1,147 candidate outlier SNPs were retrieved. Of these, 380 SNPs were located in genic regions with 23 in coding sequence and 357 in introns, and 479 were within 10 kb of the closest gene. For the 23 SNPs in coding sequence of 16 genes, eight were nonsynonymous mutations in six genes (supplementary table S10, Supplementary Material online). Gene annotations of outlier SNPs indicated some genes that were possibly involved in adaptation to fresh water. These genes included genes encoding chloride intracellular channel protein, solute carrier family 25 member 47-A-like protein, and potassium channel subfamily K member 10A, solute carrier organic anion transporter family member, zinc transporter 2, potassium voltage-gated channel subfamily H member 5, sodium bicarbonate cotransporter gene, and so forth (supplementary table S10, Supplementary Material online).

**Fig. 4.**
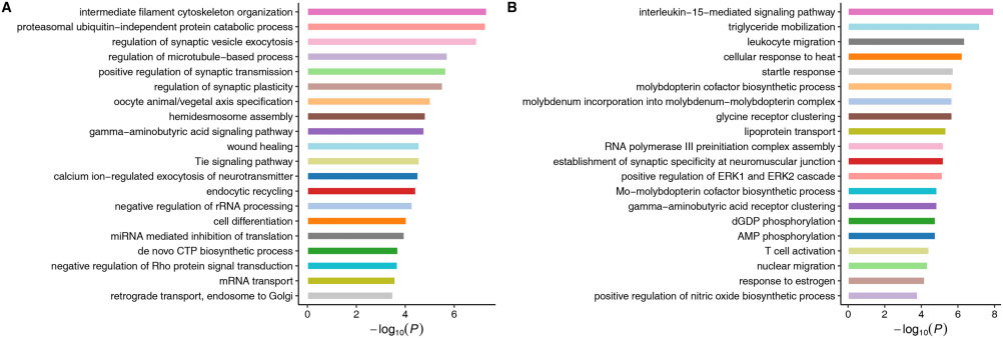
Bar plot for the GO enrichment of genes within two chromosomes inversions on LG6 (*A*) and LG22 (*B*). The top 20 significantly enriched GO terms of biological process are displayed.

Gene Ontology (GO) enrichment analyses of genes within the two chromosome inversions in terms of biological process identified a total of 72 and 73 significantly enriched GO terms for inversion regions on LG6 and LG22, respectively (*P *<* *0.01; fig. 4; supplementary tables S11 and S12, Supplementary Material online). No significantly enriched GO terms were overlapped between inversion regions of LG6 and LG22. These enriched terms were involved in diverse biological processes, for example, osmoregulation, immunoregulation, growth and maturation, locomotion, thermal response, metabolic process, and so forth (supplementary tables S11 and S12, Supplementary Material online).

## Discussion

Evolutionary change and adaptive divergence in natural populations can occur very rapidly, responding to strong selection over short ecological timescales (Carroll et al. 2007). However, the genetic architecture underlying these adaptive changes is still poorly understood. Here, we provide strong evidence of rapid parallel adaptation to fresh water and propose that chromosome inversions form a significant part of adaptive genetic variation. The apparently elevated differentiation observed in the two chromosome inversions indicates their potential role in maintaining and redistributing the adaptive substrate to fuel rapid divergence during freshwater adaptation. These results should provide exciting insights into the genetic mechanism underlying rapid adaptation of complex traits to changing environments in natural populations.

Chromosome inversions can play a key role in adaptive divergence because they protect inverted sequences from recombination in heterokaryotypes, allowing the coinheritance of multiple favorable alleles (Wellenreuther and Bernatchez 2018). Given that suppressed recombination allows mutational differences to accumulate between their variants, chromosomal inversions may create “genomic islands of divergence,” as observed in our study. The two inversions exhibited parallel association with freshwater adaptation, providing strong evidence that adaptation of *C. nasus* to fresh water in different lakes evolved similarly and independently. Most notably, drastic frequency shifts were observed for both chromosome inversions between the anadromous and resident forms, suggesting that they are strongly selected genomic regions in adaptation to freshwater environments. The results for enrichment analysis of genes within the two chromosome inversions in terms of biological process showed significant enrichment of genes involved in osmoregulation, immunoregulation, growth and maturation, locomotion, thermal response, metabolic process, and so forth. Changes in these biological processes probably underlie differences in morphology, physiology, and behavior between the anadromous and freshwater-resident forms (Yuan et al. 1976). Furthermore, the gene annotations associated with outlier SNPs also indicated that genes within the two inversions might play key roles in promoting adaptive differentiation between the two ecotypes. For example, some outlier SNPs were located within or linked to genes possibly involved in osmoregulation, including members of solute carrier family 25, potassium voltage-gated channel subfamily H, potassium channel subfamily K, and genes encoding chloride intracellular channel protein, sodium bicarbonate transporter-like protein, and so forth. In three-spined sticklebacks (*Gasterosteus aculeatus*), mutations at genes encoding solute carrier proteins have been associated with annual salinity variation in the Baltic Sea (Guo et al. 2015). The genes encoding solute carrier proteins also play key roles in promoting adaptive differentiation between the Gilbert Bay and offshore populations of the Atlantic cod (*Gadus morhua*), which experience different salinities (Sinclair-Waters et al. 2018). Furthermore, alternative functional exons of a potassium voltage-gated channel gene (*KCNH4*) are found on either side of one chromosomal inversion that has undergone parallel selection after freshwater invasion in three-spined sticklebacks, suggesting marine and freshwater specific isoforms (Jones et al. 2012). The chloride intracellular channel proteins is one of the major classes of ion channels predominantly localized to intracellular membranes, which is important for maintaining ionic homeostasis of intracellular organelles (Gururaja Rao et al. 2018). Given the different salinities experienced by the anadromous and freshwater-resident forms of *C. nasus*, these genes may play crucial roles in adaptive differentiation associated with osmoregulatory adaptation. Although the results of gene annotation are plausible, identifying the true target of selection is difficult within chromosomal rearrangements, where LD is strong (Hoffmann et al. 2004). As the two large chromosome inversions contain more than 1,800 genes, it is currently difficult to determine which genes and/or variants contribute to the fitness effects of the two inversions in freshwater adaptation of *C. nasus*.

Recent studies provide exciting insights into the role of SVs, in particular chromosome inversions, in adaptation and diversification in marine species. Chromosomal inversions have been proved playing an important role in repeated evolution of distinct marine and freshwater three-spined sticklebacks, and in the maintenance of divergent ecotypes during early stages of reproductive isolation (Jones et al. 2012). In the Atlantic cod, five large putative chromosome inversions have been associated with migratory behavior and geographical distribution, and are likely involved in the maintenance of genomic divergence on both sides of the Atlantic Ocean (Berg et al. 2017). Furthermore, the chromosomal rearrangement on LG1 that comprises two adjacent inversions is associated with parallel patterns of divergence between migratory and nonmigratory ecotypes of Atlantic cod on both sides of the Atlantic, providing further support for its role in local adaptation (Kirubakaran et al. 2016; Sinclair-Waters et al. 2018). Likewise, a large inverted region located on chromosome Omy5 has been linked to life-history strategies of anadromous and resident rainbow trout (*Oncorhynchus mykiss*) (Pearse et al. 2014). In the Atlantic herring (*Clupea harengus*), a 7.8-Mb inversion on chromosome 12 is found both in the East and West Atlantic, which possibly underlies ecological adaptation in relation to the water temperature during gonadal maturation before spawning or the water temperature at spawning/early larval development (Pettersson et al. 2019). Recently, a putative polymorphic chromosome inversion is detected within the Northwest Atlantic lineage of the capelin (*Mallotus villosus*), which may facilitate local adaptation to environmental conditions prevailing at spawning sites (Cayuela et al. 2020). In the marine snail (*Littorina saxatilis*), several candidate chromosomal inversions are associated with rapid parallel adaptation, which can store shared variation that fuels rapid parallel adaptation to heterogeneous environments (Morales et al. 2019). The importance of chromosome inversions in ecological and evolutionary processes of these marine species indicates that the analysis of inversions as well as other structural variants should be better integrated in studies pertaining to the molecular basis of adaptation and diversification (Wellenreuther and Bernatchez 2018).

Besides the nature of the genes and genomic regions under selection, the rate of genomic adaptation is also determined by the degree of environmental change, the availability of beneficial mutations, and the efficiency of positive selection. Populations adapt to novel environments in two distinct ways: selection on preexisting genetic variation and selection on new mutations (Barrett and Schluter 2008). Due to the immediately available beneficial alleles and their higher starting frequencies, adaptation is thought to be faster from standing variation than from new mutation (Innan and Kim 2004). The observation of selection at the same chromosome inversions on LG6 and LG22 and SNPs in independent replicate freshwater populations suggested that the selection resulted from standing genetic variation. Indeed, most FWA of the candidate SNPs and the alternative arrangement of the chromosome inversion on LG22 were found in the anadromous populations. In the anadromous populations, frequency of the FWA was significantly higher than MAF of the other SNPs, indicating that standing genetic variation with higher initial frequencies facilitated rapid adaptation to changing environment. The importance of standing genetic variation as source for adaptation has also been verified in genome-wide adaptation studies of a songbird (Lai et al. 2019) and three-spined sticklebacks (Jones et al. 2012). Recent simulation analyses demonstrate that when a population is adapting to a large optimum shift, a substantial allele frequency increase is expected for the selected alleles of loci with large effect size (Stetter et al. 2018). Transitions from marine to freshwater habitats constitute dramatic shifts between “adaptive zones” (Lee and Bell 1999), and this large optimum shift could facilitate strong selective strength on loci of large effect size (e.g., chromosome inversions acting as supergenes) in the rapid adaptation to fresh water. *N*_e_ is crucial in determining the effectiveness of selection relative to drift. Large populations experience less genetic drift than small ones, which increases the efficiency of selection and the power to detect selected alleles. Indeed, the frequencies of FWA and alternative arrangement of chromosome inversions on LG22 were higher in the two populations with larger *N*_e_ (Chaohu Lake and Taihu Lake) than in the two populations with smaller *N*_e_ (Hongze Lake and Luoma Lake), suggesting a stronger selective strength in populations with larger *N*_e_. All these conditions combined could lead to the observed strong selection in rapid adaptation of *C. nasus* to fresh water in different lake populations. However, the loci that showed signals of selection but not in all the four freshwater populations may reflect local adaptation.

## Concluding Remarks

Our results demonstrate strong evidence of repeated evolutionary change in response to similar selective environments in the freshwater adaptation of *C. nasus*, which contribute to the accumulating evidence that SVs form a significant part of adaptive genetic variation (Mérot et al. 2020). However, knowledge gaps for the two inversions in our study remain, including position of the inversion breakpoints and the identification of causal genes and/or mutations that facilitate inversion maintenance in the adaptation to fresh water of *C. nasus*. The two chromosome inversions can serve as a good starting point for characterizing the genetic basis of freshwater adaptations in *C. nasus*. Future work based on whole-genome resequencing and/or long-read data combined with functional validations and experimental work could allow us to clarify the nature and contribution of the putative chromosomal inversions in freshwater adaptation. In further studies, SVs should be studied in detail in a wider range of nonmodel organisms, which will provide a more comprehensive understanding of their ecological and evolutionary implications.

## Materials and Methods

### Sampling and RAD-Tag Sequencing

**Fig. 5.**
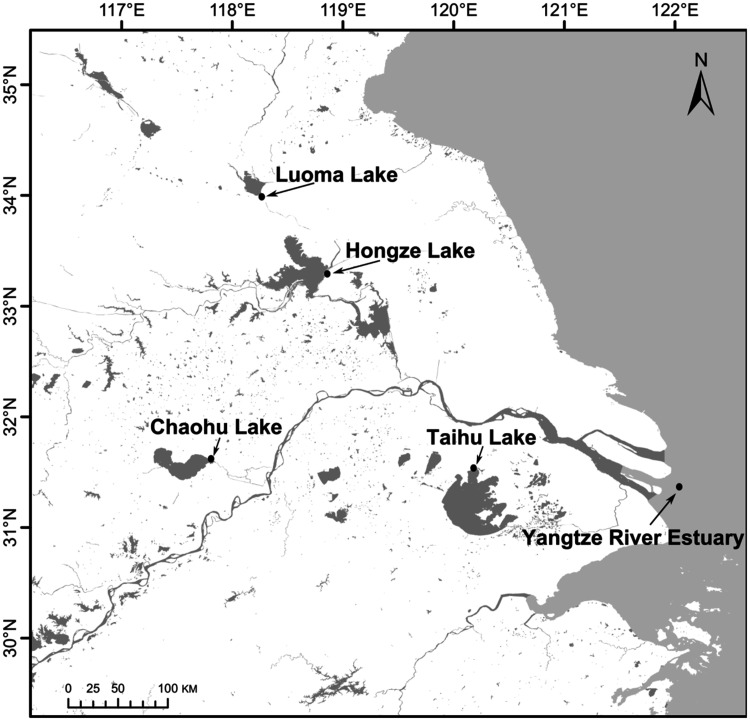
Map showing the sampling locations for the five populations analyzed, one anadromous population from the Yangtze River Estuary, four freshwater-resident populations from Luoma Lake, Hongze Lake, Chaohu Lake and Taihu Lake were collected.

A total of 111 specimens of *C. nasus* were collected from five populations during 2013–2016, which consisted of 21 individuals from Yangtze River Estuary, 18 individuals from Luoma Lake, 24 individuals from Hongze Lake, 24 individuals from Chaohu Lake, and 24 individuals from Taihu Lake (fig. 5 and supplementary table S13, Supplementary Material online). The Yangtze River Estuary samples were referred to as “ancestral anadromous population.” Samples from Luoma Lake, Hongze Lake, Chaohu Lake, and Taihu Lake were referred to as “derived freshwater-resident populations.” Muscles and fin clips were collected and immediately preserved in 95% ethanol. Genomic DNA was extracted by the standard phenol–chloroform extraction method. Samples were treated with RNase A to guarantee the DNA isolation without RNA. RAD libraries were prepared using the 6-bp cutter *Eco*RI following Etter and Johnson (2012) and were sequenced (11–13 individuals per sequencing lane) on the Illumina HiSeq 4000 platform at Allwegene Technology Inc. (Beijing, China) using paired-end 150-bp chemistry.

### RAD Data Filtering, SNP Genotyping, and Filtering

Raw sequence reads were quality-filtered as follows: 1) Read pairs with adaptors were removed using cutadapt v1.16 (Martin 2011), 2) low-quality read pairs were removed using “process_radtags” in Stacks v1.48 (Catchen et al. 2011) with a sliding window of 10% and quality score of 13, and 3) polymerase chain reaction-duplicated read pairs were removed using “clone_filter” in Stacks. The filtered reads were aligned to the chromosome-level assembly of *C. nasus* (NCBI assembly accession number: GCA_007927625.1) using BWA MEM v0.7.15-r1140 (Li 2013) with default parameters. Following the alignment, SNPs were called using a Bayesian approach as implemented in the package SAMtools v1.9 (Li et al. 2009). High-quality SNPs for downstream analyses were filtered using the following criteria: 1) present in at least 12 individuals for each population, 2) depth of coverage ≥ 6, 3) SNP overall quality score ≥ 30 and genotyping score ≥ 15, 4) observed heterozygosity for each population ≤ 0.5, 5) global MAF ≥ 0.05, and 6) retain the SNP with local MAF ≥ 0.2 in any of the five populations but failed to meet the criteria of the global MAF ≥ 0.05. SNPs VCF file was converted to other formats using PGDspider v2.1.1.5 (Lischer and Excoffier 2012) or in house Perl script. All the scripts used are available at GitHub.

For population genetic and demographic analyses that assume a set of neutral and unlinked markers, we created a reduced data set as following: First, any SNPs that were identified as outliers by either of the two detection methods (see Materials and Methods below) in any population pair were removed; second, we removed all SNPs on the two chromosome inversions (see Results section); third, only one SNP was kept in a 10-kb region to remove putative LD using VCFtools (Danecek et al. 2011). This neutral and LD-pruned data set was hereafter referred to as the neutral data set and, unless otherwise stated, was used as the primary data set for population genetic analyses (PCA, Admixture, NJ tree, and *F*_ST_) and demographic analysis.

### Summary Statistics and Population Genetic Structure

Genetic statistics, including average observed (*H*_O_) and expected (*H*_E_) heterozygosity of SNPs for each population were estimated using populations in Stacks. *F*_ST_ among all populations/between each pair of populations were calculated with Arlequin v3.5.2.2 and their significance was determined using 10,000 permutations (Excoffier and Lischer 2010). The significance was adjusted using FDR-BY method. Effective population size (*N*_e_) of each population was estimated using the LD method in NeEstimator v2.1 (Do et al. 2014).

Population structure was examined and visualized using three approaches. First, the model-based program Admixture v1.3.0 (Alexander et al. 2009) was used to determine population structure. *K* from 1 to 6 were applied with ten replicates for each *K*, the best *K* was determined using StructureSelector (Li and Liu 2018) using both cross-validation and Puechmaille methods (Puechmaille 2016). Second, PCA implemented in the R package “SeqVarTools” (Gogarten et al. 2020) was performed to visually explore patterns of allelic variation. Third, an NJ tree was constructed based on the pairwise Weir and Cockerham’s *F*_ST_ values using the R package “ape” (Paradis and Schliep 2019).

### Demographic Analysis

In order to test whether the four freshwater-resident populations were derived independently from the common anadromous population, we performed the demographic analysis using Approximate Bayesian Computation approach as implemented in DIYABC v2.1.0 (Cornuet et al. 2014). The Luoma Lake population was excluded from this analysis, as this population was obviously derived from Hongze Lake population. Thus, four populations were retained in the analysis, including one ancestral anadromous population from the Yangtze River Estuary and three derived freshwater-resident populations from Taihu Lake, Chaohu Lake, and Hongze Lake. For all simulations of the eight demographic scenarios (supplementary fig. S5, Supplementary Material online), 2,000 SNPs randomly chosen from the neutral unlinked data set were used. Three replicates were applied for simulations of each demographic scenario. For simplification, each simulation was performed with 1e6 runs, resulting in a total of 3 × 8 × 1e6 runs. Models comparison was performed using the logistic approach in DIYABC (see supplementary fig. S5, Supplementary Material online, for further parameter details of each model).

### Identification of Outlier SNPs

Loci that displayed elevated divergence between the anadromous and resident forms were identified by two methods. First, we performed the FET for the difference in allele frequency on the whole data set, a *P*-value <1e-4 (i.e., −log_10_(*P*) > 4) was used as the threshold after examining the Manhattan plot (fig. 2*A*), which corresponding to a mean *FDR* level of 0.005 (max = 0.007). Second, we used the R package pcadapt (Privé et al. 2020) to detect SNPs involved in biological adaptation. Pcadapt assumes that candidate markers are outliers with respect to how they are related to population structure based on PCA, which can handle admixed individuals and does not require grouping individuals into populations. We set *K* = 2 to reflect population structure, and any SNP with a *q*-value ≤0.1 was considered as an outlier. To reduce the possibility of false positives, only the SNPs that were identified as outliers using both methods and shared by all four population pairs were considered as candidate outliers. We defined the allele of which the frequency increased in all the derived freshwater populations as the FWA for candidate outlier SNPs. We then calculated the frequency of FWA in the ancestral anadromous and the derived freshwater-resident populations for the candidate outlier SNPs, as well as their shifts in the four population pairs.

### Putative Inversion Region Identification

As clusters of loci with unusually high LD might be generated by chromosomal rearrangements, such as inversions, we detected clusters of SNPs in high LD (outlier clusters, OCs) using the LDna as implemented in the R package “LDna” (Kemppainen et al. 2015). All populations were pooled prior to calculating LD, thereby creating sample admixture LD. The LD (*r*^2^) values between pairs of SNPs were calculated individually on each chromosome by VCFtools using the whole data set with MAF of 0.1. The *r*^2^ matrix was then used for LDna. There are two key parameters that can be set in LDna. The minimum number of edges |*E*|_min_, which corresponds to the minimum number of connections among the vertices of a cluster. This parameter controls the minimum number of SNPs within an OC. After several preliminary runs, |*E*|_min_ = 30 was applied to represent a compromise between detecting clusters large enough to represent chromosomal rearrangements and avoiding noise that result from physical linkage within chromosomes. Another key parameter φ controls the minimum LD threshold above which the median pairwise LD within a cluster is higher than the intercluster LD for the group of SNPs to be considered as an OC. This parameter was selected by initializing its value = 2 then increased the value of φ by one in each iteration until no more LD clusters were obtained for at least three times within one chromosome. Only SOCs with a minimum of 30 SNPs were retained, and clusters with a low median LD (*r*^2^ < 0.3) were also discarded. The sizes of each SOC were defined as the most extreme positions of the SNPs included in each SOC (except for LG22, see Results section for details). The resulting SOCs were then examined in the downstream analyses.

SOCs can arise due to various reasons, such as chromosome inversions, spatial population structure, or local adaptation. Inversions can be identified by detecting groups of genetically distinct individuals that correspond to different karyotypes. The suppressed recombination between arrangements due to inversions will result in the presence of three distinct groups of individuals, which corresponded to three karyotypes (AA: homokaryotypes for the reference arrangement, AB: heterokaryotypes with both reference and alternative arrangements, and BB: homokaryotypes for the alternative inverted arrangement). The second group with karyotype AB should have the highest heterozygosity relative to both the homokaryotypes that should have reduced heterozygosity. Thus, three groups will be separated along the axis of PC1 of PCA based on all SNPs within an LD cluster for inversions. Chromosome inversions were then confirmed by PCA and heterozygosity analyses for each SOC identified by LDna. The rearrangement karyotype of each individual was determined using the “k-means” algorithm as implemented in R (R Core Team 2020). Based on individual karyotype assignments, we calculated the frequency of the reference and alternative arrangement of inversions (allele “A” and “B”) and the frequency of the three karyotypes (AA, AB, and BB) for each population.

### Genome Annotation

Genes were predicted using the comprehensive evidence from both ab initio gene predictors and protein and transcript alignments. Gene predictions were mainly accomplished using Funannotate v1.8.0 (https://github.com/nextgenusfs/funannotate, last accessed November 11, 2020) and BRAKER v2.1.5 (Brůna, Hoff, et al. 2020) by incorporating the gene prediction tools GeneMark v4.58 (Brůna, Lomsadze, et al. 2020), AUGUSTUS v3.3.3 (Stanke et al. 2008), PASA v2.3.3 (Haas et al. 2003), and EVidenceModeler v1.1.1 (Haas et al. 2008). Firstly, a de novo repeat library was constructed using RepeatModeler v1.0.11, and repeats were then identified using RepeatMasker open-4.0.9. This information was further incorporated into the original repeat masked genome from NCBI. Secondly, BRAKER was used to train the gene prediction tools GeneMark-ETP and AUGUSTUS, and generate the ab initio predictions based on four RNA-seq data (NCBI SRA accession number: SRR5137767, SRR5137781, SRR5137792, SRR5138014) and protein homology information from two closely related species *Clupea harengus* (NCBI assembly accession number: GCA_000966335.1) and *Denticeps clupeoides* (NCBI assembly accession number: GCA_900700375.1). Thirdly, RNA-seq reads were aligned to the genome using HISAT2 v2.1.0 (Kim et al. 2019), and genome-guided RNA-seq assembly was performed using Trinity v2.8.5 (Grabherr et al. 2011), and the results were passed to PASA to generate high-quality gene structures. Fourthly, evidence from protein sequences of Swiss-Prot and RNA-seq reads was extracted from the alignments to the genome by using DIAMOND v0.9.21 (Buchfink et al. 2015) and Minimap2 v2.17-r941 (Li 2018). Finally, the above ab initio gene predictions, protein and transcript alignments were combined into weighted consensus gene structures using EVidenceModeler v1.1.1. The final gene predictions consisted of 28,668 gene models with average gene length of 9,261 bp.

Protein sequences were extracted and annotated using Funannotate from alignments on multiple databases including the Swiss-Prot/TrEMBL, Pfam-A, EggNOG, MEROPS, CAZYme, BUSCO, and InterProScan. SNPs consequences (e.g., missense, synonymous, etc.) for the outliers were determined by Ensembl Variant Effect Predictor (McLaren et al. 2016) with distance up and/or downstream between a variant and a gene setting to 10 kb.

### Functional Enrichment of Chromosome Inversions

Gene models and annotations for genes within the two putative chromosome inversion regions were extracted using BEDtools (Quinlan and Hall 2010). In order to identify the significantly enriched GO terms, we firstly aligned the protein sequences of *C. nasus* to the protein database of *Danio rerio*, then we retrieved the related GO terms from the Gene Ontology Annotation (GOA) database (ftp://ftp.ebi.ac.uk/pub/databases/GO/goa/, last accessed November 11, 2020). Enrichment analysis for GO terms in each candidate inversion region was performed by the R package “topGO” (Alexa and Rahnenfuhrer 2020) using the “weight01” algorithms.

## Supplementary Material

Supplementary data are available at *Molecular Biology and Evolution* online.

## Supplementary Material

msaa290_Supplementary_Data
