# Supplementary material for: Genomic Architecture of Rapid Parallel Adaptation to Fresh Water in a Wild Fish
Source: Mol Biol Evol. 2020 Nov 4;38(4):1317–29. doi: 10.1093/molbev/msaa290 (PMC8480189; doi:10.1093/molbev/msaa290)
Supplement: msaa290_Supplementary_Data [file msaa290_Supplementary_Data.pdf]

Supplementary Materials for

Genomic architecture of rapid parallel adaptation to fresh water in a wild fish

Shao-Bing Zong[1,4,#], Yu-Long Li[1,2,3,#], Jin-Xian Liu[1,2,3,*]

[1] CAS Key Laboratory of Marine Ecology and Environmental Sciences, Institute of Oceanology, Chinese Academy of Sciences, Qingdao 266071, China.

[2] Laboratory for Marine Ecology and Environmental Science, Qingdao National Laboratory for Marine Science and Technology, Qingdao 266237, China.

[3] Center for Ocean Mega-Science, Chinese Academy of Sciences, Qingdao 266071, China.

[4] University of Chinese Academy of Sciences, Beijing 100049, China.

[#] These authors contributed equally to this work

[*] **Corresponding author**: jinxianliu@gmail.com

This PDF file includes:

Figures S1 to S9

Tables S1 to S13

Table of contents

Table of contents 2

Fig. S1 Distribution of breadth of coverage of RAD sequencing for individuals in each population. 3

Fig. S2 Density plot of SNPs identified on the 24 chromosomes of the *Coilia nasus* genome. 4

Fig. S3 Distribution of SNP distance from their nearest neighbors for each chromosome. 5

Fig. S4 CLUMPAK major mode plots for the Admixture results (K = 1 to 4 after removing spurious clusters) based on neutral SNPs. 6

Fig. S5 Eight scenarios simulated in DIYABC to assess the population demographic history of *Coilia nasus*. 8

Fig. S6 Venn diagram representing the overlap of outlier SNPs detected by both Fisher's exact test (FET) and pcadapt among four anadromous-freshwater population pairs. 10

Fig. S7 Box plot for the frequency of fresh water favored allele (FWA) of putative candidate SNPs and the minor allele frequency (MAF) of neutral SNPs in the ancestral anadromous Yangtze River Estuary population. 11

Fig. S8 Population genetic analyses of LD clusters for each set of SOC loci of *Coilia nasus* 12

Fig. S9 Box plot showing the distribution of proper mapping rate of paired reads of RAD sequencing to the reference genome of *Coilia nasus* for individuals of each population. 13

Table S1 Summary of number of retained SNPs for each filtering step. 14

Table S2 Summary of genetic diversity statistics for five populations of *Coilia nasus*. 15

Table S3 Population pairwise F_{ST} obtained by using all loci (below the diagonal) and using neutral loci (above the diagonal). 16

Table S4 Summary of posterior probabilities of eight demographic history scenarios evaluated in DIYABC analysis using neutral SNPs data. 17

Table S5 Posterior distributions of population demographic parameters from the scenario with the highest posterior probability (scenario 4) inferred by DIYABC analysis using neutral SNPs data. 18

Table S6 Numbers of outlier SNPs detected by Fisher's exact test and pcadapt for four anadromous-freshwater population pairs. 19

Table S7 Annotations of genes in two inversion regions related to parallel adaptation (LG6 and LG22). 20

Table S8 Frequency of three karyotypes and two rearrangement variants of the two chromosome inversions on LG6 and LG22 in the anadromous population (Yangtze River Estuary) and other four freshwater resident populations. 61

Table S9 Population pairwise F_{ST} obtained by using all SNPs located in the chromosome inversion regions (LG6 and LG22). 62

Table S10 Gene annotations of the candidate outlier SNPs. 63

Table S11 Gene ontology (GO) enrichment of biological process for genes in chromosome inversion region on LG6. ... 122

Table S12 Gene ontology (GO) enrichment of biological process for genes in chromosome inversion region on LG22. . 125

Table S13 Site locations, date of collection and sample size for all samples included in this study. 128

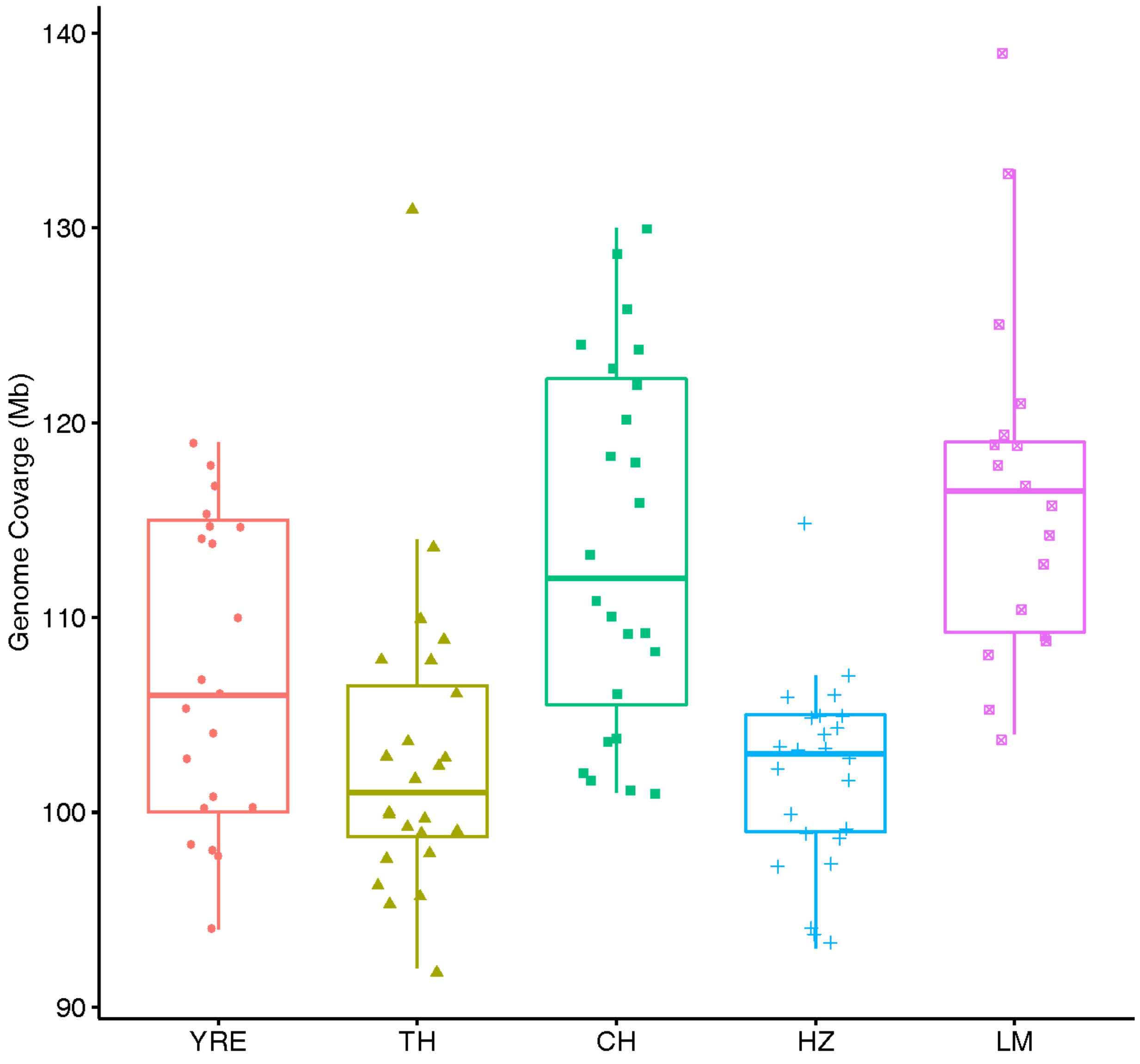

Fig. S1 Distribution of breadth of coverage of RAD sequencing for individuals in each population.

YRE: Yangtze River Estuary, TH: Taihu Lake, CH: Chaohu Lake, HZ: Hongze Lake, LM: Luoma Lake.

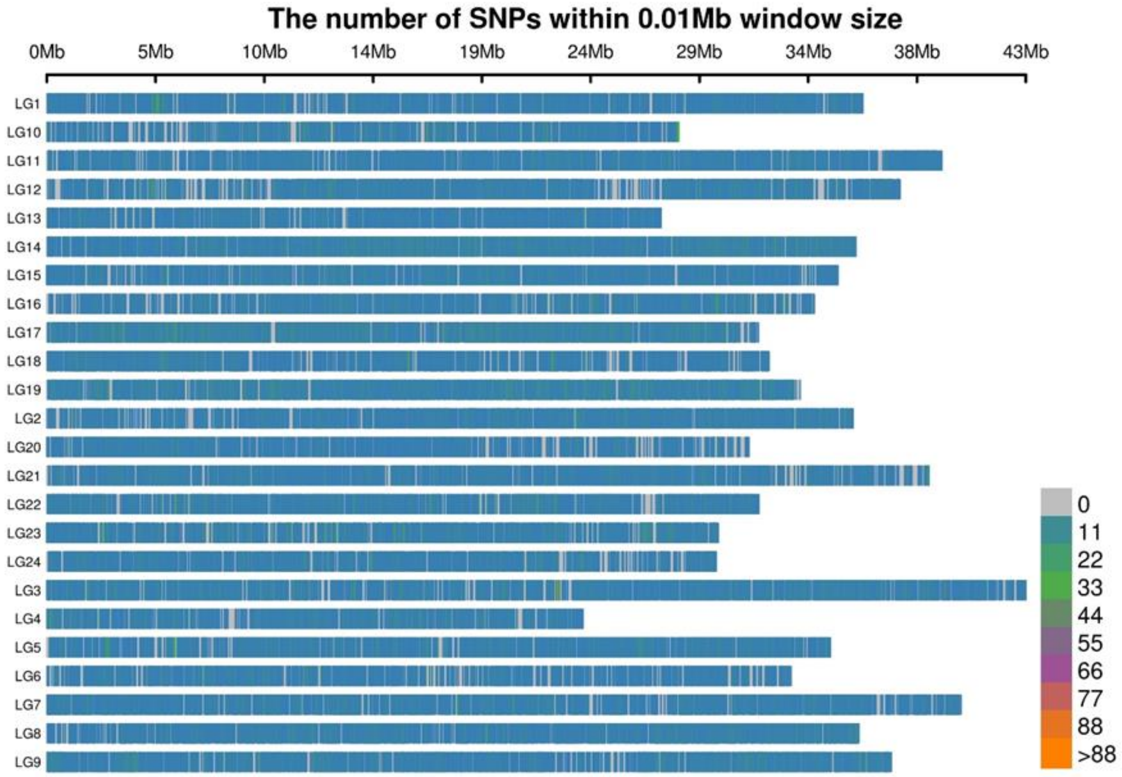

Fig. S2 Density plot of SNPs identified on the 24 chromosomes of the *Coilia nasus* genome.

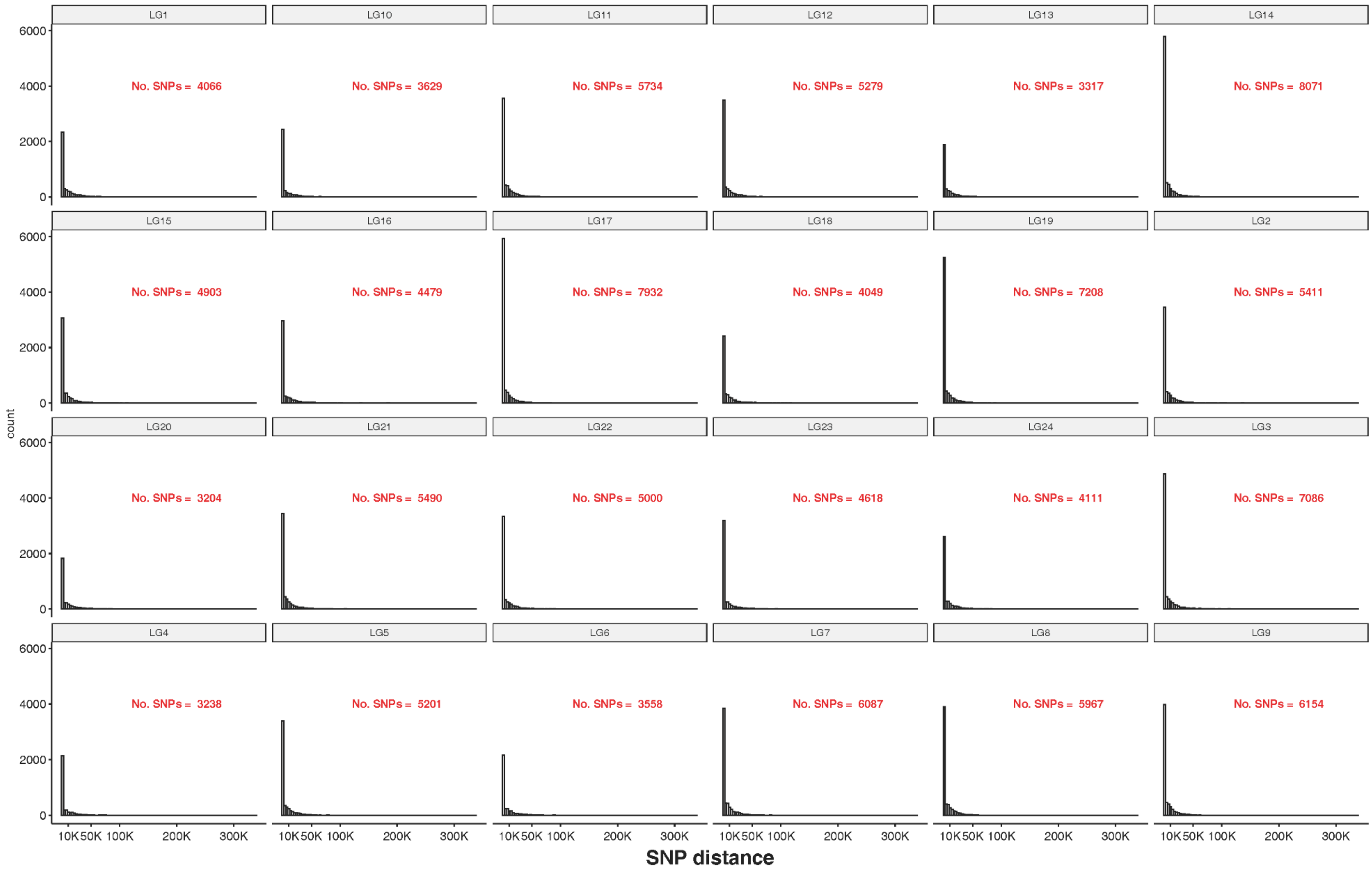

Fig. S3 Distribution of SNP distance from their nearest neighbors for each chromosome.

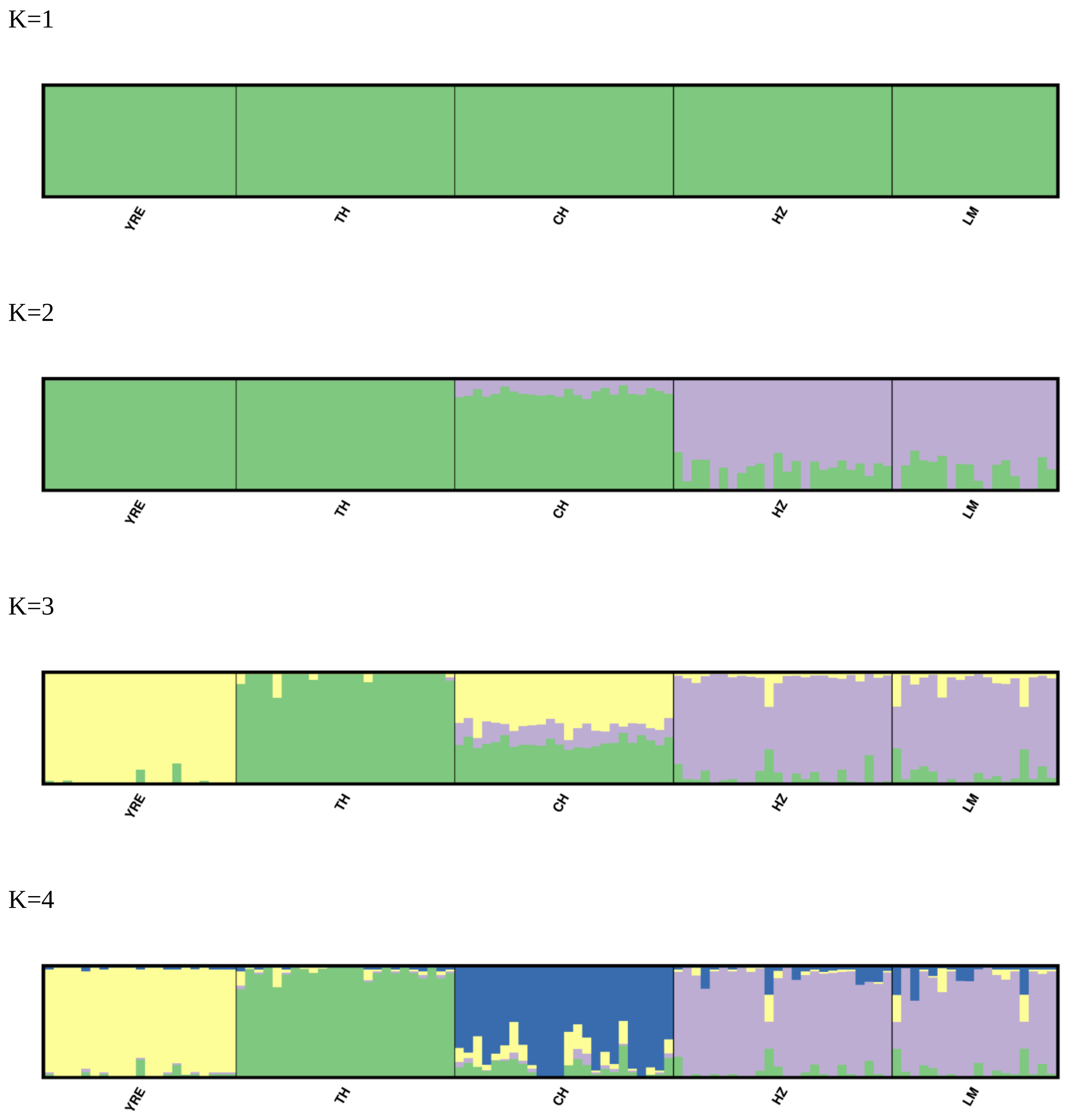

Fig. S4 CLUMPAK major mode plots for the Admixture results (K = 1 to 4 after removing spurious clusters) based on neutral SNPs.

YRE: Yangtze River Estuary, TH: Taihu Lake, CH: Chaohu Lake, HZ: Hongze Lake, LM: Luoma Lake.

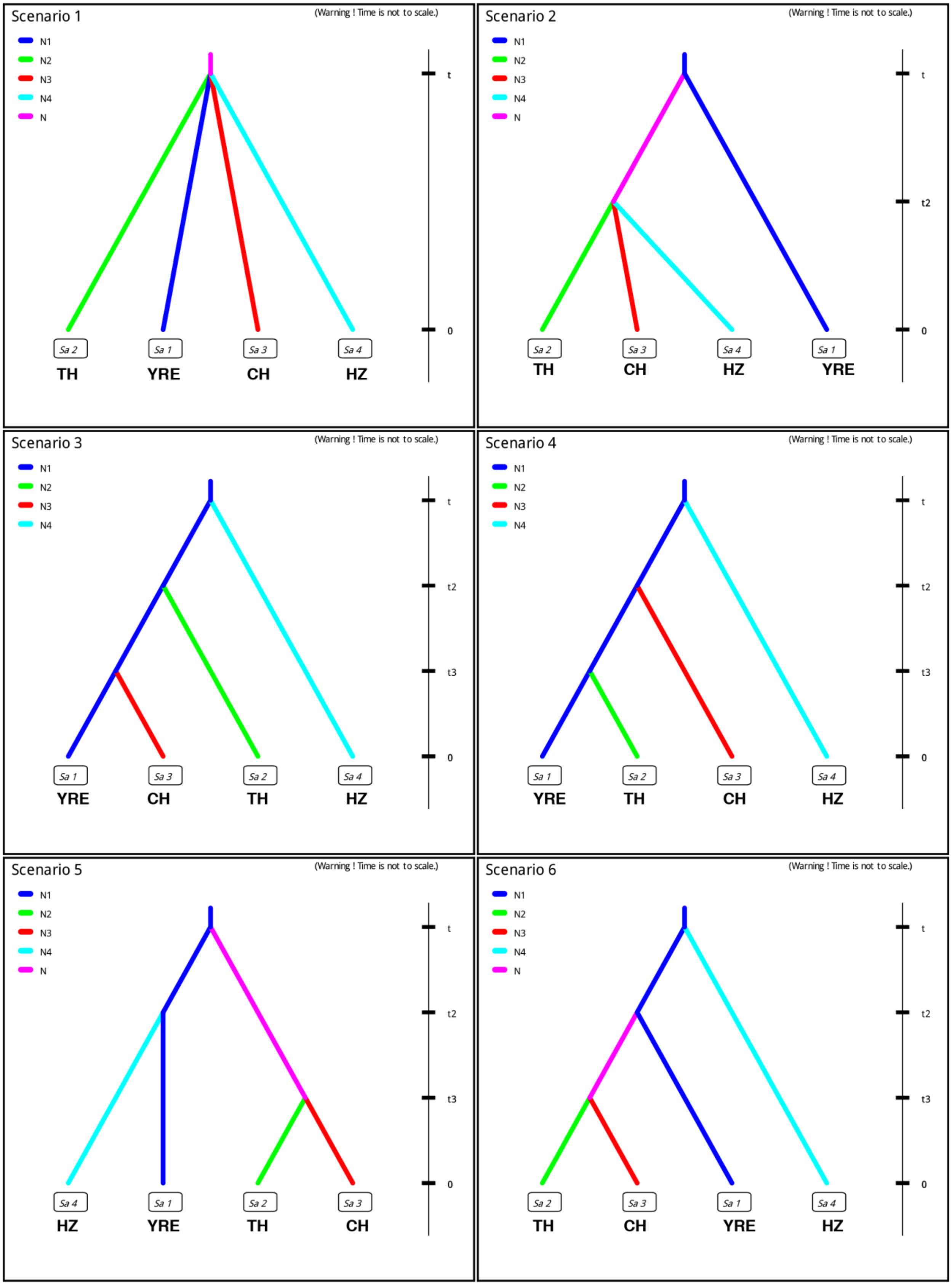
Scenario 1
(Warning ! Time is not to scale.)
N1
N2
N3
N4
N
t
0
Sa 2
Sa 1
Sa 3
Sa 4
TH
YRE
CH
HZ
Scenario 2
(Warning ! Time is not to scale.)
N1
N2
N3
N4
N
t
t2
0
Sa 2
Sa 3
Sa 4
Sa 1
TH
CH
HZ
YRE
Scenario 3
(Warning ! Time is not to scale.)
N1
N2
N3
N4
t
t2
t3
0
Sa 1
Sa 3
Sa 2
Sa 4
YRE
CH
TH
HZ
Scenario 4
(Warning ! Time is not to scale.)
N1
N2
N3
N4
t
t2
t3
0
Sa 1
Sa 2
Sa 3
Sa 4
YRE
TH
CH
HZ
Scenario 5
(Warning ! Time is not to scale.)
N1
N2
N3
N4
N
t
t2
t3
0
Sa 4
Sa 1
Sa 2
Sa 3
HZ
YRE
TH
CH
Scenario 6
(Warning ! Time is not to scale.)
N1
N2
N3
N4
N
t
t2
t3
0
Sa 2
Sa 3
Sa 1
Sa 4
TH
CH
YRE
HZ

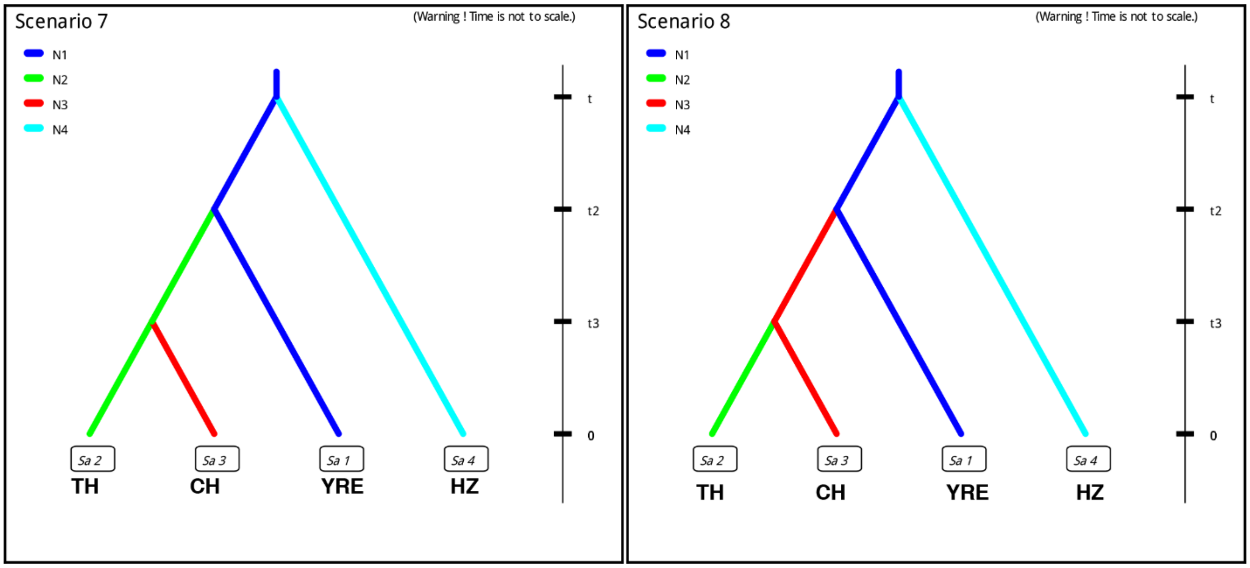

Fig. S5 Eight scenarios simulated in DIYABC to assess the population demographic history of *Coilia nasus*.

YRE: Yangtze River Estuary, TH: Taihu Lake, CH: Chaohu Lake, HZ: Hongze Lake, LM: Luoma Lake.

In the eight scenarios, t# represents the time-scale in terms of the number of generations, and N represents the effective population size (N_e) of an unknown ancestral population. N1: N_e of Yangtze River Estuary; N2: N_e of Thaihu Lake; N3: N_e of Chaohu Lake; N4: N_e of Hongze Lake.

Scenario 1: Four populations of size N1, N2, N3 and N4 diverged at time t from an ancestral population of size N.

Scenario 2: The common ancestral population of the three freshwater resident populations diverged from the Yangtze River Estuary population at time t. Then the freshwater resident populations were simultaneously derived from the common ancestral population at time t2.

Scenario 3: The three freshwater resident populations were derived independently from their common ancestral anadromous population. The Hongze Lake population was derived from the Yangtze River Estuary population at time t, then the Taihu Lake population and Chaohu Lake population formed at time t2 and t3, respectively.

Scenario 4: The three freshwater resident populations were derived independently from their common ancestral anadromous population. The Hongze Lake population was derived from the Yangtze River Estuary population at time t, then the Chaohu Lake population and Taihu Lake population formed at time t2 and t3, respectively.

Scenario 5: The common ancestral population of the Taihu Lake population and the Chaohu Lake population was derived from the Yangtze River Estuary population at time t. At time t2, the Hongze Lake population was

derived from the Yangtze River Estuary population. Then, the Chaohu Lake population and Taihu Lake population were derived from their common ancestral population at time t3.

Scenario 6: The Hongze Lake population was derived from the Yangtze River Estuary population at time t. At time t2, the common ancestral population of the Taihu Lake population and the Chaohu Lake population was derived from the Yangtze River Estuary population. Then, the Chaohu Lake population and Taihu Lake population were derived from their common ancestral population at time t3.

Scenario 7: The Hongze Lake population was derived from the Yangtze River Estuary population at time t. The Taihu Lake population was derived from the Yangtze River Estuary population at time t2, and the Chaohu Lake population was derived from the Taihu Lake at time t3.

Scenario 8: The Hongze Lake population was derived from the Yangtze River Estuary population at time t. The Chaohu Lake populations was derived from the Yangtze River Estuary population at time t2, and the Taihu Lake population was derived from the Chaohu Lake at time t3.

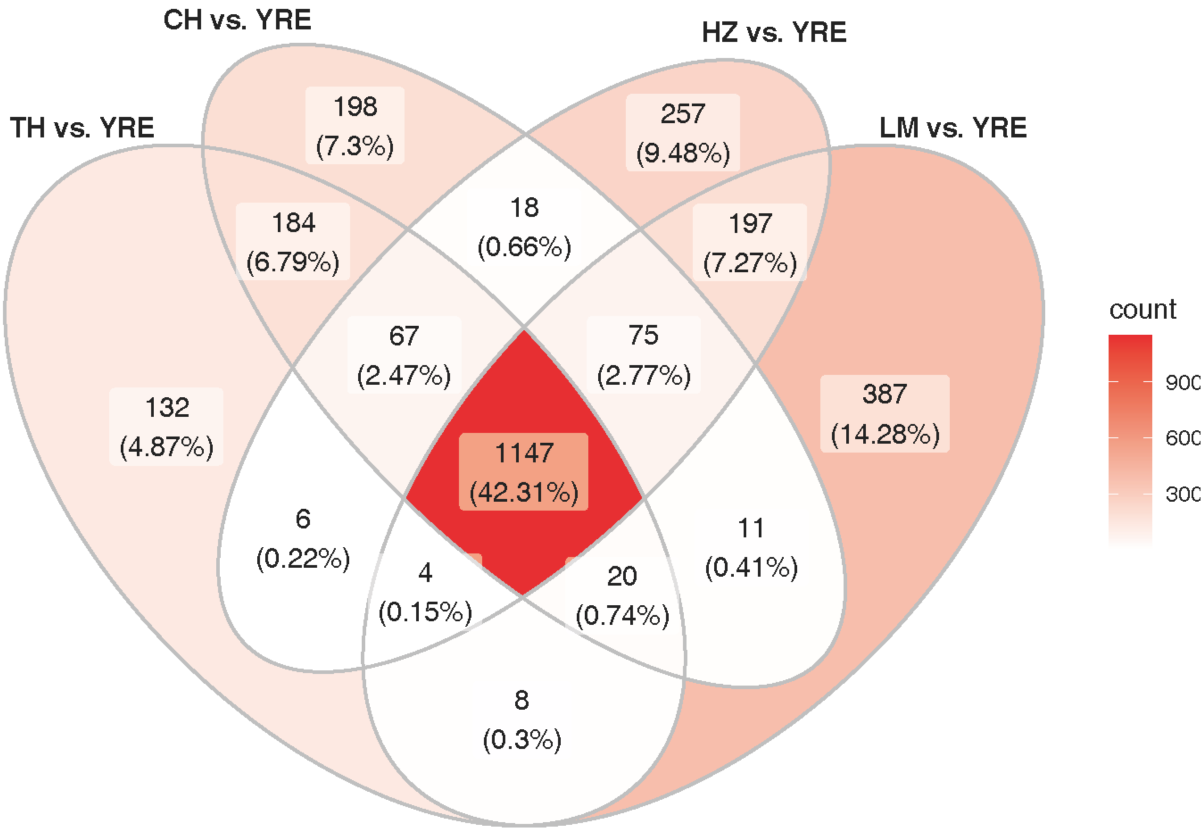

Fig. S6 Venn diagram representing the overlap of outlier SNPs detected by both Fisher's exact test (FET) and pcadapt among four anadromous-freshwater population pairs.

Note: Only outliers detected by both methods were used. YRE: Yangtze River Estuary, TH: Taihu Lake, CH: Chaohu Lake, HZ: Hongze Lake, LM: Luoma Lake.

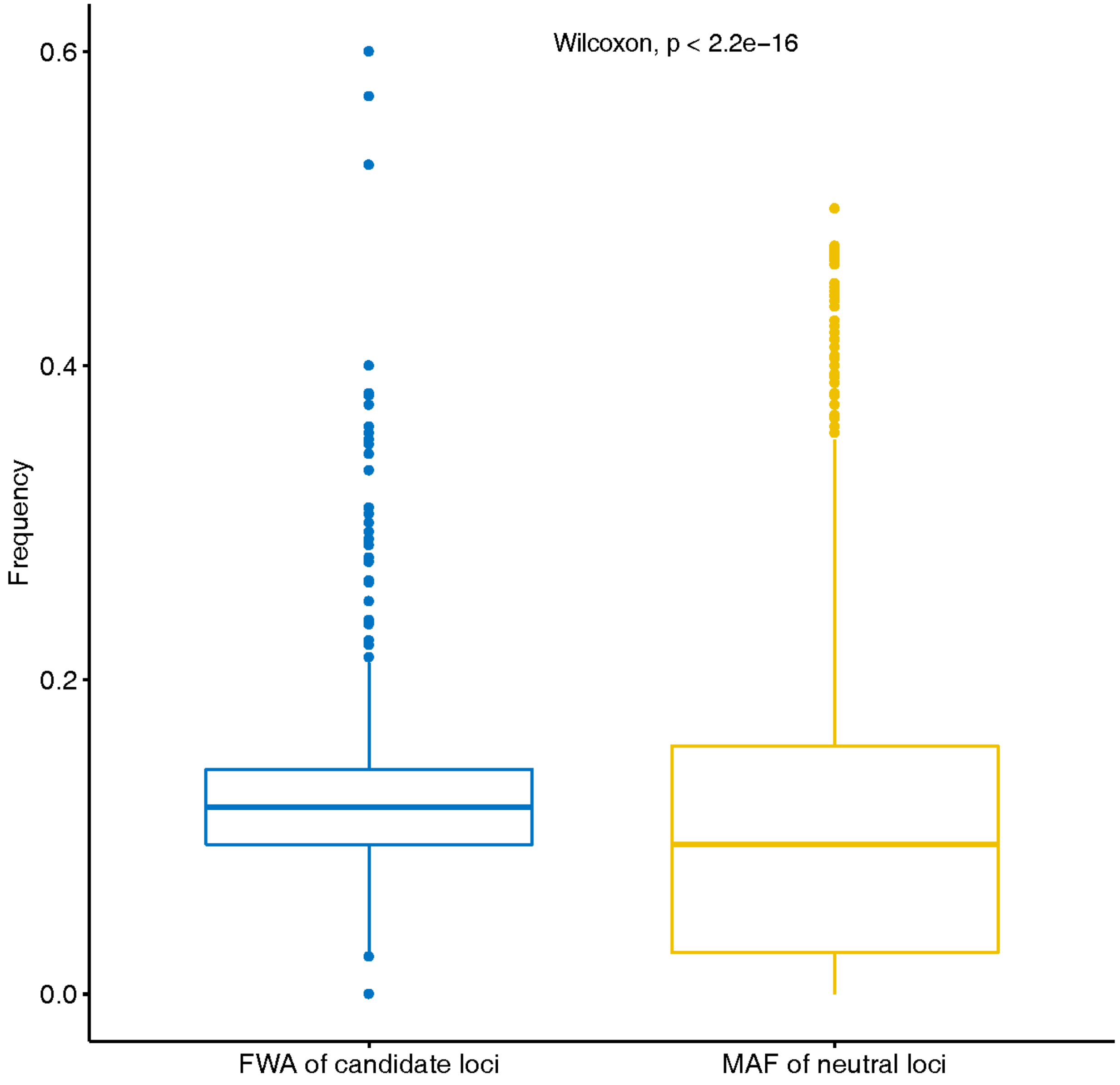

Fig. S7 Box plot for the frequency of fresh water favored allele (FWA) of putative candidate SNPs and the minor allele frequency (MAF) of neutral SNPs in the ancestral anadromous Yangtze River Estuary population.

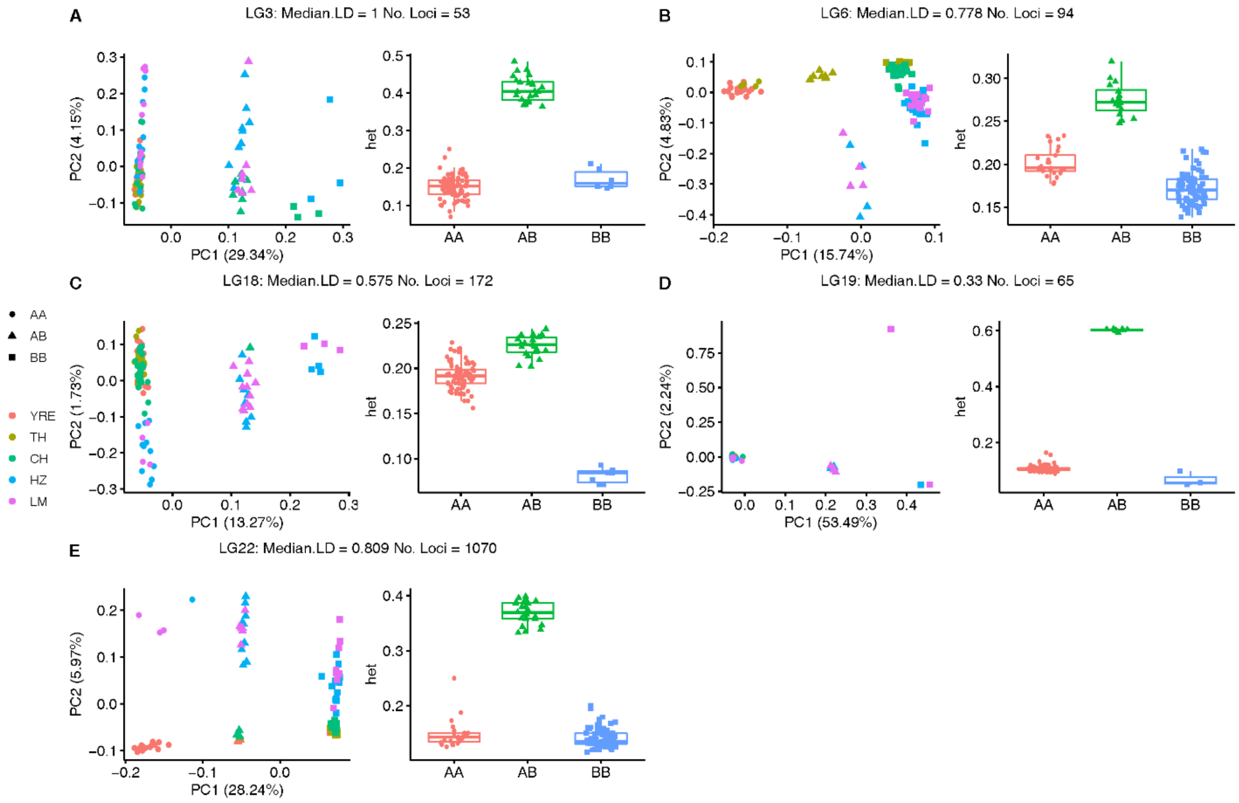

Fig. S8 Population genetic analyses of LD clusters for each set of SOC loci of *Coilia nasus*.

YRE: Yangtze River Estuary, TH: Taihu Lake, CH: Chaohu Lake, HZ: Hongze Lake, LM: Luoma Lake.
For each SOC (A to E), individuals were partitioned into three genetically distinct groups based on axis on PC1 by k-means algorithm. Populations were labeled by different colors; groups were labeled by different shapes. The separation of the three groups is visualized along the first two PCA axes, with percentage of variation explained indicated on the axes. The distribution of heterozygosity values for loci from each group was also shown.

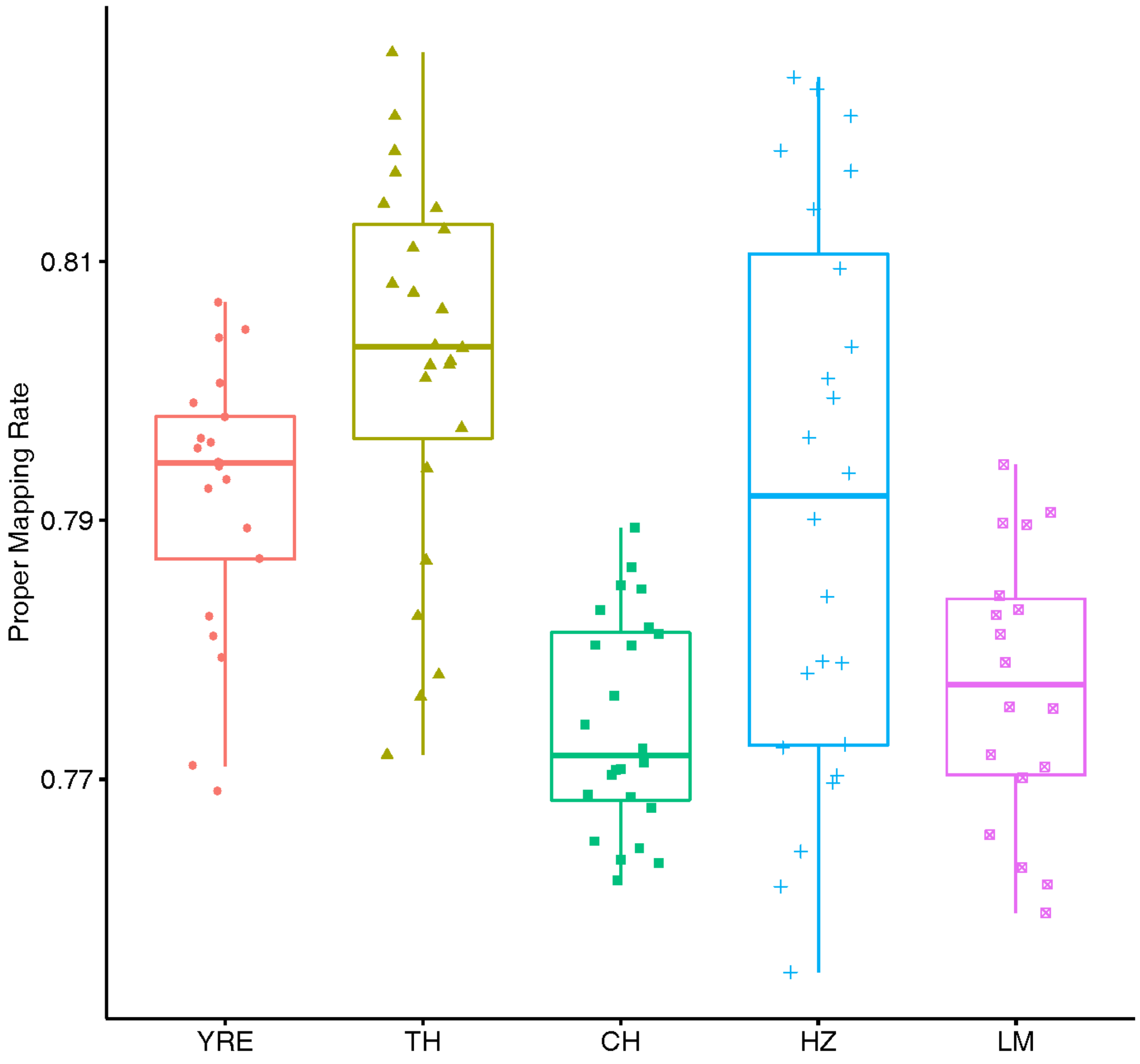

Fig. S9 Box plot showing the distribution of proper mapping rate of paired reads of RAD sequencing to the reference genome of *Coilia nasus* for individuals of each population.

Dots represent individuals in each population. YRE: Yangtze River Estuary, TH: Taihu Lake, CH: Chaohu Lake, HZ: Hongze Lake, LM: Luoma Lake.

Table S1 Summary of number of retained SNPs for each filtering step.

	Filtering steps	No. SNPs
0	Initial datasets	6,542,393
1	Bi-allelic	3,360,843
2	Q ≥ 30	2,824,267
3	GQ ≥ 15 and present in at least 12 individuals	1,656,923
4	Total coverage ≥ 0.9	1,247,353
5	Depth of coverage ≥ 6	542,446
6	MAF ≥ 0.05 or local MAF ≥ 0.2	198,814
7	$H_O \leq 0.5$	123,792
7	Final datasets	123,792

Q: SNP overall quality, GQ: SNP genotype quality, MAF: minor allele frequency, H_O: observed heterozygosity.

Table S2 Summary of genetic diversity statistics for five populations of *Coilia nasus*.

Population	No. polymorphic SNPs	H_O	H_E	N_e (95%CI)
Yangtze River Estuary	91,269	0.22±0.12	0.22±0.12	12752.8 (8735.3-23613.9)
Luoma Lake	117,017	0.23±0.12	0.24±0.12	160.4 (159.5-161.3)
Hongze Lake	119,693	0.23±0.12	0.23±0.12	170.7 (170-171.5)
Chaohu Lake	104,176	0.21±0.12	0.20±0.12	3492.2 (3195-3850.3)
Taihu Lake	92,316	0.22±0.12	0.22±0.12	3494.3 (3162.5-3903.7)

Summary statistics including the means of observed (H_O) and expected (H_E) heterozygosity, effective population size (N_e) estimated based on LD method.

Table S3 Population pairwise F_{ST} obtained by using all loci (below the diagonal) and using neutral loci (above the diagonal).

	Yangtze River Estuary	Chaohu Lake	Hongze Lake	Luoma Lake	Taihu Lake
Yangtze River Estuary	0	**0.01**	**0.04**	**0.04**	**0.01**
Chaohu Lake	**0.05**	0	**0.03**	**0.03**	**0.01**
Hongze Lake	**0.09**	**0.05**	0	0	**0.05**
Luoma Lake	**0.09**	**0.06**	0	0	**0.05**
Taihu Lake	**0.05**	**0.01**	**0.07**	**0.08**	0

Significant values after FDR-BY correction ($P < 0.01707$) are highlighted in bold, negative values are converted to zero.

Table S4 Summary of posterior probabilities of eight demographic history scenarios evaluated in DIYABC analysis using neutral SNPs data.

n	scenario 1	scenario 2	scenario 3	scenario 4	scenario 5	scenario 6	scenario 7	scenario 8
80000	0.0034<br>[0.0000,1.0000]	0<br>[0.0000,1.0000]	0<br>[0.0000,1.0000]	0.9966<br>[0.9908,1.0000]	0<br>[0.0000,1.0000]	0<br>[0.0000,1.0000]	0<br>[0.0000,1.0000]	0<br>[0.0000,1.0000]

n: number of data used; values in each bracket represent the 95% confidence intervals for each model. Posterior probabilities for all eight scenarios are estimated with 1% of simulated data sets closest to the observed. Scenario 4 is the best supported scenario because of the highest probabilities.

Table S5 Posterior distributions of population demographic parameters from the scenario with the highest posterior probability (scenario 4) inferred by DIYABC analysis using neutral SNPs data.

Parameter	mean	median	Mode	q025	q050	q250	q750	q950	q975
N1	9,920	9,940	9,960	9,710	9,780	9,900	9,970	9,990	10,000
N2	1,290	879	600	201	268	562	1,390	4,010	5,740
N3	8,000	8,210	8,510	4,800	5,560	7,400	8,850	9,690	9,840
N4	5,090	5,170	5,290	3,180	3,570	4,600	5,660	6,340	6,600
t3	60.5	59.3	55.4	32.3	37.3	50.5	69.2	88.4	95.8
t2	83.2	83.0	80.7	47.5	53.4	71.1	94.7	113	120
t	365	293	251	105	130	222	376	693	1,100

N1: effective population size (N_e) of Yangtze River Estuary; N2: N_e of Taihu Lake; N3: N_e of Chaohu Lake; N4: N_e of Hongze Lake; t: time of divergence between Yangtze River Estuary population and Hongze Lake population in generations; t2: time of divergence between Yangtze River Estuary population and Chaohu Lake population in generations; t3: time of divergence between Yangtze River Estuary population and Taihu Lake population in generations.

Table S6 Numbers of outlier SNPs detected by Fisher's exact test and pcadapt for four anadromous-freshwater population pairs.

	FET	Pcadapt	Both
Taihu Lake vs. Yangtze River Estuary	1,716	1,903	1,568
Chaohu Lake vs. Yangtze River Estuary	1,885	2,145	1,720
Hongze Lake vs. Yangtze River Estuary	4,484	8,530	1,771
Luoma Lake vs. Yangtze River Estuary	3,618	11,401	1,849
Both	1,269	1,188	1,147

FET: Fisher's exact test.

Table S7 Annotations of genes in two inversion regions related to parallel adaptation (LG6 and LG22).

LG6				LG22			
ID	Gene name	start position	end position	ID	Gene name	start position	end position
DJ_025334-T1	Homeobox domain-containing protein	474129	497526	DJ_018359-T1	Centrosomal protein 85, like	27157	43319
DJ_025335-T1	Sclerostin domain-containing 1b	554406	555905	DJ_018360-T1	Centrosomal protein 85, like	49771	53832
DJ_025336-T1	Uncharacterized protein	560306	563296	DJ_018362-T1	Mediator of RNA polymerase II transcription subunit 28	58286	62545
DJ_025337-T1	Ankyrin repeat and MYND domain containing 2a	581282	583342	DJ_018363-T1	Oocyte-specific F-box protein	65951	79150
DJ_025338-T1	ankyrin repeat and MYND domain-containing protein 2-like isoform X3	588710	593465	DJ_018364-T1	Solute carrier family 35 member F1	84622	108815
DJ_025339-T1	Transforming growth factor-beta receptor type II	596202	607452	DJ_018365-T1	solute carrier family 35 member F1	111967	121729
DJ_025340-T1	RNA binding motif single stranded interacting protein 3	655737	660912	DJ_018366-T1	Pepsinogen A1	228112	234336
DJ_025341-T1	hypothetical protein	689782	691634	DJ_018367-T1	Nogo-B receptor	251814	265205
DJ_025342-T1	RNA binding motif single stranded interacting protein 3	745518	755139	DJ_018368-T1	podocan-like	265796	267956
DJ_025343-T1	Terminal nucleotidyltransferase 5Bb	829900	837681	DJ_018369-T1	Glycoprotein integral membrane 1	269505	275535
DJ_025344-T1	hypothetical protein	876645	880498	DJ_018370-T1	p60 katanin	278895	285375
DJ_025345-T1	Palmitoyltransferase	881893	894238	DJ_018371-T1	cytospin-A-like isoform X1	288777	297523
DJ_025346-T1	uncharacterized protein LOC106588636 isoform X3	895886	900795	DJ_018372-T1	Cytospin-A	298310	299655
DJ_025347-T1	uncharacterized protein LOC106588636 isoform X3	902085	914991	DJ_018373-T1	Si:ch211-147d7.5	300824	301174
DJ_025348-T1	AT-rich interactive domain 1Ab (SWI-like)	915718	932431	DJ_018374-T1	protein C10	302887	305267
DJ_025349-T1	Uncharacterized protein	932929	938187	DJ_018375-T1	Prostaglandin E receptor 2b (subtype EP2)	313740	315129
DJ_025350-T1	AT rich interactive domain 1Ab (SWI-like)	953897	977850	DJ_018377-T1	Epithelial-stromal interaction protein 1	321407	322872
DJ_025352-T1	T-box protein ZfT-A	1031793	1034466	DJ_018384-T1	zinc finger protein 36, C3H1 type-like 1	358058	360286
DJ_025353-T1	Small heterodimer partner-1	1055751	1056852	DJ_018385-T1	DNA repair protein RAD51 homolog 2 isoform X2	391162	410744
DJ_025354-T1	Keratinocyte differentiation factor 1b	1060539	1065803	DJ_018386-T1	Uncharacterized protein	413124	415029

DJ_025355-T1	Trophoblast glycoprotein-like	1088379	1089802	DJ_018387-T1	structural maintenance of chromosomes protein 6 isoform X2	415670	428593
DJ_025356-T1	Uncharacterized protein	1095927	1098594	DJ_018388-T1	structural maintenance of chromosomes protein 6 isoform X1	432662	446605
DJ_025357-T1	hypothetical protein	1099299	1105383	DJ_018389-T1	Transmembrane protein 18	449196	450396
DJ_025358-T1	SH3 domain-binding glutamate-rich protein-like 3	1115630	1118083	DJ_018390-T1	Uncharacterized protein	497096	498110
DJ_025359-T1	Syntaxin-12	1127149	1140667	DJ_018391-T1	Acid phosphatase 1	500372	514028
DJ_025360-T1	hypothetical protein	1160172	1163329	DJ_018392-T1	Si:ch211-57i17.5	531279	534911
DJ_025361-T1	hypothetical protein	1181561	1185309	DJ_018393-T1	Fasciculation and elongation protein zeta 2 (Zygin II)	541077	553669
DJ_025362-T1	Cellular communication network factor 2b	1188399	1192765	DJ_018394-T1	Si:dkey-260n20.1	558104	573628
DJ_025363-T1	Erythrocyte membrane protein band 4.1a	1228770	1233495	DJ_018395-T1	MYT1L	576575	582713
DJ_025364-T1	SEA domain-containing protein	1260484	1275414	DJ_018396-T1	hypothetical protein	586052	591666
DJ_025366-T1	Aspartate beta-hydroxylase domain-containing 1	1539086	1539773	DJ_018397-T1	Si:dkey-260n20.1	592292	600937
DJ_025367-T1	Seizure-related 6 homolog (mouse)-like 2	1547566	1554257	DJ_018399-T1	Ribonuclease H1	687859	691493
DJ_025368-T1	seizure 6-like protein 2	1555518	1587109	DJ_018400-T1	probable allantoicase	692712	706431
DJ_025369-T1	BTB/POZ domain-containing adapter for CUL3-mediated RhoA degradation protein 3	1602512	1620427	DJ_018401-T1	Uncharacterized protein	707428	716223
DJ_025370-T1	HIRA-interacting protein 3	1642807	1645887	DJ_018405-T1	Zinc finger and SCAN domain-containing protein 29	866919	877320
DJ_025371-T1	Zgc:92313	1658882	1662700	DJ_018409-T1	hypothetical protein	1073709	1076409
DJ_025373-T1	A2M_recep domain-containing protein	1729045	1730142	DJ_018410-T1	RBR-type E3 ubiquitin transferase	1087105	1092015
DJ_025374-T1	Alpha-2-macroglobulin-2	1750858	1753008	DJ_018411-T1	RBR-type E3 ubiquitin transferase	1093084	1094160
DJ_025375-T1	Si:ch211-212c13.10	1756227	1757614	DJ_018412-T1	F-box protein 25	1099493	1101515
DJ_025376-T1	hypothetical protein	1761790	1762740	DJ_018413-T1	F-box only protein 25 isoform X1	1106251	1119361
DJ_025377-T1	Uncharacterized protein	1773052	1778624	DJ_018414-T1	Si:ch211-225h24.2	1123191	1124025
DJ_025378-T1	Nucleoporin 98 and 96 precursor	1787884	1791703	DJ_018415-T1	hypothetical protein	1140689	1142970
DJ_025379-T1	Nucleoporin 98 and 96 precursor	1810133	1849487	DJ_018416-T1	Uncharacterized protein	1152427	1153719
DJ_025380-T1	Pigment epithelium-derived factor	1912726	1927562	DJ_018417-T1	tudor domain-containing protein 6	1181246	1191679
DJ_025381-T1	Zgc:66100 protein	2021729	2027803	DJ_018418-T1	Platelet-activating factor acetylhydrolase	1193377	1202859
DJ_025382-T1	uncharacterized protein LOC106562934	2042586	2043837	DJ_018419-T1	Metalloendopeptidase	1204075	1231629
DJ_025383-T1	Fatty-acid binding protein 3b	2054228	2055901	DJ_018420-T1	Adhesion G protein-coupled receptor F3b	1233437	1244651

DJ_025384-T1	Glucocorticoid modulatory element-binding protein 1	2210918	2222351	DJ_018421-T1	adhesion G-protein coupled receptor F1	1255125	1265352
DJ_025385-T1	Zgc:73061	2228919	2230793	DJ_018422-T1	Transforming growth factor beta	1289853	1295640
DJ_025386-T1	CTP synthase 1a	2236515	2238600	DJ_018423-T1	Feline leukemia virus subgroup C cellular receptor family member 2	1309679	1337798
DJ_025387-T1	UTP--ammonia ligase	2240532	2242831	DJ_018424-T1	B-cell-activating transcription factor	1344311	1351961
DJ_025388-T1	UTP--ammonia ligase	2243185	2250883	DJ_018425-T1	adhesion G protein-coupled receptor F5-like isoform X2	1360156	1366710
DJ_025389-T1	schlafen-like protein 1 isoform X1	2341709	2356435	DJ_018428-T1	Solute carrier family 2 member 12	1426178	1434694
DJ_025390-T1	hypothetical protein	2375049	2375707	DJ_018429-T1	TATA box-binding 1	1438538	1444939
DJ_025391-T1	hypothetical protein	2473753	2475992	DJ_018430-T1	BHLH domain-containing protein	1479582	1480666
DJ_025392-T1	Protein phosphatase 1, regulatory subunit 8a	2482504	2491108	DJ_018432-T1	Eyes absent homolog	1593991	1597320
DJ_025393-T1	Serine/threonine-protein kinase pdik1l	2497544	2504901	DJ_018433-T1	Eyes absent homolog	1615665	1619803
DJ_025394-T1	Protein lin-28 homolog A	2513440	2520501	DJ_018434-T1	40S ribosomal protein S12	1622804	1626556
DJ_025395-T1	Mannosidase endo-alpha like	2529497	2543245	DJ_018435-T1	SH3 domain-binding glutamic acid-rich-like protein 3	1627723	1631331
DJ_025396-T1	hypothetical protein	2569608	2570441	DJ_018436-T1	SH3 domain-binding glutamic acid-rich-like protein 2	1640485	1650198
DJ_025397-T1	splicing factor 3A subunit 3	2579916	2593238	DJ_018437-T1	Lebercilin LCA5	1672204	1688380
DJ_025398-T1	UBIQUITIN_CONJUGAT_2 domain-containing protein	2597099	2612177	DJ_018438-T1	Pleckstrin homology domain-interacting protein	1714489	1763153
DJ_025399-T1	hypothetical protein	2625055	2630235	DJ_018439-T1	PH-interacting protein	1775497	1794013
DJ_025400-T1	Ubiquitin conjugating enzyme E2 E2	2648536	2664911	DJ_018440-T1	Similar to Tetraodon protein product CAG00085	1799835	1801507
DJ_025401-T1	Ubiquitin-conjugating enzyme E2 E1	2673366	2689562	DJ_018441-T1	Interleukin-1 receptor-associated kinase 1-binding protein 1 homolog	1804400	1806484
DJ_025402-T1	NF-kappa-B inhibitor-interacting Ras-like protein 1	2690972	2691923	DJ_018442-T1	Solute carrier family 25 member 47-A-like protein	1809283	1812878
DJ_025403-T1	Ribosomal protein L15	2693706	2697820	DJ_018443-T1	Mitochondrial basic amino acids transporter	1813863	1819501
DJ_025404-T1	Nuclear receptor subfamily 1, group D, member 2b	2711858	2718833	DJ_018444-T1	Mitochondrial basic amino acids transporter	1833769	1838778
DJ_025405-T1	Alpha-L-fucosidase	2722206	2726617	DJ_018445-T1	Non-specific serine/threonine protein kinase	1851728	1864904
DJ_025406-T1	Proline-rich coiled-coil 2A	2730344	2746021	DJ_018446-T1	hypothetical protein	1882747	1887323
DJ_025407-T1	Casein kinase II subunit beta	2750759	2754504	DJ_018447-T1	pre-mRNA-splicing regulator WTAP isoform X2	1891460	1899164
DJ_025408-T1	Sperm acrosome associated 4 like	2772426	2773129	DJ_018448-T1	Superoxide dismutase	1901204	1904205
DJ_025409-T1	UPAR/Ly6 domain-containing protein	2779868	2781287	DJ_018449-T1	Superoxide dismutase	1904247	1905654

DJ_025410-T1	UPAR/Ly6 domain-containing protein	2787481	2789953	DJ_018450-T1	Fibronectin type III domain-containing 1	1977284	1983452
DJ_025411-T1	Chloride intracellular channel protein	2794490	2799589	DJ_018451-T1	Fibronectin type III domain-containing 1	1985541	2022246
DJ_025412-T1	NG,NG-dimethylarginine dimethylaminohydrolase 2	2815725	2822397	DJ_018452-T1	Fibronectin type III domain-containing 1-like protein	2025508	2057909
DJ_025413-T1	Ribosomal_S7 domain-containing protein	2824556	2828510	DJ_018453-T1	Otoferlin a	2117946	2133414
DJ_025414-T1	PPT2A	2832656	2837995	DJ_018454-T1	Otoferlin a	2139171	2145307
DJ_025415-T1	Novel protein similar to vertebrate carnitine acetyltransferase (CRAT)	2839393	2852871	DJ_018455-T1	Otoferlin a	2149098	2153294
DJ_025416-T1	nurim	2866612	2870968	DJ_018456-T1	Otoferlin	2161396	2185378
DJ_025417-T1	CUB domain-containing protein 1 isoform X1	2873074	2885663	DJ_018457-T1	Protein disulfide isomerase-related protein P5	2187313	2198239
DJ_025418-T1	C-type lectin domain-containing protein	2887322	2888748	DJ_018458-T1	Hippocalcin-like protein 1	2204120	2206236
DJ_025419-T1	Exosome complex exonuclease RRP42	2893062	2897299	DJ_018459-T1	apolipoprotein B-100 isoform X2	2234407	2252357
DJ_025420-T1	Palmitoyltransferase	2901991	2907926	DJ_018460-T1	Apolipoprotein Bb	2253445	2273066
DJ_025421-T1	transmembrane protein 42	2916188	2917702	DJ_018461-T1	Apolipoprotein Bb, tandem duplicate 2	2273194	2275809
DJ_025422-T1	Oxysterol-binding protein	2927483	2966115	DJ_018463-T1	apolipoprotein B-100 isoform X2	2285382	2303651
DJ_025423-T1	Oxysterol-binding protein	2996668	3005342	DJ_018464-T1	Uncharacterized protein	2307168	2321248
DJ_025424-T1	Protein-UDP acetylgalactosaminyltransferase	3037637	3042689	DJ_018466-T1	hypothetical protein	2367623	2373776
DJ_025425-T1	Adenosine deaminase 2b	3049648	3054412	DJ_018467-T1	MAM domain containing glycosylphosphatidylinositol anchor 2	2394244	2419100
DJ_025426-T1	Protein-UDP acetylgalactosaminyltransferase	3060881	3065536	DJ_018468-T1	hypothetical protein	2455535	2458757
DJ_025427-T1	Protein-UDP acetylgalactosaminyltransferase	3068415	3071059	DJ_018469-T1	MAM domain containing glycosylphosphatidylinositol anchor 2	2468912	2471997
DJ_025428-T1	adenosine deaminase CECR1-A-like	3071748	3077057	DJ_018470-T1	MAM domain containing glycosylphosphatidylinositol anchor 2	2483463	2504450
DJ_025429-T1	ATPase H+ transporting V1 subunit E1b	3080550	3098510	DJ_018471-T1	MDGA2B	2523180	2529928
DJ_025430-T1	VATE1	3101813	3111647	DJ_018472-T1	hypothetical protein	2818901	2822655
DJ_025431-T1	ATPase, H+ transporting, lysosomal 31kDa, V1 subunit E1, transcript variant X2	3111689	3113185	DJ_018473-T1	WD repeat domain 20	2825555	2833122
DJ_025432-T1	protein LCHN isoform X1	3118462	3124814	DJ_018474-T1	Zinc finger protein 341	2833998	2837027
DJ_025433-T1	Component of oligomeric golgi complex 5	3126640	3127448	DJ_018475-T1	HSP90	2838941	2843131
DJ_025434-T1	Guanylate cyclase	3134053	3150555	DJ_018476-T1	HSP90AA1	2845432	2850937
DJ_025435-T1	Sensor of single-strand DNA complex subunit A	3157905	3167841	DJ_018477-T1	Serine/threonine protein phosphatase 2A regulatory subunit	2852115	2858671

DJ_025436-T1	integrator complex subunit 3 isoform X5	3170451	3173892	DJ_018478-T1	Serine/threonine protein phosphatase 2A regulatory subunit	2869278	2883184
DJ_025437-T1	Sensor of single-strand DNA complex subunit A	3175273	3180447	DJ_018480-T1	Iodothyronine deiodinase	2920145	2920948
DJ_025438-T1	Ugly duckling 2 variant 1	3193265	3195022	DJ_018481-T1	Non-specific serine/threonine protein kinase	3016668	3022815
DJ_025439-T1	GON-4-like protein	3195061	3201690	DJ_018482-T1	Serine/threonine-protein kinase Sgk1	3067224	3071582
DJ_025440-T1	Protein disulfide-isomerase	3203991	3209787	DJ_018483-T1	hypothetical protein	3080263	3084840
DJ_025441-T1	Tyrosyl-tRNA synthetase	3220898	3231514	DJ_018484-T1	hypothetical protein	3141161	3142100
DJ_025442-T1	Si:ch211-194e15.5	3233631	3242383	DJ_018485-T1	hypothetical protein	3171986	3174904
DJ_025443-T1	Syncoilin, intermediate filament protein	3299332	3304447	DJ_018486-T1	Histone deacetylase	3186894	3203255
DJ_025444-T1	Novel protein similar to vertebrate basic leucine zipper and W2 domains 2 (BZW2) (Zgc:55580)	3330133	3335711	DJ_018487-T1	Histone deacetylase	3213472	3226089
DJ_025445-T1	ankyrin repeat and MYND domain-containing protein 2-like isoform X3	3348227	3360431	DJ_018488-T1	AD domain-containing protein	3259445	3261661
DJ_025446-T1	Sclerostin domain-containing 1b	3372978	3374494	DJ_018489-T1	Si:ch211-237i5.4	3265779	3268265
DJ_025447-T1	hypothetical protein	3446656	3447550	DJ_018490-T1	N-acetylglutamate synthase, mitochondrial	3273885	3283935
DJ_025448-T1	Integrase catalytic domain-containing protein	3449627	3451363	DJ_018491-T1	N-acetylglutamate synthase, mitochondrial	3305447	3306081
DJ_025449-T1	HERPUD family member 2	3464345	3471976	DJ_018492-T1	Beta-1,4-N-acetyl-galactosaminyl transferase 2, tandem duplicate 2	3307175	3336582
DJ_025450-T1	Anion exchange protein	3486555	3505571	DJ_018493-T1	hypothetical protein	3343333	3344738
DJ_025451-T1	Anion exchange protein	3512896	3519004	DJ_018494-T1	hypothetical protein	3453149	3453888
DJ_025452-T1	Anion exchange protein	3519822	3524939	DJ_018495-T1	Collagen alpha-1(I) chain	3459907	3466862
DJ_025453-T1	Eomesodermin	3559776	3563254	DJ_018496-T1	Collagen 1a1-like	3475708	3479582
DJ_025454-T1	hypothetical protein	3599233	3609647	DJ_018497-T1	Type I collagen	3479606	3481986
DJ_025455-T1	hypothetical protein	3626470	3645117	DJ_018498-T1	Protein phosphatase 1 regulatory subunit 9B	3490975	3495417
DJ_025456-T1	hypothetical protein	3650165	3654919	DJ_018499-T1	hypothetical protein	3508351	3510520
DJ_025457-T1	hypothetical protein	3706736	3714086	DJ_018500-T1	neurabin-2	3543717	3565264
DJ_025458-T1	Low density lipoprotein receptor adaptor protein 1a	3774608	3787923	DJ_018501-T1	rap guanine nucleotide exchange factor-like 1 isoform X1	3567257	3574560
DJ_025459-T1	Transmembrane protein 57a	3791759	3795316	DJ_018502-T1	Rap guanine nucleotide exchange factor (GEF)-like 1	3585580	3597494
DJ_025460-T1	Transmembrane protein 57a	3807777	3821747	DJ_018503-T1	Uncharacterized protein	3599077	3601054
DJ_025461-T1	serum response factor-binding protein 1	3822547	3825839	DJ_018504-T1	Rap guanine nucleotide exchange factor (GEF)-like 1	3652437	3654530

DJ_025462-T1	E2F transcription factor 3	3826701	3837290	DJ_018505-T1	protein CASC3	3672200	3677793
DJ_025463-T1	hypothetical protein	3886905	3903007	DJ_018507-T1	hypothetical protein	3680915	3684632
DJ_025464-T1	hypothetical protein	3903131	3903601	DJ_018508-T1	Btz domain-containing protein	3687461	3693113
DJ_025465-T1	hypothetical protein	4037499	4042516	DJ_018509-T1	PEHE domain-containing protein	3697395	3703084
DJ_025467-T1	hypothetical protein	4144763	4151290	DJ_018510-T1	Nuclear receptor subfamily 1, group d, member 1	3711442	3722763
DJ_025468-T1	Gastrula zinc finger protein XlCGF8.2DB	4191101	4204460	DJ_018511-T1	Nuclear receptor subfamily 1, group D, member 1	3723262	3724413
DJ_025469-T1	uncharacterized protein LOC108278005	4361286	4364277	DJ_018512-T1	Uncharacterized protein	3729874	3733421
DJ_025470-T1	Neurensin 1-like	4409697	4411064	DJ_018513-T1	Uncharacterized protein	3734757	3747227
DJ_025471-T1	Acyltransferase-like 2	4422500	4456111	DJ_018514-T1	Insulin-like growth factor 2 mRNA-binding protein 1	3759493	3779951
DJ_025472-T1	Iroquois homeobox 4	4486163	4491081	DJ_018515-T1	hypothetical protein	3785089	3786321
DJ_025473-T1	Iroquois homeobox protein 1, b	4558504	4560938	DJ_018516-T1	insulin-like growth factor 2 mRNA-binding protein 1	3803347	3810078
DJ_025474-T1	hypothetical protein	4566272	4571122	DJ_018517-T1	UBIQUITIN_CONJUGAT_2 domain-containing protein	3835417	3844516
DJ_025475-T1	hypothetical protein	4571387	4572948	DJ_018518-T1	ATP synthase lipid-binding protein, mitochondrial	3848633	3850878
DJ_025476-T1	UBIQUITIN_CONJUGAT_2 domain-containing protein	4693653	4700586	DJ_018519-T1	Tubulin tyrosine ligase-like family member 6a	3880773	3891393
DJ_025477-T1	SAM_MT_RSMB_NOP domain-containing protein	4718083	4735132	DJ_018520-T1	hypothetical protein	3894632	3907518
DJ_025478-T1	tRNA (Cytosine(34)-C(5))-methyltransferase	4735465	4739934	DJ_018521-T1	Homeobox domain-containing protein	3910470	3915738
DJ_025479-T1	tRNA (Cytosine(34)-C(5))-methyltransferase	4740734	4747008	DJ_018522-T1	Homeobox protein HoxB13aa	3930346	3931575
DJ_025480-T1	3-oxo-5-alpha-steroid 4-dehydrogenase (NADP(+))	4747641	4752361	DJ_018523-T1	Homeobox domain-containing protein	3955635	3958346
DJ_025481-T1	Non-canonical poly(A) RNA polymerase PAPD7	4757025	4769237	DJ_018524-T1	Homeobox protein	3964993	3969241
DJ_025482-T1	Non-canonical poly(A) RNA polymerase PAPD7	4772816	4774500	DJ_018525-T1	homeobox protein HoxB8ab isoform X2	3977594	3979164
DJ_025484-T1	Adenylate cyclase	4842008	4863302	DJ_018526-T1	Homeobox domain-containing protein	3981178	3985130
DJ_025485-T1	Adenylate cyclase	4876856	4888598	DJ_018527-T1	Homeobox domain-containing protein	3993491	3995449
DJ_025486-T1	hypothetical protein	4944029	4946371	DJ_018528-T1	Homeobox protein HoxB5aa	3997980	3999493
DJ_025487-T1	thiopurine S-methyltransferase	4956847	4962144	DJ_018529-T1	Homeobox domain-containing protein	4013098	4014797
DJ_025488-T1	protein phosphatase 1 regulatory subunit 36 isoform X1	4966240	4972194	DJ_018530-T1	hypothetical protein	4035131	4035950
DJ_025489-T1	Syndecan	5008075	5009332	DJ_018531-T1	Homeobox B3	4038830	4040799

DJ_025490-T1	Syndecan	5010532	5013419	DJ_018532-T1	homeobox protein Hox-B2a-like	4044787	4046919
DJ_025493-T1	hypothetical protein	5143422	5144288	DJ_018533-T1	Homeo box B1a	4067869	4069780
DJ_025494-T1	Fibrinogen C-terminal domain-containing protein	5181631	5186519	DJ_018534-T1	hypothetical protein	4090361	4093715
DJ_025495-T1	angiopoietin-1 isoform X1	5188486	5212312	DJ_018535-T1	hypothetical protein	4094721	4095853
DJ_025496-T1	Zgc:92005 protein	5236750	5238483	DJ_018536-T1	hypothetical protein	4180569	4181200
DJ_025497-T1	Jacalin-type lectin domain-containing protein	5253920	5254940	DJ_018537-T1	hypothetical protein	4296121	4298036
DJ_025498-T1	Jacalin-type lectin domain-containing protein	5256249	5257462	DJ_018538-T1	hypothetical protein	4370833	4375321
DJ_025499-T1	Zymogen granule membrane protein 16	5265416	5267190	DJ_018539-T1	CBX1	4391404	4400944
DJ_025500-T1	Sterile alpha motif domain containing 12	5269401	5289339	DJ_018540-T1	Nuclear factor, erythroid 2-like 1a	4406622	4416541
DJ_025501-T1	Exostosin glycosyltransferase 1	5460200	5482334	DJ_018541-T1	Coatomer subunit zeta	4419323	4426948
DJ_025502-T1	Mediator of RNA polymerase II transcription subunit 30	5510336	5513365	DJ_018542-T1	hypothetical protein	4510153	4517840
DJ_025503-T1	Solute carrier family 30 member 8	5517726	5526317	DJ_018543-T1	Oxysterol-binding protein	4537677	4554451
DJ_025504-T1	Si:ch211-153f2.7	5536169	5539315	DJ_018544-T1	TBD domain-containing protein	4558059	4569872
DJ_025505-T1	Si:ch211-153f2.3	5542357	5543180	DJ_018545-T1	TBK1 binding protein 1	4570548	4571556
DJ_025506-T1	double-strand-break repair protein rad21 homolog isoform X2	5547245	5555254	DJ_018546-T1	Protein-serine/threonine kinase	4589856	4598266
DJ_025507-T1	UTP23, small subunit (SSU) processome component, homolog	5557147	5558401	DJ_018547-T1	hypothetical protein	4605004	4609313
DJ_025508-T1	hypothetical protein	5646196	5648021	DJ_018548-T1	C1q domain-containing protein	4662057	4665872
DJ_025509-T1	acyl-protein thioesterase 2 isoform X2	5694869	5701456	DJ_018549-T1	hypothetical protein	4738159	4739233
DJ_025510-T1	PITH domain-containing protein 1	5702263	5705747	DJ_018550-T1	Uncharacterized protein	4741433	4743097
DJ_025511-T1	Uncharacterized protein	5718714	5721052	DJ_018551-T1	Uncharacterized protein	4743255	4746712
DJ_025512-T1	60S ribosomal protein L11	5737297	5747147	DJ_018552-T1	hypothetical protein	4752357	4754130
DJ_025513-T1	Elongation factor 1-alpha	5749288	5754911	DJ_018553-T1	uncharacterized protein LOC106593263	4761391	4763403
DJ_025514-T1	Elongation factor 1-alpha	5756106	5758887	DJ_018554-T1	obscurin-like	4764274	4791185
DJ_025515-T1	sialin-like	5769667	5779172	DJ_018555-T1	hypothetical protein	4803506	4804348
DJ_025516-T1	Palmitoyl-thioesterase 1-like protein	5781629	5784542	DJ_018556-T1	Uncharacterized protein	4808397	4811706
DJ_025517-T1	palmitoyl-protein thioesterase 1 isoform X2	5785478	5790163	DJ_018557-T1	Uncharacterized protein	4814436	4818611
DJ_025518-T1	Adenylyl cyclase-associated protein	5792500	5807286	DJ_018558-T1	Peptidylprolyl isomerase	4820774	4824513
DJ_025519-T1	Si:ch211-193k19.1	5811315	5830270	DJ_018559-T1	40S ribosomal protein S30	4825661	4827523

DJ_025520-T1	neurochondrin	5833482	5838971	DJ_018560-T1	pre-mRNA-processing factor 39	4829342	4837853
DJ_025521-T1	TF_AP-2 domain-containing protein	5840825	5848808	DJ_018561-T1	Pellino E3 ubiquitin protein ligase family member 2	4861798	4875584
DJ_025522-T1	Proteasome subunit beta	5852171	5860611	DJ_018562-T1	homeobox protein OTX2	4913011	4914516
DJ_025523-T1	UPF0500 protein C1orf216 homolog isoform X1	5868006	5870604	DJ_018563-T1	N(alpha)-acetyltransferase 30, NatC catalytic subunit	4952049	4958378
DJ_025524-T1	hypothetical protein	5903846	5907013	DJ_018564-T1	Uncharacterized protein	4970673	4973416
DJ_025525-T1	protein phosphatase 1 regulatory subunit 11	5909518	5911132	DJ_018565-T1	Actin-related protein 10	4980441	4990341
DJ_025526-T1	valine--tRNA ligase, mitochondrial isoform X2	5911956	5916514	DJ_018566-T1	CLOCK-interacting pacemaker	5005636	5015230
DJ_025527-T1	valine--tRNA ligase, mitochondrial isoform X2	5917392	5930276	DJ_018567-T1	Uncharacterized protein	5021541	5028615
DJ_025528-T1	alpha-1,3-mannosyl-glycoprotein 2-beta-N-acetylglucosaminyltransferase-like isoform X1	5935752	5944250	DJ_018568-T1	ectonucleoside triphosphate diphosphohydrolase 5-like isoform X2	5030376	5043700
DJ_025529-T1	spliceosome RNA helicase DDX39B isoform X1	5947874	5955553	DJ_018569-T1	Methylmalonate-semialdehyde dehydrogenase	5047969	5064791
DJ_025530-T1	General transcription factor IIH subunit 4	5959598	5960214	DJ_018570-T1	Sterile alpha motif domain-containing protein 15	5068927	5071728
DJ_025531-T1	tumor necrosis factor-like	5962993	5964956	DJ_018571-T1	Leucine rich repeat and fibronectin type III domain containing 5	5084394	5098575
DJ_025532-T1	General transcription factor IIH subunit 4	5967797	5979945	DJ_018572-T1	transcriptional repressor protein YY1-like isoform X2	5111470	5120343
DJ_025533-T1	General transcription factor IIH, polypeptide 4	5980838	5984777	DJ_018573-T1	Enah/Vasp-like b	5123756	5129807
DJ_025534-T1	hypothetical protein	5995165	6002177	DJ_018574-T1	Enah/Vasp-like	5140044	5147815
DJ_025535-T1	Gamma-aminobutyric acid (GABA) B receptor, 1b	6030069	6034488	DJ_018575-T1	Uncharacterized protein	5174214	5184281
DJ_025536-T1	ANF_receptor domain-containing protein	6070695	6073850	DJ_018576-T1	Bromo adjacent homology domain-containing 1 protein	5191301	5192767
DJ_025538-T1	G_PROTEIN_RECEP_F3_4 domain-containing protein	6091019	6123898	DJ_018577-T1	BAH domain-containing protein	5192770	5197021
DJ_025539-T1	Tumor necrosis factor alpha-3	6139361	6141575	DJ_018579-T1	BHLH domain-containing protein	5216531	5235272
DJ_025540-T1	Suppressor of actin mutations 1-like protein A	6150524	6166439	DJ_018580-T1	Acetyl-CoA acetyltransferase, cytosolic	5242905	5250027
DJ_025541-T1	Suppressor of actin mutations 1-like protein A	6166865	6172107	DJ_018581-T1	kinesin-like protein KIF25 isoform X2	5252563	5254221
DJ_025542-T1	Acetyltransferase mec-17 homolog	6175308	6179975	DJ_018582-T1	kinesin-like protein KIF25 isoform X3	5260576	5261893
DJ_025543-T1	Si:ch211-152p11.4	6193515	6196392	DJ_018583-T1	SOUL1	5263773	5266798
DJ_025544-T1	regulator of G-protein signaling 1-like	6196530	6200726	DJ_018584-T1	Zgc:56136	5268090	5269842

DJ_025545-T1	large proline-rich protein BAG6 isoform X1	6204903	6226708	DJ_018585-T1	NHS-like protein 1	5276224	5284560
DJ_025546-T1	Thymidine kinase	6234516	6237059	DJ_018586-T1	NHS-like 1b	5285417	5325585
DJ_025547-T1	MHC class I antigen ZBA transcript variant 4	6240203	6244382	DJ_018587-T1	Lamin B receptor	5475555	5479179
DJ_025548-T1	Tropomyosin alpha-3 chain isoform X6	6248918	6257730	DJ_018588-T1	Tumor necrosis factor receptor superfamily member 3	5493161	5494378
DJ_025549-T1	Uncharacterized protein	6267788	6270198	DJ_018589-T1	hypothetical protein	5527444	5529425
DJ_025550-T1	Rho guanine nucleotide exchange factor (GEF) 1a	6276264	6309111	DJ_018590-T1	Si:ch211-249c2.1	5591991	5594413
DJ_025551-T1	Rho guanine nucleotide exchange factor (GEF) 1a	6318134	6323219	DJ_018591-T1	cilia- and flagella-associated protein 58	5662334	5666384
DJ_025552-T1	Si:dkey-240h12.4	6334582	6350885	DJ_018592-T1	hypothetical protein	5682440	5684392
DJ_025553-T1	Si:ch73-109i22.2	6367553	6377194	DJ_018593-T1	cilia- and flagella-associated protein 58	5685562	5689407
DJ_025554-T1	Signal sequence receptor subunit beta	6378456	6381606	DJ_018594-T1	Sortilin related VPS10 domain containing receptor 1	5726385	5765731
DJ_025555-T1	hypothetical protein	6390020	6391529	DJ_018595-T1	Sortilin related VPS10 domain containing receptor 1	5768835	5782435
DJ_025556-T1	hypothetical protein	6394098	6398922	DJ_018597-T1	Si:ch211-244b2.4	5882492	5889390
DJ_025557-T1	hypothetical protein	6425311	6431525	DJ_018598-T1	Aldehyde dehydrogenase family 8 member A1	5891634	5897665
DJ_025558-T1	hypothetical protein	6502661	6503080	DJ_018599-T1	HBS1-like translational GTPase	5901081	5925199
DJ_025559-T1	Growth factor receptor-bound protein 10a	6529986	6547605	DJ_018600-T1	Tr-type G domain-containing protein	5945194	5948096
DJ_025560-T1	DNA-binding protein RFX5 isoform X3	6562804	6565934	DJ_018601-T1	transcriptional activator Myb isoform X2	5959078	5970892
DJ_025561-T1	Regulatory factor X5	6566745	6572061	DJ_018602-T1	Uncharacterized protein	6118469	6119760
DJ_025562-T1	Proteasome subunit beta	6584960	6589212	DJ_018603-T1	uncharacterized protein LOC106607836 isoform X2	6162585	6170888
DJ_025563-T1	Zgc:153441 protein	6607864	6612519	DJ_018604-T1	Uridine-cytidine kinase-like 1	6181673	6192705
DJ_025564-T1	GATA-type domain-containing protein	6614827	6616233	DJ_018605-T1	hypothetical protein	6194673	6197633
DJ_025565-T1	Leucine-rich repeat-containing protein 14B	6618114	6621294	DJ_018607-T1	hypothetical protein	6229174	6240708
DJ_025566-T1	transcription termination factor 3, mitochondrial isoform X1	6625839	6629243	DJ_018608-T1	uncharacterized protein LOC108259935 isoform X2	6249953	6258398
DJ_025567-T1	phosphatidylserine synthase 1-like	6630128	6638762	DJ_018609-T1	zinc finger protein 512 isoform X2	6260563	6268082
DJ_025568-T1	Nucleoporin 153	6642095	6657208	DJ_018610-T1	hypothetical protein	6270349	6276950
DJ_025569-T1	nuclear pore complex protein Nup153 isoform X1	6660036	6667219	DJ_018611-T1	General transcription factor IIIC, polypeptide 2, beta	6276964	6306732
DJ_025570-T1	Stathmin domain containing 1	6670203	6679686	DJ_018612-T1	Solute carrier family 30 member 2	6312065	6323309

DJ_025571-T1	RNA binding motif protein 24	6679999	6682261	DJ_018613-T1	Uridine-cytidine kinase	6324255	6334020
DJ_025572-T1	Adenylyl cyclase-associated protein	6710284	6721976	DJ_018614-T1	Uridine-cytidine kinase	6337394	6347811
DJ_025573-T1	aurora kinase A and ninein-interacting protein isoform X2	6723880	6725459	DJ_018615-T1	Mitochondrial inner membrane protein MPV17	6362904	6367526
DJ_025574-T1	Vacuolar protein sorting-associated protein 28 homolog	6728075	6730566	DJ_018616-T1	hypothetical protein	6386509	6388016
DJ_025575-T1	Mitogen-activated protein kinase	6732740	6747472	DJ_018617-T1	Tripartite motif-containing 54	6388549	6405300
DJ_025576-T1	Glutamate receptor, ionotropic, N-methyl D-aspartate-associated protein 1a (glutamate binding)	6762136	6772917	DJ_018618-T1	Zgc:56703	6410248	6422494
DJ_025577-T1	transmembrane protein 249 isoform X1	6777325	6779053	DJ_018619-T1	Psmc6 protein	6425641	6430741
DJ_025578-T1	Regulation of nuclear pre-mRNA domain-containing protein 1A	6779927	6791665	DJ_018620-T1	cell growth regulator with RING finger domain protein 1 isoform X1	6431759	6434713
DJ_025579-T1	Regulation of nuclear pre-mRNA domain-containing protein 1A	6793128	6796431	DJ_018621-T1	Novel protein similar to vertebrate DDHD domain containing 1 (DDHD1)	6435295	6438253
DJ_025580-T1	Sodium/hydrogen exchanger	6800598	6801759	DJ_018622-T1	DDHD domain-containing protein	6438779	6445333
DJ_025581-T1	Sodium/hydrogen exchanger	6802514	6813009	DJ_018623-T1	syntaxin-binding protein 6 isoform X3	6448738	6453909
DJ_025582-T1	Uncharacterized protein	6817313	6819889	DJ_018624-T1	hypothetical protein	6491497	6493765
DJ_025583-T1	Cilia and flagella associated protein 69	6822156	6833218	DJ_018625-T1	Gap junction protein	6508250	6509705
DJ_025584-T1	Lysine demethylase 1B	6833696	6848019	DJ_018626-T1	MGC53823 protein	6511113	6513728
DJ_025585-T1	Galectin 17	6849320	6861280	DJ_018627-T1	Uncharacterized protein	6525221	6528193
DJ_025586-T1	VPS52 subunit of GARP complex	6863426	6866628	DJ_018628-T1	Myeloid ecotropic viral integration site 2.1	6550371	6570368
DJ_025587-T1	vacuolar protein sorting-associated protein 52 homolog	6866643	6874370	DJ_018629-T1	Potassium channel, subfamily K, member 10a	6616600	6624663
DJ_025588-T1	Ribosomal protein S18	6874682	6877652	DJ_018630-T1	Chromosome 14 open reading frame 132	6673094	6675765
DJ_025589-T1	Ring finger protein 1	6878550	6881884	DJ_018632-T1	Glutaredoxin 5 homolog	6722988	6724130
DJ_025590-T1	Solute carrier 39 (Zinc transporter) member 7	6901223	6906785	DJ_018633-T1	Spectrin repeat containing, nuclear envelope family member 3	6724847	6734824
DJ_025591-T1	Vacuolar protein sorting 45 homolog	6913882	6928989	DJ_018634-T1	Spectrin repeat-containing, nuclear envelope family member 3	6739732	6740437
DJ_025592-T1	CARD domain-containing protein	6935765	6962446	DJ_018635-T1	Calmin	6743778	6749138
DJ_025593-T1	splicing factor, proline- and glutamine-rich isoform X2	6963605	6971201	DJ_018636-T1	Serpina1 protein	6755160	6757538
DJ_025594-T1	Zinc finger MYM-type 4-like protein	6979421	6986590	DJ_018637-T1	hypothetical protein	6770621	6773105
DJ_025595-T1	Si:ch211-173p18.3	6989789	6998755	DJ_018638-T1	Protein FAM184A-like	6858616	6862836
DJ_025596-T1	zinc finger MYM-type protein 4	6998976	7011633	DJ_018639-T1	protein FAM184A-like isoform X1	6870571	6877039

DJ_025597-T1	Adenylate monophosphate kinase	7018818	7025331	DJ_018640-T1	hypothetical protein	6890162	6894363
DJ_025598-T1	RBR-type E3 ubiquitin transferase	7033996	7052123	DJ_018641-T1	hypothetical protein	6925420	6927647
DJ_025599-T1	NADH dehydrogenase [ubiquinone] iron-sulfur protein 5 isoform X1	7057575	7059144	DJ_018642-T1	hypothetical protein	6929974	6931446
DJ_025600-T1	Microtubule actin crosslinking factor 1a	7142387	7165878	DJ_018643-T1	hypothetical protein	6954429	6960253
DJ_025601-T1	Microtubule-actin cross-linking factor 1 isoform X1	7168204	7187110	DJ_018644-T1	FAM184 domain-containing protein	6979388	6982631
DJ_025602-T1	microtubule-actin cross-linking factor 1, isoforms 1/2/3/5-like isoform X19	7200106	7241105	DJ_018645-T1	MCM domain-containing protein	6993044	7021480
DJ_025603-T1	Microtubule-actin cross-linking factor 1	7254365	7311859	DJ_018646-T1	Centrosomal protein 85,-like	7034981	7036127
DJ_025604-T1	bone morphogenetic protein 8A	7316809	7325611	DJ_018647-T1	hypothetical protein	7040829	7042625
DJ_025605-T1	sodium/potassium-transporting ATPase subunit beta-1-interacting protein 1	7332031	7337389	DJ_018648-T1	hypothetical protein	7117309	7118296
DJ_025606-T1	Gamma-aminobutyric acid receptor subunit alpha-3-like protein	7345537	7352690	DJ_018649-T1	Zinc finger and SCAN domain-containing protein 29	7219861	7222593
DJ_025607-T1	Gamma-aminobutyric acid type A receptor subunit beta4	7387219	7421390	DJ_018650-T1	Pyruvate dehydrogenase E1 component subunit beta	7262914	7288616
DJ_025609-T1	Uncharacterized protein	7428219	7429791	DJ_018651-T1	Kelch-like family member 29	7433373	7433855
DJ_025610-T1	Membrane protein, palmitoylated 1	7434841	7445259	DJ_018652-T1	ATPase family AAA domain containing 2B	7470196	7472274
DJ_025612-T1	Trimethyllysine hydroxylase, epsilon	7451510	7461442	DJ_018653-T1	ATPase family AAA domain-containing 2B	7516163	7549457
DJ_025613-T1	DLLB	7468806	7470924	DJ_018654-T1	ATPase family AAA domain containing 2B	7549967	7553005
DJ_025614-T1	Exocyst complex component 3-like 2a	7486918	7499757	DJ_018655-T1	Vascular endothelial growth factor	7645513	7663610
DJ_025615-T1	Ribonucleoside-diphosphate reductase	7504181	7512017	DJ_018656-T1	Alpha-galactosidase	7701600	7710714
DJ_025616-T1	Ring finger protein 121	7513889	7524223	DJ_018657-T1	Adenylate cyclase type 3	7713912	7721517
DJ_025617-T1	FUN14 domain-containing protein 2	7526206	7530176	DJ_018658-T1	Uncharacterized protein	7734503	7753044
DJ_025618-T1	Doublecortin-like kinase 3	7531875	7535464	DJ_018659-T1	WDR26	7754491	7758386
DJ_025619-T1	Cysteine and histidine-rich protein 1	7563897	7567271	DJ_018660-T1	Zinc finger, AN1-type domain 3	7765626	7770090
DJ_025620-T1	Programmed cell death 6-interacting protein	7571445	7592308	DJ_018661-T1	28S ribosomal protein S18a, mitochondrial	7773343	7775672
DJ_025621-T1	CLIP-associating protein 2	7596589	7615717	DJ_018662-T1	radial spoke head protein 9 homolog	7776134	7777911
DJ_025622-T1	CLIP-associating protein 2 isoform X5	7623173	7647628	DJ_018663-T1	CSC1-like protein 1	7782745	7793587
DJ_025623-T1	Clasp2 protein	7667200	7670885	DJ_018664-T1	Alanine--glyoxylate aminotransferase	7807892	7816406
DJ_025624-T1	Cartilage-associated protein	7687756	7694712	DJ_018665-T1	Kinesin family member 1Ab	7828925	7836941
DJ_025625-T1	Peptidylprolyl isomerase	7695393	7703025	DJ_018666-T1	Kinesin family member 1Ab	7841620	7850503
DJ_025626-T1	PUM-HD domain-containing protein	7704959	7712634	DJ_018667-T1	Kinesin family member 1Aa	7850622	7867381

DJ_025627-T1	PUM-HD domain-containing protein	7716597	7734671	DJ_018668-T1	Espin-like b	7878268	7882463
DJ_025628-T1	Syndecan	7766408	7781277	DJ_018669-T1	Solute carrier organic anion transporter family member	7885036	7899737
DJ_025629-T1	Family with sequence similarity 83 member Hb	7788305	7794223	DJ_018670-T1	RAB41, member RAS oncogene family	7903049	7909305
DJ_025630-T1	Uncharacterized protein	7796155	7804971	DJ_018671-T1	Uncharacterized protein	7915181	7932923
DJ_025631-T1	hypothetical protein	7809704	7811433	DJ_018672-T1	Uncharacterized protein	7945546	7964177
DJ_025632-T1	WASP family member 2	7832632	7840405	DJ_018673-T1	Myosin VIIBb	7970892	7980235
DJ_025633-T1	AT hook, DNA-binding motif,-containing 1	7853624	7857829	DJ_018674-T1	Myosin VIIBb	7981431	7995854
DJ_025634-T1	Tyrosine-protein kinase	7885421	7889870	DJ_018675-T1	FGFR1 oncogene partner	8005794	8024678
DJ_025635-T1	Tyrosine-protein kinase	7895186	7900550	DJ_018677-T1	Discs, large (Drosophila) homolog-associated protein 2b	8042124	8043601
DJ_025636-T1	cartilage matrix protein isoform X1	7920604	7928379	DJ_018678-T1	Discs, large (Drosophila) homolog-associated protein 2b	8049298	8056014
DJ_025637-T1	homeobox protein NOBOX	7940317	7944872	DJ_018679-T1	Uncharacterized protein	8065041	8085698
DJ_025638-T1	Shugoshin 1	7949205	7959816	DJ_018680-T1	DEAD (Asp-Glu-Ala-Asp) box helicase 1	8126887	8128417
DJ_025639-T1	Testis and ovary-specific PAZ domain-containing 1	7967558	7980411	DJ_018681-T1	ATP-dependent RNA helicase DDX1	8128898	8137206
DJ_025640-T1	Asteroid homolog 1	7982761	7987181	DJ_018683-T1	Nbas protein	8153723	8167035
DJ_025641-T1	WAS/WASL-interacting protein family member 3	7994076	8003453	DJ_018684-T1	Neuroblastoma-amplified gene protein homolog	8173747	8253043
DJ_025642-T1	3-hydroxyisobutyrate dehydrogenase	8028606	8051215	DJ_018685-T1	hypothetical protein	8286084	8288114
DJ_025643-T1	Even-skipped homeobox 1	8067450	8069964	DJ_018686-T1	Distal-less homeobox 4a	8318907	8323923
DJ_025644-T1	Homeobox protein Hox-A3a	8080710	8086761	DJ_018687-T1	Peptidase S1 domain-containing protein	8359195	8364617
DJ_025645-T1	Homeobox A1a	8091437	8092539	DJ_018688-T1	hypothetical protein	8390028	8394822
DJ_025646-T1	Sorting nexin 10a	8116120	8123759	DJ_018689-T1	hypothetical protein	8411998	8417589
DJ_025647-T1	chromobox protein-like protein 3 isoform X4	8130304	8134691	DJ_018690-T1	N-myc proto-oncogene protein	8471906	8475453
DJ_025648-T1	RFamide-related peptide	8137893	8144512	DJ_018691-T1	hypothetical protein	8475922	8476588
DJ_025649-T1	Oxysterol-binding protein	8152372	8173553	DJ_018692-T1	Uncharacterized protein	8532595	8540929
DJ_025650-T1	non-syndromic hearing impairment protein 5-like	8174321	8181245	DJ_018693-T1	Si:ch211-174j14.2	8544194	8546274
DJ_025651-T1	Phosphatidylinositol-4,5-bisphosphate 4-phosphatase	8219537	8241412	DJ_018694-T1	Uncharacterized protein	8549598	8566046
DJ_025652-T1	Deubiquitinase OTUD6B	8243222	8248654	DJ_018695-T1	Midasin	8595030	8598782
DJ_025653-T1	leucine-rich repeat-containing protein 69	8250100	8254052	DJ_018696-T1	hypothetical protein	8656919	8659960

DJ_025654-T1	RUNX1 partner transcriptional co-repressor 1	8285433	8314851	DJ_018697-T1	PP4R3	8685447	8690198
DJ_025655-T1	hypothetical protein	8372895	8375351	DJ_018698-T1	SMK-1 domain-containing protein	8690557	8704939
DJ_025656-T1	hypothetical protein	8626458	8632833	DJ_018699-T1	Terminal nucleotidyltransferase 5A	8734717	8737347
DJ_025657-T1	Family with sequence similarity 92 member A1	8654948	8656039	DJ_018700-T1	Very long chain 3-oxoacyl-CoA synthase 4	8785496	8794847
DJ_025658-T1	Family with sequence similarity 92 member A1	8657137	8657895	DJ_018701-T1	Tcp1 protein	8802135	8812215
DJ_025659-T1	Protein kinase domain-containing protein	8696624	8709527	DJ_018702-T1	Pleckstrin homology and RhoGEF domain containing G1	8817089	8834130
DJ_025660-T1	Protein FAM91A1	8750333	8766435	DJ_018703-T1	uncharacterized protein LOC106604860 isoform X3	8848550	8850061
DJ_025661-T1	Protein FAM91A1	8766816	8781797	DJ_018704-T1	Pleckstrin homology domain-containing family G member 1	8851450	8858740
DJ_025662-T1	Spire-type actin nucleation factor 1a	8800689	8826361	DJ_018705-T1	iodotyrosine deiodinase 1	8972421	8976439
DJ_025663-T1	V-type proton ATPase subunit C	8852090	8860824	DJ_018706-T1	protein phosphatase 1 regulatory subunit 14C	9018890	9020224
DJ_025664-T1	antizyme inhibitor 1-like isoform X1	8867285	8878050	DJ_018707-T1	C1q domain-containing protein	9030965	9036515
DJ_025665-T1	RIR2B	8880645	8884060	DJ_018710-T1	osteopetrosis-associated transmembrane protein 1 precursor	9053828	9062476
DJ_025666-T1	Lysosomal protein transmembrane 4 beta	8898918	8907088	DJ_018711-T1	Nuclear receptor subfamily 2, group E, member 1	9084880	9092245
DJ_025667-T1	hypothetical protein	8920981	8928347	DJ_018712-T1	Sorting nexin 3	9097170	9114574
DJ_025668-T1	ribonuclease UK114 isoform X1	8930064	8935275	DJ_018713-T1	AFG1 like ATPase b	9115807	9153806
DJ_025669-T1	Stk3 protein	8938347	8940707	DJ_018714-T1	Fork-head domain-containing protein	9160082	9162270
DJ_025670-T1	Sperm-associated antigen 1A	8949648	8961528	DJ_018715-T1	forkhead box protein O3	9204909	9211472
DJ_025671-T1	RBR-type E3 ubiquitin transferase	8976720	8980417	DJ_018716-T1	RPA-interacting protein A	9222300	9225856
DJ_025672-T1	Fatty acid binding protein H6-isoform	9041185	9043162	DJ_018718-T1	Armadillo repeat containing 2	9265818	9288527
DJ_025673-T1	Fatty acid binding protein H6-isoform	9047371	9048337	DJ_018719-T1	sestrin-1 isoform X2	9292444	9292922
DJ_025674-T1	39S ribosomal protein L53, mitochondrial	9055248	9056131	DJ_018720-T1	sestrin-1 isoform X3	9293217	9300076
DJ_025676-T1	Zinc finger protein 704	9147724	9155439	DJ_018721-T1	hypothetical protein	9300779	9302593
DJ_025677-T1	N-lysine methyltransferase SETD8	9194086	9202733	DJ_018722-T1	Selenoprotein I	9388763	9406466
DJ_025678-T1	Zinc finger protein 704	9222647	9236420	DJ_018723-T1	Si:ch211-197l9.5	9419652	9444675
DJ_025679-T1	hypothetical protein	9246994	9249064	DJ_018724-T1	T-box transcription factor TBX18	9513958	9528422
DJ_025680-T1	Tumor protein D52	9255717	9276150	DJ_018725-T1	hypothetical protein	9566825	9571279
DJ_025681-T1	Zinc finger and BTB domain containing 10	9307738	9310991	DJ_018726-T1	hypothetical protein	9595865	9599194
DJ_025682-T1	N-lysine methyltransferase SETD8	9312424	9329269	DJ_018727-T1	Uncharacterized protein	9727417	9728164

DJ_025684-T1	hypothetical protein	9441265	9444180	DJ_018728-T1	Ig-like domain-containing protein	9737637	9740068
DJ_025685-T1	hypothetical protein	9472438	9474729	DJ_018729-T1	Uncharacterized protein	9749554	9753100
DJ_025686-T1	CUB and sushi domain-containing protein 3	9482149	9482917	DJ_018730-T1	Obscurin	9754920	9766002
DJ_025687-T1	CUB and sushi domain-containing protein 3	9525134	9528255	DJ_018731-T1	Ig-like domain-containing protein	9777123	9785406
DJ_025688-T1	CUB and Sushi multiple domains 3a	9697375	9719929	DJ_018732-T1	Obscurin	9787521	9791304
DJ_025689-T1	CUB and sushi domain-containing protein 3	9739060	9769870	DJ_018733-T1	uncharacterized protein LOC106608106	9794876	9797247
DJ_025690-T1	CUB and Sushi multiple domains 3a	9798368	9851761	DJ_018734-T1	Cytosolic phospholipase A2 gamma	9805183	9819413
DJ_025691-T1	CUB and sushi domain-containing protein 3	9854708	9900781	DJ_018736-T1	Olfactory receptor C family, b1	9825364	9830932
DJ_025692-T1	CUB and sushi domain-containing protein 3	9912244	9931826	DJ_018738-T1	Olfactory receptor C family, b1	9851472	9867703
DJ_025693-T1	Cleft lip and palate transmembrane protein 1	9941050	9954472	DJ_018739-T1	DIS3-like exonuclease 2	9881452	9886201
DJ_025694-T1	hypothetical protein	9965007	9967366	DJ_018740-T1	DIS3-like exonuclease 2	9888392	9895317
DJ_025695-T1	FAK1	9984759	9995728	DJ_018743-T1	alpha-1,2-Mannosidase	10018175	10041710
DJ_025696-T1	focal adhesion kinase 1 isoform X5	10007112	10068145	DJ_018744-T1	alpha-1,2-Mannosidase	10135822	10158847
DJ_025697-T1	DENN/MADD domain containing 3a	10119652	10121656	DJ_018745-T1	alpha-1,2-Mannosidase	10209030	10234083
DJ_025698-T1	DENN domain-containing protein 3	10121714	10143437	DJ_018933-T1	Reticulon	15809776	15823381
DJ_025699-T1	Solute carrier family 45 member 4	10152650	10164392	DJ_018934-T1	Reticulon	15835792	15850738
DJ_025701-T1	Solute carrier family 45 member 4	10181983	10183973	DJ_018935-T1	Reticulon	15869326	15872364
DJ_025702-T1	hypothetical protein	10209552	10212946	DJ_018936-T1	Uncharacterized protein	15894943	15899131
DJ_025703-T1	G-protein coupled receptor 20 isoform X1	10221905	10223117	DJ_018937-T1	Protein phosphatase 1, regulatory subunit 13Bb	15918997	15950005
DJ_025704-T1	Ankyrin-3	10232084	10240993	DJ_018938-T1	Apoptosis-stimulating protein of p53	15974291	15978364
DJ_025705-T1	serine/threonine-protein phosphatase 6 regulatory ankyrin repeat subunit A-like	10241641	10269782	DJ_018939-T1	disheveled-associated activator of morphogenesis 1-like isoform X1	16039902	16053929
DJ_025706-T1	DUF667 domain-containing protein	10272043	10272852	DJ_018940-T1	disheveled-associated activator of morphogenesis 1-like isoform X1	16054777	16090674
DJ_025707-T1	O-acyltransferase	10274925	10293601	DJ_018941-T1	trans-3-hydroxy-L-proline dehydratase	16094024	16096407
DJ_025708-T1	Zgc:63863	10296470	10335517	DJ_018942-T1	Si:ch211-207i1.2	16108667	16122865
DJ_025709-T1	Zgc:172288 protein	10459850	10461871	DJ_018943-T1	nesprin-2-like isoform X10	16123464	16129954
DJ_025710-T1	Solute carrier family 39 member 4	10550836	10569667	DJ_018944-T1	nesprin-2-like isoform X8	16130586	16145867
DJ_025711-T1	Uncharacterized protein	10580013	10586650	DJ_018945-T1	Si:ch211-207i1.2	16151333	16219359
DJ_025712-T1	Si:ch211-57n23.1	10596839	10600333	DJ_018946-T1	Uncharacterized protein	16237823	16248497

DJ_025713-T1	Protein slicer	10620155	10637301	DJ_018948-T1	Putative estrogen receptor beta2 protein	16255898	16268410
DJ_025714-T1	Homeobox protein Dlx5a	10643119	10647784	DJ_018949-T1	Delta-like protein	16376872	16397932
DJ_025715-T1	Distal-less homeobox protein 6a	10649303	10650831	DJ_018950-T1	Delta-like protein	16404437	16431155
DJ_025716-T1	Uncharacterized protein	10691775	10697992	DJ_018952-T1	hypothetical protein	16441514	16442979
DJ_025717-T1	Solute carrier family 25 member 13	10714177	10718342	DJ_018953-T1	Si:dkey-77p13.2	16476185	16502336
DJ_025718-T1	MGC69168 protein	10724101	10737616	DJ_018954-T1	hypothetical protein	16522718	16527574
DJ_025719-T1	calcium-binding mitochondrial carrier protein Aralar2 isoform X2	10739764	10772076	DJ_018955-T1	Transcription factor IIIB 90 kDa subunit	16667010	16688696
DJ_025720-T1	hypothetical protein	10774664	10778482	DJ_018957-T1	Transcription factor IIIB 90 kDa subunit	16709480	16717036
DJ_025721-T1	Dynein cytoplasmic 1 intermediate chain 1	10784503	10789834	DJ_018958-T1	Transcription factor IIIB 90 kDa subunit	16736006	16740384
DJ_025722-T1	hypothetical protein	10790925	10792485	DJ_018959-T1	BRF1 RNA polymerase III transcription initiation factor subunit b	16740791	16743681
DJ_025723-T1	cytoplasmic dynein 1 intermediate chain 1 isoform X5	10798615	10809998	DJ_018960-T1	BTB (POZ) domain containing 6b	16747845	16749747
DJ_025724-T1	Dynein cytoplasmic 1 intermediate chain 1	10854879	10868210	DJ_018961-T1	Transcription factor IIIB 90 kDa subunit	16751617	16765626
DJ_025725-T1	Dynein, cytoplasmic 1, intermediate chain 1	10870594	10875712	DJ_018962-T1	uncharacterized protein LOC106571709 isoform X2	16765671	16776213
DJ_025726-T1	Protein-serine/threonine kinase	10879549	10889028	DJ_018963-T1	SUN domain-containing protein	16777387	16780847
DJ_025727-T1	Ankyrin repeat and SOCS box containing 4	10897676	10906847	DJ_018964-T1	Xrcc3 protein	16783468	16786900
DJ_025728-T1	neurabin-1 isoform X4	10911828	10925623	DJ_018965-T1	Creatine kinase, brain b	16851111	16855472
DJ_025729-T1	Protein phosphatase 1, regulatory subunit 9A	10935583	10945828	DJ_018966-T1	Non-specific serine/threonine protein kinase	16875689	16879174
DJ_025730-T1	Protein phosphatase 1, regulatory subunit 9A	10946064	10958744	DJ_018967-T1	Non-specific serine/threonine protein kinase	16887045	16907217
DJ_025732-T1	Sarcoglycan, epsilon	10989026	11004602	DJ_018968-T1	hypothetical protein	16911856	16914522
DJ_025734-T1	ST3 beta-galactoside alpha-2,3-sialyltransferase 1	11195017	11198128	DJ_018969-T1	hypothetical protein	16918832	16919752
DJ_025735-T1	Poly [ADP-ribose] polymerase	11199955	11215259	DJ_018971-T1	MAP/microtubule affinity-regulating kinase 3	16947852	16955319
DJ_025736-T1	Cleavage and polyadenylation-specific factor 1	11216560	11229432	DJ_018972-T1	Chloride intracellular channel protein	16966455	16976872
DJ_025737-T1	AarF domain-containing kinase 5	11238954	11251528	DJ_018973-T1	Runx2b splice form 4	16993396	16996505
DJ_025738-T1	RNA polymerase II subunit A C-terminal domain phosphatase	11253168	11290329	DJ_018974-T1	Runt-related transcription factor	16997890	17018675
DJ_025739-T1	hypothetical protein	11320549	11327714	DJ_018975-T1	hypothetical protein	17039709	17044735

DJ_025740-T1	hypothetical protein	11358548	11363295	DJ_018977-T1	Opsin 8, group member a	17139084	17146175
DJ_025741-T1	hypothetical protein	11377739	11380942	DJ_018979-T1	C-C motif chemokine	17166711	17167328
DJ_025744-T1	hypothetical protein	11496434	11497003	DJ_018980-T1	C-C motif chemokine	17178110	17183324
DJ_025745-T1	Metalloendopeptidase	11506062	11513382	DJ_018981-T1	Kinase	17237666	17243121
DJ_025746-T1	Metalloendopeptidase	11515682	11524253	DJ_018982-T1	Kinase	17247581	17258122
DJ_025747-T1	Si:ch211-182e10.4	11603243	11606121	DJ_018983-T1	Kinase	17270771	17273568
DJ_025748-T1	KH domain-containing protein	11699013	11710583	DJ_018984-T1	aarF domain-containing protein kinase 3	17286292	17311328
DJ_025749-T1	Ig-like domain-containing protein	11736895	11744186	DJ_018985-T1	Coenzyme Q8A, genome duplicate a	17313406	17316920
DJ_025750-T1	tetraspanin-13-like	11746392	11751447	DJ_018986-T1	uncharacterized protein LOC106571435 isoform X2	17318809	17325854
DJ_025751-T1	Anterior gradient protein 2 homolog	11755671	11758571	DJ_018987-T1	Uncharacterized protein	17330443	17333686
DJ_025752-T1	Serine/threonine-protein phosphatase 1 regulatory subunit 10	11765044	11772883	DJ_018988-T1	Uncharacterized protein	17348664	17354657
DJ_025753-T1	ATP-binding cassette, sub-family F (GCN20), member 1	11780230	11788005	DJ_018989-T1	Uncharacterized protein	17356650	17359618
DJ_025754-T1	Si:ch211-215a10.4	11792170	11795709	DJ_018990-T1	hypothetical protein	17359697	17361128
DJ_025756-T1	hypothetical protein	11843016	11845682	DJ_018991-T1	hypothetical protein	17370903	17379053
DJ_025757-T1	hypothetical protein	11866571	11870560	DJ_018994-T1	hypothetical protein	17502548	17509608
DJ_025759-T1	hypothetical protein	11894472	11897145	DJ_018995-T1	Protein kinase domain-containing protein	17538433	17557070
DJ_025760-T1	KH RNA binding domain containing, signal transduction associated 3	11929295	11943095	DJ_018996-T1	Uncharacterized protein	17656839	17658868
DJ_025761-T1	hypothetical protein	12072396	12076388	DJ_018997-T1	GDNF inducible zinc finger protein 1	17659879	17663306
DJ_025762-T1	hypothetical protein	12090877	12092252	DJ_018998-T1	NSF attachment protein beta	17673027	17683152
DJ_025763-T1	Family with sequence similarity 135 member B	12110615	12117838	DJ_018999-T1	Glycerophosphocholine phosphodiesterase 1	17706797	17717565
DJ_025764-T1	Family with sequence similarity 135 member B	12119105	12161103	DJ_019000-T1	Glycerophosphocholine phosphodiesterase 1	17717667	17725763
DJ_025765-T1	collagen alpha-1(IX) chain-like isoform X2	12195170	12211062	DJ_019001-T1	PPUP7386	17757395	17768077
DJ_025766-T1	hypothetical protein	12211965	12232367	DJ_019002-T1	tRNA (adenine(58)-N(1))-methyltransferase non-catalytic subunit TRM6	17778637	17780644
DJ_025767-T1	hypothetical protein	12232375	12234453	DJ_019004-T1	PH domain-containing protein	17783091	17809483
DJ_025768-T1	COMA1	12239255	12257897	DJ_019005-T1	Bone morphogenetic protein 2b	17874213	17879575
DJ_025769-T1	hypothetical protein	12257931	12275439	DJ_019006-T1	1-phosphatidylinositol 4,5-bisphosphate phosphodiesterase	17929093	17937202

DJ_025770-T1	Collagen type XXII alpha 1 chain	12277727	12304503	DJ_019008-T1	1-phosphatidylinositol 4,5-bisphosphate phosphodiesterase	18029382	18050425
DJ_025771-T1	hypothetical protein	12315291	12319636	DJ_019009-T1	1-phosphatidylinositol 4,5-bisphosphate phosphodiesterase	18062437	18079805
DJ_025772-T1	Potassium channel subfamily K member	12330687	12333775	DJ_019010-T1	hypothetical protein	18079866	18083647
DJ_025773-T1	hypothetical protein	12412928	12415178	DJ_019013-T1	ras-related protein Rab-15	18102037	18107663
DJ_025774-T1	Trafficking protein particle complex subunit 9 isoform B	12491854	12499767	DJ_019014-T1	Signal-induced proliferation-associated 1-like 1	18198541	18200042
DJ_025775-T1	Trafficking protein particle complex 9	12530142	12537752	DJ_019015-T1	Uncharacterized protein	18200098	18203373
DJ_025776-T1	Trafficking protein particle complex 9	12663837	12688922	DJ_019016-T1	Signal-induced proliferation-associated 1-like 1	18215973	18220917
DJ_025778-T1	Trafficking protein particle complex 9	12691100	12697362	DJ_019017-T1	Signal-induced proliferation-associated 1-like 1	18221189	18230374
DJ_025779-T1	Trafficking protein particle complex 9	12697797	12700687	DJ_019018-T1	signal-induced proliferation-associated 1-like protein 1 isoform X1	18230758	18242402
DJ_025780-T1	protein BTG4	12702707	12704104	DJ_019019-T1	Signal-induced proliferation-associated 1 like 1	18242475	18249437
DJ_025781-T1	hypothetical protein	12706014	12710342	DJ_019020-T1	Si:ch211-212k5.3	18337442	18338498
DJ_025782-T1	SEH1-like (S. cerevisiae)	12717358	12730082	DJ_019021-T1	Uncharacterized protein	18348661	18355481
DJ_025783-T1	hypothetical protein	12803758	12805482	DJ_019022-T1	HECT domain-containing protein	18375721	18379961
DJ_025784-T1	Low density lipoprotein receptor class A domain-containing 4a	12812780	12830460	DJ_019023-T1	Uncharacterized protein	18434722	18436395
DJ_025785-T1	DUF1279 domain-containing protein	12837788	12840818	DJ_019024-T1	Uncharacterized protein	18436952	18443332
DJ_025786-T1	mRNA cap guanine-N7 methyltransferase	12842870	12850146	DJ_019025-T1	Sorting nexin	18448632	18462632
DJ_025787-T1	Collagen triple helix repeat containing 1a	12893317	12902144	DJ_019026-T1	PX domain-containing protein	18463006	18466946
DJ_025788-T1	Solute carrier family 25, member 32a	12918682	12923843	DJ_019027-T1	Si:ch211-203k16.3	18471512	18475976
DJ_025789-T1	hypothetical protein	12941365	12942074	DJ_019028-T1	Proopiomelanocortin	18480960	18483797
DJ_025790-T1	Regulating synaptic membrane exocytosis 2	12943786	12969909	DJ_019029-T1	EFR3 homolog Bb (S. cerevisiae)	18492813	18511615
DJ_025791-T1	Regulating synaptic membrane exocytosis protein 2	12993556	12997720	DJ_019030-T1	EFR3 homolog Bb	18511938	18523982
DJ_025792-T1	Regulating synaptic membrane exocytosis 2	13000444	13029760	DJ_019031-T1	Adenylate cyclase	18533282	18541073
DJ_025793-T1	Uncharacterized protein	13032827	13037753	DJ_019032-T1	Galectin	18545032	18545831
DJ_025794-T1	hypothetical protein	13039383	13043293	DJ_019033-T1	Uncharacterized protein	18549416	18554950
DJ_025795-T1	Regulating synaptic membrane exocytosis 2b	13047020	13051710	DJ_019034-T1	Adenylate cyclase	18556732	18562363
DJ_025796-T1	Regulating synaptic membrane exocytosis 2 isoform X14	13059622	13060879	DJ_019035-T1	hypothetical protein	18590606	18597857

DJ_025797-T1	Regulating synaptic membrane exocytosis protein 2	13078258	13083510	DJ_019036-T1	hypothetical protein	18607772	18622563
DJ_025798-T1	Dendrocyte expressed seven transmembrane protein	13087695	13089800	DJ_019037-T1	Protein tyrosine phosphatase non-receptor type 14	18623065	18629326
DJ_025799-T1	Si:ch211-204a13.4 protein	13102561	13113946	DJ_019038-T1	Tyrosine-protein phosphatase non-receptor type	18629890	18654485
DJ_025800-T1	Zinc finger protein, FOG family member 2b	13159044	13184822	DJ_019039-T1	hypothetical protein	18678225	18678977
DJ_025801-T1	Zinc finger protein, FOG family member 2b	13193952	13201321	DJ_019040-T1	Potassium two pore domain channel subfamily K member 2	18679307	18704212
DJ_025802-T1	ATPase family AAA domain-containing protein 2-like	13203249	13216974	DJ_019041-T1	Si:dkey-121j17.5	18717905	18729525
DJ_025803-T1	CDC-like kinase 2a	13220845	13231197	DJ_019042-T1	cGMP-dependent protein kinase	18732505	18742151
DJ_025804-T1	SET domain, bifurcated 1a	13232771	13243940	DJ_019043-T1	Abhydrolase domain containing 1	18756210	18763447
DJ_025805-T1	Setdb1a protein	13244562	13245867	DJ_019044-T1	TLDc domain-containing protein	18767406	18773566
DJ_025806-T1	F-box and leucine-rich repeat protein 6	13276294	13281956	DJ_019045-T1	Cathepsin B	18774924	18776363
DJ_025807-T1	solute carrier family 52, riboflavin transporter, member 2	13288815	13292054	DJ_019046-T1	squalene synthase isoform X1	18776366	18791252
DJ_025808-T1	riboflavin transporter 2	13296499	13298749	DJ_019047-T1	GATA binding protein 4	18799810	18803690
DJ_025809-T1	Nuclear factor of activated T cell C1	13312899	13339736	DJ_019049-T1	hypothetical protein	18832471	18833791
DJ_025810-T1	Phospholipid-transporting ATPase	13353212	13361032	DJ_019050-T1	Nuclear receptor coactivator 7	18844270	18852627
DJ_025811-T1	ATPase class II type 9B	13361209	13383351	DJ_019051-T1	Histidine triad nucleotide-binding protein 3	18869032	18873303
DJ_025812-T1	Phospholipid-transporting ATPase	13384025	13392915	DJ_019052-T1	Syntaxin 11a	18880272	18885008
DJ_025813-T1	Spalt-like transcription factor 3b	13394392	13405169	DJ_019053-T1	Syntaxin 11b.1	18891174	18892919
DJ_025814-T1	Limb and CNS expressed 1 like	13414203	13419133	DJ_019054-T1	Syntaxin 11	18898796	18900301
DJ_025815-T1	Uncharacterized protein	13420254	13421388	DJ_019055-T1	Monofunctional C1-tetrahydrofolate synthase, mitochondrial	18926001	18928398
DJ_025816-T1	tumor necrosis factor alpha-induced protein 8-like protein 2 isoform X1	13424912	13426766	DJ_019056-T1	Methylenetetrahydrofolate dehydrogenase (NADP+-dependent) 1-like	18939738	18957092
DJ_025817-T1	Sema domain, transmembrane domain (TM), and cytoplasmic domain, (Semaphorin) 6E	13547487	13558380	DJ_019057-T1	zinc finger and BTB domain-containing protein 2	19011882	19016692
DJ_025818-T1	Sema domain, transmembrane domain (TM), and cytoplasmic domain, (Semaphorin) 6E	13562355	13569793	DJ_019058-T1	DUF89 multi-domain protein	19020937	19042252
DJ_025819-T1	semaphorin-6C isoform X4	13569845	13596093	DJ_019059-T1	nesprin-1 isoform X10	19047619	19072469
DJ_025820-T1	Extracellular matrix protein 1a	13601642	13603004	DJ_019060-T1	nesprin-1 isoform X2	19074420	19087750

DJ_025821-T1	IgGFc-binding protein-like isoform X2	13608551	13613130	DJ_019061-T1	Novel protein similar to vertebrate spectrin repeat containing, nuclear envelope 1 (SYNE1)	19087808	19101102
DJ_025822-T1	IgGFc-binding protein-like	13617708	13632144	DJ_019062-T1	Spectrin repeat-containing, nuclear envelope 1b	19113125	19118753
DJ_025823-T1	Carnosine dipeptidase 2	13633723	13641313	DJ_019063-T1	Novel protein similar to vertebrate spectrin repeat containing, nuclear envelope 1 (SYNE1)	19141953	19152992
DJ_025824-T1	hypothetical protein	13723497	13727786	DJ_019064-T1	protein SERAC1 isoform X1	19162027	19174361
DJ_025825-T1	Teashirt homolog 1	13816163	13822695	DJ_019065-T1	protein SERAC1 isoform X1	19178043	19180478
DJ_025826-T1	Zinc finger protein 516	13912937	13916809	DJ_019066-T1	Papilin b, proteoglycan-like sulfated glycoprotein	19227801	19235378
DJ_025827-T1	Zinc finger protein 516	13920438	13927292	DJ_019067-T1	hypothetical protein	19239875	19241472
DJ_025828-T1	Myelin basic protein a	13956852	13964922	DJ_019068-T1	Papilin b, proteoglycan-like sulfated glycoprotein	19244104	19247377
DJ_025829-T1	Proline-rich mitotic checkpoint control factor	13969509	13974299	DJ_019069-T1	dynein regulatory complex protein 1	19259233	19269898
DJ_025830-T1	Mitochondrial ribosomal protein L24	13976223	13979098	DJ_019070-T1	protein NLRC3-like	19287920	19305017
DJ_025831-T1	Splicing factor 3b, subunit 4	13980162	13983491	DJ_019071-T1	methyltransferase-like protein 24 isoform X2	19321793	19332847
DJ_025832-T1	NAD(P)HX epimerase	13984271	13986492	DJ_019072-T1	WD_REPEATS_REGION domain-containing protein	19342004	19343470
DJ_025833-T1	chromodomain-helicase-DNA-binding protein 4	14003915	14006794	DJ_019073-T1	Cell division cycle 40 homolog (S. cerevisiae)	19346610	19359419
DJ_025834-T1	Cell division control protein 42	14020264	14027591	DJ_019074-T1	wiskott-Aldrich syndrome protein family member 1 isoform X1	19409207	19427940
DJ_025835-T1	ENOG	14028934	14043165	DJ_019075-T1	wiskott-Aldrich syndrome protein family member 1 isoform X1	19427983	19429897
DJ_025836-T1	Triosephosphate isomerase	14045392	14048826	DJ_019077-T1	SAC domain-containing protein	19466310	19479946
DJ_025837-T1	Guanine nucleotide-binding protein (G protein), beta polypeptide 3b	14051697	14057284	DJ_019078-T1	FIG4 phosphoinositide 5-phosphatase	19491889	19506349
DJ_025838-T1	C-type lectin domain family 4 member D	14065969	14073363	DJ_019079-T1	Polyphosphoinositide phosphatase	19506645	19526833
DJ_025839-T1	Uncharacterized protein	14087571	14096459	DJ_019080-T1	adenylate kinase 9 isoform X2	19527802	19542728
DJ_025840-T1	Carcinoembryonic antigen-related cell adhesion molecule 1-like protein	14123605	14135045	DJ_019081-T1	adenylate kinase 9 isoform X1	19542855	19543994
DJ_025841-T1	Target of Myb protein 1	14154368	14165441	DJ_019082-T1	adenylate kinase 9 isoform X2	19546696	19557632
DJ_025842-T1	uncharacterized protein LOC106511497	14191059	14195152	DJ_019083-T1	Peptidyl-prolyl cis-trans isomerase	19558117	19560736
DJ_025843-T1	heme oxygenase-like	14199991	14203584	DJ_019084-T1	CD164 molecule	19568292	19570528
DJ_025844-T1	hypothetical protein	14235916	14239051	DJ_019085-T1	Centrosomal protein 57,-like 1	19579722	19588798

DJ_025845-T1	Uncharacterized protein	14261117	14266607	DJ_019086-T1	Peptidyl-tRNA hydrolase domain-containing 1	19590439	19590947
DJ_025846-T1	BHLH domain-containing protein	14274151	14278276	DJ_019087-T1	Uncharacterized protein	19593032	19593727
DJ_025848-T1	GATA-type zinc finger protein 1 isoform X2	14334680	14337456	DJ_019088-T1	Uncharacterized protein	19599332	19600032
DJ_025849-T1	Kelch-like ECH-associated 1	14462660	14464787	DJ_019089-T1	5-hydroxytryptamine (serotonin) receptor 3A	19602673	19610176
DJ_025850-T1	Volume-regulated anion channel subunit LRRC8E-like protein	14466748	14472181	DJ_019090-T1	Protein ripply2	19621371	19622136
DJ_025851-T1	ANK_REP_REGION domain-containing protein	14478168	14479160	DJ_019091-T1	UTP--glucose-1-phosphate uridylyltransferase	19623361	19629168
DJ_025852-T1	MHD domain-containing protein	14480709	14487490	DJ_019092-T1	Uncharacterized protein	19640512	19645022
DJ_025853-T1	Low density lipoprotein receptor	14499043	14512771	DJ_019093-T1	AIG1-type G domain-containing protein	19658349	19681697
DJ_025854-T1	SWI/SNF related, matrix associated, actin dependent regulator of chromatin, subfamily a, member 4	14535896	14559657	DJ_019094-T1	Uncharacterized protein	19742747	19748859
DJ_025855-T1	Meiosis regulator and mRNA stability factor 1	14563331	14592736	DJ_019095-T1	Utrophin	19750678	19769886
DJ_025856-T1	BMERB domain containing 1	14618344	14624988	DJ_019096-T1	Utrophin	19782642	19794048
DJ_025857-T1	BMERB domain containing 1	14653546	14658817	DJ_019097-T1	hypothetical protein	19831557	19832470
DJ_025858-T1	protein N-terminal asparagine amidohydrolase-like	14698716	14711986	DJ_019098-T1	Uncharacterized protein	19832833	19842729
DJ_025859-T1	DEAD-box helicase 17	14712761	14720214	DJ_019099-T1	Utrophin	19858663	19885262
DJ_025860-T1	ADP-ribosylation factor-binding protein GGA1	14722981	14748394	DJ_019100-T1	Uncharacterized protein	19887968	19897413
DJ_025861-T1	MICAL-like 1a	14749716	14765214	DJ_019108-T1	F-box protein 40	20053889	20056138
DJ_025862-T1	MICAL-like 1a	14770698	14772287	DJ_019109-T1	Metabotropic glutamate receptor 1a	20077099	20095195
DJ_025863-T1	Zgc:152881 protein	14792109	14794652	DJ_019110-T1	Metabotropic glutamate receptor 1a	20095230	20102695
DJ_025864-T1	Protein phosphatase 1 regulatory subunit	14795733	14825775	DJ_019111-T1	RAB32a, member RAS oncogene family	20104413	20113982
DJ_025865-T1	Proteasome 26S subunit, non-ATPase 12	14826478	14838564	DJ_019112-T1	ras-related protein Rab-32	20119571	20126256
DJ_025866-T1	26S proteasome non-ATPase regulatory subunit 12	14843688	14844511	DJ_019113-T1	androglobin	20129876	20133540
DJ_025868-T1	Tau tubulin kinase 1a	14894527	14896087	DJ_019114-T1	Androglobin	20136140	20136579
DJ_025869-T1	Tau-tubulin kinase 1	14901201	14907330	DJ_019121-T1	Androglobin	20163718	20186922
DJ_025870-T1	Tau tubulin kinase 1a	14944329	14951309	DJ_019122-T1	Syntaxin-binding protein 5a (tomosyn)	20206525	20209989
DJ_025871-T1	Putative tau-tubulin kinase 1	14977905	14988396	DJ_019124-T1	Syntaxin-binding protein 5a (tomosyn)	20301441	20346942

DJ_025873-T1	Nuclease HARBI1	15033148	15038672	DJ_019125-T1	SAM and SH3 domain containing 1a	20603446	20628217
DJ_025874-T1	Neural cell adhesion molecule L1 isoform X1	15042963	15044465	DJ_019126-T1	hypothetical protein	20635520	20636322
DJ_025876-T1	Adhesion G protein-coupled receptor B1b	15244267	15263958	DJ_019128-T1	hypothetical protein	20662612	20664550
DJ_025877-T1	Small ArfGAP2	15307832	15317601	DJ_019129-T1	Opsin 8, group member b	20706131	20710405
DJ_025878-T1	Regulating synaptic membrane exocytosis 3	15330218	15334663	DJ_019130-T1	Mut protein	20719605	20759086
DJ_025879-T1	Regulating synaptic membrane exocytosis protein 3	15367513	15372015	DJ_019131-T1	Heat shock protein 90, alpha (cytosolic), class A member 1, tandem duplicate 2	20760599	20761800
DJ_025880-T1	Glutathione S-transferase	15391839	15396734	DJ_019132-T1	HSP90	20763529	20765394
DJ_025881-T1	glutathione S-transferase theta-2B	15397691	15400658	DJ_019133-T1	Peptidylprolyl isomerase	20767779	20768633
DJ_025882-T1	Uncharacterized protein	15413360	15448420	DJ_019134-T1	WD repeat and coiled-coil-containing protein	20786107	20791070
DJ_025883-T1	Formin homology 2 domain containing 3a	15450423	15456561	DJ_019135-T1	Major facilitator superfamily domain-containing 2B	20792999	20820525
DJ_025884-T1	hypothetical protein	15492516	15503666	DJ_019136-T1	UBX domain protein 2A	20824193	20828872
DJ_025885-T1	Formin homology 2 domain containing 3	15508330	15510605	DJ_019137-T1	Spermatogenesis-defective protein 39-like	20840336	20854787
DJ_025886-T1	Transporter	15552140	15555929	DJ_019138-T1	serine/threonine-protein kinase MRCK beta-like isoform X2	20860132	20860618
DJ_025887-T1	Transporter	15562140	15564781	DJ_019139-T1	Serine/threonine-protein kinase MRCK beta	20862712	20898478
DJ_025888-T1	Transporter	15567381	15581533	DJ_019140-T1	Serine/threonine-protein kinase MRCK beta	20900974	20920707
DJ_025889-T1	Transporter	15592911	15608858	DJ_019141-T1	CDC42-binding protein kinase beta (DMPK-like)	20936184	20945512
DJ_025890-T1	Transporter	15622221	15627659	DJ_019142-T1	LBH-like protein	20975607	20979504
DJ_025891-T1	Telomerase catalytic subunit	15629170	15639755	DJ_019143-T1	Tumor necrosis factor, alpha-induced protein 2a	21025876	21036284
DJ_025892-T1	Nucleoporin 42	15640581	15643626	DJ_019144-T1	Ectonucleotide pyrophosphatase/phosphodiesterase family member 5	21055972	21060766
DJ_025893-T1	rap guanine nucleotide exchange factor 5	15681716	15708570	DJ_019145-T1	Protein FAM167A	21064143	21068869
DJ_025894-T1	Rap guanine nucleotide exchange factor 5	15721504	15722452	DJ_019146-T1	cerebral cavernous malformations 2 protein isoform X1	21081785	21093980
DJ_025895-T1	rap guanine nucleotide exchange factor 5	15732761	15746682	DJ_019147-T1	XK-related protein	21101897	21107332
DJ_025896-T1	Interleukin 6 (interferon, beta 2)	15771326	15772605	DJ_019148-T1	Myotubularin phosphatase domain-containing protein	21173229	21189752
DJ_025897-T1	Uncharacterized protein	15789583	15810235	DJ_019149-T1	L-threonine dehydrogenase	21195650	21200854

DJ_025898-T1	uncharacterized protein LOC108256777	15829044	15831431	DJ_019150-T1	PIN2/TERF1-interacting telomerase inhibitor 1	21205243	21215927
DJ_025899-T1	cell division cycle-associated 7-like protein	15837048	15844255	DJ_019151-T1	hypothetical protein	21219181	21220297
DJ_025900-T1	Sp4 transcription factor	15851440	15864406	DJ_019152-T1	SRY-box transcription factor 7	21242831	21245650
DJ_025901-T1	Transcription factor Sp8	15921631	15923428	DJ_019153-T1	hypothetical protein	21245866	21248918
DJ_025902-T1	Integrin beta	15928810	15933135	DJ_019154-T1	Uncharacterized protein	21252991	21264087
DJ_025903-T1	Integrin beta	15933567	15953699	DJ_019155-T1	Uncharacterized protein	21266606	21281144
DJ_025904-T1	Uncharacterized protein	16002161	16009932	DJ_019156-T1	Uncharacterized protein	21284557	21292485
DJ_025905-T1	DNA-directed RNA polymerase I subunit RPA43	16015123	16019289	DJ_019157-T1	Uncharacterized protein	21294797	21296330
DJ_025906-T1	HDAC4_Gln domain-containing protein	16105027	16130945	DJ_019158-T1	Uncharacterized protein	21296426	21300514
DJ_025907-T1	hypothetical protein	16143179	16152666	DJ_019159-T1	Rp1 like 1b	21310758	21312669
DJ_025908-T1	sorting nexin-13 isoform X1	16155383	16191548	DJ_019160-T1	Tyrosine-protein kinase	21350018	21362695
DJ_025909-T1	hypothetical protein	16208359	16209464	DJ_019161-T1	uncharacterized protein LOC106581842	21376275	21376988
DJ_025910-T1	Serine/threonine-protein phosphatase 6 regulatory ankyrin repeat subunit A	16219189	16222396	DJ_019162-T1	Fibronectin type III domain containing 4a	21389944	21426216
DJ_025911-T1	serine/threonine-protein phosphatase 6 regulatory ankyrin repeat subunit A	16222833	16256199	DJ_019163-T1	hypothetical protein	21436689	21438229
DJ_025912-T1	Biotinidase	16258043	16259988	DJ_019164-T1	Si:dkey-71h2.2	21450899	21478598
DJ_025913-T1	SH3-domain binding protein 5a (BTK-associated)	16262747	16281277	DJ_019165-T1	RBR-type E3 ubiquitin transferase	21512197	21532308
DJ_025914-T1	Maturin neural progenitor differentiation regulator protein homolog	16284605	16290157	DJ_019166-T1	Zinc finger protein 513a	21539524	21549214
DJ_025915-T1	gamma-glutamylcyclotransferase-like isoform X1	16311671	16313799	DJ_019167-T1	Sorting nexin-17	21556238	21598280
DJ_025916-T1	Guanosine 5-monophosphate oxidoreductase	16320845	16328908	DJ_019168-T1	Eukaryotic translation initiation factor 2B, subunit 4 delta	21606641	21615425
DJ_025917-T1	Thyroid hormone receptor beta B	16342629	16351394	DJ_019169-T1	Nucleoside diphosphate kinase 6	21625720	21629113
DJ_025918-T1	Thyroid hormone receptor beta 2	16351469	16357079	DJ_019170-T1	BAALC binder of MAP3K1 and KLF4 b	21630469	21633425
DJ_025922-T1	hypothetical protein	16542439	16542885	DJ_019171-T1	Si:ch211-182e10.4	21640426	21646146
DJ_025923-T1	hypothetical protein	16566747	16567406	DJ_019173-T1	Uncharacterized protein	21735713	21738533
DJ_025924-T1	Retinoic acid receptor beta	16692775	16693656	DJ_019174-T1	Cholinergic receptor, nicotinic, alpha 2b (neuronal)	21745484	21751699
DJ_025925-T1	Retinoic acid receptor beta variant 1	16746135	16772489	DJ_019175-T1	Cholinergic receptor, nicotinic, alpha 2 (Neuronal)	21754505	21756478
DJ_025926-T1	hypothetical protein	16778706	16782892	DJ_019176-T1	Protein tyrosine kinase 2 beta, b	21758530	21778791

DJ_025927-T1	DNA topoisomerase 2	16792450	16825803	DJ_019177-T1	Potassium voltage-gated channel, subfamily H (eag-related), member 5a	21815353	21819941
DJ_025928-T1	PAW domain-containing protein	16831037	16842689	DJ_019178-T1	Potassium voltage-gated channel, subfamily H (eag-related), member 5a	21839029	21851472
DJ_025929-T1	3-oxoacyl-[acyl-carrier-protein] synthase	16843677	16847922	DJ_019179-T1	Potassium voltage-gated channel, subfamily H (eag-related), member 5a	21878107	21891079
DJ_025930-T1	Leucine rich repeat containing 3B	16911789	16913784	DJ_019180-T1	Potassium voltage-gated channel subfamily H member 5	21913650	21931317
DJ_025932-T1	hypothetical protein	16967379	16968115	DJ_019181-T1	Ras homolog family member J	21969325	21970910
DJ_025933-T1	hypothetical protein	16976729	16981484	DJ_019182-T1	Serine/threonine protein phosphatase 2A regulatory subunit	21981259	21993681
DJ_025934-T1	Sept7a protein	16997200	17002666	DJ_019183-T1	sphingosine-1-phosphate phosphatase 1-like	22005007	22018564
DJ_025935-T1	hypothetical protein	17014218	17016236	DJ_019184-T1	snRNA-activating protein complex subunit 1-like	22029835	22032740
DJ_025936-T1	Endonuclease/exonuclease/phosphatase family domain-containing protein 1	17079276	17081629	DJ_019185-T1	HIF1a	22035490	22036720
DJ_025937-T1	KIAA0895	17095740	17103939	DJ_019186-T1	Protein kinase C	22065694	22068379
DJ_025938-T1	Anln-like protein	17128022	17152424	DJ_019187-T1	Protein kinase C	22072656	22094168
DJ_025939-T1	hypothetical protein	17153582	17155416	DJ_019188-T1	Homeobox domain-containing protein	22159059	22167438
DJ_025940-T1	coiled-coil domain-containing protein 126	17160447	17162308	DJ_019189-T1	Sine oculis homeobox homolog 1b	22179391	22181313
DJ_025941-T1	hypothetical protein	17163668	17165871	DJ_019190-T1	Sine oculis-related homeobox 6b	22221473	22223528
DJ_025942-T1	RRM domain-containing protein	17166415	17170817	DJ_019191-T1	PPM1A	22288428	22300166
DJ_025943-T1	Igf2bp3 protein	17190127	17191371	DJ_019192-T1	Uncharacterized protein	22358140	22359208
DJ_025944-T1	Igf2bp3 protein	17213770	17222755	DJ_019193-T1	Connective tissue growth factor a	22556593	22558605
DJ_025945-T1	RRM domain-containing protein	17231512	17241498	DJ_019194-T1	mitogen-activated protein kinase kinase kinase 5	22564632	22615340
DJ_025946-T1	Glycoprotein (transmembrane) nmb	17245542	17254444	DJ_019195-T1	Low density lipoprotein receptor-related protein 11	22632031	22638897
DJ_025947-T1	Glycoprotein (transmembrane) nmb	17269883	17287292	DJ_019196-T1	Protein-L-isoaspartate O-methyltransferase	22642574	22651408
DJ_025948-T1	hypothetical protein	17482796	17487003	DJ_019197-T1	Large tumor suppressor kinase 1	22659155	22674141
DJ_025949-T1	hypothetical protein	17495296	17499144	DJ_019198-T1	Zgc:101840	22679165	22684537
DJ_025950-T1	Trafficking protein particle complex subunit	17850093	17852038	DJ_019199-T1	Retinol dehydrogenase 14	22686350	22687707
DJ_025951-T1	Trafficking protein particle complex subunit	17852759	17862234	DJ_019200-T1	Si:dkey-174m14.3	22691153	22704365
DJ_025952-T1	Angiopoietin-2-like protein	17906156	17909888	DJ_019201-T1	Cytosolic 5-nucleotidase 1A	22735507	22739529
DJ_025953-T1	hypothetical protein	17922998	17928641	DJ_019202-T1	Synaptotagmin XIVb	22747333	22756363

DJ_025954-T1	Angiopoietin 2b	17947079	17960005	DJ_019203-T1	Potassium channel, subfamily K, member 5b	22762875	22769122
DJ_025955-T1	CUB and Sushi multiple domains 2	18277142	18293398	DJ_019204-T1	BZIP domain-containing protein	22776703	22778806
DJ_025956-T1	hypothetical protein	18308720	18313671	DJ_019205-T1	hypothetical protein	22978767	22981149
DJ_025957-T1	CUB and sushi domain-containing protein 2	18317933	18329873	DJ_019206-T1	Leucine rich repeat and fibronectin type III domain containing 2b	23106090	23109005
DJ_025958-T1	CSMD2	18347404	18359568	DJ_019207-T1	transmembrane protein 181 isoform X2	23137934	23145782
DJ_025959-T1	CUB and Sushi multiple domains 2	18361451	18377757	DJ_019208-T1	Novel protein similar to vertebrate tubby like protein 4 (TULP4)	23149535	23181384
DJ_025960-T1	CUB and sushi domain-containing protein 2	18391074	18401744	DJ_019209-T1	Si:ch211-51e12.7	23193480	23196894
DJ_025961-T1	CUB and sushi domain-containing protein 2	18402277	18437019	DJ_019210-T1	flap endonuclease GEN homolog 1	23201701	23209523
DJ_025962-T1	CUB and Sushi multiple domains 2	18458003	18463981	DJ_019211-T1	Dual specificity phosphatase 10	23229109	23237980
DJ_025963-T1	Spermidine acetyltransferase KAT2B	18502600	18506577	DJ_019213-T1	TATA box binding protein (TBP)-associated factor, RNA polymerase I, A	23281379	23287954
DJ_025964-T1	Spermidine acetyltransferase KAT2B	18515097	18518993	DJ_019214-T1	MIA SH3 domain ER export factor 3	23288765	23303909
DJ_025965-T1	Histone acetyltransferase	18524427	18529223	DJ_019215-T1	BRO1 domain-containing protein	23314114	23321182
DJ_025967-T1	PEHE domain-containing protein	18538031	18552623	DJ_019218-T1	Ammonium transporter Rh type A	23484783	23498773
DJ_025968-T1	SWI/SNF related, matrix associated, actin dependent regulator of chromatin subfamily c member 1	18553320	18554926	DJ_019219-T1	Cysteine-rich venom protein	23517176	23531180
DJ_025969-T1	SWI/SNF related, matrix associated, actin dependent regulator of chromatin, subfamily c, member 1a	18555079	18559588	DJ_019220-T1	MACO1	23534143	23537720
DJ_025970-T1	Chondroitin sulfate proteoglycan 5	18626550	18639513	DJ_019221-T1	Si:dkey-12h9.6	23537920	23543058
DJ_025971-T1	Putative tyrosine-protein phosphatase non-receptor type 23	18652074	18668859	DJ_019222-T1	Calpain 3	23563321	23570462
DJ_025972-T1	Si:dkeyp-114f9.2 protein	18676331	18678616	DJ_019223-T1	Calpain 3a, (p94)	23571511	23573709
DJ_025973-T1	Mitochondrial ribosomal protein L3	18702139	18710322	DJ_019224-T1	Calpain 3b	23573973	23593574
DJ_025974-T1	Uncharacterized protein	18714992	18716354	DJ_019225-T1	Uncharacterized protein	23594954	23600092
DJ_025975-T1	Copine IVb	18718770	18736192	DJ_019226-T1	Churchill protein	23600785	23601544
DJ_025976-T1	Dynein light intermediate chain	18758597	18780540	DJ_019227-T1	Tau tubulin kinase 2b	23603755	23615244
DJ_025977-T1	MARVEL domain-containing protein	18796567	18803480	DJ_019228-T1	ELM2 and Myb/SANT-like domain containing 1a	23619175	23633857
DJ_025978-T1	hypothetical protein	18816353	18819603	DJ_019229-T1	hypothetical protein	23646264	23647455
DJ_025979-T1	Ras-related and estrogen-regulated growth inhibitor-like protein	18863161	18864218	DJ_019230-T1	Zona pellucida protein AX 1	23665483	23679365

DJ_025980-T1	Zgc:153402 protein	18934149	18936329	DJ_019231-T1	matrilin-3-like isoform X1	23687620	23695328
DJ_025981-T1	peroxisomal membrane protein 11B isoform X2	18936421	18937514	DJ_019232-T1	Lysosomal-associated transmembrane protein 4A	23697493	23710438
DJ_025982-T1	Si:ch211-93a2.3	18941562	18952030	DJ_019233-T1	Syndecan domain-containing protein	23712350	23717095
DJ_025983-T1	Synaptotagmin XI	18964873	18974309	DJ_019234-T1	Apolipoprotein Bb.2	23720873	23742355
DJ_025984-T1	SMAD family member	19008239	19010599	DJ_019235-T1	apolipoprotein B-100-like	23757485	23774024
DJ_025985-T1	SMAD family member	19028552	19034812	DJ_019236-T1	YHR209W isoform X1	23794406	23797169
DJ_025986-T1	Biogenesis of lysosome-related organelles complex 1 subunit 7	19043531	19044468	DJ_019237-T1	membrane progestin receptor beta	23800637	23809372
DJ_025987-T1	Chromatin target of PRMT1a	19045537	19052441	DJ_019238-T1	Zinc finger protein 395b	23820658	23827667
DJ_025989-T1	Interleukin enhancer binding factor 2	19054797	19061926	DJ_019239-T1	Prepronociceptin b	23829837	23833110
DJ_025990-T1	hypothetical protein	19062543	19063009	DJ_019240-T1	Si:ch211-63o20.7	23845138	23849848
DJ_025991-T1	hypothetical protein	19066653	19070401	DJ_019241-T1	Uncharacterized protein	23853513	23859059
DJ_025992-T1	Discs, large (Drosophila) homolog-associated protein 3	19076394	19099031	DJ_019242-T1	Elongator complex protein 3	23860541	23879818
DJ_025993-T1	Disks large-associated protein 3	19111871	19119064	DJ_019243-T1	kinesin-like protein KIF13B isoform X2	23881326	23887449
DJ_025994-T1	hypothetical protein	19174042	19179431	DJ_019244-T1	Kinesin family member 13Bb	23899902	23906218
DJ_025995-T1	hypothetical protein	19237745	19240213	DJ_019245-T1	Heat shock protein 90	23922187	23928511
DJ_025996-T1	Gap junction protein	19345980	19347543	DJ_019246-T1	RNA binding motif protein 25b	23933336	23941829
DJ_025997-T1	Peflin	19375997	19389279	DJ_019247-T1	RNA-binding protein 25 isoform X1	23947249	23949472
DJ_025998-T1	hypothetical protein	19399454	19408919	DJ_019248-T1	Eukaryotic initiation factor 2-alpha	23950903	23954818
DJ_026001-T1	Potassium voltage-gated channel, subfamily G, member 2	19708642	19714302	DJ_019249-T1	Vacuolar ATP synthase subunit D	23955964	23959044
DJ_026002-T1	Potassium voltage-gated channel, subfamily G, member 2	19722847	19726001	DJ_019250-T1	MAGUK p55 subfamily member 5	23961230	23980975
DJ_026003-T1	PQ loop repeat containing 1	19734918	19768431	DJ_019251-T1	Domain E	23987325	23993180
DJ_026004-T1	hypothetical protein	19775811	19776980	DJ_019252-T1	Domain E	23994689	23997774
DJ_026005-T1	Uncharacterized protein	19779734	19790393	DJ_019253-T1	Domain E	24000333	24004508
DJ_026006-T1	IgGFc-binding protein-like	19798538	19811607	DJ_019254-T1	MoCF_biosynth domain-containing protein	24035056	24047737
DJ_026007-T1	tRNA-dihydrouridine synthase 3	19812363	19813841	DJ_019255-T1	hypothetical protein	24050578	24052895
DJ_026008-T1	tRNA-dihydrouridine synthase 3	19813947	19818218	DJ_019256-T1	Alpha-(1,6)-fucosyltransferase	24180858	24222940
DJ_026009-T1	A-kinase anchoring protein 9	19824152	19881587	DJ_019257-T1	hypothetical protein	24270132	24271956
DJ_026011-T1	PACT_coil_coil domain-containing protein	19893732	19912957	DJ_019259-T1	sodium/potassium/calcium exchanger 4 isoform X1	24510816	24533622

DJ_026012-T1	Cytochrome P450 family 51 subfamily A member 1	19940143	19945784	DJ_019260-T1	Cleavage and polyadenylation specificity factor 100 kDa subunit	24581474	24584850
DJ_026013-T1	Leucine rich repeats and death domain containing 1	19946819	19949887	DJ_019261-T1	Cleavage and polyadenylation specificity factor 100 kDa subunit	24585062	24593434
DJ_026014-T1	krev interaction trapped protein 1	19951550	19967291	DJ_019262-T1	Cleavage and polyadenylation specificity factor 100 kDa subunit	24593661	24596993
DJ_026015-T1	RBR-type E3 ubiquitin transferase	19976810	19986682	DJ_019263-T1	Bifunctional lysine-specific demethylase and histidyl-hydroxylase	24599168	24604555
DJ_026016-T1	RBR-type E3 ubiquitin transferase	19988399	20023542	DJ_019264-T1	exostosin-like 3	24629194	24641601
DJ_026017-T1	CBFD_NFYB_HMF domain-containing protein	20041950	20062311	DJ_019265-T1	Zgc:154012	24645715	24664487
DJ_026018-T1	Potassium voltage-gated channel subfamily Q member 4	20131174	20135587	DJ_019266-T1	Homeobox containing 1	24670983	24681931
DJ_026019-T1	Potassium voltage-gated channel subfamily Q member 4	20136077	20158089	DJ_019267-T1	Homeobox containing 1	24681964	24685193
DJ_026020-T1	protein JTB isoform X1	20165447	20167352	DJ_019268-T1	Non-specific serine/threonine protein kinase	24724805	24742148
DJ_026021-T1	Cocaine- and amphetamine-regulated transcript 4	20172407	20173163	DJ_019269-T1	Centrosomal protein 170B	24750023	24766830
DJ_026022-T1	Uncharacterized protein	20179596	20190968	DJ_019270-T1	Centrosomal protein 170B	24766881	24773711
DJ_026023-T1	C-type lectin domain-containing protein	20234168	20235985	DJ_019271-T1	Xgb protein	24778179	24790665
DJ_026024-T1	Pleckstrin homology domain containing, family O member 1a	20260053	20268689	DJ_019272-T1	Pleckstrin homology and RhoGEF domain containing G3	24798999	24816195
DJ_026025-T1	Vacuolar protein sorting 45 homolog	20289136	20301312	DJ_019273-T1	RET2	24870212	24873600
DJ_026026-T1	Ring finger protein 1	20344062	20347286	DJ_019274-T1	Retinaldehyde binding protein 1	24874965	24876505
DJ_026027-T1	Ribosomal protein S18	20348228	20351209	DJ_019275-T1	kunitz-type protease inhibitor 1-like isoform X1	24877585	24888452
DJ_026028-T1	vacuolar protein sorting-associated protein 52 homolog	20351521	20355136	DJ_019276-T1	Kinase D-interacting substrate of 220 kDa B	24899279	24915481
DJ_026029-T1	DUF4806 domain-containing protein	20366053	20369126	DJ_019277-T1	Kinase D-interacting substrate 220b	24917433	24925324
DJ_026031-T1	Uncharacterized protein	20425862	20426768	DJ_019278-T1	DNA-binding protein inhibitor ID-2	24927380	24928029
DJ_026032-T1	Tapasin	20451412	20453441	DJ_019279-T1	G-patch domain containing 2	24970824	24979171
DJ_026033-T1	Major histocompatibility complex class I-related protein	20453982	20464355	DJ_019280-T1	hypothetical protein	24980761	24981941
DJ_026034-T1	Proteasome subunit beta	20475652	20477670	DJ_019281-T1	LNS2 domain-containing protein	24994413	25016552
DJ_026035-T1	Proteasome subunit beta	20478232	20482053	DJ_019282-T1	Myoxvirus resistance protein B	25020224	25025010
DJ_026036-T1	Proteasome subunit beta	20484018	20486114	DJ_019283-T1	Myoxvirus resistance protein B	25029264	25034924
DJ_026037-T1	Proteasome subunit beta	20489479	20490927	DJ_019284-T1	Myoxvirus resistance protein B	25040905	25043104
DJ_026038-T1	Transporter 2 ATP binding cassette	20493703	20499059	DJ_019285-T1	PlsC domain-containing protein	25056745	25059135

DJ_026039-T1	Heterogeneous nuclear ribonucleoprotein R	20506349	20512247	DJ_019286-T1	Si:ch73-21k16.5	25059298	25065528
DJ_026040-T1	hypothetical protein	20517574	20518390	DJ_019287-T1	Family with sequence similarity 228 member A	25067903	25073332
DJ_026041-T1	J domain-containing protein	20520634	20525404	DJ_019288-T1	protein max isoform X4	25111317	25116778
DJ_026042-T1	NADH dehydrogenase [ubiquinone] 1 beta subcomplex subunit 9	20554209	20555921	DJ_019289-T1	Solute carrier family 10 member 1	25118948	25130991
DJ_026043-T1	MTSS I-BAR domain containing 1	20558321	20561177	DJ_019290-T1	Splicing factor, arginine/serine-rich 5b	25131962	25136832
DJ_026044-T1	MTSS I-BAR domain containing 1	20561260	20574720	DJ_019291-T1	hypothetical protein	25172492	25173533
DJ_026045-T1	hypothetical protein	20578436	20580866	DJ_019292-T1	Acyl-coenzyme A thioesterase 2, mitochondrial	25186261	25187692
DJ_026046-T1	MTSS I-BAR domain containing 1	20587590	20603002	DJ_019293-T1	Acyl-coenzyme A thioesterase 2, mitochondrial	25194927	25196577
DJ_026047-T1	Secretagogin, EF-hand calcium binding protein	20613253	20615903	DJ_019294-T1	hypothetical protein	25199193	25199605
DJ_026048-T1	hypothetical protein	20618324	20619104	DJ_019295-T1	G protein-coupled receptor 176	25204360	25211905
DJ_026049-T1	Leucine rich repeat containing 16A	20619518	20647890	DJ_019297-T1	Thrombospondin 1b	25369730	25384398
DJ_026050-T1	Uncharacterized protein	20666011	20699454	DJ_019298-T1	hypothetical protein	25467008	25475445
DJ_026051-T1	Uncharacterized protein	20703396	20727041	DJ_019299-T1	hypothetical protein	25561108	25562005
DJ_026052-T1	DEP domain-containing mTOR-interacting protein-like	20732057	20734839	DJ_019300-T1	hypothetical protein	25587251	25587854
DJ_026053-T1	collagen alpha-1(XIV) chain isoform X1	20739818	20788355	DJ_019301-T1	Katanin_con80 domain-containing protein	25613320	25616329
DJ_026054-T1	Collagen, type XIV, alpha 1	20792541	20793317	DJ_019302-T1	DUF2012 domain-containing protein	25617815	25619632
DJ_026055-T1	collagen alpha-1(XIV) chain isoform X1	20794570	20802372	DJ_019303-T1	Apoptosis, caspase activation inhibitor	25661812	25663405
DJ_026056-T1	Uncharacterized protein	20809864	20812268	DJ_019304-T1	ryanodine receptor 3 isoform X1	25664951	25669030
DJ_026057-T1	hypothetical protein	20825491	20831527	DJ_019305-T1	Ryanodine receptor 3	25671921	25678602
DJ_026059-T1	Oxidation resistance 1b	20910534	20923252	DJ_019306-T1	Ryanodine receptor 3	25680530	25759975
DJ_026060-T1	Oxidation resistance 1	20934803	20947429	DJ_019307-T1	formin-1 isoform X1	25805885	25824540
DJ_026061-T1	Oxidation resistance 1b	20958639	20976863	DJ_019308-T1	Rho-GAP domain-containing protein	25827387	25834919
DJ_026062-T1	Coiled-coil domain containing 127a-like	20980352	20982204	DJ_019309-T1	Actin alpha 1, skeletal muscle	25836429	25839092
DJ_026063-T1	coiled-coil domain-containing protein 127	20983343	20984537	DJ_019310-T1	galactocerebrosidase-like	25845977	25854922
DJ_026064-T1	Succinate dehydrogenase [ubiquinone] flavoprotein subunit, mitochondrial	20985069	20988283	DJ_019311-T1	adenylate kinase 7-like isoform X2	25859110	25861023
DJ_026065-T1	Flavoprotein subunit of complex II	20988330	20999527	DJ_019312-T1	DNA repair protein RAD51 homolog	25862415	25865715
DJ_026066-T1	Retinoid X receptor beta-A	21002332	21019466	DJ_019313-T1	regulator of microtubule dynamics protein 3	25873508	25885711
DJ_026067-T1	hypothetical protein	21029950	21033020	DJ_019314-T1	Uncharacterized protein	25897702	25899812

DJ_026068-T1	collagen alpha-2(XI) chain isoform X1	21041815	21054716	DJ_019315-T1	SP110 nuclear body protein, tandem duplicate 1	25900027	25912175
DJ_026069-T1	Uncharacterized protein	21056551	21065454	DJ_019316-T1	Inverted formin, FH2 and WH2 domain containing	25921242	25935643
DJ_026070-T1	Collagen type XI	21065714	21081603	DJ_019317-T1	Inverted formin, FH2 and WH2 domain containing	25935757	25937287
DJ_026071-T1	Collagen, type XI, alpha 2	21081880	21087988	DJ_019318-T1	IMP--aspartate ligase 1	25942941	25953101
DJ_026072-T1	estradiol 17-beta-dehydrogenase 8	21091293	21095726	DJ_019319-T1	adenosine 3-phospho 5-phosphosulfate transporter 1 isoform X3	25968494	25974141
DJ_026073-T1	Bromodomain-containing protein 2a	21098369	21110263	DJ_019320-T1	ANK_REP_REGION domain-containing protein	25976379	25985087
DJ_026074-T1	Eukaryotic translation initiation factor 2C 4	21117669	21126964	DJ_019321-T1	transmembrane protein 151B	25994238	26001805
DJ_026075-T1	Eukaryotic translation initiation factor 2C 4	21127023	21130684	DJ_019322-T1	T-complex-associated-testis-expressed 1	26007729	26010971
DJ_026076-T1	Eukaryotic translation initiation factor 2C 4	21133055	21141450	DJ_019323-T1	RING-type E3 ubiquitin transferase RNF8	26022401	26027810
DJ_026077-T1	Nbl1_Borealin_N domain-containing protein	21153660	21161307	DJ_019324-T1	Toll-like receptor 5	26079278	26080399
DJ_026078-T1	hypothetical protein	21180456	21182495	DJ_019325-T1	protein dispatched homolog 1	26085543	26106490
DJ_026079-T1	hypothetical protein	21223298	21226307	DJ_019327-T1	BRO1 domain-containing protein	26168217	26175179
DJ_026080-T1	Follistatin like 1	21262078	21271493	DJ_019328-T1	hypothetical protein	26178604	26181732
DJ_026082-T1	hypothetical protein	21390451	21396929	DJ_019329-T1	DUF4806 domain-containing protein	26185549	26187836
DJ_026083-T1	EPH receptor A10	21408568	21411402	DJ_019330-T1	MIA SH3 domain ER export factor 3	26195495	26209812
DJ_026084-T1	EPH receptor A10	21465080	21473223	DJ_019331-T1	TATA box binding protein (TBP)-associated factor, RNA polymerase I, A	26211640	26218222
DJ_026085-T1	EPH receptor A10	21525977	21539393	DJ_019332-T1	hypothetical protein	26240264	26242203
DJ_026086-T1	Prolyl endopeptidase	21549549	21573209	DJ_019333-T1	Synaptogyrin	26314597	26327588
DJ_026087-T1	PR domain containing 1b, with ZNF domain	21602904	21615579	DJ_019334-T1	unconventional myosin-XV-like	26381722	26397315
DJ_026088-T1	Squalene epoxidase	21624542	21630204	DJ_019336-T1	Myosin XVAa	26430907	26462414
DJ_026089-T1	Squalene epoxidase	21630233	21634134	DJ_019337-T1	Myosin XVAb	26482236	26535105
DJ_026092-T1	uncharacterized protein LOC106570042 isoform X2	21649498	21667531	DJ_019338-T1	Alpha-ketoglutarate-dependent dioxygenase alkB homolog 5	26562548	26572488
DJ_026093-T1	hypothetical protein	21676966	21678344	DJ_019339-T1	Mitochondrial elongation factor 2	26594632	26605004
DJ_026094-T1	Annexin	21681825	21689111	DJ_019340-T1	Mitochondrial dynamic protein MID51	26612254	26615953

DJ_026095-T1	mediator of RNA polymerase II transcription subunit 30-like	21708999	21710258	DJ_019341-T1	Family with sequence similarity 83 member G	26673076	26702552
DJ_026096-T1	exostosin-1	21711718	21723600	DJ_019342-T1	Si:dkeyp-9d4.3	26857203	26861396
DJ_026097-T1	protein phosphatase 1 regulatory subunit 36 isoform X2	21806860	21808523	DJ_019343-T1	CASK interacting protein 1	26864556	26865585
DJ_026098-T1	protein phosphatase 1 regulatory subunit 36 isoform X1	21808853	21815747	DJ_019345-T1	NHS-like protein 1 isoform X4	26938425	26939695
DJ_026099-T1	MADF domain-containing protein	21818619	21819590	DJ_019346-T1	Golgi resident GCP60	26944164	26953556
DJ_026100-T1	Oxysterol-binding protein	21825820	21833779	DJ_019347-T1	Uncharacterized protein	26955138	26963040
DJ_026101-T1	Oxysterol-binding protein	21861875	21869998	DJ_019348-T1	Mix paired-like homeobox	26963154	26964806
DJ_026102-T1	Dolichyl-diphosphooligosaccharide--protein glycosyltransferase subunit STT3B	21876140	21910066	DJ_019349-T1	Protein lin-9 homolog	26966156	26970260
DJ_026103-T1	hypothetical protein	21917438	21924788	DJ_019350-T1	Protein lin-9 homolog	26970986	26986944
DJ_026104-T1	STT3 oligosaccharyltransferase complex catalytic subunit B	21940188	21963046	DJ_019351-T1	Poly [ADP-ribose] polymerase	26988272	26995348
DJ_026105-T1	Serine/threonine-protein kinase receptor	22035771	22058440	DJ_019352-T1	Poly [ADP-ribose] polymerase	26995863	26996958
DJ_026106-T1	hypothetical protein	22197053	22197500	DJ_019353-T1	Adenosine receptor A2c	27005442	27007237
DJ_026107-T1	Hes-related family bHLH transcription factor	22197686	22200052	DJ_019354-T1	Poly [ADP-ribose] polymerase	27007386	27011304
DJ_026108-T1	Stathmin	22202815	22210529	DJ_019356-T1	SET and MYND domain containing 2	27027687	27029144
DJ_026109-T1	LOC566469 protein	22244014	22252774	DJ_019357-T1	SET and MYND domain containing 2	27034792	27043988
DJ_026110-T1	Rho family-interacting cell polarization regulator 2	22323559	22325390	DJ_019359-T1	Prospero homeobox 1	27069943	27081973
DJ_026111-T1	Rho family-interacting cell polarization regulator 2	22327454	22340146	DJ_019360-T1	Prospero homeobox 1	27083992	27086299
DJ_026112-T1	RHO family interacting cell polarization regulator 2	22350371	22359623	DJ_019361-T1	Ribosomal protein S6 kinase polypeptide 1	27140326	27150266
DJ_026113-T1	Uncharacterized protein	22363753	22365936	DJ_019362-T1	Ribosomal protein S6 kinase polypeptide 1	27151893	27163637
DJ_026114-T1	geminin isoform X2	22367165	22370933	DJ_019363-T1	hypothetical protein	27175838	27179358
DJ_026115-T1	Chromosome 6 open reading frame 62	22374141	22377446	DJ_019364-T1	hypothetical protein	27182053	27185172
DJ_026116-T1	acyl-coenzyme A thioesterase 13	22378944	22379815	DJ_019365-T1	Uncharacterized protein	27188209	27189950
DJ_026117-T1	tyrosyl-DNA phosphodiesterase 2 isoform X1	22380257	22383176	DJ_019366-T1	hypothetical protein	27196204	27199743
DJ_026118-T1	Transmembrane protein 64	22398024	22414026	DJ_019367-T1	hypothetical protein	27208264	27208994
DJ_026119-T1	asparagine synthetase [glutamine-hydrolyzing]	22475436	22488540	DJ_019368-T1	Sortilin related VPS10 domain containing receptor 3	27221538	27235581
DJ_026120-T1	protachykinin-like isoform X2	22491800	22496844	DJ_019369-T1	Uncharacterized protein	27285925	27287599

DJ_026121-T1	Succinate dehydrogenase assembly factor 3, mitochondrial	22569329	22581609	DJ_019370-T1	uncharacterized protein LOC106590961	27287635	27290047
DJ_026122-T1	Uncharacterized protein	22583635	22611635	DJ_019371-T1	hypothetical protein	27295584	27297349
DJ_026123-T1	replication protein A 14 kDa subunit	22623662	22624529	DJ_019372-T1	Si:ch73-112l6.1	27297499	27299651
DJ_026124-T1	UBAP1-MVB12-associated (UMA) domain-containing 1	22625544	22643230	DJ_019373-T1	Uncharacterized protein	27300417	27306223
DJ_026125-T1	Uncharacterized protein	22645975	22647734	DJ_019374-T1	uncharacterized protein LOC106590961	27307499	27309461
DJ_026126-T1	Glucocorticoid-induced transcript 1 protein	22693503	22696606	DJ_019375-T1	Uncharacterized protein	27309793	27319559
DJ_026127-T1	uncharacterized protein LOC108278005	22902229	22905117	DJ_019376-T1	Si:ch73-112l6.1	27365828	27369823
DJ_026128-T1	hypothetical protein	23074287	23076044	DJ_019377-T1	PDZ-binding kinase	27371861	27374780
DJ_026129-T1	triple functional domain protein isoform X6	23093274	23111686	DJ_019378-T1	Establishment of cohesion 1 homolog 2	27376474	27387208
DJ_026130-T1	triple functional domain protein isoform X6	23113388	23122117	DJ_019379-T1	Scavenger receptor class A, member 3	27419458	27423740
DJ_026131-T1	triple functional domain protein isoform X6	23125019	23143721				
DJ_026132-T1	Trio Rho guanine nucleotide exchange factor a	23145110	23176048				
DJ_026133-T1	Tubulointerstitial nephritis antigen like 1	23185859	23210273				
DJ_026135-T1	GATA zinc finger domain-containing 2B	23276471	23279861				
DJ_026136-T1	GATA zinc finger domain-containing 2B	23280476	23291223				
DJ_026137-T1	Gonadotropin-releasing hormone receptor 1	23347608	23352176				
DJ_026138-T1	Si:ch211-57n23.4	23393746	23394663				
DJ_026139-T1	Si:ch211-57n23.4	23397870	23413377				
DJ_026140-T1	Brain protein 16	23450431	23455404				
DJ_026141-T1	testis-specific serine/threonine-protein kinase 5-like isoform X1	23455744	23460780				
DJ_026142-T1	Block of proliferation 1 protein	23483576	23505626				
DJ_026143-T1	Block of proliferation 1 protein	23505650	23508287				
DJ_026144-T1	basic helix-loop-helix transcription factor scleraxis	23524770	23526113				
DJ_026145-T1	hypothetical protein	23533272	23536235				
DJ_026146-T1	Block of proliferation 1 protein	23542080	23551845				
DJ_026147-T1	HSF_DOMAIN domain-containing protein	23552556	23568464				
DJ_026148-T1	Uncharacterized protein	23570872	23579707				

DJ_026149-T1	hypothetical protein	23593820	23596755
DJ_026150-T1	Si:ch211-133n4.6	23609795	23621078
DJ_026151-T1	casein kinase II subunit alpha-like	23621772	23634508
DJ_026153-T1	Protein tyrosine phosphatase 4A3	23649787	23653459
DJ_026154-T1	Zgc:113424	23673754	23674829
DJ_026156-T1	N-myc downstream regulated gene 1a	23702800	23711191
DJ_026157-T1	wnt1-inducible-signaling pathway protein 1 precursor	23714000	23721761
DJ_026158-T1	Src-like-adaptor 1	23746456	23751322
DJ_026160-T1	Catenin (cadherin-associated protein), alpha-like 1	23812669	23850325
DJ_026161-T1	elongator complex protein 2 isoform X3	23854430	23865201
DJ_026162-T1	elongator complex protein 2	23885263	23893153
DJ_026164-T1	Gamma-aminobutyric acid (GABA) B receptor, 2	23926282	23934251
DJ_026166-T1	Gamma-aminobutyric acid (GABA) B receptor, 2	24002750	24026362
DJ_026167-T1	Gamma-aminobutyric acid (GABA) B receptor, 2	24041681	24081696
DJ_026168-T1	hypothetical protein	24135743	24137220
DJ_026169-T1	Uncharacterized protein	24247885	24248315
DJ_026170-T1	hypothetical protein	24262388	24262943
DJ_026171-T1	uncharacterized protein LOC106570452	24277130	24279237
DJ_026172-T1	Protein-UDP acetylgalactosaminyltransferase	24285292	24306356
DJ_026173-T1	Protein-UDP acetylgalactosaminyltransferase	24311681	24330630
DJ_026174-T1	UPF0711 protein C18orf21 homolog	24362321	24380694
DJ_026175-T1	Retinitis pigmentosa 9 (Autosomal dominant)	24382282	24386978
DJ_026176-T1	hypothetical protein	24416403	24417497
DJ_026177-T1	Engulfment and cell motility protein 1	24442845	24452603
DJ_026178-T1	Engulfment and cell motility 1	24460652	24486315
DJ_026179-T1	Engulfment and cell motility protein 1	24498080	24508136
DJ_026180-T1	hypothetical protein	24508515	24509219
DJ_026181-T1	Engulfment and cell motility 1	24530109	24543650

DJ_026182-T1	peroxisome biogenesis factor 1 isoform X2	24546872	24575432
DJ_026183-T1	EF-hand calcium-binding domain-containing protein 1	24577513	24579789
DJ_026184-T1	Cyclin-dependent kinase 6	24613313	24632196
DJ_026185-T1	Cyclin-dependent kinase 6	24659494	24661339
DJ_026186-T1	Family with sequence similarity 133 member B	24667925	24668926
DJ_026187-T1	hypothetical protein	24670315	24672485
DJ_026188-T1	HEPACAM family member 2-like isoform X3	24677166	24687605
DJ_026189-T1	Coiled-coil domain containing 132	24690948	24728502
DJ_026190-T1	syndetin isoform X2	24750549	24765992
DJ_026191-T1	syndetin isoform X3	24781583	24812535
DJ_026192-T1	hypothetical protein	24837518	24839701
DJ_026193-T1	VPS50 EARP/GARPII complex subunit	24840016	24856504
DJ_026194-T1	VPS50 EARP/GARPII complex subunit	24864693	24869857
DJ_026195-T1	Calcitonin receptor	24906196	24942518
DJ_026196-T1	tissue factor pathway inhibitor 2 isoform X2	24984253	24989075
DJ_026197-T1	Putative acyl-CoA dehydrogenase 6	24989682	25005044
DJ_026198-T1	Putative acyl-CoA dehydrogenase 6	25005618	25009325
DJ_026200-T1	hypothetical protein	25088026	25100406
DJ_026201-T1	collagen alpha-2(I) chain isoform X2	25102351	25111927
DJ_026202-T1	Cas1_AcylT domain-containing protein	25117883	25120806
DJ_026203-T1	N-acetylneuraminate 9-O-acetyltransferase	25121184	25130785
DJ_026204-T1	Sodium channel beta 1 subunit	25144905	25146096
DJ_026205-T1	DUF4806 domain-containing protein	25149117	25151952
DJ_026206-T1	zinc finger and BTB domain-containing protein 22-like isoform X2	25155991	25158894
DJ_026207-T1	Cytochrome P450 LMC2	25159509	25168668
DJ_026208-T1	Synaptic Ras GTPase-activating protein 1a	25187966	25211932
DJ_026209-T1	Synaptic Ras GTPase-activating protein 1a	25212059	25220854
DJ_026210-T1	Ctssa protein	25245829	25250122

DJ_026211-T1	Zgc:123347	25252394	25261719	
DJ_026212-T1	Cathepsin S protein	25264222	25267154	
DJ_026213-T1	Endosulfine alpha a	25270238	25277636	
DJ_026214-T1	Uncharacterized protein	25284845	25287929	
DJ_026215-T1	Hyperpolarization-activated cyclic nucleotide-gated potassium channel 3	25296684	25305922	
DJ_026216-T1	NADH dehydrogenase [ubiquinone] flavoprotein 1, mitochondrial	25308917	25313909	
DJ_026217-T1	Uncharacterized protein	25316500	25318875	
DJ_026218-T1	histone-lysine N-methyltransferase 2B-like isoform X2	25320078	25356839	
DJ_026219-T1	Histone-lysine N-methyltransferase 2B-like protein	25358189	25360462	
DJ_026220-T1	Lysine (K)-specific methyltransferase 2Ba	25360826	25362494	
DJ_026221-T1	Peptidase S1 domain-containing protein	25392286	25395871	
DJ_026222-T1	Ribosomal protein S19	25396359	25398632	
DJ_026223-T1	uncharacterized protein il11 isoform X1	25445137	25452795	
DJ_026224-T1	Si:ch211-171h4.3	25457220	25462429	
DJ_026225-T1	Zgc:73124	25475606	25481820	
DJ_026226-T1	Zgc:194578 protein	25484863	25488751	
DJ_026227-T1	hypothetical protein	25489166	25492681	
DJ_026228-T1	Si:ch211-232m10.6	25499383	25502316	
DJ_026229-T1	germ cell-specific gene 1-like protein	25505911	25507116	
DJ_026230-T1	Recoverin 3	25512023	25515522	
DJ_026231-T1	Uncharacterized protein	25525259	25527271	
DJ_026232-T1	coiled-coil domain-containing protein 106-like	25527928	25529740	
DJ_026233-T1	U2 snRNP auxiliary factor large subunit	25532258	25541378	
DJ_026234-T1	CCR4-NOT transcription complex subunit 3	25547682	25553081	
DJ_026235-T1	CCR4-NOT transcription complex, subunit 3b	25555715	25561791	
DJ_026236-T1	Shisa family member 7	25572382	25582430	
DJ_026237-T1	D site albumin promoter-binding protein a	25602534	25612050	
DJ_026238-T1	Glutamate receptor, ionotropic, N-methyl D-aspartate 2D, a	25683054	25726745	

DJ_026239-T1	Voltage-dependent calcium channel gamma-7 subunit	25752787	25761428
DJ_026240-T1	Voltage-dependent calcium channel gamma-7 subunit	25761890	25771772
DJ_026241-T1	Calcium channel, voltage-dependent, gamma subunit 8a	25792810	25797701
DJ_026242-T1	Voltage-dependent calcium channel gamma-6 subunit	25807057	25811712
DJ_026243-T1	Si:dkey-14o18.2	25837299	25844477
DJ_026244-T1	Zgc:92476	25850138	25861326
DJ_026245-T1	tyrosine-protein kinase STYK1-like isoform X1	25868990	25885367
DJ_026246-T1	Chloride channel protein	25885819	25910605
DJ_026247-T1	Family with sequence similarity 131 member Ba	25917008	25932401
DJ_026248-T1	Glyceraldehyde-3-phosphate dehydrogenase	25963493	25966768
DJ_026249-T1	Glyceraldehyde-3-phosphate dehydrogenase	25967100	25970287
DJ_026250-T1	intermediate filament family orphan 1-like isoform X2	25971578	25978854
DJ_026251-T1	Intermediate filament family orphan 1a	25979479	25980254
DJ_026252-T1	Vesicle-associated membrane protein 1	25984334	25991875
DJ_026253-T1	fMet-Leu-Phe receptor	26004989	26007119
DJ_026254-T1	Lens intrinsic membrane protein 2	26026374	26033094
DJ_026255-T1	Upstream transcription factor 2, c-fos-interacting	26033547	26039625
DJ_026256-T1	Zgc:111885 protein	26053691	26058393
DJ_026257-T1	S-acyl fatty acid synthase thioesterase, medium chain	26060619	26063687
DJ_026258-T1	hypothetical protein	26065360	26066168
DJ_026259-T1	Eukaryotic translation initiation factor 2C 3	26067258	26084413
DJ_026260-T1	Sodium/hydrogen exchanger	26091804	26096447
DJ_026261-T1	Sodium/hydrogen exchanger	26097268	26110907
DJ_026262-T1	Hormone-sensitive lipase a	26123598	26136536
DJ_026263-T1	Peptidase S1 domain-containing protein	26142993	26153203
DJ_026264-T1	Uncharacterized protein	26156213	26157469

DJ_026265-T1	DEAD (Asp-Glu-Ala-Asp) box helicase 61	26157982	26161560
DJ_026266-T1	mitochondrial import receptor subunit TOM40 homolog	26168256	26174773
DJ_026267-T1	hypothetical protein	26176484	26178902
DJ_026269-T1	Apolipoprotein A-IV a	26189110	26190308
DJ_026270-T1	Zgc:110064	26198598	26200059
DJ_026271-T1	uncharacterized protein LOC108272666	26201670	26212864
DJ_026272-T1	26S proteasome non-ATPase regulatory subunit 4	26216370	26221227
DJ_026273-T1	Phosphatidylinositol-4-phosphate 5-kinase type 1 alpha	26223459	26239920
DJ_026274-T1	Trh3	26252614	26261351
DJ_026275-T1	CUGBP Elav-like family member 3	26275063	26294364
DJ_026276-T1	CELF3	26309661	26315818
DJ_026277-T1	Regulation of nuclear pre-mRNA domain containing 2a	26319060	26325774
DJ_026278-T1	C1orf51	26326344	26350024
DJ_026279-T1	Mitochondrial 28S ribosomal protein S21	26353471	26355543
DJ_026280-T1	Cellular retinoic acid-binding protein 2	26363525	26366813
DJ_026281-T1	Secretory carrier-associated membrane protein	26371304	26380600
DJ_026282-T1	Si:ch211-81a5.1	26382978	26391939
DJ_026283-T1	Histone-lysine N-methyltransferase ASH1L	26397321	26421722
DJ_026284-T1	ASH1L	26430110	26439029
DJ_026285-T1	Ash1 (absent, small, or homeotic)-like (Drosophila)	26440239	26471111
DJ_026286-T1	Si:ch211-39a7.1	26493391	26496584
DJ_026287-T1	Si:ch211-39a7.1	26513296	26518985
DJ_026288-T1	Sorting nexin family member 27a	26590404	26608599
DJ_026289-T1	S100 calcium-binding protein	26615887	26616598
DJ_026290-T1	Tripartite motif-containing 46b	26626068	26647084
DJ_026291-T1	Tripartite motif containing 46b	26648908	26653619
DJ_026292-T1	Ephrin-A1a	26690455	26703262
DJ_026293-T1	hypothetical protein	26871712	26874756

DJ_026294-T1	hypothetical protein	26941246	26941840
DJ_026295-T1	Si:dkey-266f7.9	27007438	27010100
DJ_026296-T1	Cyclin-dependent kinases regulatory subunit	27077958	27079744
DJ_026297-T1	Pre-B-cell leukemia homeobox-interacting protein 1a	27086436	27090244
DJ_026298-T1	Pre-B-cell leukemia homeobox-interacting protein 1a	27092833	27097625
DJ_026299-T1	EF-hand domain-containing protein	27101143	27102831
DJ_026300-T1	hypothetical protein	27108991	27109418
DJ_026301-T1	Si:ch211-105c13.3	27109837	27110935
DJ_026302-T1	S100 calcium-binding protein	27119412	27122418
DJ_026304-T1	Src homology 2 domain containing E	27135928	27144577
DJ_026305-T1	Cingulin b	27170876	27185493
DJ_026306-T1	Cingulin	27186907	27188629
DJ_026307-T1	Tuftelin	27213025	27220288
DJ_026308-T1	Ubiquitin-associated protein 2-like	27224884	27239652
DJ_026309-T1	Ubiquitin associated protein 2-like	27243819	27261244
DJ_026310-T1	probable C-mannosyltransferase DPY19L4 isoform X2	27265427	27268625
DJ_026311-T1	Dpy-19 like 4	27269592	27286911
DJ_026312-T1	integrator complex subunit 8 isoform X1	27289342	27307646
DJ_026313-T1	Polyadenylate-binding protein	27414426	27423284
DJ_026314-T1	14-3-3 protein beta/alpha-A-like	27424716	27444489
DJ_026315-T1	zinc finger protein 706-like	27454732	27455333
DJ_026316-T1	Grainyhead-like protein 2-like protein	27463641	27482124
DJ_026317-T1	Neurocalcin-delta B	27498925	27507108
DJ_026318-T1	Tumor necrosis factor superfamily member 5-induced protein 1 homolog	27542037	27544500
DJ_026319-T1	Tyrosine-protein phosphatase non-receptor type	27551559	27564470
DJ_026320-T1	Transmembrane protein C9orf91 homolog	27567611	27573945
DJ_026321-T1	Proline-rich transmembrane protein 1	27579446	27587926
DJ_026322-T1	Skiv2l protein	27597818	27603390
DJ_026323-T1	Class III histocompatibility antigen RD	27624619	27629025

DJ_026324-T1	Uncharacterized protein	27631780	27634788
DJ_026325-T1	Histone-lysine N-methyltransferase EHMT2-like protein	27636697	27646035
DJ_026326-T1	Euchromatic histone-lysine N-methyltransferase 2	27646558	27664051
DJ_026327-T1	Solute carrier family 44 member 4	27676087	27680849
DJ_026328-T1	Ring finger protein 5	27693398	27702232
DJ_026329-T1	MHC class I antigen	27705250	27710317
DJ_026330-T1	Dynein, axonemal, heavy polypeptide 9-like	27726662	27732351
DJ_026331-T1	hypothetical protein	27735359	27737731
DJ_026332-T1	Dystrobrevin binding protein 1b	27764762	27773274
DJ_026333-T1	protein Jumonji	27776866	27816334
DJ_026334-T1	protein Jumonji	27828949	27845722
DJ_026335-T1	protein Jumonji	27864905	27879433
DJ_026337-T1	Par-6 partitioning defective 6-like protein gamma	28106600	28109280
DJ_026338-T1	Biogenesis of lysosome-related organelles complex 1 subunit 4	28112438	28115250
DJ_026339-T1	Solute carrier family 44 member 4	28116383	28133513
DJ_026340-T1	choline transporter-like protein 4 isoform X2	28134007	28135779
DJ_026341-T1	C2H2-type domain-containing protein	28136794	28140476
DJ_026342-T1	Si:ch73-130a3.4	28152882	28157389
DJ_026343-T1	Thioredoxin-like protein	28158032	28163290
DJ_026344-T1	Novel protein similar to vertebrate sialidase 1 (Lysosomal sialidase) (NEU1)	28163380	28169102
DJ_026345-T1	Zinc finger and BTB domain containing 8B	28173568	28176819
DJ_026346-T1	hypothetical protein	28187987	28189242
DJ_026347-T1	Hairy/enhancer-of-split related with YRPW motif-like protein	28198564	28204319
DJ_026348-T1	Zgc:91944	28213457	28227723
DJ_026349-T1	Phosphatidylinositol 4-kinase beta	28234225	28250035
DJ_026350-T1	Centromere protein A	28256578	28260814
DJ_026351-T1	MINDY lysine 48 deubiquitinase 1	28265531	28279147
DJ_026353-T1	plectin isoform X4	28299550	28315321

DJ_026354-T1	Plectin	28315876	28318445
DJ_026355-T1	Uncharacterized protein	28318879	28322057
DJ_026356-T1	Plectin	28322471	28330111
DJ_026357-T1	Plectin a	28330905	28336153
DJ_026358-T1	plectin isoform X4	28339169	28355057
DJ_026359-T1	hypothetical protein	28374574	28378083
DJ_026361-T1	Eukaryotic translation initiation factor 3 subunit 6	28488675	28507465
DJ_026362-T1	Eukaryotic translation initiation factor 3 subunit 6	28512993	28520997
DJ_026363-T1	Eukaryotic translation initiation factor 3 subunit 6	28524904	28530008
DJ_026365-T1	Thyrotropin-releasing hormone receptor b	28572979	28574541
DJ_026366-T1	nudC domain-containing protein 1	28594050	28620302
DJ_026367-T1	NudC domain containing 1	28622758	28646268
DJ_026368-T1	Polycystic kidney and hepatic disease 1 (autosomal recessive)-like 1	28651459	28706109
DJ_026369-T1	Polycystic kidney and hepatic disease-like 1	28712084	28714999
DJ_026370-T1	fibrocystin-L isoform X2	28715779	28722245
DJ_026371-T1	Bone morphogenetic protein 13b-2	28724308	28725990
DJ_026372-T1	Hippocalcin	28872017	28876604
DJ_026373-T1	Transmembrane protein 54b	28901303	28902643
DJ_026374-T1	GA-binding protein subunit beta-2-like isoform X4	28906586	28920844
DJ_026375-T1	Uncharacterized protein	28942202	28945621
DJ_026376-T1	Catenin beta-1	28960266	28963127
DJ_026377-T1	Si:ch211-240b21.2	28966204	28967526
DJ_026378-T1	catenin beta-1	28972811	28995539
DJ_026379-T1	Trafficking kinesin protein 1	29005894	29015657
DJ_026380-T1	Cilia-and flagella-associated 44	29016524	29018667
DJ_026381-T1	Cilia-and flagella-associated 44	29033853	29043029
DJ_026382-T1	Gastrin domain-containing protein	29046050	29046981
DJ_026383-T1	hypothetical protein	29062005	29063084
DJ_026384-T1	hypothetical protein	29063122	29065759

DJ_026385-T1	Endothelin-1-like protein	29258932	29262408
DJ_026386-T1	Zinc finger 40-like isoform X2	29263113	29281404
DJ_026388-T1	Enhancer of filamentation 1-like protein	29536650	29540028
DJ_026389-T1	Epithelial-stromal interaction protein 1	29540132	29540778
DJ_026390-T1	Uncharacterized protein	29540817	29544470
DJ_026391-T1	Uncharacterized protein	29661298	29664159
DJ_026392-T1	hypothetical protein	29719611	29720485
DJ_026393-T1	hypothetical protein	29737686	29738571
DJ_026394-T1	hypothetical protein	29781426	29784479
DJ_026395-T1	uncharacterized protein LOC108272626 isoform X3	29815147	29820268
DJ_026396-T1	Uncharacterized protein	29823314	29826308
DJ_026397-T1	semaphorin-4A-like isoform X2	29829389	29851116
DJ_026399-T1	Uncharacterized protein	29910274	29922094
DJ_026400-T1	Uncharacterized protein	29923598	29925865
DJ_026401-T1	protein S100-A14	29933481	29936595
DJ_026402-T1	THAP domain-containing protein 7	29951353	29954732
DJ_026403-T1	snRNP core protein D1	29955563	29958755
DJ_026404-T1	Guanosine 5-monophosphate oxidoreductase	29986774	29995564
DJ_026405-T1	Guanosine 5-monophosphate oxidoreductase	29996426	30000360
DJ_026406-T1	Ataxin 1a	30023684	30029528
DJ_026407-T1	hypothetical protein	30097959	30105093
DJ_026408-T1	Protein tyrosine phosphatase receptor type Ua	30133922	30144425
DJ_026409-T1	Protein tyrosine phosphatase receptor type Ub	30185854	30194645
DJ_026410-T1	Protein tyrosine phosphatase receptor type Ua	30213650	30230618
DJ_026411-T1	Protein tyrosine phosphatase, receptor type, U, a	30240132	30253150
DJ_026412-T1	Protein tyrosine phosphatase, receptor type, U, a	30255848	30311042
DJ_026414-T1	Inosine-guanosine phosphorylase	30530698	30539145
DJ_026415-T1	coiled-coil domain-containing protein 94	30545848	30554990

DJ_026416-T1	hypothetical protein	30555000	30555782
DJ_026417-T1	hypothetical protein	30594870	30595403
DJ_026418-T1	Stathmin	30595624	30598784
DJ_026419-T1	Serine incorporator 2	30640383	30649322
DJ_026420-T1	Human immunodeficiency virus type I enhancer binding protein 3a	30687176	30701965
DJ_026421-T1	Uncharacterized protein	30715479	30720882
DJ_026422-T1	hypothetical protein	30725410	30728505
DJ_026423-T1	hypothetical protein	30774227	30777938
DJ_026424-T1	Phosphatase and actin regulator 4a	30780903	30791563
DJ_026425-T1	Phosphatase and actin regulator	30794848	30797660
DJ_026426-T1	G_PROTEIN_RECEP_F1_2 domain-containing protein	30839265	30840731
DJ_026427-T1	Terminal nucleotidyltransferase 5Bb	31034749	31037855
DJ_026428-T1	HNRPR	31041544	31048480
DJ_026429-T1	heterogeneous nuclear ribonucleoprotein R	31051080	31055948
DJ_026430-T1	ATP-binding cassette, sub-family B (MDR\/TAP), member 3	31063544	31068019
DJ_026431-T1	Proteasome subunit beta	31070001	31072210
DJ_026432-T1	Proteasome subunit beta	31082786	31085222
DJ_026433-T1	Proteasome subunit beta	31085397	31087567
DJ_026434-T1	Proteasome subunit beta	31088507	31091123
DJ_026435-T1	MHC class I antigen 1B	31093807	31100778
DJ_026436-T1	MHC class I antigen 1B	31110169	31115440
DJ_026437-T1	Tapasin	31124625	31128644
DJ_026438-T1	Daxx domain-containing protein	31130242	31131465
DJ_026439-T1	zinc finger and BTB domain-containing protein 22-like	31142499	31145187
DJ_026440-T1	Kinesin-like protein	31146550	31153640
DJ_026441-T1	Znf384l protein	31157228	31169963
DJ_026442-T1	Reggie-2	31176679	31179400
DJ_026443-T1	Flotillin	31180157	31185490
DJ_026444-T1	Tubulin beta chain	31187719	31193828
DJ_026445-T1	Uncharacterized protein	31201525	31218699

DJ_026446-T1	transcription factor 19-like	31225670	31228341
DJ_026447-T1	PVR cell adhesion molecule related 2 like	31298390	31306920
DJ_026448-T1	PVR cell adhesion molecule related 2 like	31324247	31326619
DJ_026450-T1	Uncharacterized protein	31423259	31429292
DJ_026452-T1	Sodium/potassium-transporting ATPase subunit alpha	31473434	31475663
DJ_026453-T1	Sodium/potassium-transporting ATPase subunit alpha	31477691	31495028
DJ_026454-T1	hypothetical protein	31538479	31544725
DJ_026455-T1	POU domain protein	31561257	31565887
DJ_026456-T1	POU domain protein	31569865	31581737
DJ_026457-T1	Ets2 repressor factor	31613599	31625563
DJ_026458-T1	Capicua transcriptional repressor a	31638832	31661895
DJ_026459-T1	Glycogen synthase kinase 3 alpha a	31666539	31679694
DJ_026460-T1	Uncharacterized protein	31691104	31695690
DJ_026461-T1	Uncharacterized protein	31705181	31709786
DJ_026462-T1	Glutamate receptor, ionotropic, kainate 5	31787283	31810719
DJ_026463-T1	Glutamate receptor, ionotropic, kainate 5	31815583	31838017
DJ_026464-T1	Carcinoembryonic antigen-related cell adhesion molecule 1-like protein	31848873	31860134
DJ_026465-T1	hypothetical protein	31890006	31893323
DJ_026466-T1	hypothetical protein	31986667	31996346

Table S8 Frequency of three karyotypes and two rearrangement variants of the two chromosome inversions on LG6 and LG22 in the anadromous population (Yangtze River Estuary) and other four freshwater resident populations.

Populations	AA		AB		BB		A		B	
	LG6	LG22	LG6	LG22	LG6	LG22	LG6	LG22	LG6	LG22
Chaohu Lake	0.00(0)	0.00(0)	0.00(0)	0.17(4)	1.00(24)	0.83(20)	0.00	0.09	1.00	0.91
Hongze Lake	0.00(0)	0.04(1)	0.17(4)	0.42(10)	0.83(20)	0.54(13)	0.09	0.25	0.91	0.75
Luoma Lake	0.00(0)	0.17(3)	0.22(4)	0.33(6)	0.78(14)	0.50(9)	0.11	0.34	0.89	0.66
Taihu Lake	0.17(4)	0.00(0)	0.33(8)	0.04(1)	0.50(12)	0.96(23)	0.34	0.02	0.66	0.98
Yangtze River Estuary	1.00(21)	0.81(17)	0.00(0)	0.14(3)	0.00(0)	0.05(1)	1.00	0.88	0.00	0.12

AA: homokaryotypes with reference arrangements, BB: homokaryotypes with alternative arrangements, AB: heterokaryotypes with both reference and alternative arrangements. Values in bracket represent the count of individuals.

Table S9 Population pairwise F_{ST} obtained by using all SNPs located in the chromosome inversion regions (LG6 and LG22).

	Yangtze River Estuary	Chaohu Lake	Hongze Lake	Luoma Lake	Taihu Lake
Yangtze River Estuary	0.00				
Chaohu Lake	**0.42**	0.00			
Hongze Lake	**0.34**	**0.05**	0.00		
Luoma Lake	**0.30**	**0.08**	0.00	0.00	
Taihu Lake	**0.41**	**0.03**	**0.09**	**0.11**	0.00

Significant values after FDR-BY correction ($P < 0.01707$) are highlighted in bold, negative values are converted to zero.

Table S10 Gene annotations of the candidate outlier SNPs.

No	#Chromosome	SNP position	Gene ID	Gene name	Gene location		SNP effect	Category
					start	end		
1	LG10	19979662	DJ_002220-T1	cell wall protein DAN4-like	19971151	19977977	upstream_gene_variant	Growth
2	LG10	19979662	DJ_002221-T1	N/A	19979378	19981141	intron_variant	N/A
3	LG11	10497259	DJ_003000-T1	myosin heavy chain, fast skeletal muscle-like	10491102	10502436	synonymous_variant	Thermal response
4	LG11	10497345	DJ_003000-T1	myosin heavy chain, fast skeletal muscle-like	10491102	10502436	intron_variant	Thermal response
5	LG11	10497398	DJ_003000-T1	myosin heavy chain, fast skeletal muscle-like	10491102	10502436	intron_variant	Thermal response
6	LG11	10497399	DJ_003000-T1	myosin heavy chain, fast skeletal muscle-like	10491102	10502436	intron_variant	Thermal response
7	LG11	10497462	DJ_003000-T1	myosin heavy chain, fast skeletal muscle-like	10491102	10502436	intron_variant	Thermal response
8	LG11	10503646	DJ_003000-T1	myosin heavy chain, fast skeletal muscle-like	10491102	10502436	upstream_gene_variant	Thermal response
9	LG11	10503698	DJ_003000-T1	myosin heavy chain, fast skeletal muscle-like	10491102	10502436	upstream_gene_variant	Thermal response
10	LG11	10503790	DJ_003000-T1	myosin heavy chain, fast skeletal muscle-like	10491102	10502436	upstream_gene_variant	Thermal response
11	LG11	10529081	DJ_003001-T1	myosin heavy chain, fast skeletal muscle-like	10533018	10533406	downstream_gene_variant	Thermal response
12	LG11	10529081	DJ_003002-T1	myosin heavy chain, fast skeletal muscle-like	10534224	10544361	downstream_gene_variant	Thermal response
13	LG11	10533169	DJ_003001-T1	myosin heavy chain, fast skeletal muscle-like	10533018	10533406	intron_variant	Thermal response
14	LG11	10533169	DJ_003002-T1	myosin heavy chain, fast skeletal muscle-like	10534224	10544361	downstream_gene_variant	Thermal response
15	LG11	10533170	DJ_003001-T1	myosin heavy chain, fast skeletal muscle-like	10533018	10533406	intron_variant	Thermal response
16	LG11	10533170	DJ_003002-T1	myosin heavy chain, fast skeletal muscle-like	10534224	10544361	downstream_gene_variant	Thermal response
17	LG11	10550494	DJ_003002-T1	myosin heavy chain, fast skeletal muscle-like	10534224	10544361	upstream_gene_variant	Thermal response
18	LG11	10550496	DJ_003002-T1	myosin heavy chain, fast skeletal muscle-like	10534224	10544361	upstream_gene_variant	Thermal response
19	LG11	10550715	DJ_003002-T1	myosin heavy chain, fast skeletal muscle-like	10534224	10544361	upstream_gene_variant	Thermal response
20	LG11	10550718	DJ_003002-T1	myosin heavy chain, fast skeletal muscle-like	10534224	10544361	upstream_gene_variant	Thermal response

21	LG11	24751139	DJ_003581-T1	voltage-dependent calcium channel subunit alpha-2/delta-2-like isoform X2	24729995	24756129	intron_variant	Osmoregulation
22	LG15	20697803	DJ_008842-T1	Protein Wnt	20689829	20697278	upstream_gene_variant	Signal transduction
23	LG15	20697803	DJ_008843-T1	acyl-CoA desaturase-like	20700952	20704792	downstream_gene_variant	Metabolic process
24	LG15	20697856	DJ_008842-T1	Protein Wnt	20689829	20697278	upstream_gene_variant	Signal transduction
25	LG15	20697856	DJ_008843-T1	acyl-CoA desaturase-like	20700952	20704792	downstream_gene_variant	Metabolic process
26	LG15	20697877	DJ_008842-T1	Protein Wnt	20689829	20697278	upstream_gene_variant	Signal transduction
27	LG15	20697877	DJ_008843-T1	acyl-CoA desaturase-like	20700952	20704792	downstream_gene_variant	Metabolic process
28	LG20	10209241	DJ_016040-T1	DNA-directed RNA polymerase subunit	10197927	10199702	downstream_gene_variant	Transcriptional regulation
29	LG20	10209241	DJ_016041-T1	DNA-directed RNA polymerase subunit	10200180	10203917	downstream_gene_variant	Transcriptional regulation
30	LG20	10209241	DJ_016042-T1	DNA-directed RNA polymerase subunit	10204765	10207701	upstream_gene_variant	Transcriptional regulation
31	LG20	10209241	DJ_016043-T1	DNA-directed RNA polymerase subunit	10208350	10209744	synonymous_variant	Transcriptional regulation
32	LG20	15182015	DJ_016258-T1	protein diaphanous homolog 2 isoform X1	15176812	15179468	upstream_gene_variant	Growth
33	LG20	15746780	DJ_016279-T1	ATP-binding cassette subfamily C member	15738807	15749227	intron_variant	Transmembrane transport
34	LG20	15746780	DJ_016280-T1	Multidrug resistance-associated protein 5	15749298	15762026	downstream_gene_variant	Toxic stress
35	LG20	16030041	DJ_016290-T1	sodium bicarbonate transporter-like protein 11 isoform X4	16035937	16038248	downstream_gene_variant	Osmoregulation
36	LG20	16030041	DJ_016291-T1	sodium bicarbonate transporter-like protein 11 isoform X4	16038534	16049943	downstream_gene_variant	Osmoregulation
37	LG20	17542018	DJ_016348-T1	protocadherin-11 X-linked-like isoform X1	17550473	17552819	upstream_gene_variant	Metabolic process
38	LG20	17542021	DJ_016348-T1	protocadherin-11 X-linked-like isoform X1	17550473	17552819	upstream_gene_variant	Metabolic process
39	LG20	19951222	DJ_016423-T1	EGF-containing fibulin-like extracellular matrix protein 2	19927365	19942541	upstream_gene_variant	Growth
40	LG20	19951222	DJ_016424-T1	acidic fibroblast growth factor intracellular-binding protein	19948782	19955814	intron_variant	Growth
41	LG20	23482073	DJ_016568-T1	fibroblast growth factor receptor-like 1	23476436	23501328	intron_variant	Growth
42	LG20	23490945	DJ_016568-T1	fibroblast growth factor receptor-like 1	23476436	23501328	intron_variant	Growth

43	LG20	27402505	DJ_016687-T1	proheparin-binding EGF-like growth factor	27393071	27394984	upstream_gene_variant	Signal transduction
44	LG20	27402505	DJ_016688-T1	RUN and FYVE domain-containing protein 1-like	27396735	27404316	intron_variant	Transcriptional regulation
45	LG20	28247100	DJ_016732-T1	TBC1 domain family member 9B isoform X1	28211276	28241534	downstream_gene_variant	Metabolic process
46	LG20	28247100	DJ_016733-T1	MORC family CW-type zinc finger protein 3-like isoform X2	28244864	28265454	intron_variant	Growth
47	LG20	29262600	DJ_016766-T1	myosin-binding protein C, cardiac-type isoform X4	29238491	29271505	intron_variant	Locomotion
48	LG20	29408990	DJ_016775-T1	ankyrin repeat and death domain-containing protein 1A isoform X1	29392714	29407996	upstream_gene_variant	Signal transduction
49	LG20	29408990	DJ_016776-T1	HEAT repeat-containing protein 3-like isoform X2	29408438	29413875	intron_variant	Transcriptional regulation
50	LG20	29409020	DJ_016775-T1	ankyrin repeat and death domain-containing protein 1A isoform X1	29392714	29407996	upstream_gene_variant	Signal transduction
51	LG20	29409020	DJ_016776-T1	HEAT repeat-containing protein 3-like isoform X2	29408438	29413875	intron_variant	Transcriptional regulation
52	LG21	19212287	DJ_017591-T1	Signal transducer and activator of transcription	19202534	19208229	downstream_gene_variant	Signal transduction
53	LG22	31155	DJ_018359-T1	Centrosomal protein 85,-like	27157	43319	intron_variant	Growth
54	LG22	31168	DJ_018359-T1	Centrosomal protein 85,-like	27157	43319	intron_variant	Growth
55	LG22	31203	DJ_018359-T1	Centrosomal protein 85,-like	27157	43319	intron_variant	Growth
56	LG22	31249	DJ_018359-T1	Centrosomal protein 85,-like	27157	43319	intron_variant	Growth
57	LG22	31268	DJ_018359-T1	Centrosomal protein 85,-like	27157	43319	intron_variant	Growth
58	LG22	31295	DJ_018359-T1	Centrosomal protein 85,-like	27157	43319	intron_variant	Growth
59	LG22	31296	DJ_018359-T1	Centrosomal protein 85,-like	27157	43319	intron_variant	Growth
60	LG22	55220	DJ_018360-T1	Centrosomal protein 85,-like	49771	53832	downstream_gene_variant	Growth
61	LG22	55220	DJ_018362-T1	mediator of RNA polymerase II transcription subunit 28	58286	62545	upstream_gene_variant	Transcriptional regulation
62	LG22	55267	DJ_018360-T1	Centrosomal protein 85,-like	49771	53832	downstream_gene_variant	Growth
63	LG22	55267	DJ_018362-T1	mediator of RNA polymerase II transcription subunit 28	58286	62545	upstream_gene_variant	Transcriptional regulation
64	LG22	55308	DJ_018360-T1	Centrosomal protein 85,-like	49771	53832	downstream_gene_variant	Growth
65	LG22	55308	DJ_018362-T1	mediator of RNA polymerase II transcription subunit 28	58286	62545	upstream_gene_variant	Transcriptional regulation
66	LG22	55341	DJ_018360-T1	Centrosomal protein 85,-like	49771	53832	downstream_gene_variant	Growth
67	LG22	55341	DJ_018362-T1	mediator of RNA polymerase II transcription subunit 28	58286	62545	upstream_gene_variant	Transcriptional regulation
68	LG22	55349	DJ_018360-T1	Centrosomal protein 85,-like	49771	53832	downstream_gene_variant	Growth
69	LG22	55349	DJ_018362-T1	mediator of RNA polymerase II transcription subunit 28	58286	62545	upstream_gene_variant	Transcriptional regulation
70	LG22	55413	DJ_018360-T1	Centrosomal protein 85,-like	49771	53832	downstream_gene_variant	Growth

71	LG22	55413	DJ_018362-T1	mediator of RNA polymerase II transcription subunit 28	58286	62545	upstream_gene_variant	Transcriptional regulation
72	LG22	58062	DJ_018360-T1	Centrosomal protein 85,-like	49771	53832	downstream_gene_variant	Growth
73	LG22	58062	DJ_018362-T1	mediator of RNA polymerase II transcription subunit 28	58286	62545	upstream_gene_variant	Transcriptional regulation
74	LG22	58062	DJ_018363-T1	oocyte-specific F-box protein	65951	79150	downstream_gene_variant	Growth
75	LG22	63503	DJ_018360-T1	Centrosomal protein 85,-like	49771	53832	downstream_gene_variant	Growth
76	LG22	63503	DJ_018362-T1	mediator of RNA polymerase II transcription subunit 28	58286	62545	downstream_gene_variant	Transcriptional regulation
77	LG22	63503	DJ_018363-T1	oocyte-specific F-box protein	65951	79150	downstream_gene_variant	Growth
78	LG22	70156	DJ_018362-T1	mediator of RNA polymerase II transcription subunit 28	58286	62545	upstream_gene_variant	Transcriptional regulation
79	LG22	70156	DJ_018363-T1	oocyte-specific F-box protein	65951	79150	intron_variant	Growth
80	LG22	263929	DJ_018367-T1	Dehydrodolichyl diphosphate synthase complex subunit nus1	251814	265205	intron_variant	Metabolic process
81	LG22	263929	DJ_018368-T1	nephrocan-like	265796	267956	downstream_gene_variant	Transcriptional regulation
82	LG22	263929	DJ_018369-T1	N/A	269505	275535	upstream_gene_variant	N/A
83	LG22	263987	DJ_018367-T1	Dehydrodolichyl diphosphate synthase complex subunit nus1	251814	265205	intron_variant	Metabolic process
84	LG22	263987	DJ_018368-T1	nephrocan-like	265796	267956	downstream_gene_variant	Transcriptional regulation
85	LG22	263987	DJ_018369-T1	N/A	269505	275535	upstream_gene_variant	N/A
86	LG22	264530	DJ_018367-T1	Dehydrodolichyl diphosphate synthase complex subunit nus1	251814	265205	intron_variant	Metabolic process
87	LG22	264530	DJ_018368-T1	nephrocan-like	265796	267956	downstream_gene_variant	Transcriptional regulation
88	LG22	264530	DJ_018369-T1	N/A	269505	275535	upstream_gene_variant	N/A
89	LG22	274381	DJ_018367-T1	Dehydrodolichyl diphosphate synthase complex subunit nus1	251814	265205	upstream_gene_variant	Metabolic process
90	LG22	274381	DJ_018368-T1	nephrocan-like	265796	267956	upstream_gene_variant	Transcriptional regulation
91	LG22	274381	DJ_018369-T1	N/A	269505	275535	intron_variant	N/A
92	LG22	274381	DJ_018370-T1	Katanin p60 ATPase-containing subunit A1	278895	285375	downstream_gene_variant	Growth
93	LG22	276798	DJ_018368-T1	nephrocan-like	265796	267956	upstream_gene_variant	Transcriptional regulation
94	LG22	276798	DJ_018369-T1	N/A	269505	275535	downstream_gene_variant	N/A
95	LG22	276798	DJ_018370-T1	Katanin p60 ATPase-containing subunit A1	278895	285375	downstream_gene_variant	Growth
96	LG22	276800	DJ_018368-T1	nephrocan-like	265796	267956	upstream_gene_variant	Transcriptional regulation
97	LG22	276800	DJ_018369-T1	N/A	269505	275535	downstream_gene_variant	N/A

98	LG22	276800	DJ_018370-T1	Katanin p60 ATPase-containing subunit A1	278895	285375	downstream_gene_variant	Growth
99	LG22	276876	DJ_018368-T1	nephrocan-like	265796	267956	upstream_gene_variant	Transcriptional regulation
100	LG22	276876	DJ_018369-T1	N/A	269505	275535	downstream_gene_variant	N/A
101	LG22	276876	DJ_018370-T1	Katanin p60 ATPase-containing subunit A1	278895	285375	downstream_gene_variant	Growth
102	LG22	312277	DJ_018374-T1	Protein C10	302887	305267	upstream_gene_variant	Growth
103	LG22	312277	DJ_018375-T1	prostaglandin E2 receptor EP2 subtype-like	313740	315129	downstream_gene_variant	Immune response
104	LG22	312277	DJ_018377-T1	Epithelial-stromal interaction 1	321407	322872	downstream_gene_variant	Immune response
105	LG22	327135	DJ_018377-T1	Epithelial-stromal interaction 1	321407	322872	upstream_gene_variant	Immune response
106	LG22	327159	DJ_018377-T1	Epithelial-stromal interaction 1	321407	322872	upstream_gene_variant	Immune response
107	LG22	327161	DJ_018377-T1	Epithelial-stromal interaction 1	321407	322872	upstream_gene_variant	Immune response
108	LG22	327272	DJ_018377-T1	Epithelial-stromal interaction 1	321407	322872	upstream_gene_variant	Immune response
109	LG22	387806	DJ_018385-T1	DNA repair protein RAD51 homolog 2 isoform X1	391162	410744	downstream_gene_variant	Growth
110	LG22	424433	DJ_018386-T1	uncharacterized protein C1orf115-like	413124	415029	upstream_gene_variant	N/A
111	LG22	424433	DJ_018387-T1	structural maintenance of chromosomes protein 6 isoform X1	415670	428593	intron_variant	Growth
112	LG22	424433	DJ_018388-T1	NA	432662	446605	downstream_gene_variant	N/A
113	LG22	459229	DJ_018389-T1	transmembrane protein 18	449196	450396	downstream_gene_variant	Transmembrane transport
114	LG22	459273	DJ_018389-T1	transmembrane protein 18	449196	450396	downstream_gene_variant	Transmembrane transport
115	LG22	499029	DJ_018390-T1	ALK and LTK ligand 2b	497096	498110	downstream_gene_variant	Signal transduction
116	LG22	499029	DJ_018391-T1	low molecular weight phosphotyrosine protein phosphatase isoform X2	500372	514028	downstream_gene_variant	Metabolic process
117	LG22	595578	DJ_018396-T1	N/A	586052	591666	downstream_gene_variant	N/A
118	LG22	595578	DJ_018397-T1	myelin transcription factor 1-like protein	592292	600937	intron_variant	Growth
119	LG22	1088379	DJ_018410-T1	RBR-type E3 ubiquitin transferase	1087105	1092015	intron_variant	Metabolic process
120	LG22	1088379	DJ_018411-T1	RBR-type E3 ubiquitin transferase	1093084	1094160	upstream_gene_variant	Metabolic process

121	LG22	1088380	DJ_018410-T1	RBR-type E3 ubiquitin transferase	1087105	1092015	intron_variant	Metabolic process
122	LG22	1088380	DJ_018411-T1	RBR-type E3 ubiquitin transferase	1093084	1094160	upstream_gene_variant	Metabolic process
123	LG22	1095316	DJ_018410-T1	RBR-type E3 ubiquitin transferase	1087105	1092015	downstream_gene_variant	Metabolic process
124	LG22	1095316	DJ_018411-T1	RBR-type E3 ubiquitin transferase	1093084	1094160	downstream_gene_variant	Metabolic process
125	LG22	1095316	DJ_018412-T1	F-box only protein 25 isoform X1	1099493	1101515	upstream_gene_variant	Signal transduction
126	LG22	1095355	DJ_018410-T1	RBR-type E3 ubiquitin transferase	1087105	1092015	downstream_gene_variant	Metabolic process
127	LG22	1095355	DJ_018411-T1	RBR-type E3 ubiquitin transferase	1093084	1094160	downstream_gene_variant	Metabolic process
128	LG22	1095355	DJ_018412-T1	F-box only protein 25 isoform X1	1099493	1101515	upstream_gene_variant	Signal transduction
129	LG22	1095526	DJ_018410-T1	RBR-type E3 ubiquitin transferase	1087105	1092015	downstream_gene_variant	Metabolic process
130	LG22	1095526	DJ_018411-T1	RBR-type E3 ubiquitin transferase	1093084	1094160	downstream_gene_variant	Metabolic process
131	LG22	1095526	DJ_018412-T1	F-box only protein 25 isoform X1	1099493	1101515	upstream_gene_variant	Signal transduction
132	LG22	1095573	DJ_018410-T1	RBR-type E3 ubiquitin transferase	1087105	1092015	downstream_gene_variant	Metabolic process
133	LG22	1095573	DJ_018411-T1	RBR-type E3 ubiquitin transferase	1093084	1094160	downstream_gene_variant	Metabolic process
134	LG22	1095573	DJ_018412-T1	F-box only protein 25 isoform X1	1099493	1101515	upstream_gene_variant	Signal transduction
135	LG22	1114189	DJ_018413-T1	F-box only protein 25 isoform X1	1106251	1119361	intron_variant	Signal transduction
136	LG22	1114189	DJ_018414-T1	testis development-related protein	1123191	1124025	downstream_gene_variant	Growth
137	LG22	1114196	DJ_018413-T1	F-box only protein 25 isoform X1	1106251	1119361	intron_variant	Signal transduction
138	LG22	1114196	DJ_018414-T1	testis development-related protein	1123191	1124025	downstream_gene_variant	Growth
139	LG22	1129676	DJ_018414-T1	testis development-related protein	1123191	1124025	downstream_gene_variant	Growth
140	LG22	1129682	DJ_018414-T1	testis development-related protein	1123191	1124025	downstream_gene_variant	Growth
141	LG22	1129739	DJ_018414-T1	testis development-related protein	1123191	1124025	downstream_gene_variant	Growth
142	LG22	1129894	DJ_018414-T1	testis development-related protein	1123191	1124025	downstream_gene_variant	Growth
143	LG22	1140688	DJ_018415-T1	N/A	1140689	1142970	upstream_gene_variant	N/A
144	LG22	1140857	DJ_018415-T1	N/A	1140689	1142970	intron_variant	N/A
145	LG22	1140865	DJ_018415-T1	N/A	1140689	1142970	intron_variant	N/A
146	LG22	1140867	DJ_018415-T1	N/A	1140689	1142970	intron_variant	N/A
147	LG22	1140868	DJ_018415-T1	N/A	1140689	1142970	intron_variant	N/A

148	LG22	1140869	DJ_018415-T1	N/A	1140689	1142970	intron_variant	N/A
149	LG22	1140922	DJ_018415-T1	N/A	1140689	1142970	intron_variant	N/A
150	LG22	1141000	DJ_018415-T1	N/A	1140689	1142970	intron_variant	N/A
151	LG22	1148818	DJ_018415-T1	N/A	1140689	1142970	downstream_gene_variant	N/A
152	LG22	1148818	DJ_018416-T1	hypothetical protein EXN66_Car018545	1152427	1153719	downstream_gene_variant	N/A
153	LG22	1148834	DJ_018415-T1	N/A	1140689	1142970	downstream_gene_variant	N/A
154	LG22	1148834	DJ_018416-T1	hypothetical protein EXN66_Car018545	1152427	1153719	downstream_gene_variant	N/A
155	LG22	1149032	DJ_018415-T1	N/A	1140689	1142970	downstream_gene_variant	N/A
156	LG22	1149032	DJ_018416-T1	hypothetical protein EXN66_Car018545	1152427	1153719	downstream_gene_variant	N/A
157	LG22	1158165	DJ_018416-T1	hypothetical protein EXN66_Car018545	1152427	1153719	upstream_gene_variant	N/A
158	LG22	1178777	DJ_018417-T1	Tudor domain-containing protein 6	1181246	1191679	upstream_gene_variant	Growth
159	LG22	1188902	DJ_018417-T1	Tudor domain-containing protein 6	1181246	1191679	intron_variant	Growth
160	LG22	1188902	DJ_018418-T1	Platelet-activating factor acetylhydrolase	1193377	1202859	downstream_gene_variant	Metabolic process
161	LG22	1190813	DJ_018417-T1	Tudor domain-containing protein 6	1181246	1191679	intron_variant	Growth
162	LG22	1190813	DJ_018418-T1	Platelet-activating factor acetylhydrolase	1193377	1202859	downstream_gene_variant	Metabolic process
163	LG22	1195092	DJ_018417-T1	Platelet-activating factor acetylhydrolase	1181246	1191679	downstream_gene_variant	Metabolic process
164	LG22	1195092	DJ_018418-T1	Platelet-activating factor acetylhydrolase	1193377	1202859	intron_variant	Metabolic process
165	LG22	1195092	DJ_018419-T1	Meprin A subunit alpha-like	1204075	1231629	upstream_gene_variant	Metabolic process
166	LG22	1195237	DJ_018417-T1	Platelet-activating factor acetylhydrolase	1181246	1191679	downstream_gene_variant	Metabolic process
167	LG22	1195237	DJ_018418-T1	Platelet-activating factor acetylhydrolase	1193377	1202859	intron_variant	Metabolic process
168	LG22	1195237	DJ_018419-T1	Meprin A subunit alpha-like	1204075	1231629	upstream_gene_variant	Metabolic process
169	LG22	1195267	DJ_018417-T1	Platelet-activating factor acetylhydrolase	1181246	1191679	downstream_gene_variant	Metabolic process
170	LG22	1195267	DJ_018418-T1	Platelet-activating factor acetylhydrolase	1193377	1202859	intron_variant	Metabolic process
171	LG22	1195267	DJ_018419-T1	Meprin A subunit alpha-like	1204075	1231629	upstream_gene_variant	Metabolic process
172	LG22	1195287	DJ_018417-T1	Platelet-activating factor acetylhydrolase	1181246	1191679	downstream_gene_variant	Metabolic process
173	LG22	1195287	DJ_018418-T1	Platelet-activating factor acetylhydrolase	1193377	1202859	intron_variant	Metabolic process

174	LG22	1195287	DJ_018419-T1	Meprin A subunit alpha-like	1204075	1231629	upstream_gene_variant	Metabolic process
175	LG22	1200845	DJ_018417-T1	Platelet-activating factor acetylhydrolase	1181246	1191679	downstream_gene_variant	Metabolic process
176	LG22	1200845	DJ_018418-T1	Platelet-activating factor acetylhydrolase	1193377	1202859	intron_variant	Metabolic process
177	LG22	1200845	DJ_018419-T1	Meprin A subunit alpha-like	1204075	1231629	upstream_gene_variant	Metabolic process
178	LG22	1217047	DJ_018419-T1	Meprin A subunit alpha-like	1204075	1231629	upstream_gene_variant	Metabolic process
179	LG22	1217086	DJ_018419-T1	Meprin A subunit alpha-like	1204075	1231629	upstream_gene_variant	Metabolic process
180	LG22	1239612	DJ_018419-T1	Metalloendopeptidase	1204075	1231629	downstream_gene_variant	Metabolic process
181	LG22	1239612	DJ_018420-T1	adhesion G-protein coupled receptor F3-like	1233437	1244651	intron_variant	Signal transduction
182	LG22	1239714	DJ_018419-T1	Metalloendopeptidase	1204075	1231629	downstream_gene_variant	Metabolic process
183	LG22	1239714	DJ_018420-T1	adhesion G-protein coupled receptor F3-like	1233437	1244651	intron_variant	Signal transduction
184	LG22	1316305	DJ_018423-T1	Feline leukemia virus subgroup C cellular receptor family, member 2b	1309679	1337798	intron_variant	Transmembrane transport
185	LG22	1316349	DJ_018423-T1	Feline leukemia virus subgroup C cellular receptor family, member 2b	1309679	1337798	intron_variant	Transmembrane transport
186	LG22	1316411	DJ_018423-T1	Feline leukemia virus subgroup C cellular receptor family, member 2b	1309679	1337798	intron_variant	Transmembrane transport
187	LG22	1352225	DJ_018424-T1	basic leucine zipper transcriptional factor ATF-like	1344311	1351961	upstream_gene_variant	Transcriptional regulation
188	LG22	1352225	DJ_018425-T1	adhesion G protein-coupled receptor F5-like	1360156	1366710	downstream_gene_variant	Signal transduction
189	LG22	1352499	DJ_018424-T1	basic leucine zipper transcriptional factor ATF-like	1344311	1351961	upstream_gene_variant	Transcriptional regulation
190	LG22	1352499	DJ_018425-T1	adhesion G protein-coupled receptor F5-like	1360156	1366710	downstream_gene_variant	Signal transduction
191	LG22	1437504	DJ_018428-T1	solute carrier family 2, facilitated glucose transporter member 12	1426178	1434694	downstream_gene_variant	Metabolic process
192	LG22	1437504	DJ_018429-T1	TATA box-binding protein-like protein 1	1438538	1444939	downstream_gene_variant	Transmembrane transport
193	LG22	1445098	DJ_018429-T1	solute carrier family 2, facilitated glucose transporter member 12	1438538	1444939	downstream_gene_variant	Metabolic process
194	LG22	1445137	DJ_018429-T1	TATA box-binding protein-like protein 1	1438538	1444939	downstream_gene_variant	Transmembrane transport
195	LG22	1447341	DJ_018429-T1	TATA box-binding protein-like protein 1	1438538	1444939	upstream_gene_variant	Transmembrane transport

196	LG22	1447406	DJ_018429-T1	TATA box-binding protein-like protein 1	1438538	1444939	upstream_gene_variant	Transmembrane transport
197	LG22	1481191	DJ_018430-T1	Transcription factor 21	1479582	1480666	downstream_gene_variant	Growth
198	LG22	1481213	DJ_018430-T1	Transcription factor 21	1479582	1480666	downstream_gene_variant	Growth
199	LG22	1481930	DJ_018430-T1	Transcription factor 21	1479582	1480666	downstream_gene_variant	Growth
200	LG22	1481981	DJ_018430-T1	Transcription factor 21	1479582	1480666	downstream_gene_variant	Growth
201	LG22	1482036	DJ_018430-T1	Transcription factor 21	1479582	1480666	downstream_gene_variant	Growth
202	LG22	1482080	DJ_018430-T1	Transcription factor 21	1479582	1480666	downstream_gene_variant	Growth
203	LG22	1486602	DJ_018430-T1	Transcription factor 21	1479582	1480666	downstream_gene_variant	Growth
204	LG22	1486661	DJ_018430-T1	Transcription factor 21	1479582	1480666	downstream_gene_variant	Growth
205	LG22	1486685	DJ_018430-T1	Transcription factor 21	1479582	1480666	downstream_gene_variant	Growth
206	LG22	1683162	DJ_018437-T1	lebercilin-like isoform X3	1672204	1688380	intron_variant	Metabolic process
207	LG22	1685205	DJ_018437-T1	lebercilin-like isoform X3	1672204	1688380	intron_variant	Metabolic process
208	LG22	1696647	DJ_018437-T1	lebercilin-like isoform X3	1672204	1688380	downstream_gene_variant	Metabolic process
209	LG22	1716575	DJ_018438-T1	PH-interacting protein	1714489	1763153	intron_variant	Signal transduction
210	LG22	1716579	DJ_018438-T1	PH-interacting protein	1714489	1763153	intron_variant	Signal transduction
211	LG22	1716651	DJ_018438-T1	PH-interacting protein	1714489	1763153	intron_variant	Signal transduction
212	LG22	1772914	DJ_018438-T1	PH-interacting protein	1714489	1763153	downstream_gene_variant	Signal transduction
213	LG22	1772914	DJ_018439-T1	PH-interacting protein isoform X1	1775497	1794013	upstream_gene_variant	Signal transduction
214	LG22	1794401	DJ_018439-T1	PH-interacting protein isoform X1	1775497	1794013	downstream_gene_variant	Signal transduction
215	LG22	1794401	DJ_018440-T1	Tetraodon protein product CAG00085	1799835	1801507	upstream_gene_variant	Growth
216	LG22	1794401	DJ_018441-T1	interleukin-1 receptor-associated kinase 1-binding protein 1 homolog	1804400	1806484	downstream_gene_variant	Immune response
217	LG22	1794413	DJ_018439-T1	PH-interacting protein isoform X1	1775497	1794013	downstream_gene_variant	Signal transduction
218	LG22	1794413	DJ_018440-T1	Tetraodon protein product CAG00085	1799835	1801507	upstream_gene_variant	Growth
219	LG22	1794413	DJ_018441-T1	interleukin-1 receptor-associated kinase 1-binding protein 1 homolog	1804400	1806484	downstream_gene_variant	Immune response
220	LG22	1794453	DJ_018439-T1	PH-interacting protein isoform X1	1775497	1794013	downstream_gene_variant	Signal transduction
221	LG22	1794453	DJ_018440-T1	Tetraodon protein product CAG00085	1799835	1801507	upstream_gene_variant	Growth

222	LG22	1794453	DJ_018441-T1	interleukin-1 receptor-associated kinase 1-binding protein 1 homolog	1804400	1806484	downstream_gene_variant	Immune response
223	LG22	1803431	DJ_018439-T1	PH-interacting protein isoform X1	1775497	1794013	downstream_gene_variant	Signal transduction
224	LG22	1803431	DJ_018440-T1	Tetraodon protein product CAG00085	1799835	1801507	downstream_gene_variant	Growth
225	LG22	1803431	DJ_018441-T1	interleukin-1 receptor-associated kinase 1-binding protein 1 homolog	1804400	1806484	downstream_gene_variant	Immune response
226	LG22	1803431	DJ_018442-T1	Solute carrier family 25, member 47a	1809283	1812878	downstream_gene_variant	Osmoregulation
227	LG22	1803449	DJ_018439-T1	PH-interacting protein isoform X1	1775497	1794013	downstream_gene_variant	Signal transduction
228	LG22	1803449	DJ_018440-T1	Tetraodon protein product CAG00085	1799835	1801507	downstream_gene_variant	Growth
229	LG22	1803449	DJ_018441-T1	interleukin-1 receptor-associated kinase 1-binding protein 1 homolog	1804400	1806484	downstream_gene_variant	Immune response
230	LG22	1803449	DJ_018442-T1	Solute carrier family 25, member 47a	1809283	1812878	downstream_gene_variant	Osmoregulation
231	LG22	1806079	DJ_018440-T1	Tetraodon protein product CAG00085	1799835	1801507	downstream_gene_variant	Growth
232	LG22	1806079	DJ_018441-T1	interleukin-1 receptor-associated kinase 1-binding protein 1 homolog	1804400	1806484	synonymous_variant	Immune response
233	LG22	1806079	DJ_018442-T1	Solute carrier family 25, member 47a	1809283	1812878	downstream_gene_variant	Osmoregulation
234	LG22	1806079	DJ_018443-T1	mitochondrial basic amino acids transporter-like	1813863	1819501	upstream_gene_variant	Transmembrane transport
235	LG22	1806089	DJ_018440-T1	Tetraodon protein product CAG00085	1799835	1801507	downstream_gene_variant	Growth
236	LG22	1806089	DJ_018441-T1	interleukin-1 receptor-associated kinase 1-binding protein 1 homolog	1804400	1806484	missense_variant	Immune response
237	LG22	1806089	DJ_018442-T1	mitochondrial basic amino acids transporter-like	1809283	1812878	downstream_gene_variant	Transmembrane transport
238	LG22	1806089	DJ_018443-T1	Solute carrier family 25, member 47a	1813863	1819501	upstream_gene_variant	Osmoregulation
239	LG22	1837210	DJ_018444-T1	mitochondrial basic amino acids transporter-like	1833769	1838778	intron_variant	Transmembrane transport
240	LG22	1849861	DJ_018445-T1	serine/threonine-protein kinase PAK 6-like isoform X2	1851728	1864904	downstream_gene_variant	Hypoxia
241	LG22	1849940	DJ_018445-T1	serine/threonine-protein kinase PAK 6-like isoform X2	1851728	1864904	downstream_gene_variant	Hypoxia
242	LG22	1850029	DJ_018445-T1	serine/threonine-protein kinase PAK 6-like isoform X2	1851728	1864904	downstream_gene_variant	Hypoxia
243	LG22	1860421	DJ_018445-T1	serine/threonine-protein kinase PAK 6-like isoform X2	1851728	1864904	intron_variant	Hypoxia

244	LG22	1900320	DJ_018447-T1	pre-mRNA-splicing regulator WTAP	1891460	1899164	upstream_gene_variant	Transcriptional regulation
245	LG22	1900320	DJ_018448-T1	manganese superoxide dismutase	1901204	1904205	upstream_gene_variant	Immune response
246	LG22	1900320	DJ_018449-T1	superoxide dismutase [Mn], mitochondrial	1904247	1905654	upstream_gene_variant	Immune response
247	LG22	1990705	DJ_018450-T1	fibronectin type III domain-containing protein 1	1977284	1983452	upstream_gene_variant	Growth
248	LG22	1990705	DJ_018451-T1	fibronectin type III domain-containing protein 1 isoform X3	1985541	2022246	intron_variant	Growth
249	LG22	2025932	DJ_018451-T1	fibronectin type III domain-containing protein 1 isoform X3	1985541	2022246	upstream_gene_variant	Growth
250	LG22	2025932	DJ_018452-T1	fibronectin type III domain-containing protein 1	2025508	2057909	intron_variant	Growth
251	LG22	2028531	DJ_018451-T1	fibronectin type III domain-containing protein 1 isoform X3	1985541	2022246	upstream_gene_variant	Growth
252	LG22	2028531	DJ_018452-T1	fibronectin type III domain-containing protein 1	2025508	2057909	intron_variant	Growth
253	LG22	2028549	DJ_018451-T1	fibronectin type III domain-containing protein 1 isoform X3	1985541	2022246	upstream_gene_variant	Growth
254	LG22	2028549	DJ_018452-T1	fibronectin type III domain-containing protein 1	2025508	2057909	intron_variant	Growth
255	LG22	2028588	DJ_018451-T1	fibronectin type III domain-containing protein 1 isoform X3	1985541	2022246	upstream_gene_variant	Growth
256	LG22	2028588	DJ_018452-T1	fibronectin type III domain-containing protein 1	2025508	2057909	intron_variant	Growth
257	LG22	2051481	DJ_018452-T1	fibronectin type III domain-containing protein 1	2025508	2057909	intron_variant	Growth
258	LG22	2051507	DJ_018452-T1	fibronectin type III domain-containing protein 1	2025508	2057909	intron_variant	Growth
259	LG22	2051531	DJ_018452-T1	fibronectin type III domain-containing protein 1	2025508	2057909	intron_variant	Growth
260	LG22	2051835	DJ_018452-T1	fibronectin type III domain-containing protein 1	2025508	2057909	intron_variant	Growth
261	LG22	2051848	DJ_018452-T1	fibronectin type III domain-containing protein 1	2025508	2057909	intron_variant	Growth
262	LG22	2185757	DJ_018456-T1	Otoferlin	2161396	2185378	downstream_gene_variant	Transmembrane transport
263	LG22	2185757	DJ_018457-T1	protein disulfide-isomerase A6-like	2187313	2198239	upstream_gene_variant	Immune response
264	LG22	2185777	DJ_018456-T1	Otoferlin	2161396	2185378	downstream_gene_variant	Transmembrane transport
265	LG22	2185777	DJ_018457-T1	protein disulfide-isomerase A6-like	2187313	2198239	upstream_gene_variant	Immune response

266	LG22	2202938	DJ_018457-T1	protein disulfide-isomerase A6-like	2187313	2198239	downstream_gene_variant	Immune response
267	LG22	2202938	DJ_018458-T1	Hippocalcin-like protein 1	2204120	2206236	downstream_gene_variant	Signal transduction
268	LG22	2202970	DJ_018457-T1	protein disulfide-isomerase A6-like	2187313	2198239	downstream_gene_variant	Immune response
269	LG22	2202970	DJ_018458-T1	Hippocalcin-like protein 1	2204120	2206236	downstream_gene_variant	Signal transduction
270	LG22	2228017	DJ_018459-T1	apolipoprotein B-100 isoform X2	2234407	2252357	upstream_gene_variant	Metabolic process
271	LG22	2228061	DJ_018459-T1	apolipoprotein B-100 isoform X2	2234407	2252357	upstream_gene_variant	Metabolic process
272	LG22	2228076	DJ_018459-T1	apolipoprotein B-100 isoform X2	2234407	2252357	upstream_gene_variant	Metabolic process
273	LG22	2228115	DJ_018459-T1	apolipoprotein B-100 isoform X2	2234407	2252357	upstream_gene_variant	Metabolic process
274	LG22	2228150	DJ_018459-T1	apolipoprotein B-100 isoform X2	2234407	2252357	upstream_gene_variant	Metabolic process
275	LG22	2228153	DJ_018459-T1	apolipoprotein B-100 isoform X2	2234407	2252357	upstream_gene_variant	Metabolic process
276	LG22	2248412	DJ_018459-T1	apolipoprotein B-100 isoform X2	2234407	2252357	missense_variant	Metabolic process
277	LG22	2248412	DJ_018460-T1	apolipoprotein B-100 isoform X2	2253445	2273066	upstream_gene_variant	Metabolic process
278	LG22	2248437	DJ_018459-T1	apolipoprotein B-100 isoform X2	2234407	2252357	synonymous_variant	Metabolic process
279	LG22	2248437	DJ_018460-T1	apolipoprotein B-100 isoform X2	2253445	2273066	upstream_gene_variant	Metabolic process
280	LG22	2281750	DJ_018460-T1	apolipoprotein B-100 isoform X2	2253445	2273066	downstream_gene_variant	Metabolic process
281	LG22	2281750	DJ_018461-T1	apolipoprotein B-100 isoform X2	2273194	2275809	downstream_gene_variant	Metabolic process
282	LG22	2281750	DJ_018463-T1	apolipoprotein B-100 isoform X2	2285382	2303651	upstream_gene_variant	Metabolic process
283	LG22	2281908	DJ_018460-T1	apolipoprotein B-100 isoform X2	2253445	2273066	downstream_gene_variant	Metabolic process
284	LG22	2281908	DJ_018461-T1	apolipoprotein B-100 isoform X2	2273194	2275809	downstream_gene_variant	Metabolic process
285	LG22	2281908	DJ_018463-T1	apolipoprotein B-100 isoform X2	2285382	2303651	upstream_gene_variant	Metabolic process
286	LG22	2293953	DJ_018463-T1	apolipoprotein B-100 isoform X2	2285382	2303651	intron_variant	Metabolic process
287	LG22	2309147	DJ_018463-T1	apolipoprotein B-100 isoform X2	2285382	2303651	downstream_gene_variant	Metabolic process

288	LG22	2309147	DJ_018464-T1	mitotic checkpoint serine/threonine-protein kinase BUB1 beta-like isoform X1	2307168	2321248	downstream_gene_variant	Growth
289	LG22	2382597	DJ_018466-T1	N/A	2367623	2373776	upstream_gene_variant	N/A
290	LG22	2382622	DJ_018466-T1	N/A	2367623	2373776	upstream_gene_variant	N/A
291	LG22	2382629	DJ_018466-T1	N/A	2367623	2373776	upstream_gene_variant	N/A
292	LG22	2392161	DJ_018467-T1	MAM domain-containing glycosylphosphatidylinositol anchor protein 2	2394244	2419100	downstream_gene_variant	Growth
293	LG22	2392203	DJ_018467-T1	MAM domain-containing glycosylphosphatidylinositol anchor protein 2	2394244	2419100	downstream_gene_variant	Growth
294	LG22	2392269	DJ_018467-T1	MAM domain-containing glycosylphosphatidylinositol anchor protein 2	2394244	2419100	downstream_gene_variant	Growth
295	LG22	2411695	DJ_018467-T1	MAM domain-containing glycosylphosphatidylinositol anchor protein 2	2394244	2419100	intron_variant	Growth
296	LG22	2488794	DJ_018470-T1	MAM domain-containing glycosylphosphatidylinositol anchor protein 2	2483463	2504450	intron_variant	Growth
297	LG22	2488827	DJ_018470-T1	MAM domain-containing glycosylphosphatidylinositol anchor protein 2	2483463	2504450	intron_variant	Growth
298	LG22	2516312	DJ_018471-T1	MAM domain-containing glycosylphosphatidylinositol anchor protein 2	2523180	2529928	downstream_gene_variant	Growth
299	LG22	2516424	DJ_018471-T1	MAM domain-containing glycosylphosphatidylinositol anchor protein 2	2523180	2529928	downstream_gene_variant	Growth
300	LG22	2519301	DJ_018471-T1	MAM domain-containing glycosylphosphatidylinositol anchor protein 2	2523180	2529928	downstream_gene_variant	Growth
301	LG22	2519338	DJ_018471-T1	MAM domain-containing glycosylphosphatidylinositol anchor protein 2	2523180	2529928	downstream_gene_variant	Growth
302	LG22	2519394	DJ_018471-T1	MAM domain-containing glycosylphosphatidylinositol anchor protein 2	2523180	2529928	downstream_gene_variant	Growth
303	LG22	2519666	DJ_018471-T1	MAM domain-containing glycosylphosphatidylinositol anchor protein 2	2523180	2529928	downstream_gene_variant	Growth
304	LG22	2921319	DJ_018480-T1	Thyroxine 5-deiodinase	2920145	2920948	upstream_gene_variant	Visual perception

305	LG22	2921325	DJ_018480-T1	Thyroxine 5-deiodinase	2920145	2920948	upstream_gene_variant	Visual perception
306	LG22	2921359	DJ_018480-T1	Thyroxine 5-deiodinase	2920145	2920948	upstream_gene_variant	Visual perception
307	LG22	2921640	DJ_018480-T1	Thyroxine 5-deiodinase	2920145	2920948	upstream_gene_variant	Visual perception
308	LG22	2921773	DJ_018480-T1	Thyroxine 5-deiodinase	2920145	2920948	upstream_gene_variant	Visual perception
309	LG22	4778799	DJ_018554-T1	Fc receptor-like protein 5 isoform X11	4764274	4791185	intron_variant	Immune response
310	LG22	4788845	DJ_018554-T1	Fc receptor-like protein 5 isoform X11	4764274	4791185	intron_variant	Immune response
311	LG22	4881861	DJ_018561-T1	E3 ubiquitin-protein ligase pellino homolog 2-like	4861798	4875584	downstream_gene_variant	Signal transduction
312	LG22	4908582	DJ_018562-T1	homeobox protein OTX2	4913011	4914516	downstream_gene_variant	Growth
313	LG22	4908657	DJ_018562-T1	homeobox protein OTX2	4913011	4914516	downstream_gene_variant	Growth
314	LG22	4916584	DJ_018562-T1	homeobox protein OTX2	4913011	4914516	upstream_gene_variant	Growth
315	LG22	4916759	DJ_018562-T1	homeobox protein OTX2	4913011	4914516	upstream_gene_variant	Growth
316	LG22	4916818	DJ_018562-T1	homeobox protein OTX2	4913011	4914516	upstream_gene_variant	Growth
317	LG22	4916832	DJ_018562-T1	homeobox protein OTX2	4913011	4914516	upstream_gene_variant	Growth
318	LG22	4984116	DJ_018565-T1	Actin-related protein 10	4980441	4990341	intron_variant	Locomotion
319	LG22	4984162	DJ_018565-T1	Actin-related protein 10	4980441	4990341	intron_variant	Locomotion
320	LG22	4996917	DJ_018565-T1	Actin-related protein 10	4980441	4990341	downstream_gene_variant	Locomotion
321	LG22	4996917	DJ_018566-T1	CLOCK-interacting pacemaker	5005636	5015230	upstream_gene_variant	Transcriptional regulation
322	LG22	5018083	DJ_018566-T1	CLOCK-interacting pacemaker	5005636	5015230	downstream_gene_variant	Transcriptional regulation
323	LG22	5018083	DJ_018567-T1	zinc finger protein 410	5021541	5028615	upstream_gene_variant	Transcriptional regulation
324	LG22	5018085	DJ_018566-T1	CLOCK-interacting pacemaker	5005636	5015230	downstream_gene_variant	Transcriptional regulation
325	LG22	5018085	DJ_018567-T1	zinc finger protein 410	5021541	5028615	upstream_gene_variant	Transcriptional regulation
326	LG22	5026815	DJ_018567-T1	zinc finger protein 410	5021541	5028615	intron_variant	Transcriptional regulation
327	LG22	5026815	DJ_018568-T1	ectonucleoside triphosphate diphosphohydrolase 5-like	5030376	5043700	downstream_gene_variant	Metabolic process
328	LG22	5079455	DJ_018570-T1	sterile alpha motif domain-containing protein 15	5068927	5071728	downstream_gene_variant	Immune response
329	LG22	5079455	DJ_018571-T1	leucine-rich repeat and fibronectin type-III domain-containing protein 5-like	5084394	5098575	upstream_gene_variant	Assembly

330	LG22	5079597	DJ_018570-T1	sterile alpha motif domain-containing protein 15	5068927	5071728	downstream_gene_variant	Immune response
331	LG22	5079597	DJ_018571-T1	leucine-rich repeat and fibronectin type-III domain-containing protein 5-like	5084394	5098575	upstream_gene_variant	Assembly
332	LG22	5079599	DJ_018570-T1	sterile alpha motif domain-containing protein 15	5068927	5071728	downstream_gene_variant	Immune response
333	LG22	5079599	DJ_018571-T1	leucine-rich repeat and fibronectin type-III domain-containing protein 5-like	5084394	5098575	upstream_gene_variant	Assembly
334	LG22	5079616	DJ_018570-T1	sterile alpha motif domain-containing protein 15	5068927	5071728	downstream_gene_variant	Immune response
335	LG22	5079616	DJ_018571-T1	leucine-rich repeat and fibronectin type-III domain-containing protein 5-like	5084394	5098575	upstream_gene_variant	Assembly
336	LG22	5079636	DJ_018570-T1	sterile alpha motif domain-containing protein 15	5068927	5071728	downstream_gene_variant	Immune response
337	LG22	5079636	DJ_018571-T1	leucine-rich repeat and fibronectin type-III domain-containing protein 5-like	5084394	5098575	upstream_gene_variant	Assembly
338	LG22	5079647	DJ_018570-T1	sterile alpha motif domain-containing protein 15	5068927	5071728	downstream_gene_variant	Immune response
339	LG22	5079647	DJ_018571-T1	leucine-rich repeat and fibronectin type-III domain-containing protein 5-like	5084394	5098575	upstream_gene_variant	Assembly
340	LG22	5079704	DJ_018570-T1	sterile alpha motif domain-containing protein 15	5068927	5071728	downstream_gene_variant	Immune response
341	LG22	5079704	DJ_018571-T1	leucine-rich repeat and fibronectin type-III domain-containing protein 5-like	5084394	5098575	upstream_gene_variant	Assembly
342	LG22	5079727	DJ_018570-T1	sterile alpha motif domain-containing protein 15	5068927	5071728	downstream_gene_variant	Immune response
343	LG22	5079727	DJ_018571-T1	leucine-rich repeat and fibronectin type-III domain-containing protein 5-like	5084394	5098575	upstream_gene_variant	Assembly
344	LG22	5173394	DJ_018575-T1	echinoderm microtubule-associated protein-like 1	5174214	5184281	downstream_gene_variant	Growth
345	LG22	5173516	DJ_018575-T1	echinoderm microtubule-associated protein-like 1	5174214	5184281	downstream_gene_variant	Growth
346	LG22	5185043	DJ_018575-T1	echinoderm microtubule-associated protein-like 1	5174214	5184281	upstream_gene_variant	Growth
347	LG22	5185043	DJ_018576-T1	bromo adjacent homology domain-containing 1 protein-like	5191301	5192767	downstream_gene_variant	Growth
348	LG22	5185043	DJ_018577-T1	bromo adjacent homology domain-containing 1 protein-like	5192770	5197021	downstream_gene_variant	Growth

349	LG22	5201451	DJ_018576-T1	bromo adjacent homology domain-containing 1 protein-like	5191301	5192767	upstream_gene_variant	Growth
350	LG22	5201451	DJ_018577-T1	bromo adjacent homology domain-containing 1 protein-like	5192770	5197021	upstream_gene_variant	Growth
351	LG22	5201462	DJ_018576-T1	bromo adjacent homology domain-containing 1 protein-like	5191301	5192767	upstream_gene_variant	Growth
352	LG22	5201462	DJ_018577-T1	bromo adjacent homology domain-containing 1 protein-like	5192770	5197021	upstream_gene_variant	Growth
353	LG22	5201486	DJ_018576-T1	bromo adjacent homology domain-containing 1 protein-like	5191301	5192767	upstream_gene_variant	Growth
354	LG22	5201486	DJ_018577-T1	bromo adjacent homology domain-containing 1 protein-like	5192770	5197021	upstream_gene_variant	Growth
355	LG22	5203765	DJ_018577-T1	bromo adjacent homology domain-containing 1 protein-like	5191301	5192767	upstream_gene_variant	Growth
356	LG22	5203842	DJ_018577-T1	bromo adjacent homology domain-containing 1 protein-like	5192770	5197021	upstream_gene_variant	Growth
357	LG22	5219072	DJ_018579-T1	MAX gene-associated protein-like isoform X3	5216531	5235272	intron_variant	Transcriptional regulation
358	LG22	5271409	DJ_018582-T1	kinesin-like protein KIF25 isoform X1	5260576	5261893	downstream_gene_variant	Metabolic process
359	LG22	5271409	DJ_018583-T1	heme-binding protein 2-like	5263773	5266798	downstream_gene_variant	Metabolic process
360	LG22	5271409	DJ_018584-T1	heme-binding protein 2-like	5268090	5269842	downstream_gene_variant	Metabolic process
361	LG22	5271409	DJ_018585-T1	NHS-like protein 1 isoform X3	5276224	5284560	downstream_gene_variant	Growth
362	LG22	5874706	DJ_018597-T1	poly [ADP-ribose] polymerase 12-like isoform X1	5882492	5889390	upstream_gene_variant	Signal transduction
363	LG22	5874731	DJ_018597-T1	poly [ADP-ribose] polymerase 12-like isoform X1	5882492	5889390	upstream_gene_variant	Signal transduction
364	LG22	5892479	DJ_018597-T1	poly [ADP-ribose] polymerase 12-like isoform X1	5882492	5889390	downstream_gene_variant	Signal transduction
365	LG22	5892479	DJ_018598-T1	Aldehyde dehydrogenase family 8 member A1	5891634	5897665	synonymous_variant	Metabolic process
366	LG22	5892479	DJ_018599-T1	HBS1-like protein isoform X2	5901081	5925199	downstream_gene_variant	Signal transduction
367	LG22	5897464	DJ_018597-T1	poly [ADP-ribose] polymerase 12-like isoform X1	5882492	5889390	downstream_gene_variant	Signal transduction
368	LG22	5897464	DJ_018598-T1	Aldehyde dehydrogenase family 8 member A1	5891634	5897665	intron_variant	Metabolic process
369	LG22	5897464	DJ_018599-T1	HBS1-like protein isoform X2	5901081	5925199	downstream_gene_variant	Signal transduction
370	LG22	5900291	DJ_018598-T1	Aldehyde dehydrogenase family 8 member A1	5891634	5897665	upstream_gene_variant	Metabolic process

371	LG22	5900291	DJ_018599-T1	HBS1-like protein isoform X2	5901081	5925199	downstream_gene_variant	Signal transduction
372	LG22	5900305	DJ_018598-T1	Aldehyde dehydrogenase family 8 member A1	5891634	5897665	upstream_gene_variant	Metabolic process
373	LG22	5900305	DJ_018599-T1	HBS1-like protein isoform X2	5901081	5925199	downstream_gene_variant	Signal transduction
374	LG22	5900306	DJ_018598-T1	Aldehyde dehydrogenase family 8 member A1	5891634	5897665	upstream_gene_variant	Metabolic process
375	LG22	5900306	DJ_018599-T1	HBS1-like protein isoform X2	5901081	5925199	downstream_gene_variant	Signal transduction
376	LG22	5900348	DJ_018598-T1	Aldehyde dehydrogenase family 8 member A1	5891634	5897665	upstream_gene_variant	Metabolic process
377	LG22	5900348	DJ_018599-T1	HBS1-like protein isoform X2	5901081	5925199	downstream_gene_variant	Signal transduction
378	LG22	5917033	DJ_018599-T1	HBS1-like protein isoform X2	5901081	5925199	intron_variant	Signal transduction
379	LG22	5937937	DJ_018600-T1	HBS1-like protein isoform X2	5945194	5948096	downstream_gene_variant	Signal transduction
380	LG22	5938008	DJ_018600-T1	HBS1-like protein isoform X2	5945194	5948096	downstream_gene_variant	Signal transduction
381	LG22	5938095	DJ_018600-T1	HBS1-like protein isoform X2	5945194	5948096	downstream_gene_variant	Signal transduction
382	LG22	5967998	DJ_018601-T1	transcriptional activator Myb isoform X2	5959078	5970892	synonymous_variant	Transcriptional regulation
383	LG22	6202985	DJ_018605-T1	N/A	6194673	6197633	upstream_gene_variant	N/A
384	LG22	6203001	DJ_018605-T1	N/A	6194673	6197633	upstream_gene_variant	N/A
385	LG22	6323182	DJ_018612-T1	zinc transporter 2	6312065	6323309	intron_variant	Osmoregulation
386	LG22	6323182	DJ_018613-T1	Uridine-cytidine kinase	6324255	6334020	upstream_gene_variant	Signal transduction
387	LG22	6323212	DJ_018612-T1	zinc transporter 2	6312065	6323309	intron_variant	Osmoregulation
388	LG22	6323212	DJ_018613-T1	Uridine-cytidine kinase	6324255	6334020	upstream_gene_variant	Signal transduction
389	LG22	6323318	DJ_018612-T1	zinc transporter 2	6312065	6323309	upstream_gene_variant	Osmoregulation
390	LG22	6323318	DJ_018613-T1	Uridine-cytidine kinase	6324255	6334020	upstream_gene_variant	Signal transduction
391	LG22	6375106	DJ_018615-T1	protein Mpv17	6362904	6367526	downstream_gene_variant	Growth
392	LG22	6375154	DJ_018615-T1	protein Mpv17	6362904	6367526	downstream_gene_variant	Growth
393	LG22	6375283	DJ_018615-T1	protein Mpv17	6362904	6367526	downstream_gene_variant	Growth
394	LG22	6379351	DJ_018616-T1	N/A	6386509	6388016	upstream_gene_variant	N/A
395	LG22	6379351	DJ_018617-T1	Tripartite motif containing 54	6388549	6405300	downstream_gene_variant	Growth
396	LG22	6379412	DJ_018616-T1	N/A	6386509	6388016	upstream_gene_variant	N/A
397	LG22	6379412	DJ_018617-T1	Tripartite motif containing 54	6388549	6405300	downstream_gene_variant	Growth
398	LG22	6379454	DJ_018616-T1	N/A	6386509	6388016	upstream_gene_variant	N/A

399	LG22	6379454	DJ_018617-T1	Tripartite motif containing 54	6388549	6405300	downstream_gene_variant	Growth
400	LG22	6396778	DJ_018616-T1	N/A	6386509	6388016	downstream_gene_variant	N/A
401	LG22	6396778	DJ_018617-T1	Tripartite motif containing 54	6388549	6405300	intron_variant	Growth
402	LG22	6404947	DJ_018617-T1	Tripartite motif containing 54	6388549	6405300	intron_variant	Growth
403	LG22	6404947	DJ_018618-T1	dnaJ homolog subfamily C member 5G isoform X1	6410248	6422494	downstream_gene_variant	Metabolic process
404	LG22	6422439	DJ_018618-T1	dnaJ homolog subfamily C member 5G isoform X1	6410248	6422494	missense_variant	Metabolic process
405	LG22	6422439	DJ_018619-T1	26S protease regulatory subunit 10B	6425641	6430741	upstream_gene_variant	Signal transduction
406	LG22	6422439	DJ_018620-T1	cell growth regulator with RING finger domain protein 1	6431759	6434713	downstream_gene_variant	Growth
407	LG22	6422467	DJ_018618-T1	dnaJ homolog subfamily C member 5G isoform X1	6410248	6422494	missense_variant	Metabolic process
408	LG22	6422467	DJ_018619-T1	26S protease regulatory subunit 10B	6425641	6430741	upstream_gene_variant	Signal transduction
409	LG22	6422467	DJ_018620-T1	cell growth regulator with RING finger domain protein 1	6431759	6434713	downstream_gene_variant	Growth
410	LG22	6422531	DJ_018618-T1	dnaJ homolog subfamily C member 5G isoform X1	6410248	6422494	upstream_gene_variant	Metabolic process
411	LG22	6422531	DJ_018619-T1	26S protease regulatory subunit 10B	6425641	6430741	upstream_gene_variant	Signal transduction
412	LG22	6422531	DJ_018620-T1	cell growth regulator with RING finger domain protein 1	6431759	6434713	downstream_gene_variant	Growth
413	LG22	6422577	DJ_018618-T1	dnaJ homolog subfamily C member 5G isoform X1	6410248	6422494	upstream_gene_variant	Metabolic process
414	LG22	6422577	DJ_018619-T1	26S protease regulatory subunit 10B	6425641	6430741	upstream_gene_variant	Signal transduction
415	LG22	6422577	DJ_018620-T1	cell growth regulator with RING finger domain protein 1	6431759	6434713	downstream_gene_variant	Growth
416	LG22	6422608	DJ_018618-T1	dnaJ homolog subfamily C member 5G isoform X1	6410248	6422494	upstream_gene_variant	Metabolic process
417	LG22	6422608	DJ_018619-T1	26S protease regulatory subunit 10B	6425641	6430741	upstream_gene_variant	Signal transduction
418	LG22	6422608	DJ_018620-T1	cell growth regulator with RING finger domain protein 1	6431759	6434713	downstream_gene_variant	Growth
419	LG22	6422726	DJ_018618-T1	dnaJ homolog subfamily C member 5G isoform X1	6410248	6422494	upstream_gene_variant	Metabolic process
420	LG22	6422726	DJ_018619-T1	26S protease regulatory subunit 10B	6425641	6430741	upstream_gene_variant	Signal transduction
421	LG22	6422726	DJ_018620-T1	cell growth regulator with RING finger domain protein 1	6431759	6434713	downstream_gene_variant	Growth
422	LG22	6438180	DJ_018619-T1	26S protease regulatory subunit 10B	6425641	6430741	downstream_gene_variant	Signal transduction

423	LG22	6438180	DJ_018620-T1	cell growth regulator with RING finger domain protein 1	6431759	6434713	upstream_gene_variant	Growth
424	LG22	6438180	DJ_018621-T1	Phospholipase DDHD1	6435295	6438253	intron_variant	Signal transduction
425	LG22	6438180	DJ_018622-T1	Phospholipase DDHD1	6438779	6445333	upstream_gene_variant	Signal transduction
426	LG22	6438185	DJ_018619-T1	26S protease regulatory subunit 10B	6425641	6430741	downstream_gene_variant	Signal transduction
427	LG22	6438185	DJ_018620-T1	cell growth regulator with RING finger domain protein 1	6431759	6434713	upstream_gene_variant	Growth
428	LG22	6438185	DJ_018621-T1	Phospholipase DDHD1	6435295	6438253	intron_variant	Signal transduction
429	LG22	6438185	DJ_018622-T1	Phospholipase DDHD1	6438779	6445333	upstream_gene_variant	Signal transduction
430	LG22	6513197	DJ_018625-T1	gap junction delta-2 protein	6508250	6509705	upstream_gene_variant	Visual perception
431	LG22	6513197	DJ_018626-T1	actin, alpha cardiac muscle 1	6511113	6513728	intron_variant	Locomotion
432	LG22	6513216	DJ_018625-T1	gap junction delta-2 protein	6508250	6509705	upstream_gene_variant	Visual perception
433	LG22	6513216	DJ_018626-T1	actin, alpha cardiac muscle 1	6511113	6513728	intron_variant	Locomotion
434	LG22	6521809	DJ_018626-T1	actin, alpha cardiac muscle 1	6511113	6513728	upstream_gene_variant	Locomotion
435	LG22	6521809	DJ_018627-T1	zinc finger protein 770-like	6525221	6528193	downstream_gene_variant	Transcriptional regulation
436	LG22	6524341	DJ_018627-T1	zinc finger protein 770-like	6525221	6528193	downstream_gene_variant	Transcriptional regulation
437	LG22	6552706	DJ_018628-T1	homeobox protein Meis2-like	6550371	6570368	intron_variant	Transcriptional regulation
438	LG22	6552727	DJ_018628-T1	homeobox protein Meis2-like	6550371	6570368	intron_variant	Transcriptional regulation
439	LG22	6552765	DJ_018628-T1	homeobox protein Meis2-like	6550371	6570368	intron_variant	Transcriptional regulation
440	LG22	6556137	DJ_018628-T1	homeobox protein Meis2-like	6550371	6570368	intron_variant	Transcriptional regulation
441	LG22	6627169	DJ_018629-T1	Putative potassium channel subfamily K member 10-like	6616600	6624663	downstream_gene_variant	Osmoregulation
442	LG22	6629957	DJ_018629-T1	Putative potassium channel subfamily K member 10-like	6616600	6624663	downstream_gene_variant	Osmoregulation
443	LG22	6630078	DJ_018629-T1	Putative potassium channel subfamily K member 10-like	6616600	6624663	downstream_gene_variant	Osmoregulation
444	LG22	6630095	DJ_018629-T1	Putative potassium channel subfamily K member 10-like	6616600	6624663	downstream_gene_variant	Osmoregulation
445	LG22	6726992	DJ_018632-T1	glutaredoxin-related protein 5, mitochondrial	6722988	6724130	upstream_gene_variant	Metabolic process

446	LG22	6726992	DJ_018633-T1	nesprin-3 isoform X2	6724847	6734824	intron_variant	Transmembrane transport
447	LG22	6726997	DJ_018632-T1	glutaredoxin-related protein 5, mitochondrial	6722988	6724130	upstream_gene_variant	Metabolic process
448	LG22	6726997	DJ_018633-T1	nesprin-3 isoform X2	6724847	6734824	intron_variant	Transmembrane transport
449	LG22	6731404	DJ_018632-T1	glutaredoxin-related protein 5, mitochondrial	6722988	6724130	upstream_gene_variant	Metabolic process
450	LG22	6731404	DJ_018633-T1	nesprin-3 isoform X2	6724847	6734824	intron_variant	Transmembrane transport
451	LG22	6731404	DJ_018634-T1	nesprin-3 isoform X2	6739732	6740437	upstream_gene_variant	Transmembrane transport
452	LG22	6731408	DJ_018632-T1	glutaredoxin-related protein 5, mitochondrial	6722988	6724130	upstream_gene_variant	Metabolic process
453	LG22	6731408	DJ_018633-T1	nesprin-3 isoform X2	6724847	6734824	intron_variant	Transmembrane transport
454	LG22	6731408	DJ_018634-T1	nesprin-3 isoform X2	6739732	6740437	upstream_gene_variant	Transmembrane transport
455	LG22	6731449	DJ_018632-T1	glutaredoxin-related protein 5, mitochondrial	6722988	6724130	upstream_gene_variant	Metabolic process
456	LG22	6731449	DJ_018633-T1	nesprin-3 isoform X2	6724847	6734824	intron_variant	Transmembrane transport
457	LG22	6731449	DJ_018634-T1	nesprin-3 isoform X2	6739732	6740437	upstream_gene_variant	Transmembrane transport
458	LG22	6731465	DJ_018632-T1	glutaredoxin-related protein 5, mitochondrial	6722988	6724130	upstream_gene_variant	Metabolic process
459	LG22	6731465	DJ_018633-T1	nesprin-3 isoform X2	6724847	6734824	intron_variant	Transmembrane transport
460	LG22	6731465	DJ_018634-T1	nesprin-3 isoform X2	6739732	6740437	upstream_gene_variant	Transmembrane transport
461	LG22	6731470	DJ_018632-T1	glutaredoxin-related protein 5, mitochondrial	6722988	6724130	upstream_gene_variant	Metabolic process
462	LG22	6731470	DJ_018633-T1	nesprin-3 isoform X2	6724847	6734824	intron_variant	Transmembrane transport
463	LG22	6731470	DJ_018634-T1	nesprin-3 isoform X2	6739732	6740437	upstream_gene_variant	Transmembrane transport
464	LG22	6757259	DJ_018635-T1	putative calmin-like	6743778	6749138	downstream_gene_variant	Growth
465	LG22	6757259	DJ_018636-T1	alpha-1-antitrypsin homolog	6755160	6757538	intron_variant	Metabolic process
466	LG22	6780409	DJ_018637-T1	N/A	6770621	6773105	upstream_gene_variant	N/A
467	LG22	6780411	DJ_018637-T1	N/A	6770621	6773105	upstream_gene_variant	N/A
468	LG22	6780429	DJ_018637-T1	N/A	6770621	6773105	upstream_gene_variant	N/A
469	LG22	6854702	DJ_018638-T1	Family with sequence similarity 184 member A	6858616	6862836	upstream_gene_variant	Immune response

470	LG22	6854716	DJ_018638-T1	Family with sequence similarity 184 member A	6858616	6862836	upstream_gene_variant	Immune response
471	LG22	6888498	DJ_018640-T1	Family with sequence similarity 184 member A	6890162	6894363	upstream_gene_variant	Immune response
472	LG22	6888632	DJ_018640-T1	Family with sequence similarity 184 member A	6890162	6894363	upstream_gene_variant	Immune response
473	LG22	6977501	DJ_018644-T1	protein FAM184A-like	6979388	6982631	upstream_gene_variant	Immune response
474	LG22	6977507	DJ_018644-T1	protein FAM184A-like	6979388	6982631	upstream_gene_variant	Immune response
475	LG22	6977577	DJ_018644-T1	protein FAM184A-like	6979388	6982631	upstream_gene_variant	Immune response
476	LG22	6984887	DJ_018644-T1	protein FAM184A-like	6979388	6982631	downstream_gene_variant	Immune response
477	LG22	6984887	DJ_018645-T1	DNA helicase MCM9	6993044	7021480	upstream_gene_variant	Growth
478	LG22	6984951	DJ_018644-T1	protein FAM184A-like	6979388	6982631	downstream_gene_variant	Immune response
479	LG22	6984951	DJ_018645-T1	DNA helicase MCM9	6993044	7021480	upstream_gene_variant	Growth
480	LG22	7003186	DJ_018645-T1	DNA helicase MCM9	6993044	7021480	intron_variant	Growth
481	LG22	7004581	DJ_018645-T1	DNA helicase MCM9	6993044	7021480	intron_variant	Growth
482	LG22	7004617	DJ_018645-T1	DNA helicase MCM9	6993044	7021480	intron_variant	Growth
483	LG22	7014302	DJ_018645-T1	DNA helicase MCM9	6993044	7021480	intron_variant	Growth
484	LG22	7028591	DJ_018645-T1	DNA helicase MCM9	6993044	7021480	downstream_gene_variant	Growth
485	LG22	7028591	DJ_018646-T1	centrosomal protein of 85 kDa-like isoform X1	7034981	7036127	upstream_gene_variant	Growth
486	LG22	7029173	DJ_018645-T1	DNA helicase MCM9	6993044	7021480	downstream_gene_variant	Growth
487	LG22	7029173	DJ_018646-T1	centrosomal protein of 85 kDa-like isoform X1	7034981	7036127	upstream_gene_variant	Growth
488	LG22	7029174	DJ_018645-T1	DNA helicase MCM9	6993044	7021480	downstream_gene_variant	Growth
489	LG22	7029174	DJ_018646-T1	centrosomal protein of 85 kDa-like isoform X1	7034981	7036127	upstream_gene_variant	Growth
490	LG22	7029201	DJ_018645-T1	DNA helicase MCM9	6993044	7021480	downstream_gene_variant	Growth
491	LG22	7029201	DJ_018646-T1	centrosomal protein of 85 kDa-like isoform X1	7034981	7036127	upstream_gene_variant	Growth
492	LG22	7029292	DJ_018645-T1	DNA helicase MCM9	6993044	7021480	downstream_gene_variant	Growth
493	LG22	7029292	DJ_018646-T1	centrosomal protein of 85 kDa-like isoform X1	7034981	7036127	upstream_gene_variant	Growth
494	LG22	15814278	DJ_018933-T1	reticulon-1-like isoform X1	15809776	15823381	intron_variant	Growth
495	LG22	15814313	DJ_018933-T1	reticulon-1-like isoform X1	15809776	15823381	intron_variant	Growth
496	LG22	15816473	DJ_018933-T1	reticulon-1-like isoform X1	15809776	15823381	intron_variant	Growth
497	LG22	15824416	DJ_018933-T1	reticulon-1-like isoform X1	15809776	15823381	downstream_gene_variant	Growth

498	LG22	15918083	DJ_018937-T1	Apoptosis-stimulating of p53 protein 1	15918997	15950005	upstream_gene_variant	Transcriptional regulation
499	LG22	15918198	DJ_018937-T1	Apoptosis-stimulating of p53 protein 1	15918997	15950005	upstream_gene_variant	Transcriptional regulation
500	LG22	15918205	DJ_018937-T1	Apoptosis-stimulating of p53 protein 1	15918997	15950005	upstream_gene_variant	Transcriptional regulation
501	LG22	15918213	DJ_018937-T1	Apoptosis-stimulating of p53 protein 1	15918997	15950005	upstream_gene_variant	Transcriptional regulation
502	LG22	15918244	DJ_018937-T1	Apoptosis-stimulating of p53 protein 1	15918997	15950005	upstream_gene_variant	Transcriptional regulation
503	LG22	15952353	DJ_018937-T1	Apoptosis-stimulating of p53 protein 1	15918997	15950005	downstream_gene_variant	Transcriptional regulation
504	LG22	15979967	DJ_018938-T1	Apoptosis-stimulating of p53 protein 1	15974291	15978364	downstream_gene_variant	Transcriptional regulation
505	LG22	15982511	DJ_018938-T1	Apoptosis-stimulating of p53 protein 1	15974291	15978364	downstream_gene_variant	Transcriptional regulation
506	LG22	15982562	DJ_018938-T1	Apoptosis-stimulating of p53 protein 1	15974291	15978364	downstream_gene_variant	Transcriptional regulation
507	LG22	15982577	DJ_018938-T1	Apoptosis-stimulating of p53 protein 1	15974291	15978364	downstream_gene_variant	Transcriptional regulation
508	LG22	15982580	DJ_018938-T1	Apoptosis-stimulating of p53 protein 1	15974291	15978364	downstream_gene_variant	Transcriptional regulation
509	LG22	15982647	DJ_018938-T1	Apoptosis-stimulating of p53 protein 1	15974291	15978364	downstream_gene_variant	Transcriptional regulation
510	LG22	15982655	DJ_018938-T1	Apoptosis-stimulating of p53 protein 1	15974291	15978364	downstream_gene_variant	Transcriptional regulation
511	LG22	15987970	DJ_018938-T1	Apoptosis-stimulating of p53 protein 1	15974291	15978364	downstream_gene_variant	Transcriptional regulation
512	LG22	15988010	DJ_018938-T1	Apoptosis-stimulating of p53 protein 1	15974291	15978364	downstream_gene_variant	Transcriptional regulation
513	LG22	15988042	DJ_018938-T1	Apoptosis-stimulating of p53 protein 1	15974291	15978364	downstream_gene_variant	Transcriptional regulation
514	LG22	15988096	DJ_018938-T1	Apoptosis-stimulating of p53 protein 1	15974291	15978364	downstream_gene_variant	Transcriptional regulation
515	LG22	15988123	DJ_018938-T1	Apoptosis-stimulating of p53 protein 1	15974291	15978364	downstream_gene_variant	Transcriptional regulation
516	LG22	15988142	DJ_018938-T1	Apoptosis-stimulating of p53 protein 1	15974291	15978364	downstream_gene_variant	Transcriptional regulation
517	LG22	15988166	DJ_018938-T1	Apoptosis-stimulating of p53 protein 1	15974291	15978364	downstream_gene_variant	Transcriptional regulation
518	LG22	16066466	DJ_018940-T1	Dishevelled associated activator of morphogenesis 1b	16054777	16090674	synonymous_variant	Signal transduction
519	LG22	16066567	DJ_018940-T1	Dishevelled associated activator of morphogenesis 1b	16054777	16090674	intron_variant	Signal transduction

520	LG22	16079989	DJ_018940-T1	Dishevelled associated activator of morphogenesis 1b	16054777	16090674	intron_variant	Signal transduction
521	LG22	16080050	DJ_018940-T1	Dishevelled associated activator of morphogenesis 1b	16054777	16090674	intron_variant	Signal transduction
522	LG22	16108769	DJ_018942-T1	nesprin-2-like isoform X1	16108667	16122865	intron_variant	Metabolic process
523	LG22	16108779	DJ_018942-T1	nesprin-2-like isoform X1	16108667	16122865	intron_variant	Metabolic process
524	LG22	16108784	DJ_018942-T1	nesprin-2-like isoform X1	16108667	16122865	intron_variant	Metabolic process
525	LG22	16108794	DJ_018942-T1	nesprin-2-like isoform X1	16108667	16122865	intron_variant	Metabolic process
526	LG22	16108806	DJ_018942-T1	nesprin-2-like isoform X1	16108667	16122865	intron_variant	Metabolic process
527	LG22	16108809	DJ_018942-T1	nesprin-2-like isoform X1	16108667	16122865	intron_variant	Metabolic process
528	LG22	16108812	DJ_018942-T1	nesprin-2-like isoform X1	16108667	16122865	intron_variant	Metabolic process
529	LG22	16108836	DJ_018942-T1	nesprin-2-like isoform X1	16108667	16122865	intron_variant	Metabolic process
530	LG22	16108844	DJ_018942-T1	nesprin-2-like isoform X1	16108667	16122865	intron_variant	Metabolic process
531	LG22	16108853	DJ_018942-T1	nesprin-2-like isoform X1	16108667	16122865	intron_variant	Metabolic process
532	LG22	16108860	DJ_018942-T1	nesprin-2-like isoform X1	16108667	16122865	intron_variant	Metabolic process
533	LG22	16109015	DJ_018942-T1	nesprin-2-like isoform X1	16108667	16122865	intron_variant	Metabolic process
534	LG22	16109029	DJ_018942-T1	nesprin-2-like isoform X1	16108667	16122865	intron_variant	Metabolic process
535	LG22	16109036	DJ_018942-T1	nesprin-2-like isoform X1	16108667	16122865	intron_variant	Metabolic process
536	LG22	16109051	DJ_018942-T1	nesprin-2-like isoform X1	16108667	16122865	intron_variant	Metabolic process
537	LG22	16109103	DJ_018942-T1	nesprin-2-like isoform X1	16108667	16122865	intron_variant	Metabolic process
538	LG22	16109124	DJ_018942-T1	nesprin-2-like isoform X1	16108667	16122865	intron_variant	Metabolic process
539	LG22	16272612	DJ_018948-T1	estrogen receptor beta	16255898	16268410	upstream_gene_variant	Signal transduction
540	LG22	16429363	DJ_018950-T1	protein jagged-2-like isoform X2	16404437	16431155	intron_variant	Growth
541	LG22	16727961	DJ_018958-T1	transcription factor IIIB 90 kDa subunit	16736006	16740384	downstream_gene_variant	Transcriptional regulation

542	LG22	16771379	DJ_018961-T1	transcription factor IIIB 90 kDa subunit-like	16751617	16765626	upstream_gene_variant	Transcriptional regulation
543	LG22	16771379	DJ_018962-T1	uncharacterized protein LOC105897785	16765671	16776213	intron_variant	N/A
544	LG22	16771379	DJ_018963-T1	SUN domain-containing protein 2-like	16777387	16780847	downstream_gene_variant	Growth
545	LG22	16771425	DJ_018961-T1	transcription factor IIIB 90 kDa subunit-like	16751617	16765626	upstream_gene_variant	Transcriptional regulation
546	LG22	16771425	DJ_018962-T1	uncharacterized protein LOC105897785	16765671	16776213	intron_variant	N/A
547	LG22	16771425	DJ_018963-T1	SUN domain-containing protein 2-like	16777387	16780847	downstream_gene_variant	Growth
548	LG22	16771433	DJ_018961-T1	transcription factor IIIB 90 kDa subunit-like	16751617	16765626	upstream_gene_variant	Transcriptional regulation
549	LG22	16771433	DJ_018962-T1	uncharacterized protein LOC105897785	16765671	16776213	intron_variant	N/A
550	LG22	16771433	DJ_018963-T1	SUN domain-containing protein 2-like	16777387	16780847	downstream_gene_variant	Growth
551	LG22	16771495	DJ_018961-T1	transcription factor IIIB 90 kDa subunit-like	16751617	16765626	upstream_gene_variant	Transcriptional regulation
552	LG22	16771495	DJ_018962-T1	uncharacterized protein LOC105897785	16765671	16776213	missense_variant	N/A
553	LG22	16771495	DJ_018963-T1	SUN domain-containing protein 2-like	16777387	16780847	downstream_gene_variant	Growth
554	LG22	16771532	DJ_018961-T1	transcription factor IIIB 90 kDa subunit-like	16751617	16765626	upstream_gene_variant	Transcriptional regulation
555	LG22	16771532	DJ_018962-T1	uncharacterized protein LOC105897785	16765671	16776213	synonymous_variant	N/A
556	LG22	16771532	DJ_018963-T1	SUN domain-containing protein 2-like	16777387	16780847	downstream_gene_variant	Growth
557	LG22	16778808	DJ_018962-T1	uncharacterized protein LOC105897785	16765671	16776213	downstream_gene_variant	N/A
558	LG22	16778808	DJ_018963-T1	SUN domain-containing protein 2-like	16777387	16780847	intron_variant	Growth
559	LG22	16778808	DJ_018964-T1	DNA repair protein XRCC3	16783468	16786900	upstream_gene_variant	Growth
560	LG22	16789602	DJ_018963-T1	SUN domain-containing protein 2-like	16777387	16780847	upstream_gene_variant	Growth
561	LG22	16789602	DJ_018964-T1	DNA repair protein XRCC3	16783468	16786900	downstream_gene_variant	Growth
562	LG22	16844715	DJ_018965-T1	creatine kinase, muscle a isoform X2	16851111	16855472	upstream_gene_variant	Metabolic process
563	LG22	16844732	DJ_018965-T1	creatine kinase, muscle a isoform X2	16851111	16855472	upstream_gene_variant	Metabolic process
564	LG22	16844751	DJ_018965-T1	creatine kinase, muscle a isoform X2	16851111	16855472	upstream_gene_variant	Metabolic process

565	LG22	16962143	DJ_018971-T1	MAP/microtubule affinity-regulating kinase 3 isoform X2	16947852	16955319	upstream_gene_variant	Signal transduction
566	LG22	16962143	DJ_018972-T1	Chloride intracellular channel protein	16966455	16976872	upstream_gene_variant	Osmoregulation
567	LG22	16964917	DJ_018971-T1	MAP/microtubule affinity-regulating kinase 3 isoform X2	16947852	16955319	upstream_gene_variant	Signal transduction
568	LG22	16964917	DJ_018972-T1	Chloride intracellular channel protein	16966455	16976872	upstream_gene_variant	Osmoregulation
569	LG22	16964967	DJ_018971-T1	MAP/microtubule affinity-regulating kinase 3 isoform X2	16947852	16955319	upstream_gene_variant	Signal transduction
570	LG22	16964967	DJ_018972-T1	Chloride intracellular channel protein	16966455	16976872	upstream_gene_variant	Osmoregulation
571	LG22	16965063	DJ_018971-T1	MAP/microtubule affinity-regulating kinase 3 isoform X2	16947852	16955319	upstream_gene_variant	Signal transduction
572	LG22	16965063	DJ_018972-T1	Chloride intracellular channel protein	16966455	16976872	upstream_gene_variant	Osmoregulation
573	LG22	16965068	DJ_018971-T1	MAP/microtubule affinity-regulating kinase 3 isoform X2	16947852	16955319	upstream_gene_variant	Signal transduction
574	LG22	16965068	DJ_018972-T1	Chloride intracellular channel protein	16966455	16976872	upstream_gene_variant	Osmoregulation
575	LG22	16965099	DJ_018971-T1	MAP/microtubule affinity-regulating kinase 3 isoform X2	16947852	16955319	upstream_gene_variant	Signal transduction
576	LG22	16965099	DJ_018972-T1	Chloride intracellular channel protein	16966455	16976872	upstream_gene_variant	Osmoregulation
577	LG22	16965130	DJ_018971-T1	MAP/microtubule affinity-regulating kinase 3 isoform X2	16947852	16955319	upstream_gene_variant	Signal transduction
578	LG22	16965130	DJ_018972-T1	Chloride intracellular channel protein	16966455	16976872	upstream_gene_variant	Osmoregulation
579	LG22	16969575	DJ_018972-T1	Chloride intracellular channel protein	16966455	16976872	intron_variant	Osmoregulation
580	LG22	16969692	DJ_018972-T1	Chloride intracellular channel protein	16966455	16976872	intron_variant	Osmoregulation
581	LG22	16969715	DJ_018972-T1	Chloride intracellular channel protein	16966455	16976872	intron_variant	Osmoregulation
582	LG22	16969729	DJ_018972-T1	Chloride intracellular channel protein	16966455	16976872	intron_variant	Osmoregulation
583	LG22	16969737	DJ_018972-T1	Chloride intracellular channel protein	16966455	16976872	intron_variant	Osmoregulation
584	LG22	16969740	DJ_018972-T1	Chloride intracellular channel protein	16966455	16976872	intron_variant	Osmoregulation
585	LG22	16969759	DJ_018972-T1	Chloride intracellular channel protein	16966455	16976872	intron_variant	Osmoregulation
586	LG22	16978114	DJ_018972-T1	Chloride intracellular channel protein	16966455	16976872	downstream_gene_variant	Osmoregulation

587	LG22	17007223	DJ_018974-T1	Runt-related transcription factor	16997890	17018675	intron_variant	Transcriptional regulation
588	LG22	17016108	DJ_018974-T1	Runt-related transcription factor	16997890	17018675	intron_variant	Transcriptional regulation
589	LG22	17016188	DJ_018974-T1	Runt-related transcription factor	16997890	17018675	intron_variant	Transcriptional regulation
590	LG22	17016220	DJ_018974-T1	Runt-related transcription factor	16997890	17018675	intron_variant	Transcriptional regulation
591	LG22	17016230	DJ_018974-T1	Runt-related transcription factor	16997890	17018675	intron_variant	Transcriptional regulation
592	LG22	17016307	DJ_018974-T1	Runt-related transcription factor	16997890	17018675	intron_variant	Transcriptional regulation
593	LG22	17016355	DJ_018974-T1	Runt-related transcription factor	16997890	17018675	intron_variant	Transcriptional regulation
594	LG22	17016396	DJ_018974-T1	Runt-related transcription factor	16997890	17018675	intron_variant	Transcriptional regulation
595	LG22	17016457	DJ_018974-T1	Runt-related transcription factor	16997890	17018675	intron_variant	Transcriptional regulation
596	LG22	17038387	DJ_018975-T1	N/A	17039709	17044735	downstream_gene_variant	N/A
597	LG22	17038552	DJ_018975-T1	N/A	17039709	17044735	downstream_gene_variant	N/A
598	LG22	17038564	DJ_018975-T1	N/A	17039709	17044735	downstream_gene_variant	N/A
599	LG22	17038573	DJ_018975-T1	N/A	17039709	17044735	downstream_gene_variant	N/A
600	LG22	17038591	DJ_018975-T1	N/A	17039709	17044735	downstream_gene_variant	N/A
601	LG22	17157093	DJ_018979-T1	C-C motif chemokine	17166711	17167328	upstream_gene_variant	Immune response
602	LG22	17157150	DJ_018979-T1	C-C motif chemokine	17166711	17167328	upstream_gene_variant	Immune response
603	LG22	17157218	DJ_018979-T1	C-C motif chemokine	17166711	17167328	upstream_gene_variant	Immune response
604	LG22	17157224	DJ_018979-T1	C-C motif chemokine	17166711	17167328	upstream_gene_variant	Immune response
605	LG22	17157225	DJ_018979-T1	C-C motif chemokine	17166711	17167328	upstream_gene_variant	Immune response
606	LG22	17190761	DJ_018980-T1	C-C motif chemokine	17178110	17183324	downstream_gene_variant	Immune response
607	LG22	17190767	DJ_018980-T1	C-C motif chemokine	17178110	17183324	downstream_gene_variant	Immune response
608	LG22	17190886	DJ_018980-T1	C-C motif chemokine	17178110	17183324	downstream_gene_variant	Immune response
609	LG22	17229888	DJ_018981-T1	inositol-trisphosphate 3-kinase B-like	17237666	17243121	downstream_gene_variant	Signal transduction
610	LG22	17229895	DJ_018981-T1	inositol-trisphosphate 3-kinase B-like	17237666	17243121	downstream_gene_variant	Signal transduction

611	LG22	17229988	DJ_018981-T1	inositol-trisphosphate 3-kinase B-like	17237666	17243121	downstream_gene_variant	Signal transduction
612	LG22	17230007	DJ_018981-T1	inositol-trisphosphate 3-kinase B-like	17237666	17243121	downstream_gene_variant	Signal transduction
613	LG22	17246776	DJ_018981-T1	inositol-trisphosphate 3-kinase B-like	17237666	17243121	upstream_gene_variant	Signal transduction
614	LG22	17246776	DJ_018982-T1	inositol-trisphosphate 3-kinase B-like	17247581	17258122	upstream_gene_variant	Signal transduction
615	LG22	17256932	DJ_018982-T1	inositol-trisphosphate 3-kinase B-like	17247581	17258122	intron_variant	Signal transduction
616	LG22	17256962	DJ_018982-T1	inositol-trisphosphate 3-kinase B-like	17247581	17258122	intron_variant	Signal transduction
617	LG22	17257187	DJ_018982-T1	inositol-trisphosphate 3-kinase B-like	17247581	17258122	intron_variant	Signal transduction
618	LG22	17273869	DJ_018983-T1	inositol-trisphosphate 3-kinase B-like	17270771	17273568	upstream_gene_variant	Signal transduction
619	LG22	17276224	DJ_018983-T1	inositol-trisphosphate 3-kinase B-like	17270771	17273568	upstream_gene_variant	Signal transduction
620	LG22	17276230	DJ_018983-T1	inositol-trisphosphate 3-kinase B-like	17270771	17273568	upstream_gene_variant	Signal transduction
621	LG22	17276240	DJ_018983-T1	inositol-trisphosphate 3-kinase B-like	17270771	17273568	upstream_gene_variant	Signal transduction
622	LG22	17280560	DJ_018983-T1	inositol-trisphosphate 3-kinase B-like	17270771	17273568	upstream_gene_variant	Signal transduction
623	LG22	17280560	DJ_018984-T1	atypical kinase COQ8A, mitochondrial	17286292	17311328	upstream_gene_variant	Metabolic process
624	LG22	17280704	DJ_018983-T1	inositol-trisphosphate 3-kinase B-like	17270771	17273568	upstream_gene_variant	Signal transduction
625	LG22	17280704	DJ_018984-T1	atypical kinase COQ8A, mitochondrial	17286292	17311328	upstream_gene_variant	Metabolic process
626	LG22	18448629	DJ_019024-T1	uncharacterized protein LOC116065420	18436952	18443332	upstream_gene_variant	N/A
627	LG22	18448629	DJ_019025-T1	sorting nexin-9-like isoform X2	18448632	18462632	upstream_gene_variant	Metabolic process
628	LG22	18448676	DJ_019024-T1	uncharacterized protein LOC116065420	18436952	18443332	upstream_gene_variant	N/A
629	LG22	18448676	DJ_019025-T1	sorting nexin-9-like isoform X2	18448632	18462632	upstream_gene_variant	Metabolic process
630	LG22	18454744	DJ_019025-T1	sorting nexin-9-like isoform X2	18448632	18462632	intron_variant	Metabolic process
631	LG22	18454744	DJ_019026-T1	sorting nexin-9-like isoform X2	18463006	18466946	upstream_gene_variant	Metabolic process
632	LG22	18480800	DJ_019027-T1	angiopoietin-related protein 7-like	18471512	18475976	upstream_gene_variant	Growth
633	LG22	18480800	DJ_019028-T1	pro-opiomelanocortin-1-like	18480960	18483797	upstream_gene_variant	Growth

634	LG22	18480881	DJ_019027-T1	angiopoietin-related protein 7-like	18471512	18475976	upstream_gene_variant	Growth
635	LG22	18480881	DJ_019028-T1	pro-opiomelanocortin-1-like	18480960	18483797	upstream_gene_variant	Growth
636	LG22	18488887	DJ_019028-T1	pro-opiomelanocortin-1-like	18480960	18483797	downstream_gene_variant	Growth
637	LG22	18488887	DJ_019029-T1	protein EFR3 homolog B isoform X1	18492813	18511615	downstream_gene_variant	Metabolic process
638	LG22	18532411	DJ_019030-T1	protein EFR3 homolog B isoform X1	18511938	18523982	upstream_gene_variant	Metabolic process
639	LG22	18532411	DJ_019031-T1	adenylate cyclase type 8-like isoform X1	18533282	18541073	upstream_gene_variant	Signal transduction
640	LG22	18532498	DJ_019030-T1	protein EFR3 homolog B isoform X1	18511938	18523982	upstream_gene_variant	Metabolic process
641	LG22	18532498	DJ_019031-T1	adenylate cyclase type 8-like isoform X1	18533282	18541073	upstream_gene_variant	Signal transduction
642	LG22	18532528	DJ_019030-T1	protein EFR3 homolog B isoform X1	18511938	18523982	upstream_gene_variant	Metabolic process
643	LG22	18532528	DJ_019031-T1	adenylate cyclase type 8-like isoform X1	18533282	18541073	upstream_gene_variant	Signal transduction
644	LG22	18557439	DJ_019033-T1	pollen-specific leucine-rich repeat extensin-like protein 2	18549416	18554950	downstream_gene_variant	Growth
645	LG22	18557439	DJ_019034-T1	adenylate cyclase type 8-like isoform X1	18556732	18562363	intron_variant	Signal transduction
646	LG22	18557463	DJ_019033-T1	pollen-specific leucine-rich repeat extensin-like protein 2	18549416	18554950	downstream_gene_variant	Growth
647	LG22	18557463	DJ_019034-T1	adenylate cyclase type 8-like isoform X1	18556732	18562363	intron_variant	Signal transduction
648	LG22	18557481	DJ_019033-T1	pollen-specific leucine-rich repeat extensin-like protein 2	18549416	18554950	downstream_gene_variant	Growth
649	LG22	18557481	DJ_019034-T1	adenylate cyclase type 8-like isoform X1	18556732	18562363	intron_variant	Signal transduction
650	LG22	18557574	DJ_019033-T1	pollen-specific leucine-rich repeat extensin-like protein 2	18549416	18554950	downstream_gene_variant	Growth
651	LG22	18557574	DJ_019034-T1	adenylate cyclase type 8-like isoform X1	18556732	18562363	intron_variant	Signal transduction
652	LG22	18656425	DJ_019038-T1	Tyrosine-protein phosphatase non-receptor type 14	18629890	18654485	downstream_gene_variant	Transcriptional regulation
653	LG22	18729118	DJ_019041-T1	cGMP-dependent protein kinase 2-like	18717905	18729525	synonymous_variant	Signal transduction
654	LG22	18729118	DJ_019042-T1	cGMP-dependent protein kinase 2-like	18732505	18742151	downstream_gene_variant	Signal transduction
655	LG22	18771890	DJ_019043-T1	protein ABHD1-like isoform X1	18756210	18763447	upstream_gene_variant	Metabolic process
656	LG22	18771890	DJ_019044-T1	interferon-induced protein 44-like	18767406	18773566	intron_variant	Immune response

657	LG22	18771890	DJ_019045-T1	cathepsin B	18774924	18776363	downstream_gene_variant	Signal transduction
658	LG22	18771890	DJ_019046-T1	squalene synthase isoform X1	18776366	18791252	downstream_gene_variant	Metabolic process
659	LG22	18833273	DJ_019049-T1	N/A	18832471	18833791	intron_variant	N/A
660	LG22	18880915	DJ_019051-T1	histidine triad nucleotide-binding protein 3-like isoform X1	18869032	18873303	downstream_gene_variant	Metabolic process
661	LG22	18880915	DJ_019052-T1	syntaxin-11-like	18880272	18885008	intron_variant	Metabolic process
662	LG22	19016049	DJ_019057-T1	zinc finger and BTB domain-containing protein 2	19011882	19016692	intron_variant	Transcriptional regulation
663	LG22	19016049	DJ_019058-T1	estrogen receptor beta-like	19020937	19042252	upstream_gene_variant	Signal transduction
664	LG22	19030719	DJ_019058-T1	estrogen receptor beta-like	19020937	19042252	downstream_gene_variant	Signal transduction
665	LG22	19030904	DJ_019058-T1	estrogen receptor beta-like	19020937	19042252	synonymous_variant	Signal transduction
666	LG22	19185459	DJ_019065-T1	Protein SERAC1	19178043	19180478	downstream_gene_variant	Metabolic process
667	LG22	19185489	DJ_019065-T1	Protein SERAC1	19178043	19180478	downstream_gene_variant	Metabolic process
668	LG22	19185516	DJ_019065-T1	Protein SERAC1	19178043	19180478	downstream_gene_variant	Metabolic process
669	LG22	19232279	DJ_019066-T1	papilin-like	19227801	19235378	intron_variant	Signal transduction
670	LG22	19232279	DJ_019067-T1	N/A	19239875	19241472	upstream_gene_variant	N/A
671	LG22	19253267	DJ_019068-T1	papilin-like	19244104	19247377	upstream_gene_variant	Signal transduction
672	LG22	19253267	DJ_019069-T1	dynein regulatory complex protein 1	19259233	19269898	upstream_gene_variant	Growth
673	LG22	19253298	DJ_019068-T1	papilin-like	19244104	19247377	upstream_gene_variant	Signal transduction
674	LG22	19253298	DJ_019069-T1	dynein regulatory complex protein 1	19259233	19269898	upstream_gene_variant	Growth
675	LG22	19253338	DJ_019068-T1	papilin-like	19244104	19247377	upstream_gene_variant	Signal transduction
676	LG22	19253338	DJ_019069-T1	dynein regulatory complex protein 1	19259233	19269898	upstream_gene_variant	Growth
677	LG22	19270390	DJ_019069-T1	dynein regulatory complex protein 1	19259233	19269898	downstream_gene_variant	Growth
678	LG22	19271252	DJ_019069-T1	dynein regulatory complex protein 1	19259233	19269898	downstream_gene_variant	Growth
679	LG22	19457540	DJ_019077-T1	polyphosphoinositide phosphatase	19466310	19479946	downstream_gene_variant	Locomotion
680	LG22	19473010	DJ_019077-T1	polyphosphoinositide phosphatase	19466310	19479946	intron_variant	Locomotion

681	LG22	19483530	DJ_019077-T1	polyphosphoinositide phosphatase	19466310	19479946	upstream_gene_variant	Locomotion
682	LG22	19483530	DJ_019078-T1	polyphosphoinositide phosphatase	19491889	19506349	downstream_gene_variant	Locomotion
683	LG22	19495877	DJ_019078-T1	polyphosphoinositide phosphatase	19491889	19506349	intron_variant	Locomotion
684	LG22	19506065	DJ_019078-T1	polyphosphoinositide phosphatase	19491889	19506349	intron_variant	Locomotion
685	LG22	19506065	DJ_019079-T1	polyphosphoinositide phosphatase	19506645	19526833	downstream_gene_variant	Locomotion
686	LG22	19506068	DJ_019078-T1	polyphosphoinositide phosphatase	19491889	19506349	intron_variant	Locomotion
687	LG22	19506068	DJ_019079-T1	polyphosphoinositide phosphatase	19506645	19526833	downstream_gene_variant	Locomotion
688	LG22	19506104	DJ_019078-T1	polyphosphoinositide phosphatase	19491889	19506349	intron_variant	Locomotion
689	LG22	19506104	DJ_019079-T1	polyphosphoinositide phosphatase	19506645	19526833	downstream_gene_variant	Locomotion
690	LG22	19529764	DJ_019079-T1	polyphosphoinositide phosphatase	19506645	19526833	downstream_gene_variant	Locomotion
691	LG22	19529764	DJ_019080-T1	adenylate kinase 9 isoform X1	19527802	19542728	intron_variant	Metabolic process
692	LG22	19529832	DJ_019079-T1	polyphosphoinositide phosphatase	19506645	19526833	upstream_gene_variant	Locomotion
693	LG22	19529832	DJ_019080-T1	adenylate kinase 9 isoform X1	19527802	19542728	intron_variant	Metabolic process
694	LG22	19529905	DJ_019079-T1	polyphosphoinositide phosphatase	19506645	19526833	upstream_gene_variant	Locomotion
695	LG22	19529905	DJ_019080-T1	adenylate kinase 9 isoform X1	19527802	19542728	intron_variant	Metabolic process
696	LG22	19529973	DJ_019079-T1	polyphosphoinositide phosphatase	19506645	19526833	upstream_gene_variant	Locomotion
697	LG22	19529973	DJ_019080-T1	adenylate kinase 9 isoform X1	19527802	19542728	intron_variant	Metabolic process
698	LG22	19529985	DJ_019079-T1	polyphosphoinositide phosphatase	19506645	19526833	upstream_gene_variant	Locomotion
699	LG22	19529985	DJ_019080-T1	adenylate kinase 9 isoform X1	19527802	19542728	intron_variant	Metabolic process
700	LG22	19554550	DJ_019082-T1	polyphosphoinositide phosphatase	19546696	19557632	upstream_gene_variant	Locomotion
701	LG22	19554550	DJ_019083-T1	adenylate kinase 9 isoform X1	19558117	19560736	intron_variant	Metabolic process
702	LG22	19559487	DJ_019082-T1	adenylate kinase 9 isoform X2	19546696	19557632	downstream_gene_variant	Metabolic process
703	LG22	19559487	DJ_019083-T1	probable inactive peptidyl-prolyl cis-trans isomerase-like 6 isoform X1	19558117	19560736	intron_variant	Metabolic process
704	LG22	19559487	DJ_019084-T1	sialomucin core protein 24 isoform X2	19568292	19570528	upstream_gene_variant	Immune response
705	LG22	19559491	DJ_019082-T1	adenylate kinase 9 isoform X2	19546696	19557632	downstream_gene_variant	Metabolic process
706	LG22	19559491	DJ_019083-T1	probable inactive peptidyl-prolyl cis-trans isomerase-like 6 isoform X1	19558117	19560736	intron_variant	Metabolic process
707	LG22	19559491	DJ_019084-T1	sialomucin core protein 24 isoform X2	19568292	19570528	upstream_gene_variant	Immune response

708	LG22	19559515	DJ_019082-T1	adenylate kinase 9 isoform X2	19546696	19557632	downstream_gene_variant	Metabolic process
709	LG22	19559515	DJ_019083-T1	probable inactive peptidyl-prolyl cis-trans isomerase-like 6 isoform X1	19558117	19560736	intron_variant	Metabolic process
710	LG22	19559515	DJ_019084-T1	sialomucin core protein 24 isoform X2	19568292	19570528	upstream_gene_variant	Immune response
711	LG22	19559553	DJ_019082-T1	adenylate kinase 9 isoform X2	19546696	19557632	downstream_gene_variant	Metabolic process
712	LG22	19559553	DJ_019083-T1	probable inactive peptidyl-prolyl cis-trans isomerase-like 6 isoform X1	19558117	19560736	intron_variant	Metabolic process
713	LG22	19559553	DJ_019084-T1	sialomucin core protein 24 isoform X2	19568292	19570528	upstream_gene_variant	Immune response
714	LG22	19598192	DJ_019085-T1	centrosomal protein cep57l1-like isoform X1	19579722	19588798	upstream_gene_variant	Metabolic process
715	LG22	19598192	DJ_019086-T1	putative peptidyl-tRNA hydrolase PTRHD1	19590439	19590947	upstream_gene_variant	Metabolic process
716	LG22	19598192	DJ_019087-T1	protein ripply2-like	19593032	19593727	upstream_gene_variant	Transcriptional regulation
717	LG22	19598192	DJ_019088-T1	protein ripply2-like	19599332	19600032	downstream_gene_variant	Transcriptional regulation
718	LG22	19598192	DJ_019089-T1	5-hydroxytryptamine receptor 3A-like	19602673	19610176	upstream_gene_variant	Signal transduction
719	LG22	19598306	DJ_019085-T1	centrosomal protein cep57l1-like isoform X1	19579722	19588798	upstream_gene_variant	Metabolic process
720	LG22	19598306	DJ_019086-T1	putative peptidyl-tRNA hydrolase PTRHD1	19590439	19590947	upstream_gene_variant	Metabolic process
721	LG22	19598306	DJ_019087-T1	protein ripply2-like	19593032	19593727	upstream_gene_variant	Transcriptional regulation
722	LG22	19598306	DJ_019088-T1	protein ripply2-like	19599332	19600032	downstream_gene_variant	Transcriptional regulation
723	LG22	19598306	DJ_019089-T1	5-hydroxytryptamine receptor 3A-like	19602673	19610176	upstream_gene_variant	Signal transduction
724	LG22	19598315	DJ_019085-T1	centrosomal protein cep57l1-like isoform X1	19579722	19588798	upstream_gene_variant	Metabolic process
725	LG22	19598315	DJ_019086-T1	putative peptidyl-tRNA hydrolase PTRHD1	19590439	19590947	upstream_gene_variant	Metabolic process
726	LG22	19598315	DJ_019087-T1	protein ripply2-like	19593032	19593727	upstream_gene_variant	Transcriptional regulation
727	LG22	19598315	DJ_019088-T1	protein ripply2-like	19599332	19600032	downstream_gene_variant	Transcriptional regulation
728	LG22	19598315	DJ_019089-T1	5-hydroxytryptamine receptor 3A-like	19602673	19610176	upstream_gene_variant	Signal transduction

729	LG22	19598321	DJ_019085-T1	centrosomal protein cep57l1-like isoform X1	19579722	19588798	upstream_gene_variant	Metabolic process
730	LG22	19598321	DJ_019086-T1	putative peptidyl-tRNA hydrolase PTRHD1	19590439	19590947	upstream_gene_variant	Metabolic process
731	LG22	19598321	DJ_019087-T1	protein ripply2-like	19593032	19593727	upstream_gene_variant	Transcriptional regulation
732	LG22	19598321	DJ_019088-T1	protein ripply2-like	19599332	19600032	downstream_gene_variant	Transcriptional regulation
733	LG22	19598321	DJ_019089-T1	5-hydroxytryptamine receptor 3A-like	19602673	19610176	upstream_gene_variant	Signal transduction
734	LG22	19598328	DJ_019085-T1	centrosomal protein cep57l1-like isoform X1	19579722	19588798	upstream_gene_variant	Metabolic process
735	LG22	19598328	DJ_019086-T1	putative peptidyl-tRNA hydrolase PTRHD1	19590439	19590947	upstream_gene_variant	Metabolic process
736	LG22	19598328	DJ_019087-T1	protein ripply2-like	19593032	19593727	upstream_gene_variant	Transcriptional regulation
737	LG22	19598328	DJ_019088-T1	protein ripply2-like	19599332	19600032	downstream_gene_variant	Transcriptional regulation
738	LG22	19598328	DJ_019089-T1	5-hydroxytryptamine receptor 3A-like	19602673	19610176	upstream_gene_variant	Signal transduction
739	LG22	19603920	DJ_019088-T1	protein ripply2-like	19599332	19600032	upstream_gene_variant	Transcriptional regulation
740	LG22	19603920	DJ_019089-T1	5-hydroxytryptamine receptor 3A-like	19602673	19610176	intron_variant	Signal transduction
741	LG22	19603936	DJ_019088-T1	protein ripply2-like	19599332	19600032	upstream_gene_variant	Transcriptional regulation
742	LG22	19603936	DJ_019089-T1	5-hydroxytryptamine receptor 3A-like	19602673	19610176	intron_variant	Signal transduction
743	LG22	19604007	DJ_019088-T1	protein ripply2-like	19599332	19600032	upstream_gene_variant	Transcriptional regulation
744	LG22	19604007	DJ_019089-T1	5-hydroxytryptamine receptor 3A-like	19602673	19610176	intron_variant	Signal transduction
745	LG22	19680974	DJ_019093-T1	GTPase IMAP family member 8-like	19658349	19681697	intron_variant	Metabolic process
746	LG22	19680990	DJ_019093-T1	GTPase IMAP family member 8-like	19658349	19681697	intron_variant	Metabolic process
747	LG22	19680992	DJ_019093-T1	GTPase IMAP family member 8-like	19658349	19681697	intron_variant	Metabolic process
748	LG22	19681009	DJ_019093-T1	GTPase IMAP family member 8-like	19658349	19681697	intron_variant	Metabolic process
749	LG22	19681017	DJ_019093-T1	GTPase IMAP family member 8-like	19658349	19681697	intron_variant	Metabolic process
750	LG22	19681018	DJ_019093-T1	GTPase IMAP family member 8-like	19658349	19681697	intron_variant	Metabolic process

751	LG22	20078688	DJ_019109-T1	metabotropic glutamate receptor 1 isoform X1	20077099	20095195	intron_variant	Signal transduction
752	LG22	20104822	DJ_019109-T1	metabotropic glutamate receptor 1 isoform X1	20077099	20095195	downstream_gene_variant	Signal transduction
753	LG22	20104822	DJ_019110-T1	metabotropic glutamate receptor 1 isoform X1	20095230	20102695	downstream_gene_variant	Signal transduction
754	LG22	20104822	DJ_019111-T1	ras-related protein Rab-32	20104413	20113982	downstream_gene_variant	Signal transduction
755	LG22	20175054	DJ_019121-T1	androglobin isoform X4	20163718	20186922	intron_variant	Signal transduction
756	LG22	20175055	DJ_019121-T1	androglobin isoform X4	20163718	20186922	intron_variant	Signal transduction
757	LG22	20324981	DJ_019124-T1	syntaxin-binding protein 5-like	20301441	20346942	intron_variant	Transmembrane transport
758	LG22	20325056	DJ_019124-T1	syntaxin-binding protein 5-like	20301441	20346942	intron_variant	Transmembrane transport
759	LG22	20630360	DJ_019125-T1	SAM and SH3 domain-containing 1a	20603446	20628217	downstream_gene_variant	Signal transduction
760	LG22	20630360	DJ_019126-T1	N/A	20635520	20636322	downstream_gene_variant	N/A
761	LG22	20630462	DJ_019125-T1	SAM and SH3 domain-containing 1a	20603446	20628217	downstream_gene_variant	Signal transduction
762	LG22	20630462	DJ_019126-T1	N/A	20635520	20636322	downstream_gene_variant	N/A
763	LG22	20713752	DJ_019129-T1	Opsin-5	20706131	20710405	downstream_gene_variant	Visual perception
764	LG22	20713752	DJ_019130-T1	methylmalonyl-CoA mutase, mitochondrial	20719605	20759086	downstream_gene_variant	Metabolic process
765	LG22	20713769	DJ_019129-T1	Opsin-5	20706131	20710405	downstream_gene_variant	Visual perception
766	LG22	20713769	DJ_019130-T1	methylmalonyl-CoA mutase, mitochondrial	20719605	20759086	downstream_gene_variant	Metabolic process
767	LG22	20713781	DJ_019129-T1	Opsin-5	20706131	20710405	downstream_gene_variant	Visual perception
768	LG22	20713781	DJ_019130-T1	methylmalonyl-CoA mutase, mitochondrial	20719605	20759086	downstream_gene_variant	Metabolic process
769	LG22	20713898	DJ_019129-T1	Opsin-5	20706131	20710405	downstream_gene_variant	Visual perception
770	LG22	20713898	DJ_019130-T1	methylmalonyl-CoA mutase, mitochondrial	20719605	20759086	downstream_gene_variant	Metabolic process
771	LG22	20713915	DJ_019129-T1	Opsin-5	20706131	20710405	downstream_gene_variant	Visual perception
772	LG22	20713915	DJ_019130-T1	methylmalonyl-CoA mutase, mitochondrial	20719605	20759086	downstream_gene_variant	Metabolic process
773	LG22	20713946	DJ_019129-T1	Opsin-5	20706131	20710405	downstream_gene_variant	Visual perception

774	LG22	20713946	DJ_019130-T1	methylmalonyl-CoA mutase, mitochondrial	20719605	20759086	downstream_gene_variant	Metabolic process
775	LG22	20745858	DJ_019130-T1	methylmalonyl-CoA mutase, mitochondrial	20719605	20759086	intron_variant	Metabolic process
776	LG22	20745870	DJ_019130-T1	methylmalonyl-CoA mutase, mitochondrial	20719605	20759086	intron_variant	Metabolic process
777	LG22	20745871	DJ_019130-T1	methylmalonyl-CoA mutase, mitochondrial	20719605	20759086	intron_variant	Metabolic process
778	LG22	20745882	DJ_019130-T1	methylmalonyl-CoA mutase, mitochondrial	20719605	20759086	intron_variant	Metabolic process
779	LG22	20745910	DJ_019130-T1	methylmalonyl-CoA mutase, mitochondrial	20719605	20759086	intron_variant	Metabolic process
780	LG22	20759951	DJ_019130-T1	methylmalonyl-CoA mutase, mitochondrial	20719605	20759086	intron_variant	Metabolic process
781	LG22	20759951	DJ_019131-T1	heat shock protein HSP 90-alpha 1	20760599	20761800	upstream_gene_variant	Thermal response
782	LG22	20759951	DJ_019132-T1	heat shock protein HSP 90-alpha 1	20763529	20765394	downstream_gene_variant	Thermal response
783	LG22	20759951	DJ_019133-T1	peptidyl-prolyl cis-trans isomerase FKBP1B	20767779	20768633	upstream_gene_variant	Immune response
784	LG22	20766412	DJ_019130-T1	methylmalonyl-CoA mutase, mitochondrial	20719605	20759086	downstream_gene_variant	Metabolic process
785	LG22	20766412	DJ_019131-T1	heat shock protein HSP 90-alpha 1	20760599	20761800	upstream_gene_variant	Thermal response
786	LG22	20766412	DJ_019132-T1	heat shock protein HSP 90-alpha 1	20763529	20765394	upstream_gene_variant	Thermal response
787	LG22	20766412	DJ_019133-T1	peptidyl-prolyl cis-trans isomerase FKBP1B	20767779	20768633	downstream_gene_variant	Immune response
788	LG22	20776538	DJ_019133-T1	peptidyl-prolyl cis-trans isomerase FKBP1B	20767779	20768633	upstream_gene_variant	Immune response
789	LG22	20776538	DJ_019134-T1	WD repeat and coiled coil containing	20786107	20791070	upstream_gene_variant	Signal transduction
790	LG22	20776586	DJ_019133-T1	peptidyl-prolyl cis-trans isomerase FKBP1B	20767779	20768633	upstream_gene_variant	Immune response
791	LG22	20776586	DJ_019134-T1	WD repeat and coiled coil containing	20786107	20791070	upstream_gene_variant	Signal transduction
792	LG22	20776634	DJ_019133-T1	peptidyl-prolyl cis-trans isomerase FKBP1B	20767779	20768633	upstream_gene_variant	Immune response
793	LG22	20776634	DJ_019134-T1	WD repeat and coiled coil containing	20786107	20791070	upstream_gene_variant	Signal transduction
794	LG22	20776713	DJ_019133-T1	peptidyl-prolyl cis-trans isomerase FKBP1B	20767779	20768633	upstream_gene_variant	Immune response
795	LG22	20776713	DJ_019134-T1	WD repeat and coiled coil containing	20786107	20791070	upstream_gene_variant	Signal transduction

796	LG22	20776720	DJ_019133-T1	peptidyl-prolyl cis-trans isomerase FKBP1B	20767779	20768633	upstream_gene_variant	Immune response
797	LG22	20776720	DJ_019134-T1	WD repeat and coiled coil containing	20786107	20791070	upstream_gene_variant	Signal transduction
798	LG22	20788783	DJ_019134-T1	WD repeat and coiled coil containing	20786107	20791070	intron_variant	Signal transduction
799	LG22	20788783	DJ_019135-T1	Major facilitator superfamily domain-containing 2B	20792999	20820525	downstream_gene_variant	Metabolic process
800	LG22	20788827	DJ_019134-T1	WD repeat and coiled coil containing	20786107	20791070	intron_variant	Signal transduction
801	LG22	20788827	DJ_019135-T1	Major facilitator superfamily domain-containing 2B	20792999	20820525	downstream_gene_variant	Metabolic process
802	LG22	20788893	DJ_019134-T1	WD repeat and coiled coil containing	20786107	20791070	intron_variant	Signal transduction
803	LG22	20788893	DJ_019135-T1	Major facilitator superfamily domain-containing 2B	20792999	20820525	downstream_gene_variant	Metabolic process
804	LG22	20788907	DJ_019134-T1	WD repeat and coiled coil containing	20786107	20791070	intron_variant	Signal transduction
805	LG22	20788907	DJ_019135-T1	Major facilitator superfamily domain-containing 2B	20792999	20820525	downstream_gene_variant	Metabolic process
806	LG22	20788922	DJ_019134-T1	WD repeat and coiled coil containing	20786107	20791070	intron_variant	Signal transduction
807	LG22	20788922	DJ_019135-T1	Major facilitator superfamily domain-containing 2B	20792999	20820525	downstream_gene_variant	Metabolic process
808	LG22	20788983	DJ_019134-T1	WD repeat and coiled coil containing	20786107	20791070	intron_variant	Signal transduction
809	LG22	20788983	DJ_019135-T1	Major facilitator superfamily domain-containing 2B	20792999	20820525	downstream_gene_variant	Metabolic process
810	LG22	20793368	DJ_019134-T1	WD repeat and coiled coil containing	20786107	20791070	downstream_gene_variant	Signal transduction
811	LG22	20793368	DJ_019135-T1	Major facilitator superfamily domain-containing 2B	20792999	20820525	intron_variant	Metabolic process
812	LG22	20793381	DJ_019134-T1	WD repeat and coiled coil containing	20786107	20791070	downstream_gene_variant	Signal transduction
813	LG22	20793381	DJ_019135-T1	Major facilitator superfamily domain-containing 2B	20792999	20820525	intron_variant	Metabolic process
814	LG22	20793389	DJ_019134-T1	WD repeat and coiled coil containing	20786107	20791070	downstream_gene_variant	Signal transduction
815	LG22	20793389	DJ_019135-T1	Major facilitator superfamily domain-containing 2B	20792999	20820525	intron_variant	Metabolic process
816	LG22	20793399	DJ_019134-T1	WD repeat and coiled coil containing	20786107	20791070	downstream_gene_variant	Signal transduction
817	LG22	20793399	DJ_019135-T1	Major facilitator superfamily domain-containing 2B	20792999	20820525	intron_variant	Metabolic process

818	LG22	20793486	DJ_019134-T1	WD repeat and coiled coil containing	20786107	20791070	downstream_gene_variant	Signal transduction
819	LG22	20793486	DJ_019135-T1	Major facilitator superfamily domain-containing 2B	20792999	20820525	intron_variant	Metabolic process
820	LG22	20793530	DJ_019134-T1	WD repeat and coiled coil containing	20786107	20791070	downstream_gene_variant	Signal transduction
821	LG22	20793530	DJ_019135-T1	Major facilitator superfamily domain-containing 2B	20792999	20820525	intron_variant	Metabolic process
822	LG22	20793539	DJ_019134-T1	WD repeat and coiled coil containing	20786107	20791070	downstream_gene_variant	Signal transduction
823	LG22	20793539	DJ_019135-T1	Major facilitator superfamily domain-containing 2B	20792999	20820525	intron_variant	Metabolic process
824	LG22	20817299	DJ_019135-T1	Major facilitator superfamily domain-containing 2B	20792999	20820525	intron_variant	Metabolic process
825	LG22	20817299	DJ_019136-T1	UBX domain protein 2A	20824193	20828872	downstream_gene_variant	Transcriptional regulation
826	LG22	20817334	DJ_019135-T1	Major facilitator superfamily domain-containing 2B	20792999	20820525	intron_variant	Metabolic process
827	LG22	20817334	DJ_019136-T1	UBX domain protein 2A	20824193	20828872	downstream_gene_variant	Transcriptional regulation
828	LG22	20824604	DJ_019135-T1	Major facilitator superfamily domain-containing 2B	20792999	20820525	upstream_gene_variant	Metabolic process
829	LG22	20824604	DJ_019136-T1	UBX domain protein 2A	20824193	20828872	intron_variant	Transcriptional regulation
830	LG22	20824610	DJ_019135-T1	Major facilitator superfamily domain-containing 2B	20792999	20820525	upstream_gene_variant	Metabolic process
831	LG22	20824610	DJ_019136-T1	UBX domain protein 2A	20824193	20828872	intron_variant	Transcriptional regulation
832	LG22	20824614	DJ_019135-T1	Major facilitator superfamily domain-containing 2B	20792999	20820525	upstream_gene_variant	Metabolic process
833	LG22	20824614	DJ_019136-T1	UBX domain protein 2A	20824193	20828872	intron_variant	Transcriptional regulation
834	LG22	20824641	DJ_019135-T1	Major facilitator superfamily domain-containing 2B	20792999	20820525	upstream_gene_variant	Metabolic process
835	LG22	20824641	DJ_019136-T1	UBX domain protein 2A	20824193	20828872	intron_variant	Transcriptional regulation
836	LG22	20824642	DJ_019135-T1	Major facilitator superfamily domain-containing 2B	20792999	20820525	upstream_gene_variant	Metabolic process
837	LG22	20824642	DJ_019136-T1	UBX domain protein 2A	20824193	20828872	intron_variant	Transcriptional regulation
838	LG22	20824678	DJ_019135-T1	Major facilitator superfamily domain-containing 2B	20792999	20820525	upstream_gene_variant	Metabolic process
839	LG22	20824678	DJ_019136-T1	UBX domain protein 2A	20824193	20828872	intron_variant	Transcriptional regulation

840	LG22	20843780	DJ_019137-T1	Spermatogenesis-defective protein 39	20840336	20854787	intron_variant	Maturation
841	LG22	20843818	DJ_019137-T1	Spermatogenesis-defective protein 39	20840336	20854787	intron_variant	Maturation
842	LG22	20843846	DJ_019137-T1	Spermatogenesis-defective protein 39	20840336	20854787	intron_variant	Maturation
843	LG22	20844797	DJ_019137-T1	Spermatogenesis-defective protein 39	20840336	20854787	intron_variant	Maturation
844	LG22	20844919	DJ_019137-T1	Spermatogenesis-defective protein 39	20840336	20854787	intron_variant	Maturation
845	LG22	20857739	DJ_019137-T1	Spermatogenesis-defective protein 39	20840336	20854787	upstream_gene_variant	Maturation
846	LG22	20857739	DJ_019138-T1	serine/threonine-protein kinase MRCK beta-like isoform X2	20860132	20860618	downstream_gene_variant	Signal transduction
847	LG22	20857739	DJ_019139-T1	serine/threonine-protein kinase MRCK beta-like isoform X2	20862712	20898478	downstream_gene_variant	Signal transduction
848	LG22	20905385	DJ_019139-T1	serine/threonine-protein kinase MRCK beta-like isoform X2	20862712	20898478	upstream_gene_variant	Signal transduction
849	LG22	20905385	DJ_019140-T1	serine/threonine-protein kinase MRCK beta-like isoform X2	20900974	20920707	intron_variant	Signal transduction
850	LG22	20970817	DJ_019142-T1	protein LBH-like	20975607	20979504	upstream_gene_variant	Growth
851	LG22	20970868	DJ_019142-T1	protein LBH-like	20975607	20979504	upstream_gene_variant	Growth
852	LG22	20982176	DJ_019142-T1	protein LBH-like	20975607	20979504	downstream_gene_variant	Growth
853	LG22	20982204	DJ_019142-T1	protein LBH-like	20975607	20979504	downstream_gene_variant	Growth
854	LG22	21018415	DJ_019143-T1	N/A	21025876	21036284	upstream_gene_variant	N/A
855	LG22	21018546	DJ_019143-T1	N/A	21025876	21036284	upstream_gene_variant	N/A
856	LG22	21025721	DJ_019143-T1	N/A	21025876	21036284	upstream_gene_variant	N/A
857	LG22	21045496	DJ_019143-T1	N/A	21025876	21036284	downstream_gene_variant	N/A
858	LG22	21052198	DJ_019144-T1	ectonucleotide pyrophosphatase/phosphodiesterase family member 5	21055972	21060766	downstream_gene_variant	Immune response
859	LG22	21075661	DJ_019145-T1	protein FAM167A	21064143	21068869	downstream_gene_variant	Signal transduction
860	LG22	21075661	DJ_019146-T1	Cerebral cavernous malformations protein 2 homolog	21081785	21093980	downstream_gene_variant	Growth
861	LG22	21075701	DJ_019145-T1	protein FAM167A	21064143	21068869	downstream_gene_variant	Signal transduction
862	LG22	21075701	DJ_019146-T1	Cerebral cavernous malformations protein 2 homolog	21081785	21093980	downstream_gene_variant	Growth
863	LG22	21075735	DJ_019145-T1	protein FAM167A	21064143	21068869	downstream_gene_variant	Signal transduction
864	LG22	21075735	DJ_019146-T1	Cerebral cavernous malformations protein 2 homolog	21081785	21093980	downstream_gene_variant	Growth

865	LG22	21079885	DJ_019146-T1	Cerebral cavernous malformations protein 2 homolog	21081785	21093980	downstream_gene_variant	Growth
866	LG22	21080178	DJ_019146-T1	Cerebral cavernous malformations protein 2 homolog	21081785	21093980	downstream_gene_variant	Growth
867	LG22	21087118	DJ_019146-T1	Cerebral cavernous malformations protein 2 homolog	21081785	21093980	intron_variant	Growth
868	LG22	21087151	DJ_019146-T1	Cerebral cavernous malformations protein 2 homolog	21081785	21093980	intron_variant	Growth
869	LG22	21087153	DJ_019146-T1	Cerebral cavernous malformations protein 2 homolog	21081785	21093980	intron_variant	Growth
870	LG22	21087234	DJ_019146-T1	Cerebral cavernous malformations protein 2 homolog	21081785	21093980	intron_variant	Growth
871	LG22	21166907	DJ_019148-T1	Myotubularin-related protein 9	21173229	21189752	upstream_gene_variant	Immune response
872	LG22	21167001	DJ_019148-T1	Myotubularin-related protein 9	21173229	21189752	upstream_gene_variant	Immune response
873	LG22	21167008	DJ_019148-T1	Myotubularin-related protein 9	21173229	21189752	upstream_gene_variant	Immune response
874	LG22	21167016	DJ_019148-T1	Myotubularin-related protein 9	21173229	21189752	upstream_gene_variant	Immune response
875	LG22	21167027	DJ_019148-T1	Myotubularin-related protein 9	21173229	21189752	upstream_gene_variant	Immune response
876	LG22	21175034	DJ_019148-T1	Myotubularin-related protein 9	21173229	21189752	intron_variant	Immune response
877	LG22	21198779	DJ_019148-T1	Myotubularin-related protein 9	21173229	21189752	downstream_gene_variant	Immune response
878	LG22	21198779	DJ_019149-T1	Myotubularin-related protein 9	21195650	21200854	intron_variant	Immune response
879	LG22	21198779	DJ_019150-T1	Myotubularin-related protein 9	21205243	21215927	upstream_gene_variant	Immune response
880	LG22	21198792	DJ_019148-T1	L-threonine 3-dehydrogenase, mitochondrial-like	21173229	21189752	downstream_gene_variant	Metabolic process
881	LG22	21198792	DJ_019149-T1	PIN2/TERF1-interacting telomerase inhibitor 1	21195650	21200854	intron_variant	Transcriptional regulation
882	LG22	21198792	DJ_019150-T1	Myotubularin-related protein 9	21205243	21215927	upstream_gene_variant	Immune response
883	LG22	21198874	DJ_019148-T1	L-threonine 3-dehydrogenase, mitochondrial-like	21173229	21189752	downstream_gene_variant	Metabolic process
884	LG22	21198874	DJ_019149-T1	PIN2/TERF1-interacting telomerase inhibitor 1	21195650	21200854	intron_variant	Transcriptional regulation
885	LG22	21198874	DJ_019150-T1	Myotubularin-related protein 9	21205243	21215927	upstream_gene_variant	Immune response
886	LG22	21198913	DJ_019148-T1	L-threonine 3-dehydrogenase, mitochondrial-like	21173229	21189752	downstream_gene_variant	Metabolic process

887	LG22	21198913	DJ_019149-T1	PIN2/TERF1-interacting telomerase inhibitor 1	21195650	21200854	intron_variant	Transcriptional regulation
888	LG22	21198913	DJ_019150-T1	Myotubularin-related protein 9	21205243	21215927	upstream_gene_variant	Immune response
889	LG22	21198925	DJ_019148-T1	L-threonine 3-dehydrogenase, mitochondrial-like	21173229	21189752	downstream_gene_variant	Metabolic process
890	LG22	21198925	DJ_019149-T1	PIN2/TERF1-interacting telomerase inhibitor 1	21195650	21200854	intron_variant	Transcriptional regulation
891	LG22	21198925	DJ_019150-T1	Myotubularin-related protein 9	21205243	21215927	upstream_gene_variant	Immune response
892	LG22	21198928	DJ_019148-T1	L-threonine 3-dehydrogenase, mitochondrial-like	21173229	21189752	downstream_gene_variant	Metabolic process
893	LG22	21198928	DJ_019149-T1	PIN2/TERF1-interacting telomerase inhibitor 1	21195650	21200854	intron_variant	Transcriptional regulation
894	LG22	21198928	DJ_019150-T1	Myotubularin-related protein 9	21205243	21215927	upstream_gene_variant	Immune response
895	LG22	21236159	DJ_019152-T1	transcription factor SOX-7	21242831	21245650	upstream_gene_variant	Transcriptional regulation
896	LG22	21236159	DJ_019153-T1	N/A	21245866	21248918	downstream_gene_variant	N/A
897	LG22	21249854	DJ_019152-T1	transcription factor SOX-7	21242831	21245650	downstream_gene_variant	Transcriptional regulation
898	LG22	21249854	DJ_019153-T1	N/A	21245866	21248918	upstream_gene_variant	N/A
899	LG22	21249854	DJ_019154-T1	SRY (sex determining region Y)-box 7	21252991	21264087	upstream_gene_variant	Transcriptional regulation
900	LG22	21249855	DJ_019152-T1	transcription factor SOX-7	21242831	21245650	downstream_gene_variant	Transcriptional regulation
901	LG22	21249855	DJ_019153-T1	N/A	21245866	21248918	upstream_gene_variant	N/A
902	LG22	21249855	DJ_019154-T1	SRY (sex determining region Y)-box 7	21252991	21264087	upstream_gene_variant	Transcriptional regulation
903	LG22	21249938	DJ_019152-T1	transcription factor SOX-7	21242831	21245650	downstream_gene_variant	Transcriptional regulation
904	LG22	21249938	DJ_019153-T1	N/A	21245866	21248918	upstream_gene_variant	N/A
905	LG22	21249938	DJ_019154-T1	SRY (sex determining region Y)-box 7	21252991	21264087	upstream_gene_variant	Transcriptional regulation
906	LG22	21299746	DJ_019156-T1	uncharacterized protein LOC115252990	21284557	21292485	upstream_gene_variant	N/A
907	LG22	21299746	DJ_019157-T1	uncharacterized protein LOC117773136	21294797	21296330	upstream_gene_variant	N/A
908	LG22	21299746	DJ_019158-T1	uncharacterized protein LOC117773136	21296426	21300514	intron_variant	N/A
909	LG22	21299934	DJ_019156-T1	uncharacterized protein LOC115252990	21284557	21292485	upstream_gene_variant	N/A
910	LG22	21299934	DJ_019157-T1	uncharacterized protein LOC117773136	21294797	21296330	upstream_gene_variant	N/A

911	LG22	21299934	DJ_019158-T1	uncharacterized protein LOC117773136	21296426	21300514	intron_variant	N/A
912	LG22	21363031	DJ_019160-T1	Tyrosine-protein kinase	21350018	21362695	downstream_gene_variant	Signal transduction
913	LG22	21401977	DJ_019162-T1	fibronectin type III domain-containing protein 4	21389944	21426216	intron_variant	Locomotion
914	LG22	21401995	DJ_019162-T1	fibronectin type III domain-containing protein 4	21389944	21426216	intron_variant	Locomotion
915	LG22	21404866	DJ_019162-T1	fibronectin type III domain-containing protein 4	21389944	21426216	intron_variant	Locomotion
916	LG22	21411687	DJ_019162-T1	fibronectin type III domain-containing protein 4	21389944	21426216	intron_variant	Locomotion
917	LG22	21411688	DJ_019162-T1	fibronectin type III domain-containing protein 4	21389944	21426216	intron_variant	Locomotion
918	LG22	21411694	DJ_019162-T1	fibronectin type III domain-containing protein 4	21389944	21426216	intron_variant	Locomotion
919	LG22	21411756	DJ_019162-T1	fibronectin type III domain-containing protein 4	21389944	21426216	intron_variant	Locomotion
920	LG22	21411790	DJ_019162-T1	fibronectin type III domain-containing protein 4	21389944	21426216	intron_variant	Locomotion
921	LG22	21438674	DJ_019163-T1	N/A	21436689	21438229	upstream_gene_variant	N/A
922	LG22	21438859	DJ_019163-T1	N/A	21436689	21438229	upstream_gene_variant	N/A
923	LG22	21438869	DJ_019163-T1	N/A	21436689	21438229	upstream_gene_variant	N/A
924	LG22	21438906	DJ_019163-T1	N/A	21436689	21438229	upstream_gene_variant	N/A
925	LG22	21438907	DJ_019163-T1	N/A	21436689	21438229	upstream_gene_variant	N/A
926	LG22	21438908	DJ_019163-T1	N/A	21436689	21438229	upstream_gene_variant	N/A
927	LG22	21450795	DJ_019164-T1	low density lipoprotein receptor adapter protein 1-like	21450899	21478598	downstream_gene_variant	Metabolic process
928	LG22	21463884	DJ_019164-T1	low density lipoprotein receptor adapter protein 1-like	21450899	21478598	intron_variant	Metabolic process
929	LG22	21463962	DJ_019164-T1	low density lipoprotein receptor adapter protein 1-like	21450899	21478598	intron_variant	Metabolic process
930	LG22	21463988	DJ_019164-T1	low density lipoprotein receptor adapter protein 1-like	21450899	21478598	intron_variant	Metabolic process
931	LG22	21463989	DJ_019164-T1	low density lipoprotein receptor adapter protein 1-like	21450899	21478598	intron_variant	Metabolic process
932	LG22	21482589	DJ_019164-T1	low density lipoprotein receptor adapter protein 1-like	21450899	21478598	upstream_gene_variant	Metabolic process
933	LG22	21482608	DJ_019164-T1	low density lipoprotein receptor adapter protein 1-like	21450899	21478598	upstream_gene_variant	Metabolic process
934	LG22	21482609	DJ_019164-T1	low density lipoprotein receptor adapter protein 1-like	21450899	21478598	upstream_gene_variant	Metabolic process
935	LG22	21482709	DJ_019164-T1	low density lipoprotein receptor adapter protein 1-like	21450899	21478598	upstream_gene_variant	Metabolic process

936	LG22	21482757	DJ_019164-T1	low density lipoprotein receptor adapter protein 1-like	21450899	21478598	upstream_gene_variant	Metabolic process
937	LG22	21537425	DJ_019165-T1	E3 ubiquitin-protein ligase RNF19B-like isoform X1	21512197	21532308	upstream_gene_variant	Immune response
938	LG22	21537425	DJ_019166-T1	zinc finger protein 513-like isoform X1	21539524	21549214	upstream_gene_variant	Growth
939	LG22	21542551	DJ_019166-T1	zinc finger protein 513-like isoform X1	21539524	21549214	intron_variant	Growth
940	LG22	21555380	DJ_019166-T1	zinc finger protein 513-like isoform X1	21539524	21549214	downstream_gene_variant	Growth
941	LG22	21555380	DJ_019167-T1	sorting nexin-17-like	21556238	21598280	downstream_gene_variant	Signal transduction
942	LG22	21555386	DJ_019166-T1	zinc finger protein 513-like isoform X1	21539524	21549214	downstream_gene_variant	Growth
943	LG22	21555386	DJ_019167-T1	sorting nexin-17-like	21556238	21598280	downstream_gene_variant	Signal transduction
944	LG22	21555403	DJ_019166-T1	zinc finger protein 513-like isoform X1	21539524	21549214	downstream_gene_variant	Growth
945	LG22	21555403	DJ_019167-T1	sorting nexin-17-like	21556238	21598280	downstream_gene_variant	Signal transduction
946	LG22	21576544	DJ_019167-T1	sorting nexin-17-like	21556238	21598280	downstream_gene_variant	Signal transduction
947	LG22	21592256	DJ_019167-T1	sorting nexin-17-like	21556238	21598280	downstream_gene_variant	Signal transduction
948	LG22	21592257	DJ_019167-T1	sorting nexin-17-like	21556238	21598280	downstream_gene_variant	Signal transduction
949	LG22	21593638	DJ_019167-T1	sorting nexin-17-like	21556238	21598280	downstream_gene_variant	Signal transduction
950	LG22	21593703	DJ_019167-T1	sorting nexin-17-like	21556238	21598280	downstream_gene_variant	Signal transduction
951	LG22	21596477	DJ_019167-T1	sorting nexin-17-like	21556238	21598280	downstream_gene_variant	Signal transduction
952	LG22	21596600	DJ_019167-T1	sorting nexin-17-like	21556238	21598280	downstream_gene_variant	Signal transduction
953	LG22	21614760	DJ_019168-T1	translation initiation factor eIF-2B subunit delta	21606641	21615425	synonymous_variant	Growth
954	LG22	21614848	DJ_019168-T1	translation initiation factor eIF-2B subunit delta	21606641	21615425	intron_variant	Growth
955	LG22	21624939	DJ_019168-T1	translation initiation factor eIF-2B subunit delta	21606641	21615425	upstream_gene_variant	Growth
956	LG22	21624939	DJ_019169-T1	Nucleoside diphosphate kinase	21625720	21629113	upstream_gene_variant	Metabolic process
957	LG22	21624939	DJ_019170-T1	Brain and acute leukemia cytoplasmic protein	21630469	21633425	downstream_gene_variant	Transmembrane transport

958	LG22	21626610	DJ_019169-T1	Nucleoside diphosphate kinase	21625720	21629113	intron_variant	Metabolic process
959	LG22	21626610	DJ_019170-T1	Brain and acute leukemia cytoplasmic protein	21630469	21633425	downstream_gene_variant	Transmembrane transport
960	LG22	21629214	DJ_019169-T1	Nucleoside diphosphate kinase	21625720	21629113	downstream_gene_variant	Metabolic process
961	LG22	21629214	DJ_019170-T1	Brain and acute leukemia cytoplasmic protein	21630469	21633425	downstream_gene_variant	Transmembrane transport
962	LG22	21629245	DJ_019169-T1	Nucleoside diphosphate kinase	21625720	21629113	downstream_gene_variant	Metabolic process
963	LG22	21629245	DJ_019170-T1	Brain and acute leukemia cytoplasmic protein	21630469	21633425	downstream_gene_variant	Transmembrane transport
964	LG22	21629253	DJ_019169-T1	Nucleoside diphosphate kinase	21625720	21629113	downstream_gene_variant	Metabolic process
965	LG22	21629253	DJ_019170-T1	Brain and acute leukemia cytoplasmic protein	21630469	21633425	downstream_gene_variant	Transmembrane transport
966	LG22	21629275	DJ_019169-T1	Nucleoside diphosphate kinase	21625720	21629113	downstream_gene_variant	Metabolic process
967	LG22	21629275	DJ_019170-T1	Brain and acute leukemia cytoplasmic protein	21630469	21633425	downstream_gene_variant	Transmembrane transport
968	LG22	21629285	DJ_019169-T1	Nucleoside diphosphate kinase	21625720	21629113	downstream_gene_variant	Metabolic process
969	LG22	21629285	DJ_019170-T1	Brain and acute leukemia cytoplasmic protein	21630469	21633425	downstream_gene_variant	Transmembrane transport
970	LG22	21629288	DJ_019169-T1	Nucleoside diphosphate kinase	21625720	21629113	downstream_gene_variant	Metabolic process
971	LG22	21629288	DJ_019170-T1	Brain and acute leukemia cytoplasmic protein	21630469	21633425	downstream_gene_variant	Transmembrane transport
972	LG22	21629298	DJ_019169-T1	Nucleoside diphosphate kinase	21625720	21629113	downstream_gene_variant	Metabolic process
973	LG22	21629298	DJ_019170-T1	Brain and acute leukemia cytoplasmic protein	21630469	21633425	downstream_gene_variant	Transmembrane transport
974	LG22	21629334	DJ_019169-T1	Nucleoside diphosphate kinase	21625720	21629113	downstream_gene_variant	Metabolic process
975	LG22	21629334	DJ_019170-T1	Brain and acute leukemia cytoplasmic protein	21630469	21633425	downstream_gene_variant	Transmembrane transport
976	LG22	21629337	DJ_019169-T1	Nucleoside diphosphate kinase	21625720	21629113	downstream_gene_variant	Metabolic process
977	LG22	21629337	DJ_019170-T1	Brain and acute leukemia cytoplasmic protein	21630469	21633425	downstream_gene_variant	Transmembrane transport
978	LG22	21629345	DJ_019169-T1	Nucleoside diphosphate kinase	21625720	21629113	downstream_gene_variant	Metabolic process
979	LG22	21629345	DJ_019170-T1	Brain and acute leukemia cytoplasmic protein	21630469	21633425	downstream_gene_variant	Transmembrane transport

980	LG22	21629357	DJ_019169-T1	Nucleoside diphosphate kinase	21625720	21629113	downstream_gene_variant	Metabolic process
981	LG22	21629357	DJ_019170-T1	Brain and acute leukemia cytoplasmic protein	21630469	21633425	downstream_gene_variant	Transmembrane transport
982	LG22	21629373	DJ_019169-T1	Nucleoside diphosphate kinase	21625720	21629113	downstream_gene_variant	Metabolic process
983	LG22	21629373	DJ_019170-T1	Brain and acute leukemia cytoplasmic protein	21630469	21633425	downstream_gene_variant	Transmembrane transport
984	LG22	21629377	DJ_019169-T1	Nucleoside diphosphate kinase	21625720	21629113	downstream_gene_variant	Metabolic process
985	LG22	21629377	DJ_019170-T1	Brain and acute leukemia cytoplasmic protein	21630469	21633425	downstream_gene_variant	Transmembrane transport
986	LG22	21629384	DJ_019169-T1	Nucleoside diphosphate kinase	21625720	21629113	downstream_gene_variant	Metabolic process
987	LG22	21629384	DJ_019170-T1	Brain and acute leukemia cytoplasmic protein	21630469	21633425	downstream_gene_variant	Transmembrane transport
988	LG22	21629387	DJ_019169-T1	Nucleoside diphosphate kinase	21625720	21629113	downstream_gene_variant	Metabolic process
989	LG22	21629387	DJ_019170-T1	Brain and acute leukemia cytoplasmic protein	21630469	21633425	downstream_gene_variant	Transmembrane transport
990	LG22	21629402	DJ_019169-T1	Nucleoside diphosphate kinase	21625720	21629113	downstream_gene_variant	Metabolic process
991	LG22	21629402	DJ_019170-T1	Brain and acute leukemia cytoplasmic protein	21630469	21633425	downstream_gene_variant	Transmembrane transport
992	LG22	21629403	DJ_019169-T1	Nucleoside diphosphate kinase	21625720	21629113	downstream_gene_variant	Metabolic process
993	LG22	21629403	DJ_019170-T1	Brain and acute leukemia cytoplasmic protein	21630469	21633425	downstream_gene_variant	Transmembrane transport
994	LG22	21631973	DJ_019169-T1	Nucleoside diphosphate kinase	21625720	21629113	downstream_gene_variant	Metabolic process
995	LG22	21631973	DJ_019170-T1	Brain and acute leukemia cytoplasmic protein	21630469	21633425	intron_variant	Transmembrane transport
996	LG22	21631973	DJ_019171-T1	PREDICTED: uncharacterized protein LOC107375226 isoform X1	21640426	21646146	downstream_gene_variant	N/A
997	LG22	21631992	DJ_019169-T1	Nucleoside diphosphate kinase	21625720	21629113	downstream_gene_variant	Metabolic process
998	LG22	21631992	DJ_019170-T1	Brain and acute leukemia cytoplasmic protein	21630469	21633425	intron_variant	Transmembrane transport
999	LG22	21631992	DJ_019171-T1	PREDICTED: uncharacterized protein LOC107375226 isoform X1	21640426	21646146	downstream_gene_variant	N/A
1000	LG22	21632299	DJ_019169-T1	Nucleoside diphosphate kinase	21625720	21629113	downstream_gene_variant	Metabolic process
1001	LG22	21632299	DJ_019170-T1	Brain and acute leukemia cytoplasmic protein	21630469	21633425	intron_variant	Transmembrane transport

1002	LG22	21632299	DJ_019171-T1	PREDICTED: uncharacterized protein LOC107375226 isoform X1	21640426	21646146	downstream_gene_variant	N/A
1003	LG22	21632315	DJ_019169-T1	Nucleoside diphosphate kinase	21625720	21629113	downstream_gene_variant	Metabolic process
1004	LG22	21632315	DJ_019170-T1	Brain and acute leukemia cytoplasmic protein	21630469	21633425	intron_variant	Transmembrane transport
1005	LG22	21632315	DJ_019171-T1	PREDICTED: uncharacterized protein LOC107375226 isoform X1	21640426	21646146	downstream_gene_variant	N/A
1006	LG22	21632734	DJ_019169-T1	Nucleoside diphosphate kinase	21625720	21629113	downstream_gene_variant	Metabolic process
1007	LG22	21632734	DJ_019170-T1	Brain and acute leukemia cytoplasmic protein	21630469	21633425	intron_variant	Transmembrane transport
1008	LG22	21632734	DJ_019171-T1	PREDICTED: uncharacterized protein LOC107375226 isoform X1	21640426	21646146	downstream_gene_variant	N/A
1009	LG22	21741797	DJ_019173-T1	uncharacterized protein LOC105897759	21735713	21738533	downstream_gene_variant	N/A
1010	LG22	21741797	DJ_019174-T1	neuronal acetylcholine receptor subunit alpha-2-like	21745484	21751699	upstream_gene_variant	Signal transduction
1011	LG22	21780176	DJ_019176-T1	protein-tyrosine kinase 2-beta	21758530	21778791	upstream_gene_variant	Immune response
1012	LG22	21780264	DJ_019176-T1	protein-tyrosine kinase 2-beta	21758530	21778791	upstream_gene_variant	Immune response
1013	LG22	21846861	DJ_019178-T1	Potassium voltage-gated channel subfamily H member 5	21839029	21851472	intron_variant	Osmoregulation
1014	LG22	21847044	DJ_019178-T1	Potassium voltage-gated channel subfamily H member 5	21839029	21851472	intron_variant	Osmoregulation
1015	LG22	21847092	DJ_019178-T1	Potassium voltage-gated channel subfamily H member 5	21839029	21851472	intron_variant	Osmoregulation
1016	LG22	21847104	DJ_019178-T1	Potassium voltage-gated channel subfamily H member 5	21839029	21851472	intron_variant	Osmoregulation
1017	LG22	21890601	DJ_019179-T1	Potassium voltage-gated channel subfamily H member 5	21878107	21891079	intron_variant	Osmoregulation
1018	LG22	21890639	DJ_019179-T1	Potassium voltage-gated channel subfamily H member 5	21878107	21891079	intron_variant	Osmoregulation
1019	LG22	21890677	DJ_019179-T1	Potassium voltage-gated channel subfamily H member 5	21878107	21891079	intron_variant	Osmoregulation
1020	LG22	21890681	DJ_019179-T1	Potassium voltage-gated channel subfamily H member 5	21878107	21891079	intron_variant	Osmoregulation
1021	LG22	21974380	DJ_019181-T1	Rho-related GTP-binding protein RhoJ (Fragment)	21969325	21970910	downstream_gene_variant	Signal transduction
1022	LG22	21974380	DJ_019182-T1	Serine/threonine protein phosphatase 2A regulatory subunit	21981259	21993681	downstream_gene_variant	Signal transduction
1023	LG22	21974388	DJ_019181-T1	Rho-related GTP-binding protein RhoJ (Fragment)	21969325	21970910	downstream_gene_variant	Signal transduction

1024	LG22	21974388	DJ_019182-T1	Serine/threonine protein phosphatase 2A regulatory subunit	21981259	21993681	downstream_gene_variant	Signal transduction
1025	LG22	21974440	DJ_019181-T1	Rho-related GTP-binding protein RhoJ (Fragment)	21969325	21970910	downstream_gene_variant	Signal transduction
1026	LG22	21974440	DJ_019182-T1	Serine/threonine protein phosphatase 2A regulatory subunit	21981259	21993681	downstream_gene_variant	Signal transduction
1027	LG22	21974635	DJ_019181-T1	Rho-related GTP-binding protein RhoJ (Fragment)	21969325	21970910	downstream_gene_variant	Signal transduction
1028	LG22	21974635	DJ_019182-T1	Serine/threonine protein phosphatase 2A regulatory subunit	21981259	21993681	downstream_gene_variant	Signal transduction
1029	LG22	21974691	DJ_019181-T1	Rho-related GTP-binding protein RhoJ (Fragment)	21969325	21970910	downstream_gene_variant	Signal transduction
1030	LG22	21974691	DJ_019182-T1	Serine/threonine protein phosphatase 2A regulatory subunit	21981259	21993681	downstream_gene_variant	Signal transduction
1031	LG22	21988525	DJ_019182-T1	Serine/threonine protein phosphatase 2A regulatory subunit	21981259	21993681	intron_variant	Signal transduction
1032	LG22	21988559	DJ_019182-T1	Serine/threonine protein phosphatase 2A regulatory subunit	21981259	21993681	intron_variant	Signal transduction
1033	LG22	21997015	DJ_019182-T1	Serine/threonine protein phosphatase 2A regulatory subunit	21981259	21993681	upstream_gene_variant	Signal transduction
1034	LG22	21997015	DJ_019183-T1	sphingosine-1-phosphate phosphatase 1-like	22005007	22018564	downstream_gene_variant	Metabolic process
1035	LG22	21997061	DJ_019182-T1	Serine/threonine protein phosphatase 2A regulatory subunit	21981259	21993681	upstream_gene_variant	Signal transduction
1036	LG22	21997061	DJ_019183-T1	sphingosine-1-phosphate phosphatase 1-like	22005007	22018564	downstream_gene_variant	Metabolic process
1037	LG22	21997131	DJ_019182-T1	Serine/threonine protein phosphatase 2A regulatory subunit	21981259	21993681	upstream_gene_variant	Signal transduction
1038	LG22	21997131	DJ_019183-T1	sphingosine-1-phosphate phosphatase 1-like	22005007	22018564	downstream_gene_variant	Metabolic process
1039	LG22	21997198	DJ_019182-T1	Serine/threonine protein phosphatase 2A regulatory subunit	21981259	21993681	upstream_gene_variant	Signal transduction
1040	LG22	21997198	DJ_019183-T1	sphingosine-1-phosphate phosphatase 1-like	22005007	22018564	downstream_gene_variant	Metabolic process
1041	LG22	21997249	DJ_019182-T1	Serine/threonine protein phosphatase 2A regulatory subunit	21981259	21993681	upstream_gene_variant	Signal transduction
1042	LG22	21997249	DJ_019183-T1	sphingosine-1-phosphate phosphatase 1-like	22005007	22018564	downstream_gene_variant	Metabolic process
1043	LG22	22020801	DJ_019183-T1	Serine/threonine protein phosphatase 2A regulatory subunit	22005007	22018564	upstream_gene_variant	Signal transduction
1044	LG22	22020801	DJ_019184-T1	sphingosine-1-phosphate phosphatase 1-like	22029835	22032740	downstream_gene_variant	Metabolic process
1045	LG22	22091168	DJ_019187-T1	Protein kinase C eta type	22072656	22094168	intron_variant	Signal transduction

1046	LG22	22101788	DJ_019187-T1	Protein kinase C eta type	22072656	22094168	upstream_gene_variant	Signal transduction
1047	LG22	22101804	DJ_019187-T1	Protein kinase C eta type	22072656	22094168	upstream_gene_variant	Signal transduction
1048	LG22	22101855	DJ_019187-T1	Protein kinase C eta type	22072656	22094168	upstream_gene_variant	Signal transduction
1049	LG22	22156275	DJ_019188-T1	Homeobox protein six1b	22159059	22167438	upstream_gene_variant	Transcriptional regulation
1050	LG22	22170812	DJ_019188-T1	Homeobox protein six1b	22159059	22167438	downstream_gene_variant	Transcriptional regulation
1051	LG22	22170812	DJ_019189-T1	Homeobox protein six1b	22179391	22181313	upstream_gene_variant	Transcriptional regulation
1052	LG22	22181666	DJ_019189-T1	Homeobox protein six1b	22179391	22181313	downstream_gene_variant	Transcriptional regulation
1053	LG22	22181685	DJ_019189-T1	Homeobox protein six1b	22179391	22181313	downstream_gene_variant	Transcriptional regulation
1054	LG22	22181706	DJ_019189-T1	Homeobox protein six1b	22179391	22181313	downstream_gene_variant	Transcriptional regulation
1055	LG22	22181737	DJ_019189-T1	Homeobox protein six1b	22179391	22181313	downstream_gene_variant	Transcriptional regulation
1056	LG22	22221712	DJ_019190-T1	Homeobox protein SIX6	22221473	22223528	intron_variant	Visual perception
1057	LG22	22301902	DJ_019191-T1	protein phosphatase 1A isoform X2	22288428	22300166	upstream_gene_variant	Signal transduction
1058	LG22	22302077	DJ_019191-T1	protein phosphatase 1A isoform X2	22288428	22300166	upstream_gene_variant	Signal transduction
1059	LG22	22303583	DJ_019191-T1	protein phosphatase 1A isoform X2	22288428	22300166	upstream_gene_variant	Signal transduction
1060	LG22	22303633	DJ_019191-T1	protein phosphatase 1A isoform X2	22288428	22300166	upstream_gene_variant	Signal transduction
1061	LG22	22303646	DJ_019191-T1	protein phosphatase 1A isoform X2	22288428	22300166	upstream_gene_variant	Signal transduction
1062	LG22	22303692	DJ_019191-T1	protein phosphatase 1A isoform X2	22288428	22300166	upstream_gene_variant	Signal transduction
1063	LG22	22303715	DJ_019191-T1	protein phosphatase 1A isoform X2	22288428	22300166	upstream_gene_variant	Signal transduction
1064	LG22	22303809	DJ_019191-T1	protein phosphatase 1A isoform X2	22288428	22300166	upstream_gene_variant	Signal transduction
1065	LG22	26940636	DJ_019345-T1	NHS-like protein 1 isoform X1	26938425	26939695	upstream_gene_variant	Locomotion
1066	LG22	26940636	DJ_019346-T1	Golgi resident protein GCP60-like isoform X1	26944164	26953556	downstream_gene_variant	Metabolic process
1067	LG22	26940660	DJ_019345-T1	NHS-like protein 1 isoform X1	26938425	26939695	upstream_gene_variant	Locomotion
1068	LG22	26940660	DJ_019346-T1	Golgi resident protein GCP60-like isoform X1	26944164	26953556	downstream_gene_variant	Metabolic process

1069	LG22	26940672	DJ_019345-T1	NHS-like protein 1 isoform X1	26938425	26939695	upstream_gene_variant	Locomotion
1070	LG22	26940672	DJ_019346-T1	Golgi resident protein GCP60-like isoform X1	26944164	26953556	downstream_gene_variant	Metabolic process
1071	LG22	26940683	DJ_019345-T1	NHS-like protein 1 isoform X1	26938425	26939695	upstream_gene_variant	Locomotion
1072	LG22	26940683	DJ_019346-T1	Golgi resident protein GCP60-like isoform X1	26944164	26953556	downstream_gene_variant	Metabolic process
1073	LG22	26940721	DJ_019345-T1	NHS-like protein 1 isoform X1	26938425	26939695	upstream_gene_variant	Locomotion
1074	LG22	26940721	DJ_019346-T1	Golgi resident protein GCP60-like isoform X1	26944164	26953556	downstream_gene_variant	Metabolic process
1075	LG22	26940731	DJ_019345-T1	NHS-like protein 1 isoform X1	26938425	26939695	upstream_gene_variant	Locomotion
1076	LG22	26940731	DJ_019346-T1	Golgi resident protein GCP60-like isoform X1	26944164	26953556	downstream_gene_variant	Metabolic process
1077	LG22	27001935	DJ_019351-T1	poly [ADP-ribose] polymerase 1	26988272	26995348	upstream_gene_variant	Signal transduction
1078	LG22	27001935	DJ_019352-T1	poly [ADP-ribose] polymerase 1	26995863	26996958	upstream_gene_variant	Signal transduction
1079	LG22	27001935	DJ_019353-T1	adenosine receptor A1-like	27005442	27007237	upstream_gene_variant	Signal transduction
1080	LG22	27001935	DJ_019354-T1	Poly [ADP-ribose] polymerase 1	27007386	27011304	downstream_gene_variant	Signal transduction
1081	LG22	27001938	DJ_019351-T1	poly [ADP-ribose] polymerase 1	26988272	26995348	upstream_gene_variant	Signal transduction
1082	LG22	27001938	DJ_019352-T1	poly [ADP-ribose] polymerase 1	26995863	26996958	upstream_gene_variant	Signal transduction
1083	LG22	27001938	DJ_019353-T1	adenosine receptor A1-like	27005442	27007237	upstream_gene_variant	Signal transduction
1084	LG22	27001938	DJ_019354-T1	Poly [ADP-ribose] polymerase 1	27007386	27011304	downstream_gene_variant	Signal transduction
1085	LG22	27003586	DJ_019351-T1	poly [ADP-ribose] polymerase 1	26988272	26995348	upstream_gene_variant	Signal transduction
1086	LG22	27003586	DJ_019352-T1	poly [ADP-ribose] polymerase 1	26995863	26996958	upstream_gene_variant	Signal transduction
1087	LG22	27003586	DJ_019353-T1	adenosine receptor A1-like	27005442	27007237	upstream_gene_variant	Signal transduction
1088	LG22	27003586	DJ_019354-T1	Poly [ADP-ribose] polymerase 1	27007386	27011304	downstream_gene_variant	Signal transduction
1089	LG22	27003597	DJ_019351-T1	poly [ADP-ribose] polymerase 1	26988272	26995348	upstream_gene_variant	Signal transduction
1090	LG22	27003597	DJ_019352-T1	poly [ADP-ribose] polymerase 1	26995863	26996958	upstream_gene_variant	Signal transduction
1091	LG22	27003597	DJ_019353-T1	adenosine receptor A1-like	27005442	27007237	upstream_gene_variant	Signal transduction
1092	LG22	27003597	DJ_019354-T1	Poly [ADP-ribose] polymerase 1	27007386	27011304	downstream_gene_variant	Signal transduction

1093	LG22	27003622	DJ_019351-T1	poly [ADP-ribose] polymerase 1	26988272	26995348	upstream_gene_variant	Signal transduction
1094	LG22	27003622	DJ_019352-T1	poly [ADP-ribose] polymerase 1	26995863	26996958	upstream_gene_variant	Signal transduction
1095	LG22	27003622	DJ_019353-T1	adenosine receptor A1-like	27005442	27007237	upstream_gene_variant	Signal transduction
1096	LG22	27003622	DJ_019354-T1	Poly [ADP-ribose] polymerase 1	27007386	27011304	downstream_gene_variant	Signal transduction
1097	LG22	27003644	DJ_019351-T1	poly [ADP-ribose] polymerase 1	26988272	26995348	upstream_gene_variant	Signal transduction
1098	LG22	27003644	DJ_019352-T1	poly [ADP-ribose] polymerase 1	26995863	26996958	upstream_gene_variant	Signal transduction
1099	LG22	27003644	DJ_019353-T1	adenosine receptor A1-like	27005442	27007237	upstream_gene_variant	Signal transduction
1100	LG22	27003644	DJ_019354-T1	Poly [ADP-ribose] polymerase 1	27007386	27011304	downstream_gene_variant	Signal transduction
1101	LG22	27003651	DJ_019351-T1	poly [ADP-ribose] polymerase 1	26988272	26995348	upstream_gene_variant	Signal transduction
1102	LG22	27003651	DJ_019352-T1	poly [ADP-ribose] polymerase 1	26995863	26996958	upstream_gene_variant	Signal transduction
1103	LG22	27003651	DJ_019353-T1	adenosine receptor A1-like	27005442	27007237	upstream_gene_variant	Signal transduction
1104	LG22	27003651	DJ_019354-T1	Poly [ADP-ribose] polymerase 1	27007386	27011304	downstream_gene_variant	Signal transduction
1105	LG22	27043140	DJ_019357-T1	N-lysine methyltransferase SMYD2-A-like	27034792	27043988	intron_variant	Metabolic process
1106	LG22	27043332	DJ_019357-T1	N-lysine methyltransferase SMYD2-A-like	27034792	27043988	intron_variant	Metabolic process
1107	LG22	27083437	DJ_019359-T1	prospero homeobox protein 1-like	27069943	27081973	upstream_gene_variant	Transcriptional regulation
1108	LG22	27083437	DJ_019360-T1	prospero homeobox protein 1-like	27083992	27086299	downstream_gene_variant	Transcriptional regulation
1109	LG6	740928	DJ_025342-T1	RNA-binding motif, single-stranded-interacting protein 3-like	745518	755139	downstream_gene_variant	Transcriptional regulation
1110	LG6	740988	DJ_025342-T1	RNA-binding motif, single-stranded-interacting protein 3-like	745518	755139	downstream_gene_variant	Transcriptional regulation
1111	LG6	847313	DJ_025343-T1	terminal nucleotidyltransferase 5A-likeprotein 3-like	829900	837681	upstream_gene_variant	Transcriptional regulation
1112	LG6	890579	DJ_025345-T1	palmitoyltransferase ZDHHC18-like	881893	894238	intron_variant	Metabolic process
1113	LG6	890579	DJ_025346-T1	fibrous sheath-interacting protein 2 isoform X3	895886	900795	downstream_gene_variant	Growth
1114	LG6	902806	DJ_025345-T1	palmitoyltransferase ZDHHC18-like	881893	894238	intron_variant	Metabolic process

1115	LG6	902806	DJ_025346-T1	fibrous sheath-interacting protein 2 isoform X3	895886	900795	downstream_gene_variant	Growth
1116	LG6	902806	DJ_025347-T1	fibrous sheath-interacting protein 2-like	902085	914991	missense_variant	Growth
1117	LG6	902881	DJ_025345-T1	palmitoyltransferase ZDHHC18-like	881893	894238	intron_variant	Metabolic process
1118	LG6	902881	DJ_025346-T1	fibrous sheath-interacting protein 2 isoform X3	895886	900795	downstream_gene_variant	Growth
1119	LG6	902881	DJ_025347-T1	fibrous sheath-interacting protein 2-like	902085	914991	missense_variant	Growth
1120	LG6	902925	DJ_025345-T1	palmitoyltransferase ZDHHC18-like	881893	894238	intron_variant	Metabolic process
1121	LG6	902925	DJ_025346-T1	fibrous sheath-interacting protein 2 isoform X3	895886	900795	downstream_gene_variant	Growth
1122	LG6	902925	DJ_025347-T1	fibrous sheath-interacting protein 2-like	902085	914991	synonymous_variant	Growth
1123	LG6	1056631	DJ_025353-T1	nuclear receptor subfamily 0 group B member 2-like	1055751	1056852	missense_variant	Signal transduction
1124	LG6	1056631	DJ_025354-T1	keratinocyte differentiation factor 1-like	1060539	1065803	downstream_gene_variant	Growth
1125	LG6	1079835	DJ_025355-T1	trophoblast glycoprotein-like	1088379	1089802	downstream_gene_variant	Signal transduction
1126	LG6	1136100	DJ_025359-T1	Syntaxin 12	1127149	1140667	intron_variant	Transmembrane transport
1127	LG6	2789130	DJ_025409-T1	sperm acrosome membrane-associated protein 4-like isoform X1	2779868	2781287	downstream_gene_variant	Growth
1128	LG6	2789130	DJ_025410-T1	ly-6/neurotoxin-like protein 1	2787481	2789953	intron_variant	Metabolic process
1129	LG6	2789130	DJ_025411-T1	Chloride intracellular channel protein	2794490	2799589	upstream_gene_variant	Osmoregulation
1130	LG6	2809555	DJ_025411-T1	Chloride intracellular channel protein	2794490	2799589	downstream_gene_variant	Osmoregulation
1131	LG6	2809555	DJ_025412-T1	N(G),N(G)-dimethylarginine dimethylaminohydrolase 2-like	2815725	2822397	upstream_gene_variant	Growth
1132	LG6	2834442	DJ_025413-T1	40S ribosomal protein S5	2824556	2828510	upstream_gene_variant	Transcriptional regulation
1133	LG6	2834442	DJ_025414-T1	lysosomal thioesterase PPT2	2832656	2837995	intron_variant	Metabolic process
1134	LG6	2834442	DJ_025415-T1	carnitine O-acetyltransferase-like	2839393	2852871	downstream_gene_variant	Metabolic process
1135	LG6	2851916	DJ_025415-T1	carnitine O-acetyltransferase-like	2839393	2852871	intron_variant	Metabolic process
1136	LG6	2851950	DJ_025415-T1	carnitine O-acetyltransferase-like	2839393	2852871	intron_variant	Metabolic process

1137	LG6	2889434	DJ_025417-T1	CUB domain-containing protein 1	2873074	2885663	downstream_gene_variant	Growth
1138	LG6	2889434	DJ_025418-T1	tetranectin-like	2887322	2888748	upstream_gene_variant	Metabolic process
1139	LG6	2889434	DJ_025419-T1	exosome complex component RRP42	2893062	2897299	downstream_gene_variant	Transcriptional regulation
1140	LG6	2895741	DJ_025418-T1	tetranectin-like	2887322	2888748	upstream_gene_variant	Metabolic process
1141	LG6	2895741	DJ_025419-T1	exosome complex component RRP42	2893062	2897299	intron_variant	Transcriptional regulation
1142	LG6	2895741	DJ_025420-T1	palmitoyltransferase ZDHHC3-like	2901991	2907926	upstream_gene_variant	Metabolic process
1143	LG6	2911128	DJ_025420-T1	palmitoyltransferase ZDHHC3-like	2901991	2907926	downstream_gene_variant	Metabolic process
1144	LG6	2911128	DJ_025421-T1	transmembrane protein 42-like	2916188	2917702	downstream_gene_variant	Transmembrane transport
1145	LG6	2911276	DJ_025420-T1	palmitoyltransferase ZDHHC3-like	2901991	2907926	downstream_gene_variant	Metabolic process
1146	LG6	2911276	DJ_025421-T1	transmembrane protein 42-like	2916188	2917702	downstream_gene_variant	Transmembrane transport
1147	LG6	2911348	DJ_025420-T1	palmitoyltransferase ZDHHC3-like	2901991	2907926	downstream_gene_variant	Metabolic process
1148	LG6	2911348	DJ_025421-T1	transmembrane protein 42-like	2916188	2917702	downstream_gene_variant	Transmembrane transport
1149	LG6	2915848	DJ_025420-T1	palmitoyltransferase ZDHHC3-like	2901991	2907926	downstream_gene_variant	Metabolic process
1150	LG6	2915848	DJ_025421-T1	transmembrane protein 42-like	2916188	2917702	downstream_gene_variant	Transmembrane transport
1151	LG6	2916034	DJ_025420-T1	palmitoyltransferase ZDHHC3-like	2901991	2907926	downstream_gene_variant	Metabolic process
1152	LG6	2916034	DJ_025421-T1	transmembrane protein 42-like	2916188	2917702	downstream_gene_variant	Transmembrane transport
1153	LG6	2921798	DJ_025421-T1	transmembrane protein 42	2916188	2917702	upstream_gene_variant	Transmembrane transport
1154	LG6	2921798	DJ_025422-T1	oxysterol-binding protein-related protein 10-like isoform X2	2927483	2966115	upstream_gene_variant	Metabolic process
1155	LG6	2921831	DJ_025421-T1	transmembrane protein 42	2916188	2917702	upstream_gene_variant	Transmembrane transport
1156	LG6	2921831	DJ_025422-T1	oxysterol-binding protein-related protein 10-like isoform X2	2927483	2966115	upstream_gene_variant	Metabolic process
1157	LG6	2931599	DJ_025422-T1	oxysterol-binding protein-related protein 10-like isoform X2	2927483	2966115	intron_variant	Metabolic process
1158	LG6	2931624	DJ_025422-T1	oxysterol-binding protein-related protein 10-like isoform X2	2927483	2966115	intron_variant	Metabolic process

1159	LG6	2943556	DJ_025422-T1	oxysterol-binding protein-related protein 10-like isoform X2	2927483	2966115	intron_variant	Metabolic process
1160	LG6	3383083	DJ_025446-T1	sclerostin domain-containing protein 1	3372978	3374494	downstream_gene_variant	Signal transduction
1161	LG6	3446228	DJ_025447-T1	hypothetical protein G5714_002794	3446656	3447550	downstream_gene_variant	N/A
1162	LG6	3446228	DJ_025448-T1	sterile alpha motif domain-containing protein 3-like	3449627	3451363	downstream_gene_variant	Metabolic process
1163	LG6	3500230	DJ_025450-T1	sodium bicarbonate cotransporter 3-like isoform X2	3486555	3505571	intron_variant	Osmoregulation
1164	LG6	3553628	DJ_025453-T1	Eomesodermin homolog a	3559776	3563254	downstream_gene_variant	Transcriptional regulation
1165	LG6	3553699	DJ_025453-T1	Eomesodermin homolog a	3559776	3563254	downstream_gene_variant	Transcriptional regulation
1166	LG6	3553714	DJ_025453-T1	Eomesodermin homolog a	3559776	3563254	downstream_gene_variant	Transcriptional regulation
1167	LG6	3553740	DJ_025453-T1	Eomesodermin homolog a	3559776	3563254	downstream_gene_variant	Transcriptional regulation
1168	LG6	3553929	DJ_025453-T1	Eomesodermin homolog a	3559776	3563254	downstream_gene_variant	Transcriptional regulation
1169	LG6	3558300	DJ_025453-T1	Eomesodermin homolog a	3559776	3563254	downstream_gene_variant	Transcriptional regulation
1170	LG6	3558303	DJ_025453-T1	Eomesodermin homolog a	3559776	3563254	downstream_gene_variant	Transcriptional regulation
1171	LG6	3558384	DJ_025453-T1	Eomesodermin homolog a	3559776	3563254	downstream_gene_variant	Transcriptional regulation
1172	LG6	3558895	DJ_025453-T1	Eomesodermin homolog a	3559776	3563254	downstream_gene_variant	Transcriptional regulation
1173	LG6	3568628	DJ_025453-T1	Eomesodermin homolog a	3559776	3563254	upstream_gene_variant	Transcriptional regulation
1174	LG6	3607847	DJ_025454-T1	N/A	3599233	3609647	intron_variant	N/A
1175	LG6	3646115	DJ_025455-T1	N/A	3626470	3645117	downstream_gene_variant	N/A
1176	LG6	3646115	DJ_025456-T1	RNA-binding motif, single-stranded-interacting protein 3 isoform X2	3650165	3654919	upstream_gene_variant	Transcriptional regulation
1177	LG6	3646133	DJ_025455-T1	N/A	3626470	3645117	downstream_gene_variant	N/A
1178	LG6	3646133	DJ_025456-T1	RNA-binding motif, single-stranded-interacting protein 3 isoform X2	3650165	3654919	upstream_gene_variant	Transcriptional regulation
1179	LG6	3909730	DJ_025463-T1	N/A	3886905	3903007	upstream_gene_variant	N/A
1180	LG6	3909730	DJ_025464-T1	N/A	3903131	3903601	downstream_gene_variant	N/A
1181	LG6	4689772	DJ_025476-T1	ubiquitin-conjugating enzyme E2Q-like protein 1	4693653	4700586	upstream_gene_variant	Signal transduction

1182	LG6	4689792	DJ_025476-T1	ubiquitin-conjugating enzyme E2Q-like protein 1	4693653	4700586	upstream_gene_variant	Signal transduction
1183	LG6	4689803	DJ_025476-T1	ubiquitin-conjugating enzyme E2Q-like protein 1	4693653	4700586	upstream_gene_variant	Signal transduction
1184	LG6	5148497	DJ_025493-T1	N/A	5143422	5144288	upstream_gene_variant	N/A
1185	LG6	7197203	DJ_025602-T1	microtubule-actin cross-linking factor 1-like isoform X1	7200106	7241105	upstream_gene_variant	Locomotion
1186	LG6	7197224	DJ_025602-T1	microtubule-actin cross-linking factor 1-like isoform X1	7200106	7241105	upstream_gene_variant	Locomotion
1187	LG6	7197227	DJ_025602-T1	microtubule-actin cross-linking factor 1-like isoform X1	7200106	7241105	upstream_gene_variant	Locomotion
1188	LG6	7384590	DJ_025607-T1	gamma-aminobutyric acid receptor subunit beta-4-like isoform X1	7387219	7421390	downstream_gene_variant	Metabolic process
1189	LG6	7384706	DJ_025607-T1	gamma-aminobutyric acid receptor subunit beta-4-like isoform X1	7387219	7421390	downstream_gene_variant	Metabolic process
1190	LG6	7384744	DJ_025607-T1	gamma-aminobutyric acid receptor subunit beta-4-like isoform X1	7387219	7421390	downstream_gene_variant	Metabolic process
1191	LG6	7384745	DJ_025607-T1	gamma-aminobutyric acid receptor subunit beta-4-like isoform X1	7387219	7421390	downstream_gene_variant	Metabolic process
1192	LG6	7428850	DJ_025607-T1	gamma-aminobutyric acid receptor subunit beta-4-like isoform X1	7387219	7421390	upstream_gene_variant	Metabolic process
1193	LG6	7428850	DJ_025609-T1	V-set and immunoglobulin domain-containing protein 1-like	7428219	7429791	intron_variant	Immune response
1194	LG6	7428850	DJ_025610-T1	55 kDa erythrocyte membrane protein	7434841	7445259	upstream_gene_variant	Signal transduction
1195	LG6	7461214	DJ_025612-T1	trimethyllysine dioxygenase, mitochondrial-like	7451510	7461442	intron_variant	Metabolic process
1196	LG6	7461214	DJ_025613-T1	delta-like protein B	7468806	7470924	downstream_gene_variant	Growth
1197	LG6	7628529	DJ_025622-T1	CLIP-associating protein 2 isoform X3	7623173	7647628	intron_variant	Locomotion
1198	LG6	8075638	DJ_025643-T1	homeobox protein XHOX-3-like	8067450	8069964	upstream_gene_variant	Transcriptional regulation
1199	LG6	8075638	DJ_025644-T1	homeobox protein Hox-A3a	8080710	8086761	upstream_gene_variant	Transcriptional regulation
1200	LG6	8075773	DJ_025643-T1	homeobox protein XHOX-3-like	8067450	8069964	upstream_gene_variant	Transcriptional regulation
1201	LG6	8075773	DJ_025644-T1	homeobox protein Hox-A3a	8080710	8086761	upstream_gene_variant	Transcriptional regulation
1202	LG6	8279887	DJ_025654-T1	protein CBFA2T1 isoform X3	8285433	8314851	downstream_gene_variant	Transcriptional regulation
1203	LG6	8279942	DJ_025654-T1	protein CBFA2T1 isoform X3	8285433	8314851	downstream_gene_variant	Transcriptional regulation
1204	LG6	8279977	DJ_025654-T1	protein CBFA2T1 isoform X3	8285433	8314851	downstream_gene_variant	Transcriptional regulation

1205	LG6	9034353	DJ_025672-T1	fatty acid-binding protein, heart-like	9041185	9043162	upstream_gene_variant	Metabolic process
1206	LG6	9034379	DJ_025672-T1	fatty acid-binding protein, heart-like	9041185	9043162	upstream_gene_variant	Metabolic process
1207	LG6	9141736	DJ_025676-T1	zinc finger protein 704 isoform X1	9147724	9155439	upstream_gene_variant	Transcriptional regulation
1208	LG6	9144305	DJ_025676-T1	zinc finger protein 704 isoform X1	9147724	9155439	upstream_gene_variant	Transcriptional regulation
1209	LG6	9144436	DJ_025676-T1	zinc finger protein 704 isoform X1	9147724	9155439	upstream_gene_variant	Transcriptional regulation
1210	LG6	9431799	DJ_025684-T1	N/A	9441265	9444180	downstream_gene_variant	N/A
1211	LG6	10064822	DJ_025696-T1	focal adhesion kinase 1 isoform X11	10007112	10068145	intron_variant	Signal transduction
1212	LG6	10136339	DJ_025698-T1	DENN domain-containing protein 3	10121714	10143437	intron_variant	Growth
1213	LG6	11187521	DJ_025734-T1	CMP-N-acetylneuraminate-beta-galactosamide-alpha-2,3-sialyltransferase 1	11195017	11198128	upstream_gene_variant	Metabolic process
1214	LG6	11187616	DJ_025734-T1	CMP-N-acetylneuraminate-beta-galactosamide-alpha-2,3-sialyltransferase 2	11195017	11198128	upstream_gene_variant	Metabolic process
1215	LG6	11834635	DJ_025756-T1	dynein heavy chain 6, axonemal	11843016	11845682	upstream_gene_variant	Locomotion
1216	LG6	11834636	DJ_025756-T1	dynein heavy chain 6, axonemal	11843016	11845682	upstream_gene_variant	Locomotion
1217	LG6	12822528	DJ_025784-T1	low-density lipoprotein receptor class A domain-containing protein 4 isoform X1	12812780	12830460	intron_variant	Signal transduction
1218	LG6	13151217	DJ_025800-T1	zinc finger protein ZFPM2-like	13159044	13184822	upstream_gene_variant	Transcriptional regulation
1219	LG6	15239690	DJ_025876-T1	Brain-specific angiogenesis inhibitor 1	15244267	15263958	upstream_gene_variant	Signal transduction
1220	LG6	15245987	DJ_025876-T1	Brain-specific angiogenesis inhibitor 1	15244267	15263958	intron_variant	Signal transduction
1221	LG6	15246013	DJ_025876-T1	Brain-specific angiogenesis inhibitor 1	15244267	15263958	intron_variant	Signal transduction
1222	LG6	15246175	DJ_025876-T1	Brain-specific angiogenesis inhibitor 1	15244267	15263958	intron_variant	Signal transduction
1223	LG6	15246235	DJ_025876-T1	Brain-specific angiogenesis inhibitor 1	15244267	15263958	intron_variant	Signal transduction
1224	LG6	15257456	DJ_025876-T1	Brain-specific angiogenesis inhibitor 1	15244267	15263958	intron_variant	Signal transduction
1225	LG6	15343723	DJ_025878-T1	regulating synaptic membrane exocytosis protein 3	15330218	15334663	upstream_gene_variant	Metabolic process
1226	LG6	15343763	DJ_025878-T1	regulating synaptic membrane exocytosis protein 3	15330218	15334663	upstream_gene_variant	Metabolic process

1227	LG6	15343785	DJ_025878-T1	regulating synaptic membrane exocytosis protein 3	15330218	15334663	upstream_gene_variant	Metabolic process
1228	LG6	15374192	DJ_025879-T1	regulating synaptic membrane exocytosis protein 3	15367513	15372015	upstream_gene_variant	Metabolic process
1229	LG6	15390482	DJ_025880-T1	glutathione S-transferase A-like	15391839	15396734	downstream_gene_variant	Metabolic process
1230	LG6	15390482	DJ_025881-T1	glutathione S-transferase theta-3-like	15397691	15400658	upstream_gene_variant	Metabolic process
1231	LG6	15451093	DJ_025882-T1	FH1/FH2 domain-containing protein 3-like isoform X1	15413360	15448420	upstream_gene_variant	Locomotion
1232	LG6	15451093	DJ_025883-T1	FH1/FH2 domain-containing protein 3-like isoform X1	15450423	15456561	intron_variant	Locomotion
1233	LG6	15489077	DJ_025884-T1	N/A	15492516	15503666	downstream_gene_variant	N/A
1234	LG6	15489136	DJ_025884-T1	N/A	15492516	15503666	downstream_gene_variant	N/A
1235	LG6	15514585	DJ_025885-T1	FH1/FH2 domain-containing protein 3-like isoform X4	15508330	15510605	upstream_gene_variant	Locomotion
1236	LG6	15514616	DJ_025885-T1	FH1/FH2 domain-containing protein 3-like isoform X4	15508330	15510605	upstream_gene_variant	Locomotion
1237	LG6	15583284	DJ_025888-T1	sodium-dependent neutral amino acid transporter B(0)AT1-like	15567381	15581533	downstream_gene_variant	Metabolic process
1238	LG6	15583284	DJ_025889-T1	sodium-dependent neutral amino acid transporter B(0)AT1-like	15592911	15608858	upstream_gene_variant	Metabolic process
1239	LG6	15645969	DJ_025891-T1	telomerase reverse transcriptase	15629170	15639755	upstream_gene_variant	Growth
1240	LG6	15645969	DJ_025892-T1	nucleoporin-like protein 2	15640581	15643626	upstream_gene_variant	Transcriptional regulation
1241	LG6	15688067	DJ_025893-T1	rap guanine nucleotide exchange factor 5 isoform X4	15681716	15708570	intron_variant	Signal transduction
1242	LG6	15690905	DJ_025893-T1	rap guanine nucleotide exchange factor 5 isoform X4	15681716	15708570	intron_variant	Signal transduction
1243	LG6	15690920	DJ_025893-T1	rap guanine nucleotide exchange factor 5 isoform X4	15681716	15708570	intron_variant	Signal transduction
1244	LG6	15691099	DJ_025893-T1	rap guanine nucleotide exchange factor 5 isoform X4	15681716	15708570	intron_variant	Signal transduction
1245	LG6	16122849	DJ_025906-T1	Histone deacetylase 9b	16105027	16130945	intron_variant	Transcriptional regulation
1246	LG6	16122908	DJ_025906-T1	Histone deacetylase 9b	16105027	16130945	intron_variant	Transcriptional regulation
1247	LG6	16122956	DJ_025906-T1	Histone deacetylase 9b	16105027	16130945	intron_variant	Transcriptional regulation
1248	LG6	16131875	DJ_025906-T1	Histone deacetylase 9b	16105027	16130945	upstream_gene_variant	Transcriptional regulation
1249	LG6	16131877	DJ_025906-T1	Histone deacetylase 9b	16105027	16130945	upstream_gene_variant	Transcriptional regulation
1250	LG6	16148582	DJ_025907-T1	N/A	16143179	16152666	intron_variant	N/A

1251	LG6	16148582	DJ_025908-T1	Sorting nexin 13	16155383	16191548	upstream_gene_variant	Signal transduction
1252	LG6	16153726	DJ_025907-T1	N/A	16143179	16152666	upstream_gene_variant	N/A
1253	LG6	16153726	DJ_025908-T1	Sorting nexin 13	16155383	16191548	upstream_gene_variant	Signal transduction
1254	LG6	16153755	DJ_025907-T1	N/A	16143179	16152666	upstream_gene_variant	N/A
1255	LG6	16153755	DJ_025908-T1	Sorting nexin 13	16155383	16191548	upstream_gene_variant	Signal transduction
1256	LG6	16181783	DJ_025908-T1	Sorting nexin 13	16155383	16191548	intron_variant	Signal transduction
1257	LG6	16181841	DJ_025908-T1	Sorting nexin 13	16155383	16191548	intron_variant	Signal transduction
1258	LG6	16181985	DJ_025908-T1	Sorting nexin 13	16155383	16191548	intron_variant	Signal transduction
1259	LG6	16182010	DJ_025908-T1	Sorting nexin 13	16155383	16191548	intron_variant	Signal transduction
1260	LG6	16182052	DJ_025908-T1	Sorting nexin 13	16155383	16191548	intron_variant	Signal transduction
1261	LG6	16182082	DJ_025908-T1	Sorting nexin 13	16155383	16191548	intron_variant	Signal transduction
1262	LG6	16192853	DJ_025908-T1	Sorting nexin 13	16155383	16191548	downstream_gene_variant	Signal transduction
1263	LG6	16192924	DJ_025908-T1	Sorting nexin 13	16155383	16191548	downstream_gene_variant	Signal transduction
1264	LG6	16195057	DJ_025908-T1	Sorting nexin 13	16155383	16191548	downstream_gene_variant	Signal transduction
1265	LG6	16195116	DJ_025908-T1	Sorting nexin 13	16155383	16191548	downstream_gene_variant	Signal transduction
1266	LG6	16248454	DJ_025911-T1	serine/threonine-protein phosphatase 6 regulatory ankyrin repeat subunit A isoform X2	16222833	16256199	intron_variant	Signal transduction
1267	LG6	16248454	DJ_025912-T1	biotinidase-like	16258043	16259988	downstream_gene_variant	Metabolic process
1268	LG6	16248457	DJ_025911-T1	serine/threonine-protein phosphatase 6 regulatory ankyrin repeat subunit A isoform X2	16222833	16256199	intron_variant	Signal transduction
1269	LG6	16248457	DJ_025912-T1	biotinidase-like	16258043	16259988	downstream_gene_variant	Metabolic process
1270	LG6	16256809	DJ_025911-T1	serine/threonine-protein phosphatase 6 regulatory ankyrin repeat subunit A isoform X2	16222833	16256199	downstream_gene_variant	Signal transduction
1271	LG6	16256809	DJ_025912-T1	biotinidase-like	16258043	16259988	downstream_gene_variant	Metabolic process

1272	LG6	16256809	DJ_025913-T1	serine/threonine-protein phosphatase 6 regulatory ankyrin repeat subunit A isoform X2	16262747	16281277	downstream_gene_variant	Signal transduction
1273	LG6	16256865	DJ_025911-T1	biotinidase-like	16222833	16256199	downstream_gene_variant	Metabolic process
1274	LG6	16256865	DJ_025912-T1	serine/threonine-protein phosphatase 6 regulatory ankyrin repeat subunit A isoform X2	16258043	16259988	downstream_gene_variant	Signal transduction
1275	LG6	16256865	DJ_025913-T1	SH3 domain-binding protein 5-like	16262747	16281277	downstream_gene_variant	Signal transduction
1276	LG6	16281236	DJ_025913-T1	SH3 domain-binding protein 5-like	16262747	16281277	synonymous_variant	Signal transduction
1277	LG6	16281236	DJ_025914-T1	maturin	16284605	16290157	upstream_gene_variant	Growth
1278	LG6	16281245	DJ_025913-T1	SH3 domain-binding protein 5-like	16262747	16281277	synonymous_variant	Signal transduction
1279	LG6	16281245	DJ_025914-T1	maturin	16284605	16290157	upstream_gene_variant	Growth
1280	LG6	16281386	DJ_025913-T1	SH3 domain-binding protein 5-like	16262747	16281277	upstream_gene_variant	Signal transduction
1281	LG6	16281386	DJ_025914-T1	maturin	16284605	16290157	upstream_gene_variant	Growth
1282	LG6	16281419	DJ_025913-T1	SH3 domain-binding protein 5-like	16262747	16281277	upstream_gene_variant	Signal transduction
1283	LG6	16281419	DJ_025914-T1	maturin	16284605	16290157	upstream_gene_variant	Growth
1284	LG6	16281422	DJ_025913-T1	SH3 domain-binding protein 5-like	16262747	16281277	upstream_gene_variant	Signal transduction
1285	LG6	16281422	DJ_025914-T1	maturin	16284605	16290157	upstream_gene_variant	Growth
1286	LG6	16285258	DJ_025913-T1	SH3 domain-binding protein 5-like	16262747	16281277	upstream_gene_variant	Signal transduction
1287	LG6	16285258	DJ_025914-T1	maturin	16284605	16290157	intron_variant	Growth
1288	LG6	16285260	DJ_025913-T1	SH3 domain-binding protein 5-like	16262747	16281277	upstream_gene_variant	Signal transduction
1289	LG6	16285260	DJ_025914-T1	maturin	16284605	16290157	intron_variant	Growth
1290	LG6	16285277	DJ_025913-T1	SH3 domain-binding protein 5-like	16262747	16281277	upstream_gene_variant	Signal transduction
1291	LG6	16285277	DJ_025914-T1	maturin	16284605	16290157	intron_variant	Growth
1292	LG6	16285349	DJ_025913-T1	SH3 domain-binding protein 5-like	16262747	16281277	upstream_gene_variant	Signal transduction
1293	LG6	16285349	DJ_025914-T1	maturin	16284605	16290157	intron_variant	Growth
1294	LG6	16288764	DJ_025913-T1	SH3 domain-binding protein 5-like	16262747	16281277	upstream_gene_variant	Signal transduction
1295	LG6	16288764	DJ_025914-T1	maturin	16284605	16290157	intron_variant	Growth
1296	LG6	16288775	DJ_025913-T1	SH3 domain-binding protein 5-like	16262747	16281277	upstream_gene_variant	Signal transduction
1297	LG6	16288775	DJ_025914-T1	maturin	16284605	16290157	intron_variant	Growth

1298	LG6	16288911	DJ_025913-T1	SH3 domain-binding protein 5-like	16262747	16281277	upstream_gene_variant	Signal transduction
1299	LG6	16288911	DJ_025914-T1	maturin	16284605	16290157	intron_variant	Growth
1300	LG6	16288939	DJ_025913-T1	SH3 domain-binding protein 5-like	16262747	16281277	upstream_gene_variant	Signal transduction
1301	LG6	16288939	DJ_025914-T1	maturin	16284605	16290157	intron_variant	Growth
1302	LG6	16288951	DJ_025913-T1	SH3 domain-binding protein 5-like	16262747	16281277	upstream_gene_variant	Signal transduction
1303	LG6	16288951	DJ_025914-T1	maturin	16284605	16290157	intron_variant	Growth
1304	LG6	19801470	DJ_026006-T1	IgGFc-binding protein-like	19798538	19811607	intron_variant	Growth
1305	LG6	19819227	DJ_026006-T1	IgGFc-binding protein-like	19798538	19811607	downstream_gene_variant	Growth
1306	LG6	19819227	DJ_026007-T1	tRNA-dihydrouridine(47) synthase [NAD(P)(+)]-like	19812363	19813841	upstream_gene_variant	Transcriptional regulation
1307	LG6	19819227	DJ_026008-T1	tRNA-dihydrouridine(47) synthase [NAD(P)(+)]-like	19813947	19818218	upstream_gene_variant	Transcriptional regulation
1308	LG6	19819227	DJ_026009-T1	A-kinase anchor protein 9 isoform X5	19824152	19881587	upstream_gene_variant	Signal transduction
1309	LG6	19945509	DJ_026012-T1	lanosterol 14-alpha demethylase-like	19940143	19945784	intron_variant	Hypoxia
1310	LG6	19945509	DJ_026013-T1	leucine-rich repeat protein SHOC-2	19946819	19949887	downstream_gene_variant	Signal transduction
1311	LG6	19945509	DJ_026014-T1	krev interaction trapped protein 1	19951550	19967291	downstream_gene_variant	Growth
1312	LG6	19945521	DJ_026012-T1	lanosterol 14-alpha demethylase-like	19940143	19945784	intron_variant	Hypoxia
1313	LG6	19945521	DJ_026013-T1	leucine-rich repeat protein SHOC-2	19946819	19949887	downstream_gene_variant	Signal transduction
1314	LG6	19945521	DJ_026014-T1	krev interaction trapped protein 1	19951550	19967291	downstream_gene_variant	Growth
1315	LG6	20160383	DJ_026019-T1	potassium voltage-gated channel subfamily KQT member 4	20136077	20158089	downstream_gene_variant	Osmoregulation
1316	LG6	20160383	DJ_026020-T1	protein JTB-like isoform X2	20165447	20167352	upstream_gene_variant	Growth
1317	LG6	20163929	DJ_026019-T1	potassium voltage-gated channel subfamily KQT member 4	20136077	20158089	downstream_gene_variant	Osmoregulation
1318	LG6	20163929	DJ_026020-T1	protein JTB-like isoform X3	20165447	20167352	upstream_gene_variant	Growth
1319	LG6	20163929	DJ_026021-T1	cocaine- and amphetamine-regulated transcript protein-like	20172407	20173163	downstream_gene_variant	Signal transduction
1320	LG6	20175634	DJ_026020-T1	protein JTB-like isoform X3	20165447	20167352	downstream_gene_variant	Growth
1321	LG6	20175634	DJ_026021-T1	cocaine- and amphetamine-regulated transcript protein-like	20172407	20173163	upstream_gene_variant	Signal transduction
1322	LG6	20175634	DJ_026022-T1	cocaine- and amphetamine-regulated transcript protein-like	20179596	20190968	downstream_gene_variant	Signal transduction
1323	LG6	20183366	DJ_026022-T1	cocaine- and amphetamine-regulated transcript protein-like	20179596	20190968	intron_variant	Signal transduction

1324	LG6	21859600	DJ_026101-T1	Oxysterol-binding protein	21861875	21869998	upstream_gene_variant	Metabolic process
1325	LG6	21859673	DJ_026101-T1	Oxysterol-binding protein	21861875	21869998	upstream_gene_variant	Metabolic process
1326	LG6	21886476	DJ_026102-T1	Dolichyl-diphosphooligosaccharide--protein glycosyltransferase subunit STT3B	21876140	21910066	intron_variant	Transcriptional regulation
1327	LG6	22218676	DJ_026108-T1	stathmin-2 isoform X1	22202815	22210529	upstream_gene_variant	Growth
1328	LG6	22255044	DJ_026109-T1	Y+L amino acid transporter 2-like	22244014	22252774	downstream_gene_variant	Osmoregulation
1329	LG6	22323092	DJ_026110-T1	protein FAM65B isoform X1	22323559	22325390	upstream_gene_variant	Sensory perception
1330	LG6	22323092	DJ_026111-T1	protein FAM65B-like isoform X1	22327454	22340146	upstream_gene_variant	Sensory perception
1331	LG6	22417537	DJ_026118-T1	Transmembrane protein 64	22398024	22414026	upstream_gene_variant	Transmembrane transport
1332	LG6	22468167	DJ_026119-T1	asparagine synthetase	22475436	22488540	upstream_gene_variant	Metabolic process
1333	LG6	22490125	DJ_026119-T1	asparagine synthetase	22475436	22488540	downstream_gene_variant	Metabolic process
1334	LG6	22490125	DJ_026120-T1	Protachykinin 1	22491800	22496844	downstream_gene_variant	Immune response
1335	LG6	22490157	DJ_026119-T1	asparagine synthetase	22475436	22488540	downstream_gene_variant	Metabolic process
1336	LG6	22490157	DJ_026120-T1	Protachykinin 1	22491800	22496844	downstream_gene_variant	Immune response
1337	LG6	22607634	DJ_026122-T1	collagen alpha-1(XXVIII) chain-like	22583635	22611635	intron_variant	Signal transduction
1338	LG6	22607701	DJ_026122-T1	collagen alpha-1(XXVIII) chain-like	22583635	22611635	intron_variant	Signal transduction
1339	LG6	22638615	DJ_026124-T1	UBAP1-MVB12-associated (UMA)-domain containing protein 1	22625544	22643230	intron_variant	Metabolic process
1340	LG6	22638615	DJ_026125-T1	glucocorticoid-induced transcript 1 protein-like isoform X3	22645975	22647734	upstream_gene_variant	Transcriptional regulation
1341	LG6	23069475	DJ_026128-T1	N/A	23074287	23076044	downstream_gene_variant	N/A
1342	LG6	23450128	DJ_026140-T1	protein HGH1 homolog isoform X1	23450431	23455404	upstream_gene_variant	Growth
1343	LG6	23450128	DJ_026141-T1	testis-specific serine/threonine-protein kinase 5-like	23455744	23460780	downstream_gene_variant	Signal transduction
1344	LG6	23491057	DJ_026142-T1	ribosome biogenesis protein BOP1	23483576	23505626	intron_variant	Transcriptional regulation
1345	LG6	23491163	DJ_026142-T1	ribosome biogenesis protein BOP1	23483576	23505626	intron_variant	Transcriptional regulation
1346	LG6	23535329	DJ_026144-T1	basic helix-loop-helix transcription factor scleraxis-like	23524770	23526113	downstream_gene_variant	Transcriptional regulation

1347	LG6	23535329	DJ_026145-T1	N/A	23533272	23536235	intron_variant	N/A
1348	LG6	23535329	DJ_026146-T1	ribosome biogenesis protein BOP1	23542080	23551845	downstream_gene_variant	Transcriptional regulation
1349	LG6	23721344	DJ_026157-T1	Wnt1-inducible-signaling pathway protein 1	23714000	23721761	intron_variant	Immune response
1350	LG6	23721657	DJ_026157-T1	Wnt1-inducible-signaling pathway protein 1	23714000	23721761	intron_variant	Immune response
1351	LG6	27584703	DJ_026321-T1	proline-rich transmembrane protein 1-like	27579446	27587926	intron_variant	Transmembrane transport
1352	LG6	27691342	DJ_026328-T1	E3 ubiquitin-protein ligase RNF5	27693398	27702232	downstream_gene_variant	Metabolic process
1353	LG6	27772365	DJ_026332-T1	dysbindin-like isoform X1	27764762	27773274	intron_variant	Transcriptional regulation
1354	LG6	27772365	DJ_026333-T1	protein Jumonji-like isoform X2	27776866	27816334	downstream_gene_variant	Growth
1355	LG6	28096616	DJ_026337-T1	partitioning defective 6 homolog gamma-like	28106600	28109280	upstream_gene_variant	Metabolic process
1356	LG6	28141567	DJ_026339-T1	choline transporter-like protein 4	28116383	28133513	downstream_gene_variant	Metabolic process
1357	LG6	28141567	DJ_026340-T1	choline transporter-like protein 4	28134007	28135779	downstream_gene_variant	Metabolic process
1358	LG6	28141567	DJ_026341-T1	activity-dependent neuroprotector homeobox protein 2	28136794	28140476	upstream_gene_variant	Transcriptional regulation
1359	LG6	28383497	DJ_026359-T1	N/A	28374574	28378083	upstream_gene_variant	N/A
1360	LG6	30596681	DJ_026417-T1	N/A	30594870	30595403	downstream_gene_variant	N/A
1361	LG6	30596681	DJ_026418-T1	stathmin-like	30595624	30598784	synonymous_variant	Growth
1362	LG6	30640883	DJ_026419-T1	Serine incorporator 2	30640383	30649322	intron_variant	Metabolic process
1363	LG6	30641031	DJ_026419-T1	Serine incorporator 2	30640383	30649322	intron_variant	Metabolic process
1364	LG6	30641071	DJ_026419-T1	Serine incorporator 2	30640383	30649322	intron_variant	Metabolic process
1365	LG6	30641074	DJ_026419-T1	Serine incorporator 2	30640383	30649322	intron_variant	Metabolic process
1366	LG6	30641099	DJ_026419-T1	Serine incorporator 2	30640383	30649322	intron_variant	Metabolic process
1367	LG6	30692409	DJ_026420-T1	transcription factor HIVEP3-like	30687176	30701965	intron_variant	Transcriptional regulation
1368	LG6	30692507	DJ_026420-T1	transcription factor HIVEP3-like	30687176	30701965	intron_variant	Transcriptional regulation
1369	LG6	31991080	DJ_026466-T1	N/A	31986667	31996346	intron_variant	N/A
1370	LG6	32128810	DJ_026470-T1	N/A	32134720	32136756	upstream_gene_variant	N/A

Note: all genes within 10Kb of the candidate outlier SNPs were presented.

Table S11 Gene ontology (GO) enrichment of biological process for genes in chromosome inversion region on LG6.

GO.ID	Term	Annotated	Significant	Expected	Rank in classicFisher	classicFisher	weightFisher	FDR
GO:0045104	intermediate filament cytoskeleton organization	36	11	1.34	1	4.70E-08	4.70E-08	0.00024
GO:0010499	proteasomal ubiquitin-independent protein catabolic process	29	10	1.08	3	5.20E-08	5.20E-08	0.00024
GO:2000300	regulation of synaptic vesicle exocytosis	39	11	1.46	4	1.20E-07	1.20E-07	0.00037
GO:0032886	regulation of microtubule-based process	138	16	5.15	32	5.90E-05	1.90E-06	0.00402
GO:0050806	positive regulation of synaptic transmission	86	14	3.21	9	3.40E-06	2.20E-06	0.00402
GO:0048167	regulation of synaptic plasticity	95	13	3.55	27	5.10E-05	3.00E-06	0.00457
GO:0060832	oocyte animal/vegetal axis specification	5	4	0.19	14	9.40E-06	9.40E-06	0.01227
GO:0031581	hemidesmosome assembly	16	6	0.6	17	1.50E-05	1.50E-05	0.01713
GO:0007214	gamma-aminobutyric acid signaling pathway	32	8	1.2	19	1.70E-05	1.70E-05	0.01726
GO:0042060	wound healing	297	20	11.09	253	0.00833	2.70E-05	0.02243
GO:0048014	Tie signaling pathway	6	4	0.22	22	2.70E-05	2.70E-05	0.02243
GO:0048791	calcium ion-regulated exocytosis of neurotransmitter	44	9	1.64	23	3.00E-05	3.00E-05	0.02284
GO:0032456	endocytic recycling	56	10	2.09	25	3.70E-05	3.70E-05	0.02601
GO:2000233	negative regulation of rRNA processing	3	3	0.11	28	5.20E-05	5.20E-05	0.03394
GO:0030154	cell differentiation	3310	140	123.62	591	0.05768	9.00E-05	0.05482
GO:0035278	miRNA mediated inhibition of translation	14	5	0.52	38	0.00011	0.00011	0.06282
GO:0044210	'de novo' CTP biosynthetic process	4	3	0.15	49	0.0002	0.0002	0.1066
GO:0035024	negative regulation of Rho protein signal transduction	9	4	0.34	50	0.00021	0.00021	0.1066
GO:0051028	mRNA transport	59	9	2.2	58	0.00032	0.00026	0.12503
GO:0042147	retrograde transport, endosome to Golgi	59	9	2.2	59	0.00032	0.00032	0.14619
GO:0035095	behavioral response to nicotine	5	3	0.19	65	0.00049	0.00049	0.17909
GO:0090023	positive regulation of neutrophil chemotaxis	5	3	0.19	66	0.00049	0.00049	0.17909
GO:1903904	negative regulation of establishment of T cell polarity	5	3	0.19	67	0.00049	0.00049	0.17909
GO:1905872	negative regulation of protein localization to cell leading edge	5	3	0.19	68	0.00049	0.00049	0.17909
GO:2001107	negative regulation of Rho guanyl-nucleotide exchange factor activity	5	3	0.19	69	0.00049	0.00049	0.17909
GO:0061035	regulation of cartilage development	47	7	1.76	134	0.00169	0.00054	0.18977
GO:0051893	regulation of focal adhesion assembly	12	4	0.45	82	0.00075	0.00075	0.25381

GO:0031061	negative regulation of histone methylation	6	3	0.22	96	0.00095	0.00095	0.28934
GO:0051574	positive regulation of histone H3-K9 methylation	6	3	0.22	97	0.00095	0.00095	0.28934
GO:2000391	positive regulation of neutrophil extravasation	6	3	0.22	98	0.00095	0.00095	0.28934
GO:0006438	valyl-tRNA aminoacylation	2	2	0.07	118	0.00139	0.00139	0.37354
GO:0044375	regulation of peroxisome size	2	2	0.07	119	0.00139	0.00139	0.37354
GO:0045654	positive regulation of megakaryocyte differentiation	2	2	0.07	120	0.00139	0.00139	0.37354
GO:0061526	acetylcholine secretion	2	2	0.07	121	0.00139	0.00139	0.37354
GO:2000405	negative regulation of T cell migration	7	3	0.26	130	0.00162	0.00162	0.42291
GO:0045663	positive regulation of myoblast differentiation	15	4	0.56	140	0.0019	0.0019	0.4692
GO:0048026	positive regulation of mRNA splicing, via spliceosome	15	4	0.56	141	0.0019	0.0019	0.4692
GO:0051726	regulation of cell cycle	466	24	17.4	635	0.07095	0.00237	0.56986
GO:0015918	sterol transport	64	6	2.39	422	0.03192	0.00246	0.57563
GO:0051491	positive regulation of filopodium assembly	8	3	0.3	156	0.00252	0.00252	0.57563
GO:0033336	caudal fin development	19	5	0.71	78	0.00054	0.00366	0.7169
GO:0002084	protein depalmitoylation	9	3	0.34	176	0.00368	0.00368	0.7169
GO:0015810	aspartate transmembrane transport	9	3	0.34	177	0.00368	0.00368	0.7169
GO:0015871	choline transport	9	3	0.34	178	0.00368	0.00368	0.7169
GO:0043490	malate-aspartate shuttle	9	3	0.34	179	0.00368	0.00368	0.7169
GO:0046600	negative regulation of centriole replication	9	3	0.34	180	0.00368	0.00368	0.7169
GO:0051639	actin filament network formation	18	4	0.67	183	0.00389	0.00389	0.7169
GO:2001106	regulation of Rho guanyl-nucleotide exchange factor activity	8	5	0.3	10	3.70E-06	0.00405	0.7169
GO:0051570	regulation of histone H3-K9 methylation	9	5	0.34	13	8.00E-06	0.00405	0.7169
GO:0030974	thiamine pyrophosphate transmembrane transport	3	2	0.11	190	0.00408	0.00408	0.7169
GO:0035606	peptidyl-cysteine S-trans-nitrosylation	3	2	0.11	191	0.00408	0.00408	0.7169
GO:1902746	regulation of lens fiber cell differentiation	3	2	0.11	192	0.00408	0.00408	0.7169
GO:0030199	collagen fibril organization	43	6	1.61	200	0.00499	0.00499	0.80065
GO:0060173	limb development	25	4	0.93	308	0.01307	0.00511	0.80065
GO:0018279	protein N-linked glycosylation via asparagine	10	3	0.37	202	0.00511	0.00511	0.80065
GO:0070778	L-aspartate transmembrane transport	10	3	0.37	203	0.00511	0.00511	0.80065
GO:1903204	negative regulation of oxidative stress-induced neuron death	10	3	0.37	204	0.00511	0.00511	0.80065
GO:0031124	mRNA 3'-end processing	37	4	1.38	546	0.04813	0.00511	0.80065

GO:0042391	regulation of membrane potential	354	19	13.22	714	0.07331	0.00517	0.80065
GO:0006896	Golgi to vacuole transport	11	3	0.41	222	0.00684	0.00684	0.96149
GO:0007130	synaptonemal complex assembly	11	3	0.41	223	0.00684	0.00684	0.96149
GO:0034721	histone H3-K4 demethylation, trimethyl-H3-K4-specific	11	3	0.41	224	0.00684	0.00684	0.96149
GO:0060036	notochord cell vacuolation	11	3	0.41	225	0.00684	0.00684	0.96149
GO:0098957	anterograde axonal transport of mitochondrion	11	3	0.41	226	0.00684	0.00684	0.96149
GO:1903010	regulation of bone development	11	3	0.41	227	0.00684	0.00684	0.96149
GO:0038083	peptidyl-tyrosine autophosphorylation	46	6	1.72	234	0.00698	0.00698	0.96631
GO:2000049	positive regulation of cell-cell adhesion mediated by cadherin	4	2	0.15	247	0.00795	0.00795	1
GO:0006412	translation	447	31	16.69	87	0.00075	0.00853	1
GO:0048484	enteric nervous system development	48	6	1.79	256	0.00859	0.00859	1
GO:1901741	positive regulation of myoblast fusion	12	3	0.45	257	0.00887	0.00887	1
GO:0006163	purine nucleotide metabolic process	391	16	14.6	1700	0.39003	0.0089	1
GO:0043161	proteasome-mediated ubiquitin-dependent protein catabolic process	275	21	10.27	129	0.00162	0.009	1

Table S12 Gene ontology (GO) enrichment of biological process for genes in chromosome inversion region on LG22.

GO.ID	Term	Annotated	Significant	Expected	Rank in classicFisher	classicFisher	weightFisher	FDR
GO:0035723	interleukin-15-mediated signaling pathway	19	8	0.49	1	1.10E-08	1.10E-08	1.00E-04
GO:0006642	triglyceride mobilization	6	5	0.15	4	6.50E-08	6.50E-08	3.00E-04
GO:0050900	leukocyte migration	117	8	3.01	281	0.01081	4.30E-07	0.00131
GO:0034605	cellular response to heat	8	5	0.21	5	5.80E-07	5.80E-07	0.00132
GO:0001964	startle response	16	6	0.41	8	1.80E-06	1.80E-06	0.0024
GO:0018315	molybdenum incorporation into molybdenum-molybdopterin complex	5	4	0.13	9	2.10E-06	2.10E-06	0.0024
GO:0032324	molybdopterin cofactor biosynthetic process	5	4	0.13	10	2.10E-06	2.10E-06	0.0024
GO:0072579	glycine receptor clustering	5	4	0.13	11	2.10E-06	2.10E-06	0.0024
GO:0042953	lipoprotein transport	11	5	0.28	16	4.50E-06	4.50E-06	0.00457
GO:0007529	establishment of synaptic specificity at neuromuscular junction	6	4	0.15	18	6.20E-06	6.20E-06	0.00515
GO:0070898	RNA polymerase III preinitiation complex assembly	6	4	0.15	19	6.20E-06	6.20E-06	0.00515
GO:0070374	positive regulation of ERK1 and ERK2 cascade	81	11	2.08	20	7.10E-06	7.10E-06	0.00541
GO:0006777	Mo-molybdopterin cofactor biosynthetic process	7	4	0.18	22	1.40E-05	1.40E-05	0.00914
GO:0097112	gamma-aminobutyric acid receptor clustering	7	4	0.18	23	1.40E-05	1.40E-05	0.00914
GO:0006186	dGDP phosphorylation	3	3	0.08	27	1.70E-05	1.70E-05	0.00971
GO:0006756	AMP phosphorylation	3	3	0.08	28	1.70E-05	1.70E-05	0.00971
GO:0042110	T cell activation	102	9	2.63	121	0.00127	3.80E-05	0.02042
GO:0007097	nuclear migration	38	7	0.98	39	4.50E-05	4.50E-05	0.02284
GO:0043627	response to estrogen	29	5	0.75	103	0.00079	6.60E-05	0.03174
GO:0045429	positive regulation of nitric oxide biosynthetic process	5	3	0.13	53	0.00016	0.00016	0.0731
GO:0098962	regulation of postsynaptic neurotransmitter receptor activity	13	4	0.33	71	0.00026	0.00026	0.11312
GO:0000003	reproduction	351	13	9.03	864	0.12201	0.00032	0.12712
GO:0006398	mRNA 3'-end processing by stem-loop binding and cleavage	6	3	0.15	76	0.00032	0.00032	0.12712
GO:0030030	cell projection organization	1417	32	36.47	2381	0.80478	0.00038	0.14467
GO:0006357	regulation of transcription by RNA polymerase II	1851	75	47.64	42	5.40E-05	0.00045	0.16447

GO:0098789	pre-mRNA cleavage required for polyadenylation	7	3	0.18	89	0.00055	0.00055	0.18612
GO:1902616	acyl carnitine transmembrane transport	7	3	0.18	90	0.00055	0.00055	0.18612
GO:0002926	tRNA wobble base 5-methoxycarbonylmethyl-2-thiouridinylation	2	2	0.05	98	0.00066	0.00066	0.20795
GO:1990092	calcium-dependent self proteolysis	2	2	0.05	99	0.00066	0.00066	0.20795
GO:0000122	negative regulation of transcription by RNA polymerase II	386	22	9.94	86	0.00046	0.00084	0.25584
GO:0050821	protein stabilization	30	5	0.77	107	0.00093	0.00093	0.27411
GO:0045859	regulation of protein kinase activity	334	9	8.6	1806	0.4919	0.00126	0.34129
GO:0030240	skeletal muscle thin filament assembly	9	3	0.23	114	0.00127	0.00127	0.34129
GO:0051597	response to methylmercury	9	3	0.23	115	0.00127	0.00127	0.34129
GO:0030241	skeletal muscle myosin thick filament assembly	10	3	0.26	132	0.00178	0.00178	0.45685
GO:0042632	cholesterol homeostasis	35	5	0.9	136	0.0019	0.0019	0.45685
GO:0120009	intermembrane lipid transfer	35	5	0.9	137	0.0019	0.0019	0.45685
GO:0050687	negative regulation of defense response to virus	3	2	0.08	139	0.00195	0.00195	0.45685
GO:1990074	polyuridylation-dependent mRNA catabolic process	3	2	0.08	140	0.00195	0.00195	0.45685
GO:0002574	thrombocyte differentiation	22	4	0.57	145	0.0022	0.0022	0.49028
GO:0098970	postsynaptic neurotransmitter receptor diffusion trapping	22	4	0.57	146	0.0022	0.0022	0.49028
GO:0030301	cholesterol transport	37	5	0.95	153	0.00245	0.00245	0.53299
GO:0007605	sensory perception of sound	115	9	2.96	161	0.00292	0.00292	0.62047
GO:0050885	neuromuscular process controlling balance	24	4	0.62	165	0.00306	0.00306	0.63544
GO:0040029	regulation of gene expression, epigenetic	140	6	3.6	1014	0.15316	0.00383	0.72906
GO:0006526	arginine biosynthetic process	4	2	0.1	173	0.00383	0.00383	0.72906
GO:0071233	cellular response to leucine	4	2	0.1	174	0.00383	0.00383	0.72906
GO:1901031	regulation of response to reactive oxygen species	4	2	0.1	175	0.00383	0.00383	0.72906
GO:0031936	negative regulation of chromatin silencing	13	3	0.33	178	0.004	0.004	0.73096
GO:0032525	somite rostral/caudal axis specification	13	3	0.33	179	0.004	0.004	0.73096
GO:0048814	regulation of dendrite morphogenesis	42	5	1.08	187	0.0043	0.0043	0.77037
GO:0031629	synaptic vesicle fusion to presynaptic active zone membrane	14	3	0.36	195	0.00499	0.00499	0.85642
GO:0046686	response to cadmium ion	14	3	0.36	196	0.00499	0.00499	0.85642
GO:0070212	protein poly-ADP-ribosylation	15	3	0.39	214	0.00612	0.00612	0.85642
GO:0098884	postsynaptic neurotransmitter receptor internalization	15	3	0.39	215	0.00612	0.00612	0.85642

GO:0016082	synaptic vesicle priming	33	5	0.85	123	0.00145	0.00615	0.85642
GO:0007249	I-kappaB kinase/NF-kappaB signaling	90	4	2.32	1149	0.20186	0.00615	0.85642
GO:0008611	ether lipid biosynthetic process	5	2	0.13	220	0.00628	0.00628	0.85642
GO:0010587	miRNA catabolic process	5	2	0.13	221	0.00628	0.00628	0.85642
GO:0019430	removal of superoxide radicals	5	2	0.13	222	0.00628	0.00628	0.85642
GO:0035630	bone mineralization involved in bone maturation	5	2	0.13	223	0.00628	0.00628	0.85642
GO:0043116	negative regulation of vascular permeability	5	2	0.13	224	0.00628	0.00628	0.85642
GO:0090131	mesenchyme migration	5	2	0.13	225	0.00628	0.00628	0.85642
GO:0090281	negative regulation of calcium ion import	5	2	0.13	226	0.00628	0.00628	0.85642
GO:1904059	regulation of locomotor rhythm	5	2	0.13	227	0.00628	0.00628	0.85642
GO:1990253	cellular response to leucine starvation	5	2	0.13	228	0.00628	0.00628	0.85642
GO:2000623	negative regulation of nuclear-transcribed mRNA catabolic process, nonsense-mediated decay	5	2	0.13	229	0.00628	0.00628	0.85642
GO:0032958	inositol phosphate biosynthetic process	16	3	0.41	250	0.0074	0.0074	0.99432
GO:0008654	phospholipid biosynthetic process	113	7	2.91	469	0.0269	0.00876	1
GO:0010996	response to auditory stimulus	32	4	0.82	263	0.00882	0.00882	1
GO:0045190	isotype switching	6	2	0.15	267	0.00926	0.00926	1
GO:0090141	positive regulation of mitochondrial fission	6	2	0.15	268	0.00926	0.00926	1
GO:0050790	regulation of catalytic activity	1556	39	40.05	2017	0.5937	0.00941	1

Table S13 Site locations, date of collection and sample size for all samples included in this study.

Population	Code	Sample size	Sample date	Longitude	Latitude
Yangtze River Estuary	YRE	21	2013.11.05	122.086°E	31.316°N
Luoma Lake	LM	18	2016.11.25	118.301°E	33.994°N
Hongze Lake	HZ	24	2016.11.22	118.845°E	33.295°N
Chaohu Lake	CH	24	2016.11.24	117.843°E	31.608°N
Taihu Lake	TH	24	2016.11.23	120.196°E	31.549°N
